# Photoredox C(2)-Arylation of Indole- and Tryptophan-Containing Biomolecules

**DOI:** 10.1021/acs.orglett.4c01019

**Published:** 2024-05-02

**Authors:** Bruno M. da S. Santos, Fernanda G. Finelli, David R. Spring

**Affiliations:** † Instituto de Pesquisas de Produtos Naturais, Universidade Federal do Rio de Janeiro, Rio de Janeiro 21941-599, Brazil; ‡ Yusuf Hamied Department of Chemistry, University of Cambridge, Cambridge CB2 1EW, U.K.

## Abstract

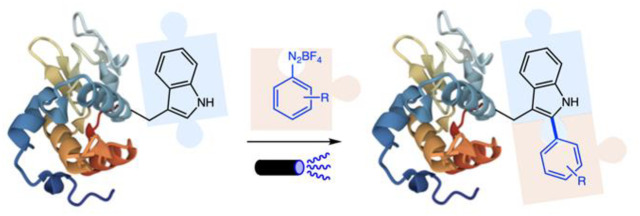

We introduce a novel and straightforward methodology for photoredox arylation of an indole scaffold using aryldiazonium salts under mild and metal-free conditions. Our approach enables the regioselective and chemoselective introduction of several aryl groups to the C(2) position of indoles and tryptophan, even in competition with other amino acids. This approach extends to the late-stage functionalization of peptides and lysozyme, heralding the unprecedented arylation of tryptophan residues in wild-type proteins and offering broad utility in chemical biology.

The indole moiety holds significant importance across medicinal chemistry, organic synthesis, and natural product research, mainly due to its remarkable pharmacological activities. Moreover, the presence of this scaffold in tryptophan (Trp) also makes the structure an attractive target for the highly selective modification of biomacromolecules. While Trp is the least abundant among canonical amino acids, comprising roughly 1% of the eukaryotic proteome, it simultaneously stands as a highly prevalent amino acid, being present in nearly 90% of all proteins.^1−5^

Traditional strategies for modifying biomacromolecules depend on unnatural amino acids incorporated into their structures through genetic engineering. However, novel biorthogonal approaches enable the specific modification of amino acid residues within fully formed wild-type proteins or peptides.^6−9^ Highly selective modification of biomacromolecules offers the potential, for instance, to modulate their activities, stabilities, or specificities; to incorporate fluorescent dyes, radioactive isotopes, or contrast agents to track cellular processes; or to modify biomaterials to improve their interactions with cells and tissues in regenerative medicine.^10−13^

Current strategies for the selective modification of Trp often rely on transition-metal-catalyzed C–H activation reactions,^14−19^ although several metal-free approaches have also been reported.^20−23^ It is noteworthy, however, that many of these methods involve intricate reaction mixtures or necessitate the use of harsh conditions, such as elevated temperatures, thereby limiting their application in more sensitive systems.

Photocatalysis has emerged as a powerful tool to promote challenging reactions, including late-stage C–H functionalization within complex molecular scaffolds, under mild conditions through controlled radical processes.^24−26^ Despite growing interest in this field, the development of operationally simple, selective, and efficient photochemical methods for covalent modifications of Trp residues within peptides and proteins remains a highly demanding endeavor.

Prior contributions include C–N and C–S functionalization of Trp residues of peptides and proteins,^27^ and C–C alkylation of Trp residues of peptides.^28,29^ Notably, the C–C functionalization of amino acid residues exhibits enduring stability, resisting hydrolysis even under harsh conditions. This resilience renders it an attractive strategy for protein and peptide modification. Preliminary studies suggest that aryl linkages exhibit stability under chemically stressing conditions, including acidic, basic, and oxidizing environments.^30^

In this scenario, aryldiazonium salts are excellent precursors of aryl radicals in photochemical processes with high reduction potentials, providing a wide range of chemical transformations under mild conditions.^31,32^ Particularly, most of the arene-diazonium tetrafluoroborate salts present good thermal stabilities and shelf lives, with many being commercially available. With due precaution and following reported protocols for the stability assessment of these chemicals, they can be used from laboratory to industrial scales.^33−35^

Considering the promising potential of photochemical strategies and the significance of the indole scaffold in diverse areas, we postulated that the indole core could react with aryldiazonium salts under biocompatible conditions in a metal-free photoredox platform. This straightforward methodology could enable the chemoselective arylation of Trp residues within general biomacromolecules, including wild-type proteins (Scheme 1).

**Scheme 1:**
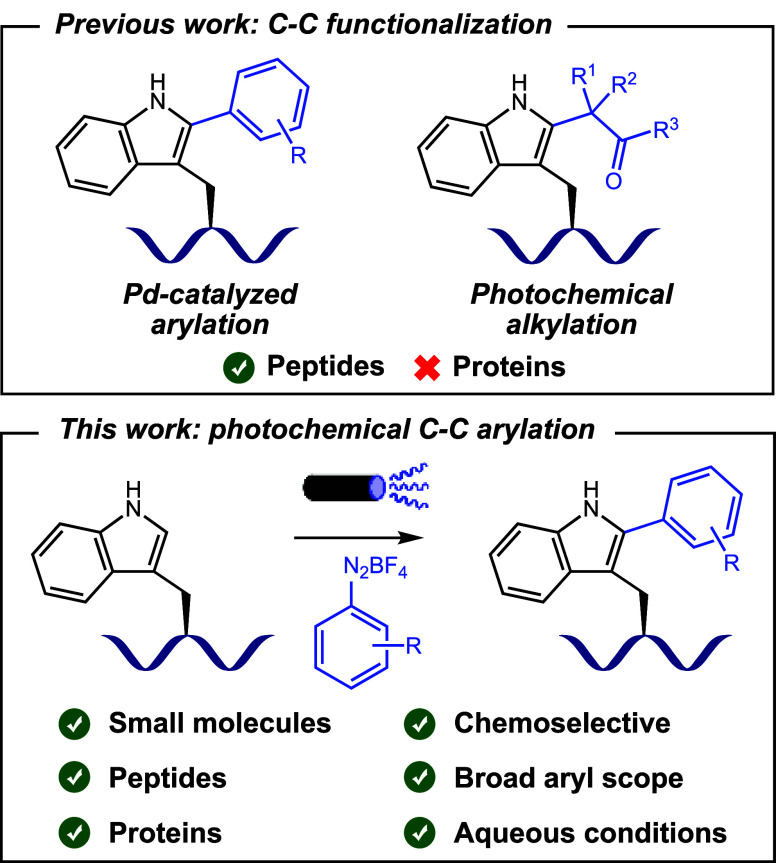
Modification of Trp Residues in Biomolecules

In our initial efforts to develop this idea, we observed that treatment of a highly nucleophilic indole, such as *N*-Me-indole, with aryldiazonium salt in the presence of an organophotocatalyst and a visible light source leads to mainly formation of C(3)-diazo aryl compounds. To avoid this undesired electrophilic aromatic substitution reaction, we modulated the indole nucleophilicity by employing *N*-Boc-indole (**1**). Upon overnight green LED irradiation, the mixture of indole **1**, aryldiazonium salt **2**, and 1 mol % eosin Y could effectively lead to the regioselective C(2)-arylation product **3** in 57% isolated yield (Table 1).

**Table 1:**
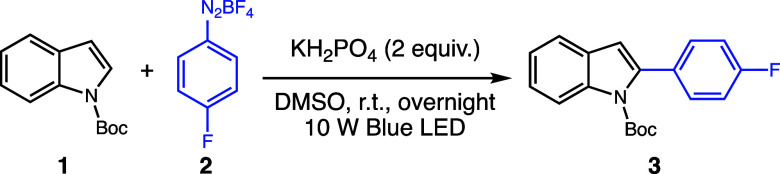
Control Experiments for C(2)-Arylation of *N*-Boc-indole (**1**)

entry	deviations	yield^a^
1	none	61%
2	no light	traces
3	adding 1 mol % eosin Y, green LED	57%
4	without eosin Y, green LED	51%
5	without base	54%
6	reaction time reduced to 3 h	60%

a Determined by ^1^H qNMR using 1,3-benzodioxole as the internal standard.

Further optimization steps revealed that the absence of eosin Y furnished the desired product in a 51% yield. The use of this photocatalyst, while accelerating the reaction kinetics, led to no substantial improvement in the reaction yield. Monobasic phosphate emerged as a suitable base, leading to a modest improvement in the reaction yield and a cleaner reaction mixture. For full optimization details, see the Supporting Information.

We then proceeded to evaluate the generality of the reaction scope (Scheme 2, left). The electron-poor diazonium salts **4**–**7** exhibited exceptional reactivity. On the other hand, the electron-rich **12**–**15** delivered more modest results. This trend can be ascribed to the superior reduction potential of the electron-deficient diazonium salts, facilitating the generation of reactive aryl radicals. Notably, the use of the *p*-nitro-substituted diazonium salt required a lower LED power input for optimal results, as higher power levels led to a considerable decrease in yield, likely due to diazonium salt degradation. The presence of halogen substituents in diazonium salts was also well-tolerated (**3**, **10**, and **11**) leading to products in good to excellent isolated yields. We also evaluated the influence of steric hindrance on the outcome of the reaction. Both *meta*- and *ortho*-trifluoromethyl-substituted diazonium salts were found to be amenable to the reaction, exhibiting yields comparable to their *para*-substituted counterpart (**7**–**9**). In general, we could observe other arylated regioisomers during the studies, but only in trace amounts. However, when the C(2) position is compromised, C(3)-arylation proceeds smoothly with reduced yields (**18**). Finally, the reaction employing free 1*H*-indole was highly exothermic and instantaneous, causing decomposition of the starting materials (**19**).

**Scheme 2:**
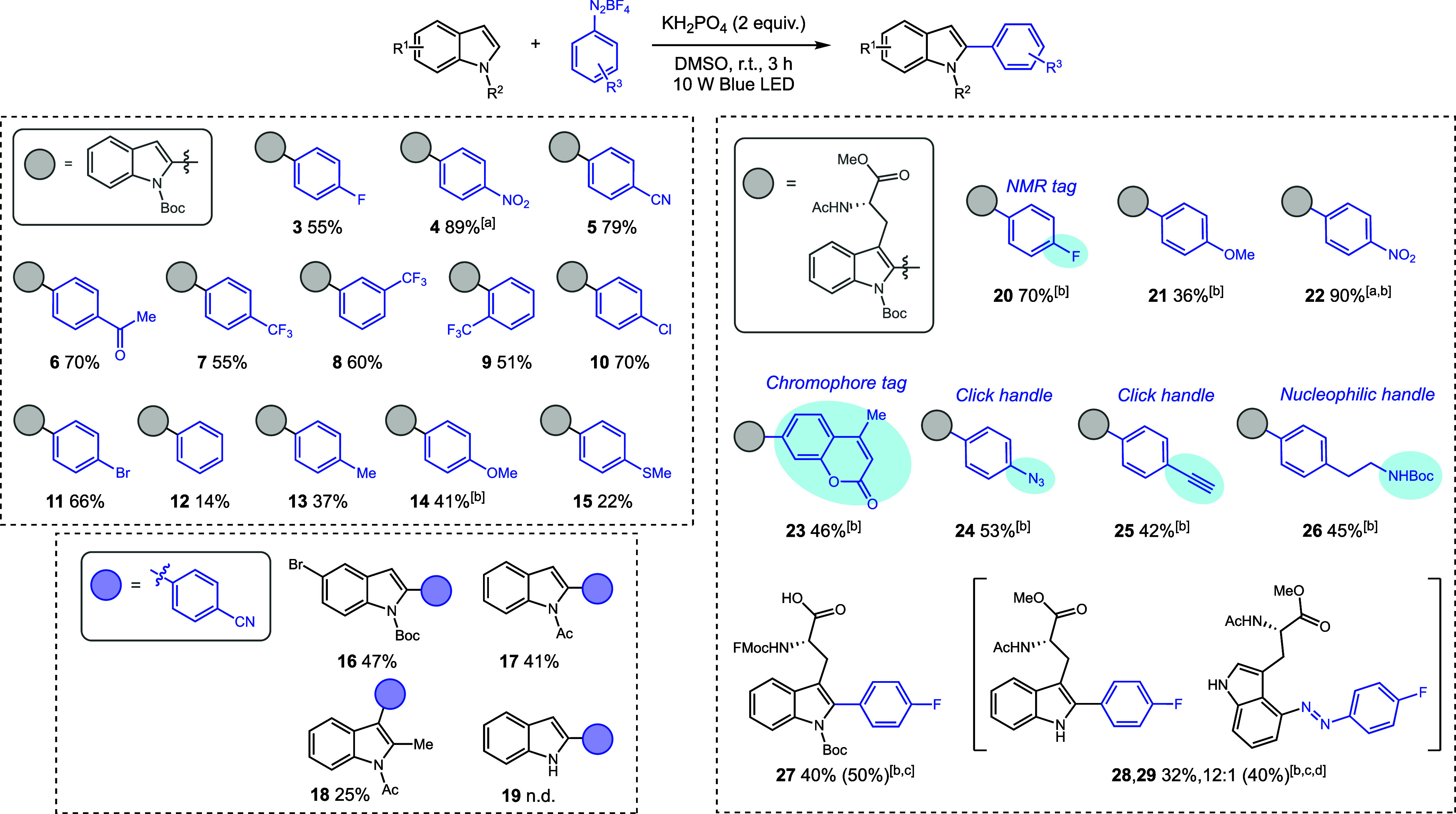
Photoredox C(2)-Arylation of the Indole Motif Irradiation performed with 1 W blue LED power instead of 10 W. Overnight reaction. Recovered starting material in brackets. Determined by ^1^H qNMR using 1,3-benzodioxole as the internal standard. Isolated yields.

Next, we advanced to undertake the arylation of Ac-Trp(Boc)-OMe (Scheme 2, right). This endeavor yielded a series of arylated tryptophan derivatives, featuring notably complex structures such as the coumarin derivative **23** alongside readily functionalizable compounds bearing azide, alkynyl, and ethylamino substituents (**24**–**26**) in commendable isolated yields. These results indicate the feasibility of performing these reactions to introduce more complex molecules into the indole motif using the aryl group as a linker in bioconjugation strategies. To explore the selectivity of the reaction, we undertook the arylation of the free-acid **27** and the unprotected indole derivative **28**. The undesired aryl diazo product **29** is not generated significantly when the more nucleophilic C(3) position of the indole is substituted. Furthermore, chiral HPLC analysis conclusively demonstrated the absence of racemization, proving that this reaction is suitable for keeping the stereochemical integrity of the synthesized compounds (see Supporting Information, section 5).

To extend the utility of this methodology to more complex peptide systems, we conceived a competition study aimed at elucidating the selectivity of Trp arylation in the presence of other amino acids with either reactive or structurally similar side chains. In this regard, we conducted reactions using aryldiazonium salt **2** and equimolar mixtures of Ac-Trp(Boc)-OMe and each of the following amino acids individually: Ac-Phe-OMe, Ac-His-OMe, Ac-Tyr-OMe, Ac-Pro-OMe, Ac-Ser-OMe, and Ac-Cys-OMe. The results were analyzed by ^19^F NMR spectroscopy (see Supporting Information, section 6).

For most of the evaluated amino acids, we did not observe any significant competition, affirming the chemoselective arylation of Trp within the final reaction mixtures. Consistent with prior literature reports,^36,37^ Cys exhibited high reactivity toward the arylation of its sulfur moiety with a 3:1 ratio of the Ar-Cys to Ar-Trp products, respectively. Notably, the utilization of *S*-protected Cys eliminated any observable competition, and arylated Trp was the only detectable product in the final reaction mixture. These findings demonstrate that peptides containing any combination of these amino acids will indeed undergo the chemoselective C(2)-arylation of their Trp residues. Moreover, they highlight the robustness of this methodology as a powerful tool for bioconjugation strategies, particularly considering that Cys often participates in disulfide bonds within biomolecules, rendering it unreactive as a competitive arylation site.

Based on these results, we designed the model peptide Fmoc-FC^Trt^W^Boc^S^*t*Bu^A-OH, prepared by automated peptide synthesis. The Trp residue was strategically positioned in the middle of the peptide chain, as a nonterminal residue, to simulate conditions of increased steric hindrance. After some necessary adjustments and reaction condition optimizations, the C(2)-arylation of the Trp residue in peptides **30**–**33** was successfully accomplished after an overnight reaction using 10 equiv of different aryldiazonium salts and 40 W blue LED irradiation (Scheme 3, left).

**Scheme 3:**
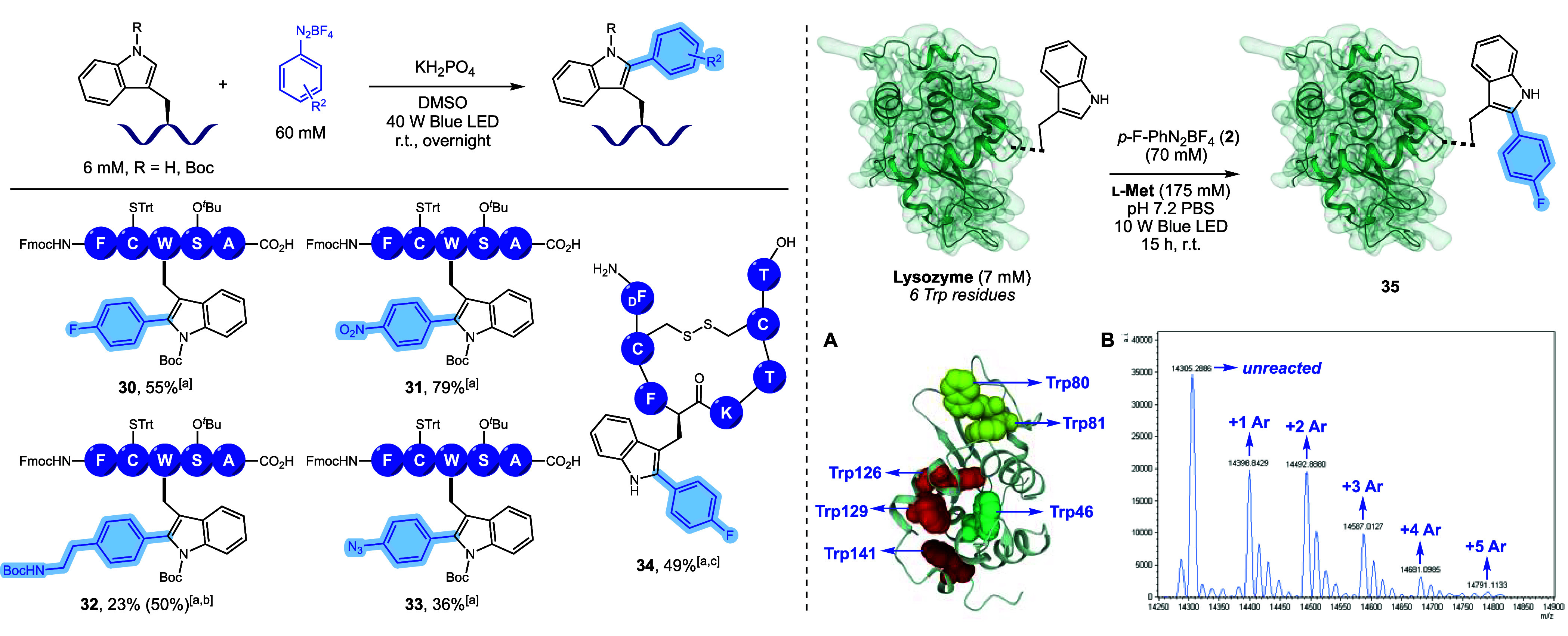
Photoredox C(2)-Arylation of Peptides and Lysozyme Isolated yield. Recovered starting material in brackets. Three hours of reaction. (A) Trp residue positions in the protein structure. Arylation was detected in all these residues. (B) Deconvoluted intact mass spectra of the reaction mixture.

In a proof-of-concept demonstration, we applied our methodology to octreotide acetate, a somatostatin analogue containing an unprotected Trp residue. After 3 h, we achieved the C(2)-arylated product **34** in 49% isolated yield (see Supporting Information, section 6.4).

The originality and robustness of the methodology were also demonstrated by the late-stage functionalization of chicken HEW lysozyme, a 14 kDa enzyme containing six Trp residues and four disulfide bonds accounting for all the Cys residues in its structure. We have accomplished the C(2)-arylation of Trp residues using 7 mM enzyme and 70 mM aryldiazonium salt **2** in PBS, 10 W blue LED, and 175 mM l-methionine as a sacrificial antioxidant of Met residues in the enzyme. Analysis of product **35** at the intact level via mass spectrometry indicated that the desired arylation of the biomacromolecule was successfully performed. We observed a mixture of proteins with varying degrees of arylation ranging from one to five arylated residues in the structure (Scheme 3, right).

Subsequent trypsin digestion combined with a peptide mapping strategy conclusively confirmed that the C(2)-arylation occurred exclusively at the six Trp residues to different degrees. Interestingly, we did not observe any products featuring simultaneous arylation of the adjacent Trp-80 and Trp-81 residues, suggesting that the arylation of one of these residues somehow hinders the reaction at the other, potentially due to increased steric hindrance or deactivating π-stacking interactions.

A series of experiments was designed to elucidate the reaction mechanism. The investigation pointed to the formation of a photoexcitable EDA complex between the indole moiety and aryldiazonium salts as the key step for the generation of aryl radicals. In support of this hypothesis, a UV–vis absorbance experiment revealed a bathochromic shift when comparing the mixture of **1** and **2** to their isolated solutions. Subsequent ^1^H NMR and ^1^H–^15^N HMBC analyses did not detect any HMBC correlations between indole hydrogens and diazonium salt nitrogen, with no discernible changes in covalent connectivity compared with the isolated materials.

A ^1^H NMR titration experiment further bolstered it by demonstrating a downfield shift in the signals of both **6** and **7** upon being mixed in varying proportions, indicative of EDA complex formation. Job’s plot analysis, using ^1^H NMR and UV–vis data, consistently pointed toward a complex stoichiometry of 1:1 (see Supporting Information, section 4, for details).

ON/OFF experiments demonstrated that the product formation was solely observed during light irradiation. Conversely, diazonium salt degradation exhibited a constant rate seemingly independent of light exposure. These observations suggest that the arylation mechanism proceeds predominantly during light exposure, while a concurrent side reaction, responsible for diazonium salt degradation, occurs continually and independently of light. These reactions compete for the achievement of satisfactory product yields.

No detectable product was observed in a radical-trapping experiment with TEMPO, providing compelling evidence of a radical mechanism governing the reaction. Furthermore, the benzylic radical **39** could be detected by HRMS analysis.

Based on these findings, a plausible reaction mechanism is proposed in Scheme 4. The sequence commences with the formation of an EDA complex **36** between the indole moiety, acting as the donor, and diazonium salt, serving as the acceptor. Upon excitation, this complex undergoes electron transfer from **1** to **2**, which, once reduced decomposes into nitrogen gas and the aryl radical **37**. This reactive intermediate subsequently adds to another indole unit selectively at the C(2) position, yielding the C(3) benzylic radical **39**.^38^ Oxidation of this radical by the indole radical cation **38** leads to the product after elimination and the reestablishment of aromaticity. As previously stated, kinetic measurements show that the presence of eosin Y increases the reaction rates, indicating a change in the reaction mechanism when this photocatalyst is present.

**Scheme 4:**
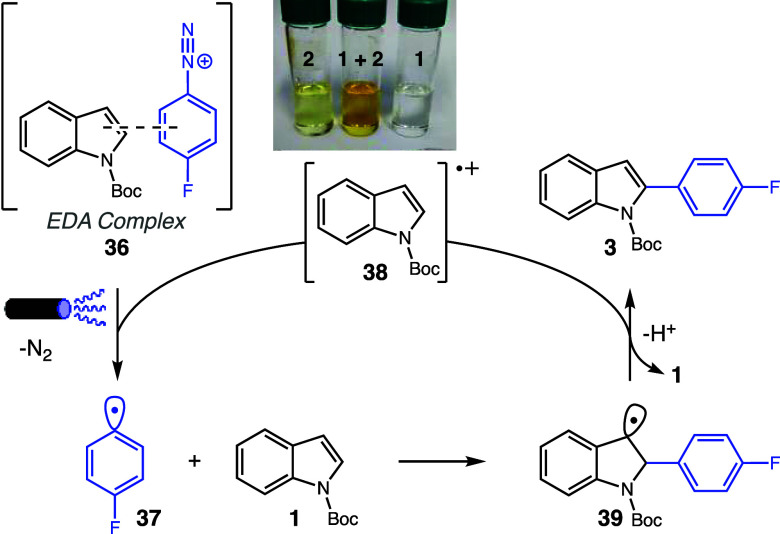
Plausible Reaction Mechanism

Herein, we introduce a straightforward metal-free methodology for photoinduced C(sp^2^)–C(sp^2^) arylation of indoles with aryldiazonium salts, enabling the chemoselective modification of Trp residues within peptides and lysozyme. These results demonstrate the unprecedented arylation of Trp residues in wild-type proteins, highlighting the significance of this method for bioconjugation. The aromatic ring serves as a reliable and stable linker for the attachment to complex structures, making it versatile for a multitude of prospective applications. The simplicity of the methodology, devoid of complex or costly reagents, not only positions it as a platform for the selective late-stage functionalization of Trp residues in biomacromolecules but also extends its utility to indole-containing molecules in general.

## Data Availability

The data underlying this study are available in the published article and its Supporting Information.

## Supporting Information Available

Additional experimental details, characterization data, and spectra (PDF)
