# Supplementary material for: Photoredox C(2)-Arylation of Indole- and Tryptophan-Containing Biomolecules
Source: Org Lett. 2024 May 2;26(19):4065–70. doi: 10.1021/acs.orglett.4c01019 (PMC11194849; doi:10.1021/acs.orglett.4c01019)
Supplement: Supplementary file 1 — ol4c01019_si_001.pdf [file ol4c01019_si_001.pdf]

Supporting Information

Photoredox C(2)-Arylation of Indole and Tryptophan-Containing Biomolecules

Bruno M. da S. Santos,[a] Fernanda G. Finelli*[a,b] and David R. Spring[b]

[a] Instituto de Pesquisas de Produtos Naturais, Universidade Federal do Rio de Janeiro, Rio de Janeiro, Brazil; [b] Yusuf Hamied Department of Chemistry, University of Cambridge, Cambridge, UK

*Corresponding author: finelli@ippn.ufrj.br

Table of Contents

1. GENERAL INFORMATION 3
1.1. MATERIALS AND INSTRUMENTS 3
2. METHOD OPTIMIZATION: INDOLE ARYLATION 3
2.1. GENERAL PROCEDURE FOR REACTION OPTIMIZATION STUDIES 3
2.2. REACTION OPTIMIZATION RESULTS 4
3. SYNTHETIC PROCEDURES 5
3.1. ARYL DIAZONIUM SALT SYNTHESIS 5
3.1.1. GENERAL PROCEDURE[1] 5
3.1.2. EXPERIMENTAL DETAILS FOR ARYL DIAZONIUM SALTS 6
3.2. PROTECTED INDOLES SYNTHESIS 9
3.2.1. GENERAL PROCEDURE: *N*-BOC PROTECTION OF INDOLES 9
3.2.2. EXPERIMENTAL DETAILS FOR PROTECTED INDOLES 9
3.3. ARYLATED INDOLES SYNTHESIS 11
3.3.1. GENERAL PROCEDURE FOR PHOTOCHEMICAL INDOLE ARYLATION REACTION 11
3.3.2. EXPERIMENTAL DETAILS FOR ARYLATED INDOLES 11
4. MECHANISTIC EXPERIMENTS 20
4.1. EVIDENCE OF EDA COMPLEX FORMATION: 20
4.2. ^{1}H NMR TITRATION EXPERIMENT 22
4.3. DETERMINATION OF EDA COMPLEX STOICHIOMETRY 23
4.4. ON/OFF EXPERIMENTS 24
4.5. RADICAL TRAPPING WITH TEMPO 25
4.6. MECHANISTIC HYPOTHESIS 26
4.7. KINETIC EVIDENCE FOR MECHANISM SWITCH IN THE PRESENCE OF EOSIN Y PHOTOCATALYST 26
4.8. QUANTUM YIELD MEASUREMENT 27
5. HPLC TRACES 29
5.1. OPTICAL PURITY OF **20** 29
6. AMINO ACID COMPETITION EXPERIMENT 31
7. PEPTIDE SUBSTRATES REACTION AND CHARACTERIZATION 32
7.1. MODEL PEPTIDE **64** SYNTHESIS 32
7.2. MODEL PEPTIDE ARYLATION REACTION OPTIMIZATION 33
7.3. SCOPE OF ARYL DIAZONIUM SALTS FOR THE ARYLATION OF MODEL PEPTIDE 64 34
7.4. ARYLATION OF OCTREOTIDE 39
8. ENZYME SUBSTRATE REACTION AND CHARACTERIZATION 41
8.1. LYSOZYME ARYLATION PROTOCOL 41
8.2. PRODUCT CHARACTERIZATION – WITHOUT METHIONINE ADDITION 41
8.3. PRODUCT CHARACTERIZATION – WITH ADDITION OF L-METHIONINE AS ANTIOXIDANT 54
9. NMR SPECTRA 55
10. REFERENCES 107

1. General Information

1.1. Materials and Instruments

Solvents and commercially available reagents were used without further purification, unless otherwise stated. Solvents and chemicals were purchased from Sigma-Aldrich, Acros Organics or Fluorochem. Octreotide Acetate (>99%) was purchased from TargetMol. Reactions were purged and performed under nitrogen or argon atmosphere.

Thin layer chromatography (TLC) was performed on Silicycle F-254 aluminum sheets coated with silica gel and visualized with UV light quenching and/or chemical staining with phosphomolybdic acid or Ceric Ammonium Molybdate (CAM).

Standard chromatographic purification was accomplished through flash chromatography on silica gel (60 Å, 230-400 Mesh) with mobile phases described in each experimental procedure.

Reverse-phase chromatography was performed on an automated Biotage® Isolera One™ using a Snap Ultra C18 12 g column.

Preparative HPLC purification was carried out on an Agilent 1260 Infinity or a Shimadzu Corp. Nexera 2 equipment.

HPLC analysis were conducted on an Agilent 1260 Infinity system or on a Shimadzu Prominence LC-20A with a PDA detector.

UV-LCMS analysis was performed on a Waters ACQUITY H-Class UPLC with ESCi Multi-Mode Ionization Waters SQ Detector 2 spectrometer.

LC-MS/MS analysis of tryptic peptides from protein digestion samples were performed on a Bruker NanoElute2 equipped with a PepSep C18 column (15 cm x 75 μm, 1.9 μm), coupled to a Bruker Maxis Impact ESI-Q-TOF mass spectrometer.

High resolution mass spectrometry (HRMS) measurements were recorded using either a Micromass Q-TOF mass spectrometer, a Waters LCT Premier Time of Flight mass spectrometer or a Bruker solariX XR 7T with ESI/FT-ICR configuration.

^{1}H, ^{19}F and ^{13}C NMR spectra were recorded either on a Bruker DPX-400, a Bruker Avance III 500 MHz HD Smart Probe, a Varian MR-400 or a Varian VNMRSYS-500 (400 or 500 MHz for ^{1}H, 470 or 376 MHz for ^{19}F and 101 or 126 MHz for ^{13}C). Chemical shifts are expressed as parts per million (ppm) with TMS or residual protic deuterated solvent as internal standard. Coupling constants are expressed in Hertz, with multiplicity expressed using standard abbreviations. In ^{13}C NMR data, reported multiplicities are related to carbon-fluorine coupling. ^{1}H NMR determined yields were obtained using 1,3-Benzodioxole as internal standard and using a relaxation delay of 40 seconds and observe pulse of 90 degrees.

Infrared (IR) spectra were collected either on a Shimadzu IRaffinity1 or on a Perkin-Elmer Spectrum One spectrometer equipped with an ATR probe.

Irradiation setup: Reactions promoted by light were carried out in EvoluChem™ PhotoRedOx Box equipped with a 40 W Kessil A160WE tuna blue light with peak emission in the region of 450-460 nm. Unless otherwise stated, the LED power was set to 10 W via manual control. The reactions were irradiated inside the EvoluChem device using a holder for 8 x 4 mL borosilicate vials, positioned approximately 10 cm away from the light source.

Automated peptide synthesis was carried out on a CEM Liberty Automated Microwave Peptide Synthesizer.

2. Method Optimization: Indole Arylation

2.1. General Procedure for reaction optimization studies

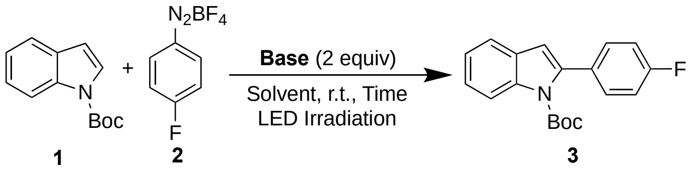

Scheme S1: Model reaction for optimization studies.

A 5 mL glass vial was charged with and adequate magnetic stirring bar, *tert*-butyl 1H-indole-1-carboxylate (326 mg, 1.5 mmol, 5 equiv.), base (0.6 mmol, 2 equiv.) and photocatalyst (3 μmol, 1 mol%). 1 mL of solvent was added, and the mixture was stirred for a couple of minutes before 4-fluorobenzenediazonium tetrafluoroborate (36 mg, 0.3 mmol, 1 equiv.) were added at once. The vial was closed, and the mixture was sparged with argon for 15 minutes in the dark. After sparging, the vial was sealed with parafilm®, keeping the argon balloon attached to it. The reaction was then irradiated with a 10 W Blue LED (450-460 nm) for the required time. After the reaction time, reaction vial was opened to air and 0.3 mmol of 1,3-benzodioxole was added as internal standard. After complete homogenization, an NMR sample was prepared with 50 mL of the reaction mixture, 100 μL of DMSO and 400 μL of $CDCl_3$ to determine reaction yields.

An inversion recovery sequence was applied to a representative sample, and the longest T1 relaxation time was measured as 8 seconds. In order to have a suitable NMR yield determination, every sample in the optimization study was analyzed using a relaxation delay of 40 seconds (5 x T1 relaxation time)..

2.2. Reaction optimization results

Reaction with electron-rich indoles: We first explored our idea by employing a highly nucleophilic indole core. *N*-methylindole (**40**), 4-chlorobenzene diazonium salt (**41**), and 1 mol% of Eosin Y in DMSO were exposed to a 10 W green LED and after 2 hours we detected a mixture of **43** and **44** in 23% and 19% yield, respectively, with total consumption of diazonium salt. After performing this reaction in the dark, we observed the major formation of diazo aryl product **43**, thus proving this side reaction occurs through a thermal pathway.

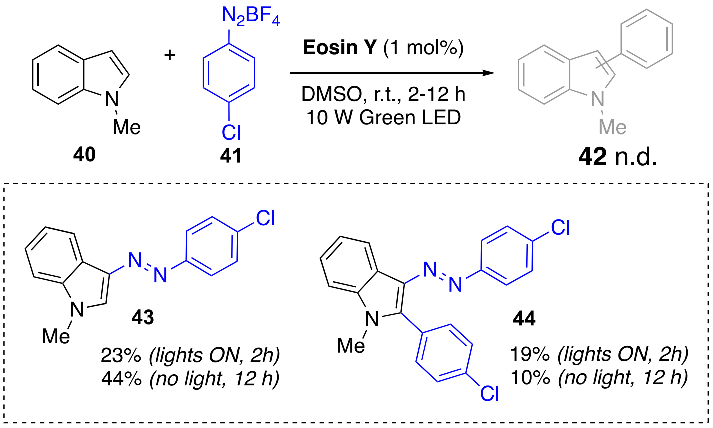

Scheme S2: Reaction with electron-rich indole.

Initial conditions: Our preliminary studies pointed towards this standard reaction conditions. Reaction was performed using the general procedure described in section 2.1. No base or photocatalyst were used in the initial conditions, and the reaction was irradiated for 15 hours. The effects of variations are depicted in the table below. Changes that cause a significant positive impact in reaction yields are highlighted in blue font. Subsequent entries in the table incorporate this variation to their procedure.

Table S1: Initial reaction conditions and optimization studies

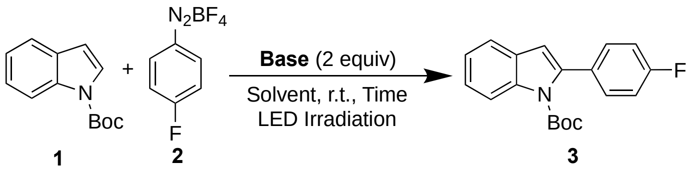

Entry	Deviation from initial conditions	Yield of 8
1	None	54%
2	Using 1 mol% of Eosin Y disodium salt and Green LED	57%
3	Using 1 mol% Rhodamine B and Green LED	41%
4	Using 1 mol% of 4CzIPN	30%
	Base Study (2 equiv., No photocatalyst)	
5	DABCO	12%
6	DIPEA	20%
7	K_2CO_3	29%
8	$NaHCO_3$	40%
9	$CsHCO_3$	27%
10	NaOAc	12%
11	NH_4OAc	9%
12	$(NH_4)_2SO_4$	51%
13	K_2HPO_4	33%
**14**	**KH_2PO_4**	**61%**

	Solvent Study (No photocatalyst)	
15	Acetone	32%
16	CH_3CN	18%
17	MeOH	22%
18	CH_2Cl_2	6%
19	1,4-Dioxanes	7%
21	DMSO + 10 equiv. of Water	57%
22	DMSO +20 equiv. of Water	38%
	Stoichiometry Study (Indole / Diazonium salt)	
23	3 : 1.	41%
24	2 : 1	34%
25	1 : 5	41%
	LED Irradiation	
26	10 W Green LED (515-525 nm)	51%
27	10 W Purple LED (395-400 nm)	52%
28	10 W White LED	61%
	**Effect of Oxygen**	
29	Without argon sparge	50%
	Time Study	
30	20 minutes	20%[a]
31	60 minutes	44%[a]
**32**	**3 hours**	**55%[a,b]**

[a]Yield determined based on a 50 μL aliquot of reaction taken in each time. The corresponding amount of 1,3-benzodioxole was added to each of these aliquots. [b]After 3 hours we observed total consumption of the limiting starting material.

3. Synthetic Procedures

3.1. Aryl Diazonium salt synthesis

3.1.1. General procedure[1]

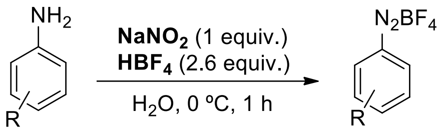

Scheme S3: Aryl diazonium salt synthesis.

To an ice-cooled Erlenmeyer flask containing a suspension of the respective aniline (10 mmol, 1 equiv.) in water (4.0 mL, 2.5 M) was added dropwise a 48% w/w tetrafluoroboric acid solution (3.4 mL, 26 mmol, 2.6 equiv.) under vigorous magnetic stirring. A cold solution of sodium nitrite (690 mg, 10 mmol, 1 equiv.) in water (2.0 mL, 5.0 M) was very slowly added, in a rate of 1 drop per 3 seconds. Reaction was stirred for 1 hour at 0 ºC before the diazonium salt was filtered off. The filtered solid was recrystallized from acetone and ethyl ether (1:1) to yield the pure diazonium salt after removing excess solvent from the solid in high vacuum.

Use and Storage: Diazonium salts should be stored in a capped vial, covered in aluminum foil, in the freezer. Typically, they can be used for a month before further recrystallization is necessary. Light stability tests: The diazonium salts were first prepared in small scale and a small amount was submitted to light stability tests. Both the dry solid and a 1 M DMSO solution were submitted to irradiation of a 10 W Blue LED for 1 hour with cooling fans. We did not observe any significant changes in temperature or visual aspects as well as any vigorous gas evolution.

3.1.2. Experimental details for Aryl Diazonium salts

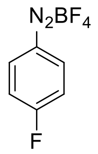

4-fluorobenzenediazonium tetrafluoroborate (2): Synthesized according to general procedure using 4-fluoroaniline (0.95 mL, 10 mmol). The title compound was isolated as a white solid (1.6 g, 75% yield). Spectral data matches previous literature.[1]

^{1}H NMR (500 MHz, D_2O) δ 8.74 (dd, J = 9.4, 4.3 Hz, 2H), 7.76 (dd, J = 9.3, 8.0 Hz, 2H).

^{13}C NMR (126 MHz, D_2O) δ 169.9 (d, J = 271.2 Hz), 136.4 (d, J = 12.6 Hz), 120.1 (d, J = 25.3 Hz), 109.6.

^{19}F NMR (470 MHz, D_2O) δ -81.65 – -85.53 (m), -150.28, -150.33 (Boron isotopic effect on BF_4^- signal).

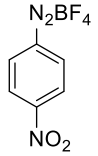

4-nitrobenzenediazonium tetrafluoroborate (45): Synthesized according to general procedure using 4-nitroaniline (1.4 g, 10 mmol). The title compound was isolated as a light-yellow solid (1.6 g, 70% yield). Spectral data matches previous literature.[1]

^{1}H NMR (500 MHz, D_2O) δ 8.94 (d, J = 9.3 Hz, 2H), 8.78 (d, J = 9.3 Hz, 2H).

^{13}C NMR (126 MHz, D_2O) δ 153.9, 134.1, 126.55, 120.5.

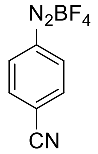

4-cyanobenzenediazonium tetrafluoroborate (46): Synthesized according to general procedure using 4-aminobenzonitrile (1.2 g, 10 mmol). The title compound was isolated as a white solid (1.4 g, 65% yield). Spectral data matches previous literature.[2]

^{1}H NMR (500 MHz, D_2O) δ 8.82 (d, J = 9.1 Hz, 2H), 8.39 (d, J = 9.1 Hz, 2H).

^{13}C NMR (126 MHz, D_2O) δ 135.4, 132.6, 123.4, 119.4, 116.0.

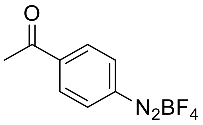

4-acetylbenzenediazonium tetrafluoroborate (47): Synthesized according to general procedure using 4-aminoacetophenone (1.4 g, 10 mmol). The title compound was isolated as a white solid (1.2 g, 50% yield). Spectral data matches previous literature.[3]

^{1}H NMR (500 MHz, D_2O) δ 8.76 (d, J = 8.9 Hz, 2H), 8.46 (d, J = 8.9 Hz, 2H), 2.80 (s, 3H).

^{13}C NMR (126 MHz, D_2O) δ 199.8, 145.7, 132.7, 130.8, 118.0, 26.7.

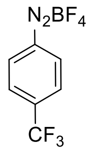

4-(trifluoromethyl)benzenediazonium tetrafluoroborate (48): Synthesized according to general procedure using 4-(trifluoromethyl)aniline (1.6 g, 10 mmol). The title compound was isolated as a white solid (1.7 g, 65% yield). Spectral data matches previous literature.[2]

^{1}H NMR (500 MHz, Acetone) δ 9.03 (d, J = 9.2 Hz, 2H), 8.41 (d, J = 9.1 Hz, 2H).

^{13}C NMR (126 MHz, Acetone) δ 139.9 (q, J = 33.9 Hz), 134.0 (s), 128.6 (q, J = 3.9 Hz), 123.5 (s), 120.9 (q, J = 91.4 Hz).

^{19}F NMR (470 MHz, D_2O) δ -64.31, -150.79, -150.84. (Boron isotopic effect on BF_4^- signal).

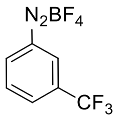

3-(trifluoromethyl)benzenediazonium tetrafluoroborate (49): Synthesized according to general procedure using 3-(trifluoromethyl)aniline (1.6 g, 10 mmol). The title compound was isolated as a white solid (1.7 g, 65% yield). Spectral data matches previous literature.[1]

^{1}H NMR (500 MHz, D_2O) δ 9.04 (s, 1H), 8.87 (d, *J* = 8.4 Hz, 1H), 8.64 (d, *J* = 8.1 Hz, 1H), 8.20 (t, *J* = 8.3 Hz, 1H).

^{13}C NMR (126 MHz, D_2O) δ 138.4 (q, *J* = 3.2 Hz), 135.5, 133.2 (q, *J* = 35.6 Hz), 132.9, 129.6 (q, *J* = 4.0 Hz), 121.6 (q, *J* = 272.8 Hz), 116.1.

^{19}F NMR (470 MHz, D_2O) δ -63.62, -150.39, -150.44. (Boron isotopic effect on BF_4^- signal).

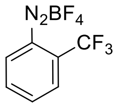

2-(trifluoromethyl)benzenediazonium tetrafluoroborate (50): Synthesized according to general procedure using 2-(trifluoromethyl)aniline (1.6 g, 10 mmol). The title compound was isolated as a white solid (1.3 g, 50% yield). Spectral data matches previous literature.[1]

^{1}H NMR (500 MHz, D_2O) δ 8.95 (d, *J* = 8.3 Hz, 1H), 8.54 (t, *J* = 7.9 Hz, 1H), 8.42 (d, *J* = 8.0 Hz, 1H), 8.29 (t, *J* = 8.1 Hz, 1H).

^{13}C NMR (126 MHz, D_2O) δ 142.6, 135.4, 135.3, 130.7 (q, *J* = 3.7 Hz), 130.0 (q, *J* = 36.1 Hz), 120.5 (q, *J* = 273,7 Hz), 111.6.

^{19}F NMR (470 MHz, D_2O) δ -60.83, -150.40, -150.45. (Boron isotopic effect on BF_4^- signal).

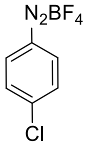

4-chlorobenzenediazonium tetrafluoroborate (41): Synthesized according to general procedure using 4-chloroaniline (1.3 g, 10 mmol). The title compound was isolated as a white solid (1.5 g, 65% yield). Spectral data matches previous literature.[1]

^{1}H NMR (500 MHz, D_2O) δ 8.60 (d, *J* = 9.2 Hz, 2H), 8.04 (d, *J* = 9.2 Hz, 2H).

^{13}C NMR (126 MHz, D_2O) δ 149.4, 133.5, 132.3, 112.2.

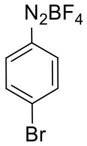

4-bromobenzenediazonium tetrafluoroborate (51): Synthesized according to general procedure using 4-bromoaniline (1.7 g, 10 mmol). The title compound was isolated as a white solid (1.6 g, 60% yield). Spectral data matches previous literature.[3]

^{1}H NMR (500 MHz, D_2O) δ 8.49 (d, *J* = 9.1 Hz, 2H), 8.21 (d, *J* = 9.1 Hz, 2H).

^{13}C NMR (126 MHz, D_2O) δ 138.9, 135.3, 133.0, 112.8.

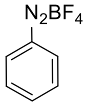

Benzenediazonium tetrafluoroborate (52): Synthesized according to general procedure using aniline (0.9 g, 10 mmol). The title compound was isolated as a white solid (1.3 g, 70% yield). Spectral data matches previous literature.[1]

^{1}H NMR (500 MHz, D_2O) δ 8.62 – 8.57 (m, 2H), 8.32 (t, *J* = 7.7 Hz, 1H), 8.03 – 7.96 (m, 2H).

^{13}C NMR (126 MHz, D_2O) δ 141.8, 132.1, 131.7, 115.3.

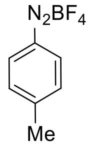

4-methylbenzenediazonium tetrafluoroborate (53): Synthesized according to general procedure using *p*-toluidine (1.1 g, 10 mmol). The title compound was isolated as a white solid (1.3 g, 65% yield). Spectral data matches previous literature.[1]

^{1}H NMR (500 MHz, D_2O) δ 8.46 (d, *J* = 8.8 Hz, 2H), 7.81 (d, *J* = 8.6 Hz, 2H), 2.66 (s, 3H).

^{13}C NMR (126 MHz, D_2O) δ 156.4, 132.4, 132.1, 109.7, 22.3.

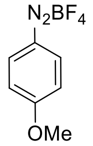

4-methoxybenzenediazonium tetrafluoroborate (54): Synthesized according to general procedure using 4-methoxyanilne (1.2 g, 10 mmol). The title compound was isolated as a white solid (1.4 g, 65% yield). Spectral data matches previous literature.[1]

^{1}H NMR (500 MHz, D_2O) δ 8.51 (d, *J* = 9.5 Hz, 2H), 7.43 (d, *J* = 9.5 Hz, 2H), 4.12 (s, 3H).

^{13}C NMR (126 MHz, D_2O) δ 170.2, 135.5, 117.8, 101.0, 57.3.

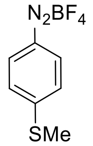

4-(methylthio)benzenediazonium tetrafluoroborate (55): Synthesized according to general procedure using 4-methylthioanilne (1.4 g, 10 mmol). The title compound was isolated as a white solid (1.5 g, 65% yield). Spectral data matches previous literature.[2]

^{1}H NMR (500 MHz, D_2O) δ 8.34 (d, *J* = 9.0 Hz, 2H), 7.72 (d, *J* = 9.0 Hz, 2H), 2.70 (s, 3H).

^{13}C NMR (126 MHz, D_2O) δ 161.7, 131.5, 126.8, 104.7, 14.1.

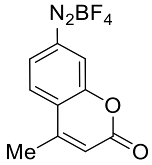

4-methyl-2-oxo-2H-chromene-7-diazonium tetrafluoroborate (56): Synthesized according to general procedure using 7-amino-4-methyl-2H-chromen-2-one (1.8 g, 10 mmol). The title compound was isolated as a white solid (1.4 g, 52% yield). Spectral data matches previous literature.[6]

^{1}H NMR (400 MHz, DMSO) δ 8.81 (s, 1H), 8.65 (d, *J* = 7.9 Hz, 1H), 8.37 (d, *J* = 8.3 Hz, 1H), 6.91 (s, 1H), 2.57 (s, 4H).

^{13}C NMR (101 MHz, DMSO) δ 157.8, 151.9, 151.2, 129.7, 128.4, 127.4, 120.6, 120.2, 116.9, 18.0.

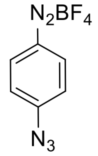

4-azidobenzenediazonium tetrafluoroborate (57): Synthesized according to general procedure using 4-azidoanilne (1.3 g, 10 mmol). The title compound was isolated as a white solid (2.0 g, 84% yield). Spectral data matches previous literature.[4]

^{1}H NMR (400 MHz, D_2O) δ 8.53 (d, *J* = 8.7 Hz, 1H), 7.56 (d, *J* = 8.7 Hz, 1H).

^{13}C NMR (101 MHz, D_2O) δ 155.2, 134.5, 122.1, 106.0.

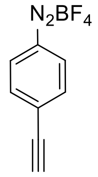

4-ethynylbenzenediazonium tetrafluoroborate (58): Synthesized according to general procedure using 4-ethynylaniline (1.2 g, 10 mmol). The title compound was isolated as a white solid (1.2 g, 54% yield). Spectral data matches previous literature.[3]

^{1}H NMR (400 MHz, MeOD) δ 8.64 (d, *J* = 9.0 Hz, 2H), 8.05 (d, *J* = 8.9 Hz, 2H), 4.56 (s, 1H).

^{13}C NMR (101 MHz, DMSO) δ 134.1, 133.7, 133.0, 115.5, 91.4, 81.4.

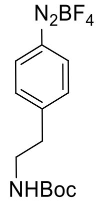

4-(2-((*tert*-butoxycarbonyl)amino)ethyl)benzenediazonium tetrafluoroborate (59): Synthesized according to general procedure using 4-(2-((tert-butoxycarbonyl)amino)ethyl)aniline (2.4 g, 10 mmol). The title compound was isolated as a white solid (2.4 g, 73% yield). Spectral data matches previous literature.[5]

^{1}H NMR (400 MHz, MeOD) δ 8.56 (d, *J* = 8.3 Hz, 2H), 7.85 (t, *J* = 10.2 Hz, 2H), 3.42 (t, *J* = 6.6 Hz, 2H), 3.09 (t, *J* = 6.6 Hz, 2H), 1.43 (s, 9H).

^{13}C NMR (101 MHz, D_2O) δ 145.6, 144.9, 132.6, 132.5, 132.1, 78.7, 36.3, 29.9, 27.5.

3.2. Protected Indoles synthesis

3.2.1. General procedure: *N*-Boc protection of Indoles

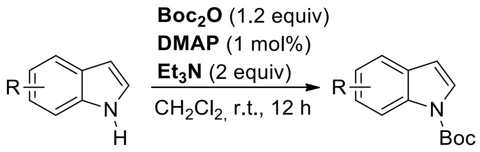

Scheme S4: *N*-Boc protection of Indoles.

To an ice-cooled solution of indole (1 equiv.), DMAP (1 mol%), and triethylamine (1.3 equiv.) in dichloromethane (0.5 M) was added di-*tert*-butyl-dicarbonate (1.2 equiv.) under vigorous stirring. The reaction was then stirred at room temperature for 12 h before quenching with saturated NH_4Cl aqueous solution (20 mL). The aqueous phase was extracted with ethyl acetate (20 mL, 3 x) and the combined organic phases washed with brine (20 mL). The organic phase was dried over MgSO4 and the solvent removed under vacuum. The crude product was purified through column chromatography using the specified mobile phase in each case to yield the pure product.

3.2.2. Experimental details for Protected Indoles

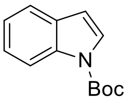

***tert*-butyl 1H-indole-1-carboxylate (1):** Synthesized according to general procedure using 1H-indole (3.0 g, 26 mmol, 1 equiv.), Boc_2O (6.7 g, 31 mmol, 1.2 equiv), DMAP (31 mg, 0.26 mmol, 1 mol%) and triethylamine (4.6 mL, 34 mmol, 1.3 equiv) in dichloromethane (51 mL, 0.5 M). The crude product was purified through column chromatography using 5% EtOAc/Hexanes. The title compound was isolated as a colorless oil (5.0 g, 90% yield). Spectral data matches previous literature.[7]

^{1}H NMR (500 MHz, $CDCl_3$) δ 8.15 (d, *J* = 7.5 Hz, 1H), 7.60 (d, *J* = 3.5 Hz, 1H), 7.57 (d, *J* = 7.8 Hz, 1H), 7.34 – 7.29 (m, 1H), 7.25 – 7.20 (m, 1H), 6.57 (d, *J* = 3.7 Hz, 1H), 1.68 (s, 9H).

^{13}C NMR (126 MHz, $CDCl_3$) δ 149.9, 135.3, 130.6, 125.9, 124.3, 122.7, 121.0, 115.2, 107.4, 83.7, 28.3.

IR (ATR): $\tilde{\nu}$(cm^{-1}) = 2980 (w) (C-H), 1730 (s) (C=O)

HRMS (ESI) m/z: [M+H]$^+$ Calcd for $C_{13}H_{16}NO_2$ 218.1176; Found 218.1158.

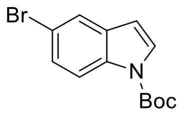

***tert*-butyl 5-Bromo-1H-indole-1-carboxylate (60):** Synthesized according to general procedure using 5-Bromo-1H-indole (1.0 g, 5.1 mmol, 1 equiv.), Boc_2O (1.3 g, 6.1 mmol, 1.2 equiv.), DMAP (6.1 mg, 51 μmol, 1 mol%) and triethylamine (0.9 mL, 7 mmol, 1.3 equiv) in dichloromethane (10 mL, 0.51 M). The crude product was purified through column chromatography using 2% EtOAc/Hexanes. The title compound was isolated as a white solid (1.3 g, 88% yield). Spectral data matches previous literature.[8]

^{1}H NMR (500 MHz, $CDCl_3$) δ 8.02 (d, *J* = 8.2 Hz, 1H), 7.68 (s, 1H), 7.58 (d, *J* = 3.4 Hz, 1H), 7.39 (d, *J* = 8.8 Hz, 1H), 6.50 (d, *J* = 3.7 Hz, 1H), 1.67 (s, 9H).

^{13}C NMR (126 MHz, $CDCl_3$) δ 149.5, 134.0, 132.4, 127.1 (2C), 123.6, 116.7, 116.1, 106.6, 84.2, 28.3.

IR (ATR): $\tilde{\nu}$(cm^{-1}) = 2983 (w), 2931 (w) (C-H), 1729 (m) (C=O), 680 (m) (C-Br).

HRMS (ESI) m/z: [M+H]$^+$ Calcd for $C_{13}H_{15}BrNO_2$ 296.0281; Found 296.0280.

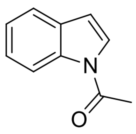

***N*-Acetyl-Indole (61):** To a round-bottomed flask containing 1H-indole (0.58 g, 4.9 mmol, 1 equiv.), dimethylamino pyridine (0.23 g, 1.9 mmol, 0.4 equiv.) and triethylamine (2.1 mL, 15 mmol, 3 equiv.) dissolved in dichloromethane (10 mL, 0.50 M), acetic anhydride (1.9 mL, 20 mmol, 4 equiv.) were added dropwise under magnetic stirring. The reaction was stirred overnight at room temperature before it was quenched with saturated NH_4Cl aqueous solution (20 mL). The aqueous phase was extracted with ehtyl acetate (20 mL, 3x) and the combined organic phases washed with brine (20 mL). The organic phase was dried with $MgSO_4$ and the solvent removed *in vacuo*. The crude mixture was purified through column chromatography using 10% Ethyl Acetate in Hexanes to yield the pure product as a colorless oil (0.774 g, 99% yield). Spectral data matches previous literature.[10]

^{1}H NMR (500 MHz, $CDCl_3$) δ 8.46 (d, *J* = 7.9 Hz, 1H), 7.58 (d, *J* = 7.8 Hz, 1H), 7.39-7.35 (m, 2H), 7.29 (t, *J* = 7.5 Hz, 1H), 6.63 (d, *J* = 3.8 Hz, 1H), 2.60 (s, 3H).

^{13}C NMR (126 MHz, $CDCl_3$) δ 168.7, 135.6, 130.5, 125.3, 125.1, 123.7, 120.9, 116.6, 109.2, 24.0.

IR (ATR): $\tilde{\nu}$(cm^{-1}) = 1701 (m) (C=O).

HRMS (ESI) m/z: [M+H]$^+$ Calcd for $C_{10}H_{10}NO$ 160.0757; Found 160.0756.

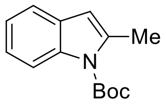

***tert*-butyl 2-methyl-1H-indole-1-carboxylate (62):** Synthesized according to general procedure using 2-methyl-1H-indole (1.0 g, 7.6 mmol, 1 equiv.), Boc_2O (2.0 g, 9.1 mmol, 1.2 equiv.), DMAP (9.3 mg, 76 μmol, 1 mol%) and triethylamine (1.4 mL, 9.9 mmol, 1.3 equiv) in dichloromethane (15 mL, 0,51 M). The crude product was purified through column chromatography using 5% EtOAc/Hexanes. The title compound was isolated as a white solid (1.5 g, 85% yield). Spectral data matches previous literature.[9]

^{1}H NMR (500 MHz, $CDCl_3$) δ 8.10 (d, *J* = 8.1 Hz, 1H), 7.43 (d, *J* = 7.5 Hz, 1H), 7.25 – 7.16 (m, 2H), 6.32 (s, 1H), 2.60 (s, 3H), 1.69 (s, 9H).

^{13}C NMR (126 MHz, $CDCl_3$) δ 150.8, 137.9, 136.6, 129.5, 123.2, 122.7, 119.6, 115.6, 108.1, 83.7, 28.4, 17.2.

IR (ATR): $\tilde{\nu}$(cm^{-1}) = 2984 (w), 2929 (w) (C-H), 1721 (m) (C=O).

HRMS (ESI) m/z: [M+Na]$^+$ Calcd for $C_{14}H_{17}NNaO_2$ 254.1151; Found 254.1152.

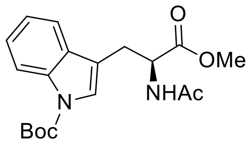

***tert*-butyl (*S*)-3-(2-acetamido-3-methoxy-3-oxopropyl)-1*H*-indole-1-carboxylate (63):** Synthesized according to general procedure using methyl acethyl-L-tryptophanate (0.26 g, 1.0 mmol, 1 equiv.), Boc_2O (0.44 g, 2.0 mmol, 2 equiv.), DMAP (12 mg, 0.10

mmol, 10 mol%) and triethylamine (0.20 mL, 1.3 mmol, 1.3 equiv.) in dichloromethane (2 mL, 0.5 M). The crude product was purified through column chromatography using 50% EtOAc/Hexanes. The title compound was isolated as a white solid (239 mg, 66% yield). Spectral data matches previous literature.[12]

^{1}H NMR (500 MHz, $CDCl_3$) δ 8.09 (s, 1H), 7.47 (d, *J* = 7.8 Hz, 1H), 7.36 (s, 1H), 7.33 – 7.28 (m, 1H), 7.25 – 7.20 (m, 1H), 6.02 (d, *J* = 7.4 Hz, 1H), 4.94 (dt, *J* = 7.6, 5.3 Hz, 1H), 3.71 (s, 3H), 3.26 (ddd, *J* = 35.7, 14.7, 5.1 Hz, 2H), 1.98 (s, 3H), 1.67 (s, 9H).

^{13}C NMR (101 MHz, $CDCl_3$) δ 172.2, 169.8, 149.6, 135.3, 130.7, 124.6, 124.1, 122.7, 118.8, 115.4, 115.0, 83.8, 77.5, 77.2, 76.8, 52.7, 52.5, 28.3, 27.4, 23.2.

IR (ATR): ṽ(cm^{-1}) = 2981 (w) (C-H), 1733 (m), 1658 (m) (C=O).

HRMS (ESI) m/z: [M+Na]$^+$ Calcd for $C_{19}H_{24}N_2O_5Na$ 383.1583; Found 383.1571.

$[\alpha]_D^{25}$ = +65.5° (c=1.0 in $CHCl_3$)

3.3. Arylated Indoles Synthesis

3.3.1. General procedure for Photochemical Indole Arylation reaction

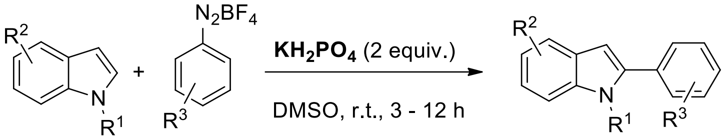

Scheme S5: Photochemical Indole arylation.

To a 5 mL glass vial charged with a magnetic stirring bar, *N*-protected indole (5 equiv.), and potassium phosphate monobasic (2 equiv.) was added 1 mL of DMSO. The mixture was stirred for a couple of minutes before the diazonium salt (1 equiv.) was added at once. The vial was closed with a rubber septum, and the mixture was purged with argon for 15 minutes in the dark. The vial was then sealed with parafilm and kept under argon atmosphere. The reaction was then irradiated with a Blue LED (450-460 nm) for the required time. After the reaction time, the mixture was then diluted with ethyl acetate (10 mL) and washed twice with saturated sodium hydrogen carbonate solution (10 mL) and once with brine (10 mL). The organic phase was dried over MgSO4 and the solvent removed *in vacuo*. The crude reaction mixture was then submitted to flash column chromatography with the specified system for each case.

3.3.2. Experimental details for arylated indoles

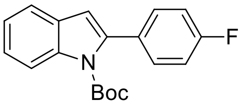

***tert*-butyl 2-(4-fluorophenyl)-1H-indole-1-carboxylate (3):** Synthesized according to general procedure using *tert*-butyl 1H-indole-1-carboxylate (0.33 g, 1.5 mmol, 5 equiv.), potassium phosphate monobasic (82 mg, 0.60 mmol, 2 equiv.), 4-fluorobenzenediazonium tetrafluoroborate (63 mg, 0.30 mmol, 1 equiv.) and DMSO (1 mL, 0.3 M). The reaction was irradiated for 3 hours using 10W Blue LED. The crude product was purified through column chromatography using Hexanes. Further purification was required, which was accomplished through reverse phase chromatography using a gradient of 75-100% methanol in water. The title compound was isolated as a white solid (51 mg, 55% yield). Spectral data matches previous literature.[13]

^{1}H NMR (500 MHz, $CDCl_3$) δ 8.21 (d, *J* = 8.4 Hz, 1H), 7.55 (d, *J* = 7.7 Hz, 1H), 7.42 – 7.37 (m, 2H), 7.36 – 7.32 (m, 1H), 7.29 – 7.22 (m, 1H), 7.15 – 7.06 (m, 2H), 6.54 (s, 1H), 1.36 (s, 9H).

^{13}C NMR (126 MHz, $CDCl_3$) δ 162.6 (d, *J* = 247.1 Hz), 150.2, 139.5, 137.5, 131.2 (d, *J* = 3.5 Hz), 130.5 (d, *J* = 8.1 Hz), 129.2, 124.6, 123.2, 120.6, 115.4, 114.9 (d, *J* = 21.6 Hz), 110.3, 83.7, 27.8.

^{19}F NMR (470 MHz, $CDCl_3$) δ -114.35 (tt, *J* = 8.7, 5.3 Hz)

IR (ATR): ṽ(cm^{-1}) = 2986 (w) (C-H), 1727 (s) (C=O).

HRMS (ESI) m/z: [M+Na]$^+$ Calcd for $C_{19}H_{18}FNO_2Na$ 334.1214; Found 334.1220.

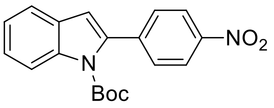

***tert*-butyl 2-(4-nitrophenyl)-1H-indole-1-carboxylate (4):** Synthesized according to general procedure using *tert*-butyl 1H-indole-1-carboxylate (0.33 g, 1.5 mmol, 5 equiv.), potassium phosphate monobasic (82 mg, 0.60 mmol, 2 equiv.), 4-nitrobenzenediazonium tetrafluoroborate (71 mg, 0.30 mmol, 1 equiv.) and DMSO (1 mL, 0.3 M). The reaction was irradiated for 3 hours using 1 W Blue LED. The crude product was purified through column chromatography using 10% ethyl acetate in hexanes. The title compound was isolated as a yellow solid (90 mg, 89% yield). Spectral data matches previous literature.[14]

^{1}H NMR (500 MHz, CDCl$_3$) δ 8.30 – 8.25 (m, 2H), 8.21 (dd, *J* = 8.4, 0.6 Hz, 1H), 7.63 – 7.57 (m, 3H), 7.40 (ddd, *J* = 8.5, 7.3, 1.2 Hz, 1H), 7.33 – 7.27 (m, 1H), 6.69 (s, 1H), 1.41 (s, 9H).

^{13}C NMR (126 MHz, CDCl$_3$) δ 149.9, 147.0, 141.4, 138.1, 137.9, 129.4, 129.0, 125.5, 123.5, 123.2, 121.1, 115.6, 112.2, 84.5, 27.8.

IR (ATR): $\tilde{\nu}$(cm^{-1}) = 2981 (w), 2933 (C-H), 1727 (s) (C=O), 1508 (s) (N-O).

HRMS (ESI) m/z: [M+Na]$^+$ Calcd for $C_{19}H_{18}N_2NaO_4$ 361.1159; Found 361.1158.

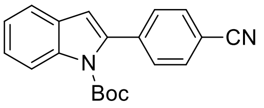

***tert*-butyl 2-(4-cyanophenyl)-1H-indole-1-carboxylate (5):** Synthesized according to general procedure using *tert*-butyl 1H-indole-1-carboxylate (0.33 g, 1.5 mmol, 5 equiv.), potassium phosphate monobasic (82 mg, 0.60 mmol, 2 equiv.), 4-cyanobenzenediazonium tetrafluoroborate (65 mg, 0.30 mmol, 1 equiv.) and DMSO (1 mL, 0.3 M). The reaction was irradiated for 3 hours using 10 W Blue LED. The crude product was purified through column chromatography using 10% ethyl acetate in hexanes. The title compound was isolated as a white solid (75 mg, 79% yield). Spectral data matches previous literature.[14]

^{1}H NMR (500 MHz, CDCl$_3$) δ 8.21 (d, *J* = 8.4 Hz, 1H), 7.73 – 7.68 (m, 2H), 7.58 (d, *J* = 7.7 Hz, 1H), 7.56 – 7.51 (m, 2H), 7.38 (ddd, *J* = 8.4, 7.3, 1.2 Hz, 1H), 7.32 – 7.27 (m, 1H), 6.64 (s, 1H), 1.38 (s, 9H).

^{13}C NMR (126 MHz, CDCl$_3$) δ 149.9, 139.6, 138.4, 137.8, 131.7, 129.4, 129.0, 125.3, 123.4, 121.0, 118.9, 115.6, 111.8, 111.2, 84.4, 27.8.

IR (ATR): $\tilde{\nu}$(cm^{-1}) = 2984 (w), 2932 (C-H), 2228 (m) (CN), 1726 (s) (C=O).

HRMS (ESI) m/z: [M+Na]$^+$ Calcd for $C_{20}H_{18}N_2NaO_2$ 341.1260; Found 341.1257.

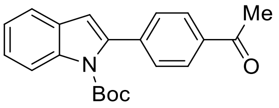

***tert*-butyl 2-(4-acetylphenyl)-1H-indole-1-carboxylate (6):** Synthesized according to general procedure using *tert*-butyl 1H-indole-1-carboxylate (0.33 g, 1.5 mmol, 5 equiv.), potassium phosphate monobasic (82 mg, 0.60 mmol, 2 equiv.), 4-acetylbenzenediazonium tetrafluoroborate (70 mg, 0.30 mmol, 1 equiv.) and DMSO (1 mL, 0.3 M). The reaction was irradiated for 3 hours using 10 W Blue LED. The crude product was purified through column chromatography using 10% ethyl acetate in hexanes. The title compound was isolated as a white solid (75 mg, 70% yield). Spectral data matches previous literature.[15]

^{1}H NMR (500 MHz, CDCl$_3$) δ 8.21 (d, *J* = 8.4 Hz, 1H), 8.01 (d, *J* = 8.4 Hz, 2H), 7.58 (d, *J* = 7.7 Hz, 1H), 7.54 (d, *J* = 8.4 Hz, 2H), 7.40 – 7.33 (m, 1H), 7.28 (dd, *J* = 11.1, 4.0 Hz, 1H), 6.64 (s, 1H), 2.65 (s, 3H), 1.36 (s, 9H).

^{13}C NMR (126 MHz, CDCl$_3$) δ 197.7, 150.1, 139.7, 139.4, 137.8, 136.0, 129.2, 128.8, 128.0, 125.0, 123.3, 120.9, 115.4, 111.2, 84.1, 77.4, 77.2, 76.9, 27.8, 26.8.

IR (ATR): $\tilde{\nu}$(cm^{-1}) = 2990 (w) (C-H), 1728 (s), 1677 (s) (C=O).

HRMS (ESI) m/z: [M+Na]$^+$ Calcd for $C_{21}H_{21}NNaO_3$ 358.1414; Found 358.1411.

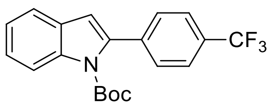

***tert*-butyl 2-(4-(trifluoromethyl)phenyl)-1H-indole-1-carboxylate (7):** Synthesized according to general procedure using *tert*-butyl 1H-indole-1-carboxylate (0.33 g, 1.5 mmol, 5 equiv.), potassium phosphate monobasic (82 mg, 0.60 mmol, 2 equiv.), 4-(trifluoromethyl)benzenediazonium tetrafluoroborate (78 mg, 0.30 mmol, 1 equiv.) and DMSO (1 mL, 0.3 M). The reaction was irradiated for 3 hours using 10 W Blue LED. The crude product was purified through column chromatography using 5% ethyl acetate in hexanes. Fractions containing the product were then submitted to a reversed-phase column chromatography using a gradient of 75-100% of methanol in water. The title compound was isolated as a light-yellow oil (60 mg, 55% yield). Spectral data matches previous literature.[14]

^{1}H NMR (500 MHz, $CDCl_3$) δ 8.22 (d, *J* = 8.4 Hz, 1H), 7.67 (d, *J* = 8.1 Hz, 2H), 7.58 (d, *J* = 7.8 Hz, 1H), 7.55 (d, *J* = 8.1 Hz, 2H), 7.37 (t, *J* = 7.8 Hz, 1H), 7.28 (t, *J* = 7.6 Hz, 1H) (Signal superimposed with solvent trace), 6.62 (s, 1H), 1.34 (s, 9H).

^{13}C NMR (126 MHz, $CDCl_3$) δ 149.9, 138.8, 138.6, 137.6, 129.6 (q, *J* = 32.4 Hz), 129.0, 129.0,124.2 (q, J=272.2 Hz), 124.9, 124.7 (q, *J* = 3.7 Hz), 123.2, 120.7, 115.4, 111.0, 83.9, 27.6.

^{19}F NMR (470 MHz, $CDCl_3$) δ -62.49

IR (ATR): $\tilde{\nu}$(cm^{-1}) = 2925 (w) (C-H), 1739 (s) (C=O).

HRMS (ESI) m/z: $[2M+Na]^+$ Calcd for $C_{40}H_{36}F_6N_2NaO_4$ 745.2471; Found 745.2463.

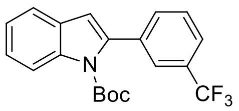

***tert*-butyl 2-(3-(trifluoromethyl)phenyl)-1H-indole-1-carboxylate (8):** Synthesized according to general procedure using *tert*-butyl 1H-indole-1-carboxylate (0.33 g, 1.5 mmol, 5 equiv.), potassium phosphate monobasic (82 mg, 0.60 mmol, 2 equiv.), 3-(trifluoromethyl)benzenediazonium tetrafluoroborate (78 mg, 0.30 mmol, 1 equiv.) and DMSO (1 mL, 0.3 M). The reaction was irradiated for 3 hours using 10 W Blue LED. The crude product was purified through column chromatography using 5% ethyl acetate in hexanes. Fractions containing the product were then submitted to a reversed-phase column chromatography using a gradient of 75-100% of methanol in water. The title compound was isolated as a light-yellow solid (65 mg, 60% yield).

^{1}H NMR (500 MHz, $CDCl_3$) δ 8.25 (d, *J* = 8.4 Hz, 1H), 7.69 (s, 1H), 7.63 (d, *J* = 7.9 Hz, 2H), 7.58 (d, *J* = 7.7 Hz, 1H), 7.53 (t, *J* = 7.7 Hz, 1H), 7.37 (t, *J* = 7.8 Hz, 1H), 7.31 – 7.25 (m, 1H), 6.62 (s, 1H), 1.31 (s, 9H).

^{13}C NMR (126 MHz, $CDCl_3$) δ 150.1, 138.8, 137.7, 136.0, 132.0, 130.5 (q, *J* = 32.3 Hz), 129.1, 128.5, 125.8 (q, *J* = 3.8 Hz), 124.2 (q, J = 273.4 Hz), 125.0, 124.3 (q, *J* = 3.7 Hz), 123.3, 120.8, 115.6, 111.1, 84.0, 27.7.

^{19}F NMR (470 MHz, $CDCl_3$) δ -62.63

IR (ATR): $\tilde{\nu}$(cm^{-1}) = 2982 (w) (C-H), 1733 (s) (C=O).

HRMS (ESI) m/z: $[M+Na]^+$ Calcd for $C_{20}H_{18}F_3NNaO_2$ 384.1182; Found 384.1170.

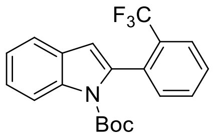

***tert*-butyl 2-(2-(trifluoromethyl)phenyl)-1H-indole-1-carboxylate (9):** Synthesized according to general procedure using *tert*-butyl 1H-indole-1-carboxylate (0.33 g, 1.5 mmol, 5 equiv.), potassium phosphate monobasic (82 mg, 0.60 mmol, 2 equiv.), 2-(trifluoromethyl)benzenediazonium tetrafluoroborate (78 mg, 0.30 mmol, 1 equiv.) and DMSO (1 mL, 0.3 M). The reaction was irradiated for 3 hours using 10 W Blue LED. The crude product was purified through column chromatography using 1% ethyl acetate in hexanes. The title compound was isolated as a light-yellow solid (55 mg, 51% yield).

M. P. 107 ºC

^{1}H NMR (500 MHz, $CDCl_3$) δ 8.33 (d, *J* = 8.4 Hz, 1H), 7.76 (d, *J* = 7.8 Hz, 1H), 7.58 (t, *J* = 7.2 Hz, 2H), 7.52 (t, *J* = 7.6 Hz, 1H), 7.43 (d, *J* = 7.5 Hz, 1H), 7.38 (dd, *J* = 8.2, 7.4 Hz, 1H), 7.29 (t, *J* = 7.5 Hz, 1H), 6.56 (s, 1H), 1.23 (s, 9H).

^{13}C NMR (126 MHz, $CDCl_3$) δ 149.7, 136.7, 135.3, 134.1 (q, *J* = 4.2 Hz), 132.1, 130.9, 129.4 (q, *J* = 30.1 Hz), 128.9, 128.1, 125.8 (q, *J* = 5.0 Hz), 123.9 (q, J = 274.7 Hz), 124.6, 123.0, 120.6, 115.6, 111.1 (d, *J* = 1.3 Hz), 83.2, 27.4.

^{19}F NMR (470 MHz, $CDCl_3$) δ -59.33

IR (ATR): ṽ(cm^{-1}) = 2990 (w) (C-H), 1727 (s) (C=O).

HRMS (ESI) m/z: [M+Na]⁺ Calcd for $C_{20}H_{18}F_3NNaO_2$ 384.1182; Found 384.1185.

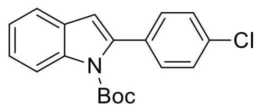

***tert*-butyl 2-(4-chlorophenyl)-1H-indole-1-carboxylate (10):** Synthesized according to general procedure using *tert*-butyl 1H-indole-1-carboxylate (0.33 g, 1.5 mmol, 5 equiv.), potassium phosphate monobasic (82 mg, 0.60 mmol, 2 equiv.), 4-chlorobenzenediazonium tetrafluoroborate (68 mg, 0.30 mmol, 1 equiv.) and DMSO (1 mL, 0.3 M). The reaction was irradiated for 3 hours using 10 W Blue LED. The crude product was purified through column chromatography using 1% ethyl acetate in hexanes. Fractions containing the product were then submitted to a reversed-phase column chromatography using a gradient of 80-100% of methanol in water. The title compound was isolated as a white solid (69 mg, 70% yield). Spectral data matches previous literature.[16]

^{1}H NMR (500 MHz, CDCl$_3$) δ 8.21 (d, *J* = 8.3 Hz, 1H), 7.56 (d, *J* = 7.7 Hz, 1H), 7.41 – 7.32 (m, 5H), 7.29 – 7.24 (m, 1H), 6.56 (s, 1H), 1.37 (s, 9H).

^{13}C NMR (126 MHz, CDCl$_3$) δ 150.2, 139.3, 137.6, 133.7, 133.6, 130.1, 129.2, 128.1, 124.7, 123.2, 120.7, 115.5, 110.5, 83.9, 27.8.

IR (ATR): ṽ(cm^{-1}) = 2980 (w) (C-H), 1725 (s) (C=O).

HRMS (ESI) m/z: [M+Na]⁺ Calcd for $C_{19}H_{18}ClNNaO_2$ 350.0918; Found 350.0908.

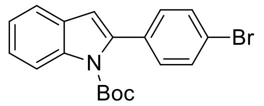

***tert*-butyl 2-(4-bromophenyl)-1H-indole-1-carboxylate (11):** Synthesized according to general procedure using *tert*-butyl 1H-indole-1-carboxylate (0.33 g, 1.5 mmol, 5 equiv.), potassium phosphate monobasic (82 mg, 0.60 mmol, 2 equiv.), 4-bromobenzenediazonium tetrafluoroborate (81 mg, 0.30 mmol, 1 equiv.) and DMSO (1 mL, 0.3 M). The reaction was irradiated for 3 hours using 10 W Blue LED. The crude product was purified through column chromatography using 1% ethyl acetate in hexanes. Fractions containing the product were then submitted to a reversed-phase column chromatography using a gradient of 80-100% of methanol in water. The title compound was isolated as a white solid (74 mg, 66% yield). Spectral data matches previous literature.[17]

^{1}H NMR (500 MHz, CDCl$_3$) δ 8.22 (d, *J* = 8.4 Hz, 1H), 7.56 (t, *J* = 8.2 Hz, 3H), 7.35 (d, *J* = 7.5 Hz, 1H), 7.31 (d, *J* = 8.4 Hz, 2H), 7.29 – 7.25 (m, 1H), 6.57 (s, 1H), 1.38 (s, 9H).

^{13}C NMR (126 MHz, CDCl$_3$) δ 150.1, 139.3, 137.6, 134.0, 131.1, 130.4, 129.2, 124.7, 123.2, 121.8, 120.7, 115.4, 110.5, 83.9, 27.8.

IR (ATR): ṽ(cm^{-1}) = 2979 (w), 2929 (w) (C-H), 1731 (s) (C=O).

HRMS (ESI) m/z: [M+Na]⁺ Calcd for $C_{19}H_{18}BrNNaO_2$ 394.0413; Found 394.0420.

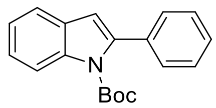

***tert*-butyl 2-phenyl-1H-indole-1-carboxylate (12):** Synthesized according to general procedure using *tert*-butyl 1H-indole-1-carboxylate (0.33 g, 1.5 mmol, 5 equiv.), potassium phosphate monobasic (82 mg, 0.60 mmol, 2 equiv.), benzenediazonium tetrafluoroborate (58 mg, 0.30 mmol, 1 equiv.) and DMSO (1 mL, 0.3 M). The reaction was irradiated for 3 hours using 10 W Blue LED. The crude product was purified through column chromatography using hexanes. Fractions containing the product were then submitted to a reversed-phase column chromatography using a gradient of 75-100% of methanol in water. The title compound was isolated as a colorless oil (12 mg, 14% yield). Spectral data matches previous literature.[15]

^{1}H NMR (500 MHz, CDCl$_3$) δ 8.24 (dd, *J* = 8.3, 0.6 Hz, 1H), 7.57 (d, *J* = 7.6 Hz, 1H), 7.45 – 7.39 (m, 4H), 7.39 – 7.33 (m, 2H), 7.29 – 7.25 (m, 2H), 6.57 (s, 1H), 1.32 (s, 9H).

^{13}C NMR (126 MHz, CDCl$_3$) δ 150.3, 140.6, 137.6, 135.1, 129.3, 128.9, 127.9, 127.7, 124.4, 123.0, 120.6, 115.3, 110.0, 83.5, 76.9, 27.7.

IR (ATR): ṽ(cm^{-1}) = 2979 (w), 2932 (w) (C-H), 1728 (s) (C=O).

HRMS (ESI) m/z: [M+H]⁺ Calcd for $C_{19}H_{20}NO_2$ 294.1494; Found 294.1488.

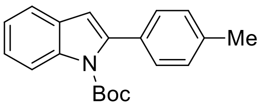

***tert*-butyl 2-(*p*-tolyl)-1H-indole-1-carboxylate (13):** Synthesized according to general procedure using *tert*-butyl 1H-indole-1-carboxylate (0.33 g, 1.5 mmol, 5 equiv.), potassium phosphate monobasic (82 mg, 0.60 mmol, 2 equiv.), 4-methylbenzenediazonium tetrafluoroborate (62 mg, 0.30 mmol, 1 equiv.) and DMSO (1 mL, 0.3 M). The reaction was irradiated for 3 hours using 10 W Blue LED. The crude product was purified through column chromatography using 1% ethyl acetate in hexanes. Fractions containing the product were then submitted to a reversed-phase column chromatography using a gradient of 75-100% of methanol in water. The title compound was isolated as a white solid (34 mg, 37% yield). Spectral data matches previous literature.[15]

^{1}H NMR (500 MHz, $CDCl_3$) δ 8.21 (d, *J* = 8.3 Hz, 1H), 7.56 (d, *J* = 7.7 Hz, 1H), 7.36 – 7.31 (m, 3H), 7.28 – 7.21 (m, 3H), 6.55 (s, 1H), 2.42 (s, 3H), 1.36 (s, 9H).

^{13}C NMR (126 MHz, $CDCl_3$) δ 150.3, 140.7, 137.4, 137.4, 132.0, 129.3, 128.6, 128.5, 124.1, 122.8, 120.4, 115.2, 109.6, 83.4, 77.3, 77.0, 76.8, 27.6, 21.3.

IR (ATR): $\tilde{\nu}$(cm^{-1}) = 2975 (w), 2864 (w) (C-H), 1744 (s) (C=O).

HRMS (ESI) m/z: [M+Na]$^+$ Calcd for $C_{20}H_{21}NNaO_2$ 330.1464; Found 330.1467.

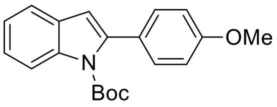

***tert*-butyl 2-(4-methoxyphenyl)-1H-indole-1-carboxylate (14):** Synthesized according to general procedure using *tert*-butyl 1H-indole-1-carboxylate (0.33 g, 1.5 mmol, 5 equiv.), potassium phosphate monobasic (82 mg, 0.60 mmol, 2 equiv.), 4-methoxybenzenediazonium tetrafluoroborate (66 mg, 0.30 mmol, 1 equiv.) and DMSO (1 mL, 0.3 M). The reaction was irradiated for 12 hours using 10 W Blue LED. The crude product was purified through column chromatography using 1% ethyl acetate in hexanes. Fractions containing the product were then submitted to a reversed-phase column chromatography using a gradient of 75-100% of methanol in water. The title compound was isolated as a white solid (40 mg, 41% yield). Spectral data matches previous literature.[14]

^{1}H NMR (500 MHz, $CDCl_3$) δ 8.20 (dd, *J* = 8.3, 0.6 Hz, 1H), 7.54 (d, *J* = 7.3 Hz, 1H), 7.37 – 7.34 (m, 2H), 7.32 (ddd, *J* = 8.4, 7.3, 1.3 Hz, 1H), 7.27 – 7.23 (m, 1H), 6.97 – 6.93 (m, 2H), 6.52 (s, 1H), 3.86 (d, *J* = 2.2 Hz, 3H), 1.37 (s, 9H).

^{13}C NMR (126 MHz, $CDCl_3$) δ 159.4, 150.4, 140.5, 137.4, 130.0, 129.4, 127.6, 124.2, 123.0, 120.4, 115.3, 113.4, 109.6, 83.5, 55.5, 27.8.

IR (ATR): $\tilde{\nu}$(cm^{-1}) = 2926 (w), 2852 (w) (C-H), 1740 (s) (C=O).

HRMS (ESI) m/z: [M+H]$^+$ Calcd for $C_{20}H_{22}NO_3$ 324.1600; Found 324.1619.

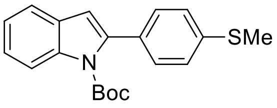

***tert*-butyl 2-(4-(methylthio)phenyl)-1H-indole-1-carboxylate (15):** Synthesized according to general procedure using *tert*-butyl 1H-indole-1-carboxylate (0.33 g, 1.5 mmol, 5 equiv.), potassium phosphate monobasic (82 mg, 0.60 mmol, 2 equiv.), 4-(methylthio)benzenediazonium tetrafluoroborate (71 mg, 0.30 mmol, 1 equiv.) and DMSO (1 mL, 0.3 M). The reaction was irradiated for 3 hours using 10 W Blue LED. The crude product was purified through column chromatography using 1-10% ethyl acetate in hexanes. Fractions containing the product were then submitted to a second column chromatography purification using a gradient of -50% of dichloromethane in hexanes. The title compound was isolated as a yellowish solid (22 mg, 22% yield).

^{1}H NMR (500 MHz, $CDCl_3$) δ 8.19 (dd, *J* = 8.3, 0.6 Hz, 1H), 7.55 (d, *J* = 7.4 Hz, 1H), 7.34 (m, 3H), 7.32 – 7.28 (m, 12H), 7.27 – 7.23 (m, 2H), 6.54 (s, 1H), 2.53 (s, 3H), 1.36 (s, 9H).

^{13}C NMR (126 MHz, $CDCl_3$) δ 150.3, 140.2, 138.2, 137.6, 131.8, 129.4, 129.2, 126.1, 124.4, 123.1, 120.6, 115.4, 110.1, 83.7, 27.8, 16.1.

IR (ATR): $\tilde{\nu}$(cm^{-1}) = 2976 (w), 2924 (w) (C-H), 1731 (s) (C=O).

HRMS (ESI) m/z: [M+Na]$^+$ Calcd for $C_{20}H_{21}NNaO_2S$ 362.1185; Found 362.1188.

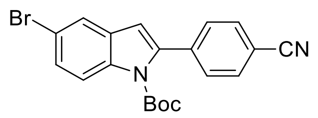

***tert*-butyl 5-bromo-2-(4-cyanophenyl)-1H-indole-1-carboxylate (16):** Synthesized according to general procedure using *tert*-butyl 5-Bromo-1H-indole-1-carboxylate (0.44 g, 1.5 mmol, 5 equiv.), potassium phosphate monobasic (82 mg, 0.60 mmol, 2 equiv.), 4-cyanobenzenediazonium tetrafluoroborate (65 mg, 0.30 mmol, 1 equiv.) and DMSO (1 mL, 0.3 M). The reaction was irradiated for 3 hours using 10 W Blue LED. The crude product was purified through column chromatography using 1-5% ethyl acetate in hexanes. The title compound was isolated as a white solid (56 mg, 47% yield). Spectral data matches previous literature.[18]

^{1}H NMR (500 MHz, CDCl$_3$) δ 8.07 (d, *J* = 8.8 Hz, 1H), 7.70 (t, *J* = 5.6 Hz, 3H), 7.52 (d, *J* = 8.1 Hz, 2H), 7.45 (dd, *J* = 8.9, 1.9 Hz, 1H), 6.55 (s, 1H), 1.35 (s, 9H).

^{13}C NMR (126 MHz, CDCl$_3$) δ 149.6, 139.5, 139.1, 136.5, 131.8, 130.7, 129.4, 128.1, 123.5, 118.8, 117.1, 116.6, 111.6, 110.6, 84.9, 27.8.

IR (ATR): $\tilde{\nu}$(cm^{-1}) = 2976 (w) (C-H), 2227 (m) (CN), 1735 (s) (C=O).

HRMS (ESI) m/z: [M+H]$^+$ Calcd for $C_{20}H_{18}BrN_2O_2$ 397.0552; Found 397.0499.

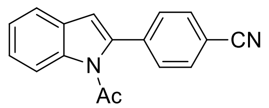

4-(1-acetyl-1H-indol-2-yl)benzonitrile (17): Synthesized according to general procedure using *N*-Acetyl-Indole (0.24 g, 1.5 mmol, 5 equiv.), potassium phosphate monobasic (82 mg, 0.60 mmol, 2 equiv.), 4-cyanobenzenediazonium tetrafluoroborate (65 mg, 0.30 mmol, 1 equiv.) and DMSO (1 mL, 0.3 M). The reaction was irradiated for 3 hours using 10 W Blue LED. The crude product was purified through column chromatography using 5-10% ethyl acetate in hexanes. The title compound was isolated as a white solid (32 mg, 41% yield). Spectral data matches previous literature.[19]

^{1}H NMR (500 MHz, CDCl$_3$) δ 8.23 (d, *J* = 8.4 Hz, 1H), 7.74 (d, *J* = 8.3 Hz, 2H), 7.62 – 7.55 (m, 3H), 7.40 (ddd, *J* = 8.5, 7.4, 1.3 Hz, 1H), 7.34 – 7.29 (m, 1H), 6.73 (s, 1H), 2.24 (s, 3H).

^{13}C NMR (126 MHz, CDCl$_3$) δ 170.5, 138.8, 138.0, 137.9, 132.5, 129.2, 128.9, 126.0, 124.0, 121.2, 118.5, 115.7, 113.6, 112.1, 28.1.

IR (ATR): $\tilde{\nu}$(cm^{-1}) = 3069 (w) , 2940 (w) (C-H), 2226 (m) (CN), 1706 (s) (C=O).

HRMS (ESI) m/z: [M+H]$^+$ Calcd for $C_{17}H_{13}N_2O$ 261.1028; Found 261.1022.

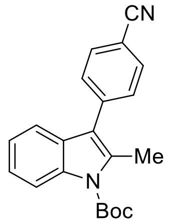

***tert*-butyl 3-(4-cyanophenyl)-2-methyl-1H-indole-1-carboxylate (18):** Synthesized according to general procedure using *tert*-butyl 2-methyl-1H-indole-1-carboxylate (0.35 g, 1.5 mmol, 5 equiv.), potassium phosphate monobasic (82 mg, 0.60 mmol, 2 equiv.), 4-cyanobenzenediazonium tetrafluoroborate (65 mg, 0.30 mmol, 1 equiv.) and DMSO (1 mL, 0.3 M). The reaction was irradiated for 3 hours using 10 W Blue LED. The crude product was purified through column chromatography using 5-10% ethyl acetate in hexanes. Fractions containing product were submitted to further purification using reversed-phase column chromatography using a gradient of 75-100% of Methanol in water. The fractions containing product were then resuspended in a 9:1 mixture of methanol / water and most part of the product precipitated. The title compound was isolated as a white solid (25 mg, 25% yield).

M.P. 145 ºC

^{1}H NMR (500 MHz, CDCl$_3$) δ 8.17 (d, *J* = 8.4 Hz, 1H), 7.77 (d, *J* = 8.3 Hz, 2H), 7.55 (d, *J* = 8.3 Hz, 2H), 7.41 (d, *J* = 7.8 Hz, 1H), 7.34 – 7.28 (m, 1H), 7.25 – 7.19 (m, 1H), 2.60 (s, 3H), 1.72 (s, 9H).

^{13}C NMR (126 MHz, CDCl$_3$) δ 150.7, 139.4, 135.9, 134.8, 132.5, 130.9, 128.7, 124.3, 123.2, 119.6, 119.1, 118.3, 115.7, 110.7, 84.5, 28.4, 15.0.

IR (ATR): $\tilde{\nu}$(cm^{-1}) = 2977 (w) , 2931 (w) (C-H), 2221 (m) (CN), 1724 (s) (C=O).

HRMS (ESI) m/z: [M+H]⁺ Calcd for $C_{21}H_{21}N_2O_2$ 333.1603; Found 333.1598.

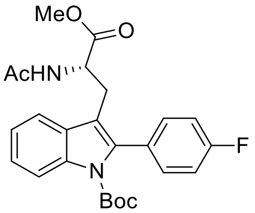

(*S*)-*tert*-butyl-3-(2-acetamido-3-methoxy-3-oxopropyl)-2-(4-fluorophenyl)-1H-indole-1-carboxylate (20): Synthesized according to general procedure using *tert*-butyl (S)-3-(2-acetamido-3-methoxy-3-oxopropyl)-1H-indole-1-carboxylate (0.19 g, 0.53 mmol, 2.5 equiv.), potassium phosphate monobasic (57 mg, 0.42 mmol, 2 equiv.), 4-fluorobenzenediazonium tetrafluoroborate (45 mg, 0.21 mmol, 1 equiv.) and DMSO (0.8 mL, 0.3 M). The reaction was irradiated for 12 hours using 10 W Blue LED. The crude product was purified through column chromatography using 35% ethyl acetate in hexanes. The title compound was isolated as a white solid (67 mg, 70% yield).

^{1}H NMR (500 MHz, $CDCl_3$) (A drop of 2-propanol was added) δ 8.21 (d, *J* = 8.3 Hz, 1H), 7.58 (d, *J* = 7.4 Hz, 1H), 7.35 (ddd, J = 8.4, 7.3, 1.3 Hz, 1H), 7.33 – 7.26 (m, 3H), 7.15 (t, *J* = 8.7 Hz, 2H), 5.77 (d, *J* = 8.0 Hz, 1H), 4.72 (dd, *J* = 14.6, 6.7 Hz, 1H), 3.49 (s, 3H), 3.11 (dd, *J* = 14.5, 6.3 Hz, 1H), 3.02 (dd, *J* = 14.5, 7.0 Hz, 1H), 1.79 (s, 3H), 1.27 (s, 9H).

^{13}C NMR (126 MHz, $CDCl_3$) δ 172.3, 169.7, 162.6 (d, *J* = 248.2 Hz), 150.0, 136.4, 136.3, 131.7 (d, *J* = 8.0 Hz), 129.9 (d, *J* = 3.5 Hz), 129.3, 125.1, 123.1, 118.8, 115.5, 115.5, 115.4 (d, J = 21.6 Hz), 83.6, 52.5, 52.4, 27.7, 27.2, 23.1.

^{19}F NMR (470 MHz, $CDCl_3$) δ -113.34 (tt, *J* = 8.8, 5.5 Hz)

IR (ATR): $\tilde{\nu}$(cm^{-1}) = 1729 (s), 1661 (s) (C=O).

HRMS (ESI) m/z: [M+Na]⁺ Calcd for $C_{25}H_{27}FN_2NaO_5$ 477.1802; Found 477.1803.

$[\alpha]_D^{25}$ = +19.0° (c = 0.8 in $CHCl_3$)

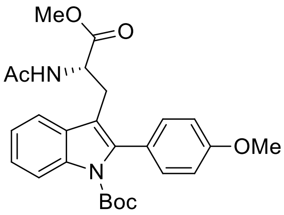

(*S*)-*tert*-butyl-3-(2-acetamido-3-methoxy-3-oxopropyl)-2-(4-methoxyphenyl)-1H-indole-1-carboxylate (21): Synthesized according to general procedure using *tert*-butyl (S)-3-(2-acetamido-3-methoxy-3-oxopropyl)-1H-indole-1-carboxylate (0.19 g, 0.53 mmol, 2.5 equiv.), potassium phosphate monobasic (57 mg, 0.42 mmol, 2 equiv.), 4-methoxybenzenediazonium tetrafluoroborate (47 mg, 0.21 mmol, 1 equiv.) and DMSO (0.8 mL, 0.3 M). The reaction was irradiated for 12 hours using 10 W Blue LED. The crude product was purified through column chromatography using 35% ethyl acetate in hexanes. The title compound was isolated as a white solid (35 mg, 36% yield).

M. P. 174 °C (decomposition)

^{1}H NMR (400 MHz, $CDCl_3$) δ 8.20 (d, *J* = 8.2 Hz, 1H), 7.54 (d, *J* = 7.6 Hz, 1H), 7.34 (t, *J* = 7.6 Hz, 1H), 7.27 (dd, *J* = 15.6, 7.8 Hz, 3H), 7.00 (d, *J* = 8.6 Hz, 2H), 5.83 (d, *J* = 7.7 Hz, 1H), 4.70 (dd, *J* = 13.5, 7.1 Hz, 1H), 3.87 (s, 3H), 3.52 (s, 3H), 3.18 (dd, *J* = 14.5, 5.8 Hz, 1H), 3.08 (dd, *J* = 14.5, 7.2 Hz, 1H), 1.28 (s, 9H).

^{13}C NMR (101 MHz, $CDCl_3$) δ 171.9, 171.4, 159.6, 150.1, 137.5, 136.4, 131.1, 129.2, 126.0, 124.9, 123.0, 118.5, 115.5, 114.6, 114.0, 83.5, 55.5, 52.8, 52.6, 27.8, 26.8, 22.8.

IR (ATR): $\tilde{\nu}$(cm^{-1}) = 1729 (s), 1668 (s) (C=O).

HRMS (ESI) m/z: [M+Na]⁺ Calcd for $C_{26}H_{30}N_2NaO_6$ 489.2002; Found 489.1999.

$[\alpha]_D^{25}$ = +19.0° (c = 0.3 in $CHCl_3$)

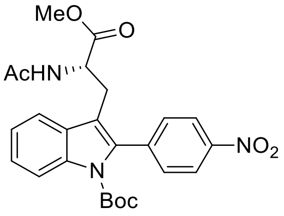

***(S)-tert*-butyl 3-(2-acetamido-3-methoxy-3-oxopropyl)-2-(4-nitrophenyl)-1H-indole-1-carboxylate (22):** Synthesized according to general procedure using *tert*-butyl (S)-3-(2-acetamido-3-methoxy-3-oxopropyl)-1H-indole-1-carboxylate (0.19 g, 0.53 mmol, 2.5 equiv.), potassium phosphate monobasic (57 mg, 0.42 mmol, 2 equiv.), 4-nitrobenzenediazonium tetrafluoroborate (50 mg, 0.21 mmol, 1 equiv.) and DMSO (0.8 mL, 0.3 M). The reaction was irradiated for 12 hours using 10 W Blue LED. The crude product was purified through column chromatography using 35% ethyl acetate in hexanes. The title compound was isolated as a yellow solid (91 mg, 90% yield).

M. P. 156 ºC (decomposition)

^{1}H NMR (400 MHz, CDCl$_3$) δ 8.32 (d, *J* = 8.5 Hz, 2H), 8.21 (d, *J* = 8.3 Hz, 1H), 7.63 (d, *J* = 7.7 Hz, 1H), 7.54 (d, *J* = 8.5 Hz, 2H), 7.38 (t, *J* = 7.7 Hz, 1H), 7.31 (t, *J* = 7.4 Hz, 1H), 5.89 (d, *J* = 7.8 Hz, 1H), 4.74 (q, *J* = 7.0 Hz, 1H), 3.48 (s, 3H), 3.17 – 3.00 (m, 2H), 1.79 (s, 3H), 1.30 (s, 9H).

^{13}C NMR (101 MHz, CDCl$_3$) δ 172.1, 149.7, 147.3, 140.8, 136.6, 134.8, 130.9, 129.3, 125.7, 123.4, 119.2, 116.9, 115.7, 84.3, 52.6, 52.3, 27.8, 27.5, 23.1.

IR (ATR): $\tilde{\nu}$(cm^{-1}) = 1731 (s), 1667 (s) (C=O), 1519 (s) (N-O).

HRMS (ESI) m/z: [M+Na]$^+$ Calcd for $C_{25}H_{27}N_3NaO_7$ 504.1747 Found 504.1744.

[α]$_D^{25}$ = +12.6º (c = 1.0 in CHCl$_3$)

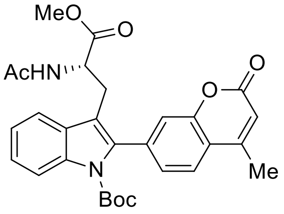

***(S)-tert*-butyl (S)-3-(2-acetamido-3-methoxy-3-oxopropyl)-2-(4-methyl-2-oxo-2H-chromen-7-yl)-1H-indole-1-carboxylate (23):** Synthesized according to general procedure using *tert*-butyl (S)-3-(2-acetamido-3-methoxy-3-oxopropyl)-1H-indole-1-carboxylate (0.19 g, 0.53 mmol, 2.5 equiv.), potassium phosphate monobasic (57 mg, 0.42 mmol, 2 equiv.), 4-methyl-2-oxo-2H-chromene-7-diazonium tetrafluoroborate (58 mg, 0.21 mmol, 1 equiv.) and DMSO (0.8 mL, 0.3 M). The reaction was irradiated for 12 hours using 10 W Blue LED. The crude product was purified through column chromatography using 35% ethyl acetate in hexanes. The title compound was isolated as a yellow oil (50 mg, 46% yield).

^{1}H NMR (400 MHz, CDCl$_3$) δ 8.20 (d, *J* = 8.3 Hz, 1H), 7.73 – 7.67 (m, 1H), 7.59 (d, *J* = 7.8 Hz, 1H), 7.39 (ddd, *J* = 8.4, 7.3, 1.3 Hz, 1H), 7.35 – 7.29 (m, 3H), 6.37 (d, *J* = 1.2 Hz, 1H), 5.99 (d, *J* = 7.9 Hz, 1H), 4.72 (q, *J* = 6.9 Hz, 1H), 3.51 (s, 3H), 3.17 (dd, *J* = 14.4, 6.8 Hz, 1H), 3.10 (dd, *J* = 14.4, 6.8 Hz, 1H), 2.51 (d, *J* = 1.2 Hz, 3H), 1.86 (s, 3H), 1.33 (s, 9H).

^{13}C NMR (101 MHz, CDCl$_3$) δ 171.9, 171.3, 161.0, 153.3, 152.5, 149.8, 137.6, 136.5, 135.6, 129.2, 126.1, 125.6, 124.5, 123.4, 119.7, 119.0, 118.3, 116.1, 115.8, 115.6, 84.3, 52.8, 52.6, 29.8, 27.8, 27.4, 22.8, 18.8.

IR (ATR): $\tilde{\nu}$(cm^{-1}) = 2923 (w), 2850 (w) (C-H), 1728 (s), 1670 (m), 1619 (m) (C=O).

HRMS (ESI) m/z: [M+Na]$^+$ Calcd for $C_{29}H_{30}N_2NaO_7$ 541.1951. Found 541.1948.

[α]$_D^{25}$ = +4.0º (c = 0.45 in CHCl$_3$)

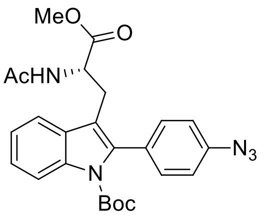

***(S)-tert*-butyl 3-(2-acetamido-3-methoxy-3-oxopropyl)-2-(4-azidophenyl)-1H-indole-1-carboxylate (24):** Synthesized according to general procedure using *tert*-butyl (S)-3-(2-acetamido-3-methoxy-3-oxopropyl)-1H-indole-1-carboxylate (0.19 g, 0.53 mmol, 2.5 equiv.), potassium phosphate monobasic (57 mg, 0.42 mmol, 2 equiv.), 4-azidobenzenediazonium tetrafluoroborate (49 mg, 0.21 mmol, 1 equiv.) and DMSO (0.8 mL, 0.3 M). The reaction was irradiated for 12 hours using 10 W Blue LED. The crude product was purified through column chromatography using 35% ethyl acetate in hexanes. The title compound was isolated as a yellow solid (53 mg, 53% yield).

^{1}H NMR (400 MHz, $CDCl_3$) δ 8.20 (d, *J* = 8.2 Hz, 1H), 7.59 (d, *J* = 7.7 Hz, 1H), 7.32 (ddd, *J* = 23.3, 15.0, 8.7 Hz, 4H), 7.12 (d, *J* = 8.4 Hz, 2H), 5.71 (d, *J* = 7.8 Hz, 1H), 4.73 (dd, *J* = 14.4, 6.9 Hz, 1H), 3.50 (s, 3H), 3.12 (dd, *J* = 14.4, 6.3 Hz, 1H), 3.04 (dd, *J* = 14.4, 7.0 Hz, 1H), 1.79 (s, 3H), 1.30 (s, 9H).

^{13}C NMR (101 MHz, $CDCl_3$) δ 172.3, 169.6, 150.0, 140.0, 136.5, 136.4, 131.4, 130.5, 129.4, 125.1, 123.1, 118.9, 118.9, 115.6, 115.5, 83.7, 52.5, 52.4, 27.8, 27.7, 27.2, 23.2.

IR (ATR): $\tilde{\nu}$(cm^{-1}) = 2121 (m) (N_3), 1731 (s), 1672 (s) (C=O).

HRMS (ESI) m/z: [M+Na]$^+$ Calcd for $C_{25}H_{27}N_5NaO_5$ 500.1910. Found 504.1908.

$[\alpha]_D^{25}$ = +21.2° (c = 1.0 in $CHCl_3$)

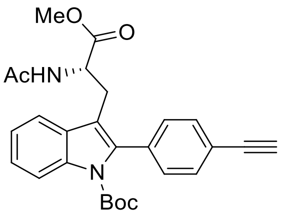

***(S)-tert*-butyl 3-(2-acetamido-3-methoxy-3-oxopropyl)-2-(4-ethynylphenyl)-1H-indole-1-carboxylate (25):** Synthesized according to general procedure using *tert*-butyl (S)-3-(2-acetamido-3-methoxy-3-oxopropyl)-1H-indole-1-carboxylate (0.19 g, 0.53 mmol, 2.5 equiv.), potassium phosphate monobasic (57 mg, 0.42 mmol, 2 equiv.), 4-ethynylbenzenediazonium tetrafluoroborate (45 mg, 0.21 mmol, 1 equiv.) and DMSO (0.8 mL, 0.3 M). The reaction was irradiated for 12 hours using 10 W Blue LED. The crude product was purified through column chromatography using 35% ethyl acetate in hexanes. The title compound was isolated as a yellow solid (41 mg, 42% yield). Handling observations: This substance is unstable at temperatures higher than 35 °C.

^{1}H NMR (400 MHz, $CDCl_3$) δ 8.22 (d, *J* = 8.2 Hz, 1H), 7.60 (t, *J* = 8.0 Hz, 3H), 7.40 – 7.33 (m, 1H), 7.33 – 7.27 (m, 3H), 5.64 (d, *J* = 7.9 Hz, 1H), 4.74 (dd, *J* = 14.3, 6.6 Hz, 1H), 3.49 (s, 3H), 3.23 – 3.11 (m, 2H), 3.06 (dd, *J* = 14.5, 6.9 Hz, 1H), 1.78 (s, 3H), 1.26 (s, H).

^{13}C NMR (101 MHz, $CDCl_3$) δ 172.2, 149.9, 136.6, 136.5, 134.5, 132.0, 130.0, 129.4, 125.2, 123.2, 121.9, 119.0, 115.6, 115.5, 83.8, 83.3, 78.4, 52.5, 52.4, 29.8, 27.7, 27.1, 23.2.

IR (ATR): $\tilde{\nu}$(cm^{-1}) = 3296 (m) (C≡C-H), 1731 (s), 1660 (s) (C=O).

HRMS (ESI) m/z: [M+Na]$^+$ Calcd for $C_{27}H_{28}N_2NaO_5$ 483.1896. Found 483.1888.

$[\alpha]_D^{25}$ = +18.9° (c = 0.35 in $CHCl_3$)

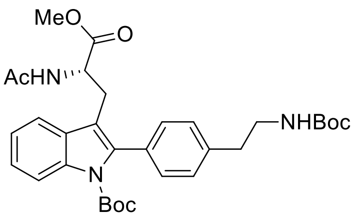

***(S)-tert*-butyl 3-(2-acetamido-3-methoxy-3-oxopropyl)-2-(4-(2-((tert-butoxycarbonyl)amino)ethyl)phenyl)-1H-indole-1-carboxylate (26):** Synthesized according to general procedure using *tert*-butyl (S)-3-(2-acetamido-3-methoxy-3-oxopropyl)-1H-indole-1-carboxylate (0.19 g, 0.53 mmol, 2.5 equiv.), potassium phosphate monobasic (57 mg, 0.42 mmol, 2 equiv.), 4-(2-((*tert*-butoxycarbonyl)amino)ethyl)benzenediazonium tetrafluoroborate (70 mg, 0.21 mmol, 1 equiv.) and DMSO (0.8 mL, 0.3 M). The reaction was irradiated for 12 hours using 10 W Blue LED. The crude product was purified through column chromatography using 35% ethyl acetate in hexanes. The title compound was isolated as a yellow solid (55 mg, 45% yield).

^{1}H NMR (400 MHz, CDCl$_3$) δ 8.23 (d, *J* = 8.2 Hz, 1H), 7.54 (d, *J* = 7.5 Hz, 1H), 7.39 – 7.32 (m, 1H), 7.32 – 7.23 (m, 5H), 5.80 (d, *J* = 6.6 Hz, 1H), 4.66 (q, *J* = 6.7 Hz, 1H), 3.49 (s, 3H), 3.41 (s, 2H), 3.15 (dd, J = 14.4, 6.2 Hz, 1H), 3.11 – 3.01 (m, 1H), 2.87 (t, *J* = 7.1 Hz, 2H), 1.82 (s, 3H), 1.23 (s, 9H).

^{13}C NMR (101 MHz, CDCl$_3$) δ 172.1, 170.7, 150.0, 137.4, 136.5, 132.1, 130.2, 129.3, 128.8, 125.0, 123.1, 118.6, 115.5, 114.9, 83.4, 52.6, 52.6, 36.1, 29.8, 28.6, 27.6, 27.0, 23.0.

IR (ATR): $\tilde{\nu}$(cm^{-1}) = 1728 (s) (C=O).

HRMS (ESI) m/z: [M+Na]$^+$ Calcd for $C_{32}H_{41}N_3NaO_7$ 602.2842. Found 602.2841.

[α]$_D^{25}$ = +23.6° (c = 0.250 in CHCl$_3$)

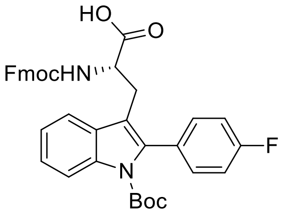

***(S)*-2-((((9H-fluoren-9-yl)methoxy)carbonyl)amino)-3-(1-(*tert*-butoxycarbonyl)-2-(4-fluorophenyl)-1H-indol-3-yl)propanoic acid (27):** Synthesized according to general procedure using *(S)*-2-((((9H-fluoren-9-yl)methoxy)carbonyl)amino)-3-(1-(*tert*-butoxycarbonyl)-1H-indol-3-yl)propanoic acid (0.28 g, 0.53 mmol, 2.5 equiv.), potassium phosphate monobasic (57 mg, 0.42 mmol, 2 equiv.), 4-fluorobenzenediazonium tetrafluoroborate (45 mg, 0.21 mmol, 1 equiv.) and DMSO (0.8 mL, 0.3 M). The reaction was irradiated for 12 hours using 10 W Blue LED. The crude product was purified through column chromatography using 50% ethyl acetate in hexanes. The title compound was isolated as a white solid (52 mg, 40% yield). The starting material could also be recovered in good extent (0.14 g, 50% recover).

^{1}H NMR (400 MHz, CDCl$_3$) δ 8.26 (d, *J* = 8.3 Hz, 1H), 7.75 (t, *J* = 7.8 Hz, 2H), 7.63 (d, *J* = 7.7 Hz, 1H), 7.49 (dd, *J* = 15.1, 7.5 Hz, 2H), 7.43 – 7.33 (m, 4H), 7.27 (dq, *J* = 14.4, 7.0 Hz, 4H), 7.05 (d, *J* = 7.8 Hz, 2H), 4.86 (d, *J* = 7.9 Hz, 1H), 4.50 (dd, *J* = 13.4, 7.9 Hz, 1H), 4.34 (d, *J* = 6.6 Hz, 2H), 4.17 – 4.05 (m, 1H), 3.20 (dd, *J* = 14.6, 5.4 Hz, 1H), 2.97 (dd, *J* = 14.5, 8.4 Hz, 1H), 1.27 (s, 9H).

^{13}C NMR (101 MHz, CDCl$_3$) δ 176.5, 176.0, 155.9, 149.9, 143.9, 142.5 (d, *J* = 223.0 Hz), 141.4, 136.5, 131.8 (d, *J* = 7.6 Hz), 129.7 (d, *J* = 3.5 Hz), 129.0, 127.9, 127.2, 127.2, 125.2, 123.3, 120.1, 118.7, 115.7, 115.5 (d, *J* = 21.6 Hz), 115.3, 83.6, 67.0, 53.9, 47.2, 27.8, 20.7.

^{19}F NMR (376 MHz, CDCl$_3$) δ -114.03.

IR (ATR): $\tilde{\nu}$(cm^{-1}) = 2927 (w) (C-H), 1721 (m) (C=O).

HRMS (ESI) m/z: [M+Na]$^+$ Calcd for $C_{37}H_{33}F_2N_2NaO_6$ 643.2220. Found 643.2225.

[α]$_D^{25}$ = +11.2° (c = 0.25 in CHCl$_3$)

4. Mechanistic Experiments

4.1. Evidence of EDA complex formation:

A UV-Vis absorbance experiment has been carried out to confirm EDA complex formation. Spectra of *N*-Boc-Indole (0.15 M) and *p*-fluorobenzenediazonium tetrafluorborate (0,15 M) solutions in DMSO were recorded as controls. Next, we added the solid diazonium salt to a solution of *N*-Boc Indole in DMSO, keeping a final concentration of 0.15 M for each component, and recorded the UV-Vis spectrum of the mixture. An evident bathochromic shift can be observed, strongly indicating the formation of a charge transfer complex between these two reactants. There’s also visual evidence for this complexation, since the mixture acquires an intense yellow color.

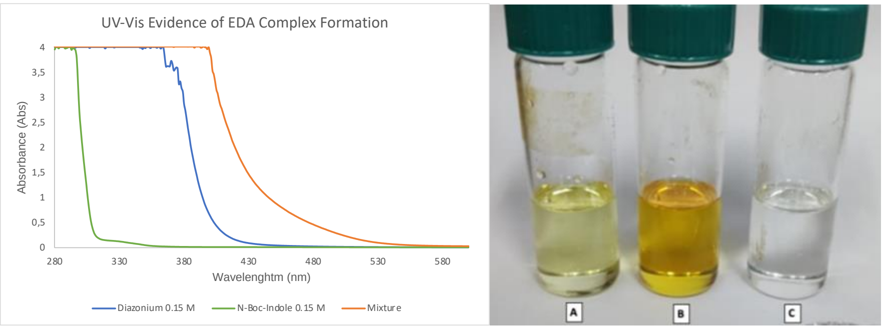

Figure S1: Evidence for EDA complex formation. <u>Left:</u> UV-Vis spectra of a 0.15 M *N*-Boc-Indole solution (green), 0.15 M Diazonium salt solution (blue) and the 1:1 mixture (orange, final concentration of 0.15 M for each compound) in DMSO. <u>Right:</u> From left to right, the above-mentioned solutions of Diazonium salt (A), the mixture (B) and *N*-Boc Indole (C).

The difference spectrum indicates a maximum of 399 nm for EDA complex absorbance. This plot was made with the difference in absorbance values for the mixture compared to the sum of the absorbances of the individual components for each wavelength.

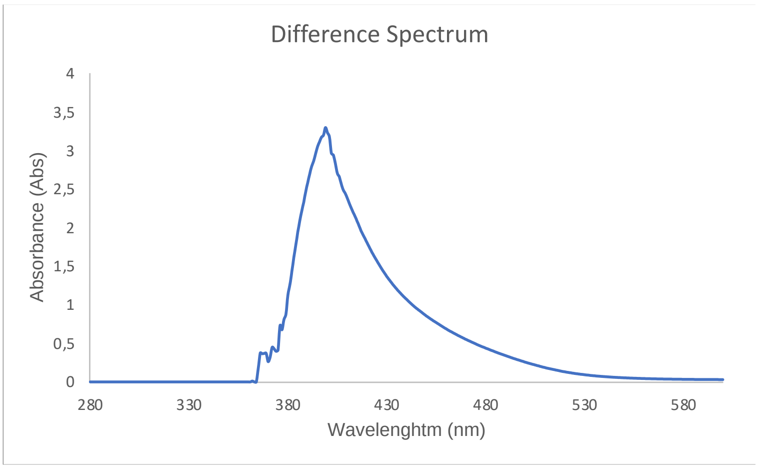

Figure S2: Difference spectrum showing the CT band formed with the interaction of N-Boc Indole and the Diazonium salt.

We observed the formation of the complex through time by recording UV-Vis spectra immediately after mixing diazonium salt and *N*-Boc-Indole and on 5 minutes intervals. The solution was kept in the dark inside spectrophotometer while waiting for the next run. Absorbance in 399 nm indicates a very clear kinetic profile for this EDA formation, showing it is not instantaneously formed in a reasonable extent, but requires at least 15 minutes for stabilization.

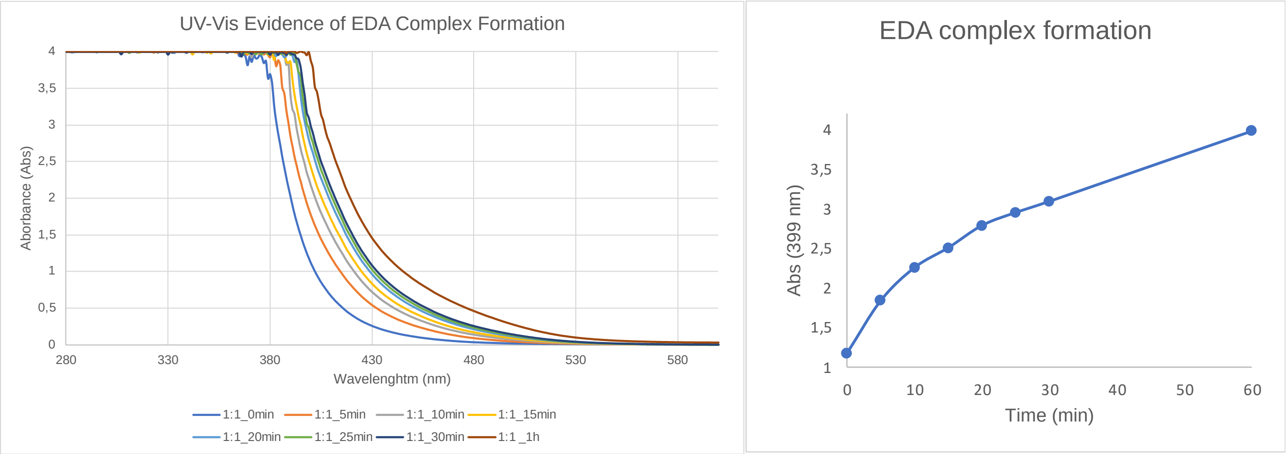

Figure S3: EDA complex time study. <u>Left:</u> Spectra of the mixture of *N*-Boc Indole and Diazonium salt in several times. <u>Right:</u> Isolated absorbances in 399 nm in each time.

To exclude the possibility of a side reaction between diazonium salt and *N*-Boc indole being responsible for spectral differences, a ^{1}H-^{15}N HMBC NMR of the mixture was recorded. The analysis showed no correlations of any hydrogen from Indole structure with the nitrogen from diazonium salt and *vice-versa*. These results indicate no new covalent bonds were formed, corroborating the hypothesis that spectroscopic differences are due to the formation of a charge transfer complex.

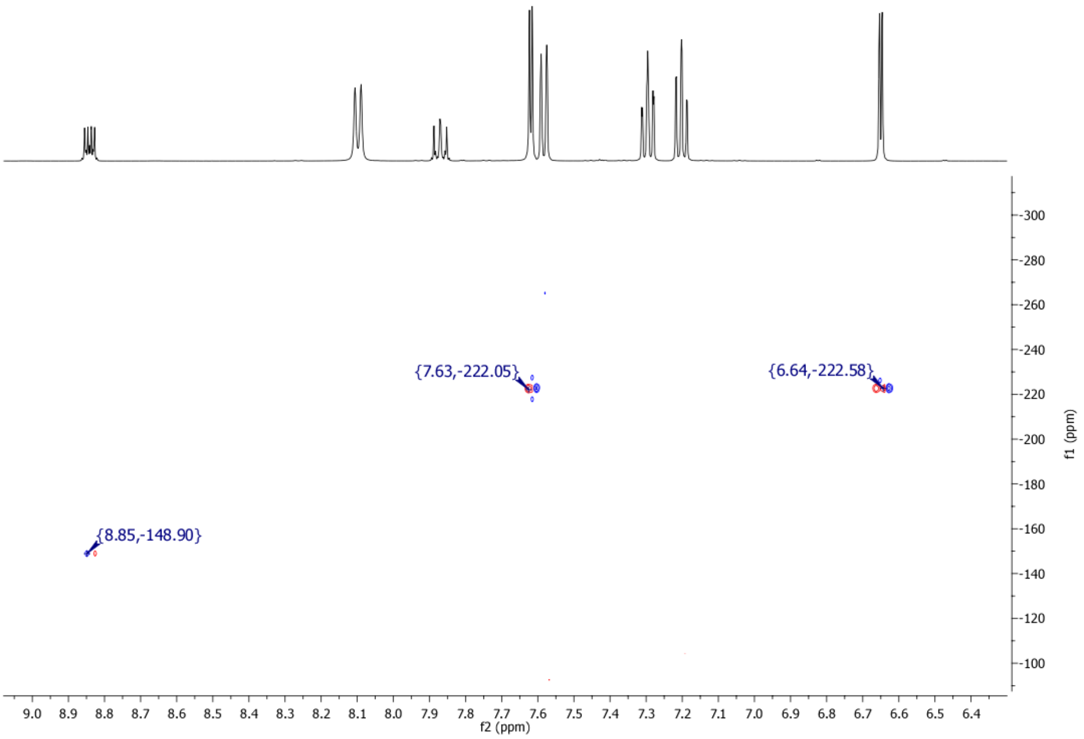

Figure S4: ^{1}H-^{15}N HMBC analysis of *N*-Boc Indole and 4-fluorobenzenediazonium tetrafluoroborate mixture. Horizontal trace is adorned with ^{1}H NMR spectra recorded from the same sample. Vertical trace refers to ^{15}N chemical shifts, -222 ppm referring to the *N*-Boc-Indole nitrogen and -149 ppm to the nitrogen directly attached to aromatic ring in diazonium salt structure. (DMSO-d6, 500 MHz)

4.2. ^{1}H NMR titration experiment

Another evidence for EDA complex formation could be extracted from ^{1}H NMR analysis. Different amounts of Diazonium salt were added to solutions of *N*-Boc-Indole in 150 μL of DMSO and 450 μL of $CDCl_3$. Deuterated solvent residual peak was used as internal standard. The sum of the components concentrations was kept constant and equal to 0.3 M. Seven solutions with molar fractions of 0, 0.1, 0.3, 0.5, 0.7, 0.9 and 1.0 of N-Boc Indole were analyzed, and the results can be seen in figure S5.

It is possible to see a downfield shift of both diazonium and H(7) *N*-Boc-Indole signals when increasing the concentration of the former, indicating EDA complex formation.

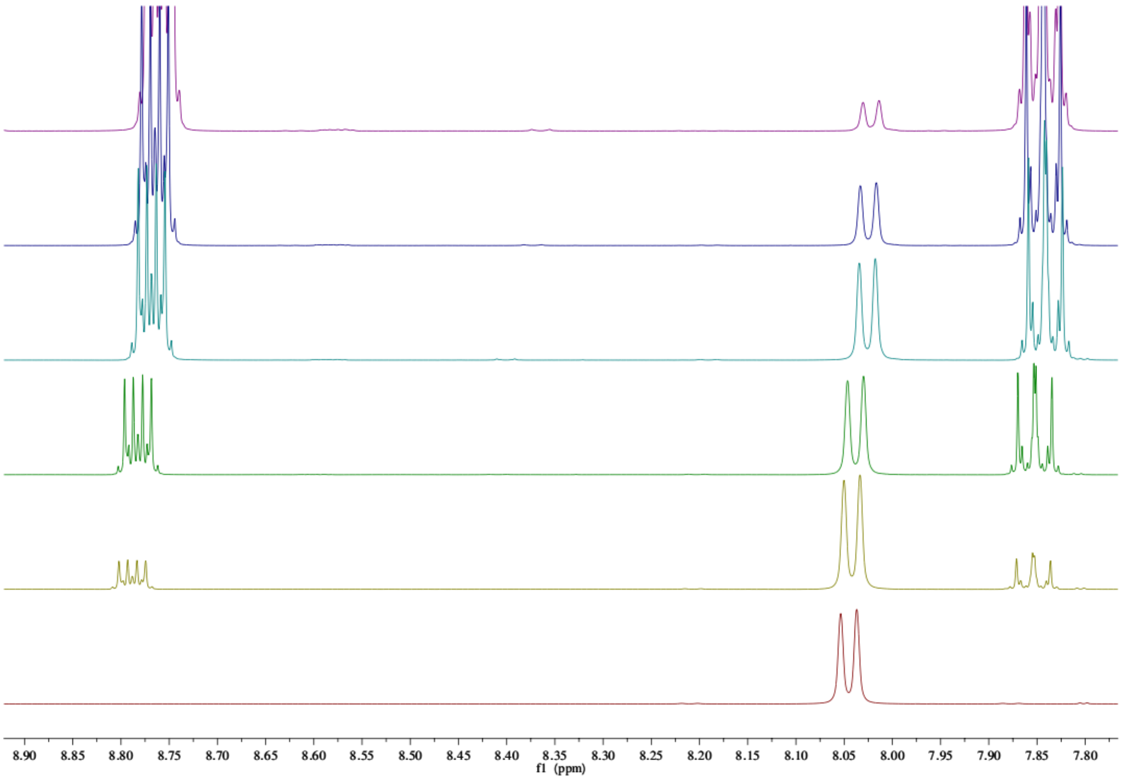

Figure S5: ^1H NMR titration. Increasing molar ratio of *N*-Boc indole from top to bottom. Downfield shift of both diazonium salt and *N*-Boc-Indole is evident.

4.3. Determination of EDA complex stoichiometry

The binding stoichiometry for EDA complex was determined through the method of continuous variation (Job's Plot analysis) using both ^1H NMR spectroscopy[20] and UV-Vis spectrophotometry.

With data obtained from NMR titration, we analyzed the effect of *N*-Boc-Indole molar fraction in chemical shift variation when compared to the pure substance.

Considering the complexation has a fast exchange rate compared to the NMR time scale, the observed *N*-Boc-Indole chemical shift ($\delta_{observed}$) is a weighted average of the signal from the free indole (δ_{free}) and from the complexed one (δ_c, not observable).

We plotted data of *N*-Boc-Indole concentration and chemical shift variation ratio against *N*-Boc-Indole molar fraction. Data can be seen in table and graphic below.

Table S2: Data from ^1H NMR Titration. $\Delta\delta$ refers to the difference between the chemical shift of H(7) in that sample and standard chemical shift from pure *N*-Boc-Indole.

Mole Fraction (*N*-Boc Indole)	[*N*-Boc-Indole] (mol/L)	δ (ppm)	[*N*-Boc-Indole]x Δδ
0,1	0,06	8,0221	0,001392
0,3	0,18	8,025	0,003654
0,5	0,3	8,026	0,005790
0,7	0,42	8,0382	0,002982
0,9	0,54	8,0419	0,001836
1	0,6	8,0453	0,000000

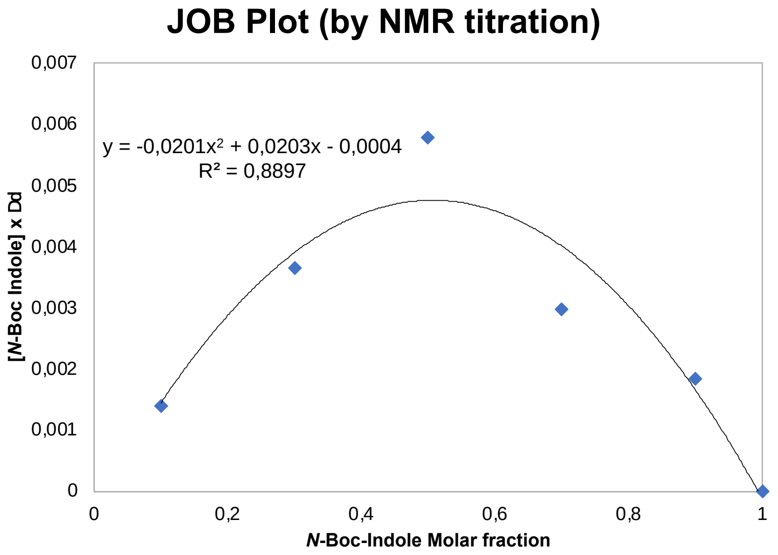

Figure S6: Curve from Table S2 data. It is possible to see maximum interaction at 1:1 binding stoichiometry.

The quadratic regression of data distribution gives the function shown in graphic, which has a maximum value of -b/2a = 0.51, indicating a binding stoichiometry of 1:1 for the EDA complex between diazonium salt and *N*-Boc-Indole.

UV-Vis data corroborates these conclusions. UV-Vis Spectra of diazonium salt and *N*-Boc-Indole mixtures were recorded in DMSO. Solutions were prepared keeping the sum of the components' final concentrations constant and equal to 0.3 M. Three mixtures containing 2:1, 1:1 and 1:2 ratios of *N*-Boc-Indole and diazonium salt, respectively, were recorded and the 399 nm absorbance was maximum when using equal amounts of both components, corroborating the 1:1 binding stoichiometry for the EDA complex.

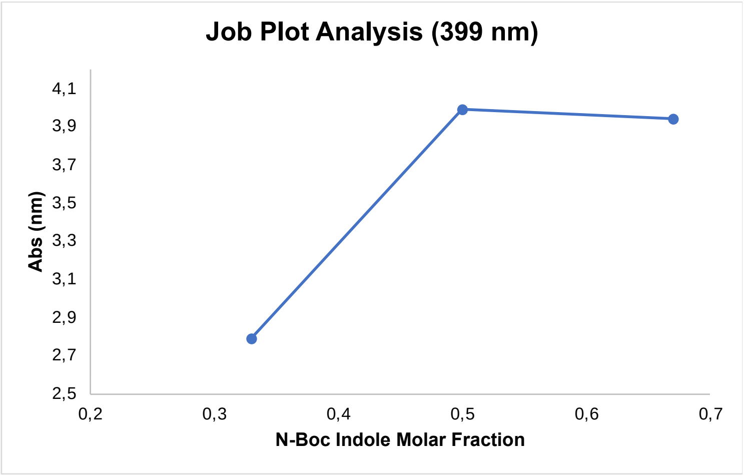

Figure S7: UV-Vis absorbance in 399 nm of mixtures of *N*-Boc Indole and Diazonium salt in different molar fractions of the indole.

4.4. ON/OFF experiments

To determine if there was any significant propagation mechanism operating in the reaction, an ON/OFF experiment was designed. A standard reaction was carried out, and light irradiation was interrupted after 10 minutes, keeping the reaction in the dark for the same time before light irradiation was restarted. This cycle was repeated until reaction time reached 60 minutes. Then, the intervals jumped to 1 hour (under irradiation and in the dark) until the 4 hours of reaction were completed. A 50 μL sample was taken to monitor reaction progress at the start and at every time interval the light was switched either ON or OFF.

Reaction was monitored using ^{1}H NMR. The final reaction volume was measured to be 1.3 mL, so a stock solution of 1,3-Benzodioxole used as internal standard was prepared dissolving 37 mg (0.3 mmol) in 1.3 mL of $CDCl_3$. The 50 µL of reaction sample was mixed with 50 µL of benzodioxole stock solution, 100 µL of DMSO and 400 µL of $CDCl_3$.

Results can be seen in the figure below. They indicate that product (orange line) in only formed during irradiation periods, (blue areas in the figure) which is evidence that there is no substantial contribution of propagation mechanisms. However, it is clear from the results that the diazonium salt degradation happens irrespective of light irradiation through a thermal path, indicating that the desired arylation reaction competes with aryl diazonium salt degradation.

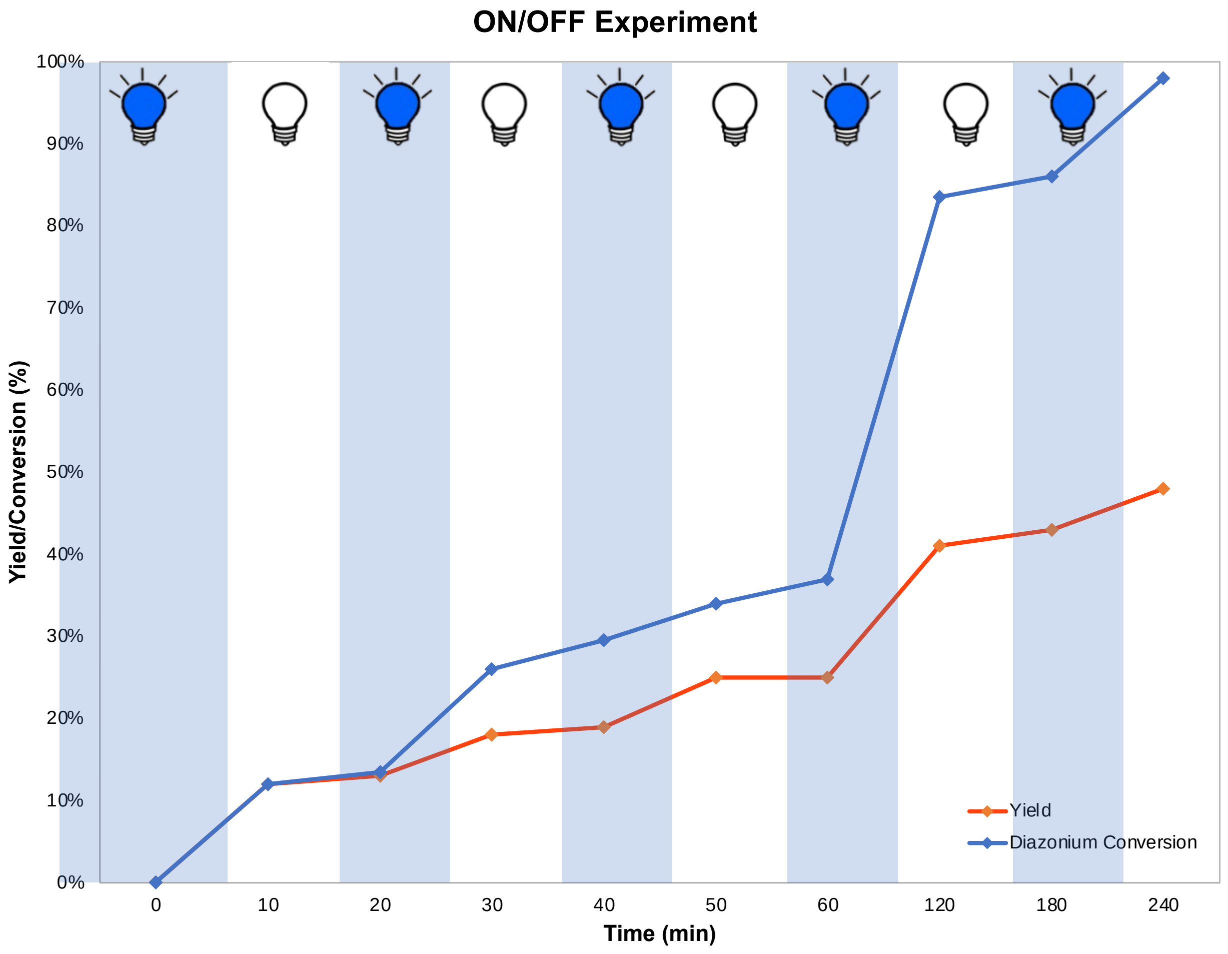

Figure S8: ON-OFF Experiment. Blue areas indicate Blue LED irradiation was ON. The x-axis is not linear for illustrative purposes.

4.5. Radical Trapping with TEMPO

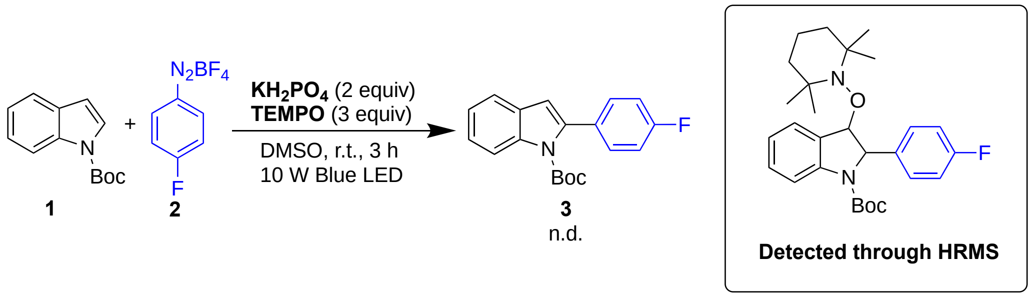

Scheme S6: Radical trapping experiment using TEMPO as radical scavenger.

Procedure: A vial containing a magnetic stir bar was charged with potassium phosphate monobasic (82 mg, 0.60 mmol, 2 equiv.), *N*-Boc-Indole (0.33 g, 1.5 mmol, 5 equiv.) and DMSO (1 mL, 0.3 M). Then, diazonium salt **2** (63 mg, 0.30 mmol, 1 equiv.) was

added to the reaction mixture. After homogenization, TEMPO (0.14 g, 0.90 mmol, 3 equiv.) was carefully included. Reaction was purged with argon for 15 minutes before it was submitted to light irradiation with 10 W blue LED for 3 hours. 36.6 mg of 1,3-Benzodioxole were added and after complete homogenization a 50 μL sample was taken for ^{1}H NMR analysis. The sample was diluted in 100 μL of DMSO and 450 μL of $CDCl_3$ and NMR data was acquired using a relaxation delay of 40 seconds and an observe pulse of 90 degrees. NMR analysis showed no detectable amounts of arylated product, which is strong evidence that it is formed through a radical mechanism.

Crude reaction mixture was also submitted to HRMS by direct infusion, and the benzylic radical intermediate **39** could be detected efficiently.

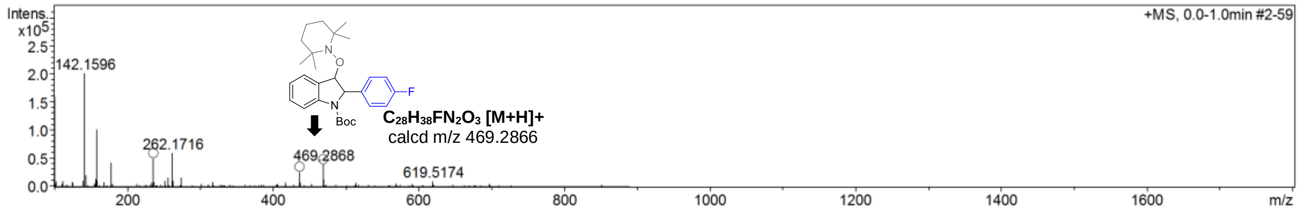

Figure S9: HRMS of radical trapping experiment using TEMPO as radical scavenger.

4.6. Mechanistic hypothesis

Based on the above experiments, we propose a reasonable mechanism (. First step involves the formation of an EDA complex between diazonium salt and *N*-Boc-Indole. This complex can absorb blue light irradiation, and, once excited, lead to a SET event resulting in the aryl radical **37** after nitrogen release.

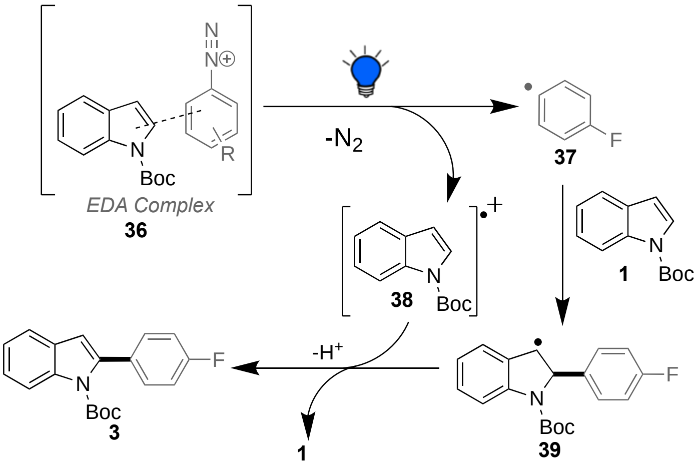

Scheme S7: Plausible reaction mechanism.

This aryl radical intermediate can then add to a neutral *N*-Boc-indole, leading to benzylic radical **39** formation. Product is then formed after oxidation and re-aromatization. The oxidating agent in this step is probably the *N*-Boc-Indole radical cation **38**.

4.7. Kinetic evidence for mechanism switch in the presence of Eosin Y photocatalyst

The similar yields obtained both in the presence and absence of Eosin Y (Table S1, entries 1 and 2) can mislead to the conclusion that the photocatalyst has no participation in reaction when it is present. However, kinetic data raises strong evidence that Eosin Y does participate in reaction mechanism when present.

Three standard reactions were set up with 3 mol%, 1 mol% and no Eosin Y added to the reaction mixture. A 50 μL sample was taken from each reaction before light irradiation and on 10-minute intervals. This sample was then diluted in 100 μL of DMSO and 400 μL of $CDCl_3$ before 50 μL of a stock solution of 37 mg of 1,3-benzodioxole (0.3 mmol) in 1.3 mL of $CDCl_3$ (final reaction volume) was added. The diluted samples were kept in the freezer while waiting for analysis. ^{1}H NMR analysis was performed to monitor reaction

progress, and the initial progress can be approximated to a linear range to give the initial reaction rates. Results are shown in figure S10 below.

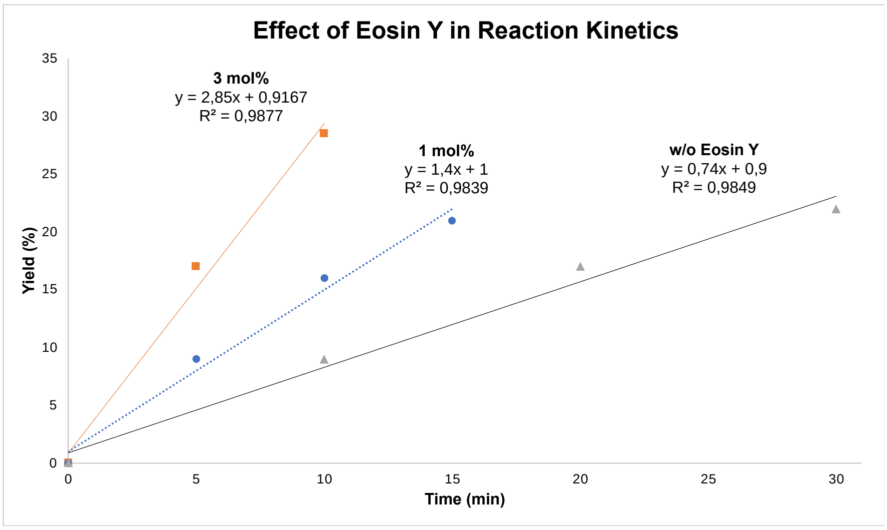

Figure S10: Kinetic data from reactions with different amounts of Eosin Y.

It is clear from the results that although reaction proceeds very well without photocatalyst, it is faster when in the presence of Eosin Y, and that higher amounts of Eosin Y led to higher reaction rates. These results indicate that Eosin Y participates in reaction mechanism, probably being responsible for diazonium salt reduction and benzylic radical oxidation steps.

4.8. Quantum Yield Measurement

Ferrioxalate actinometer solution measures the decomposition of ferric ions to ferrous ions, which are complexed by 1,10-phenanthroline and monitored by UV/Vis absorbance at 510 nm. The moles of iron-phenanthroline complex formed are related to moles of photons absorbed.[21]

1. The solutions were prepared and stored in the dark:
   - Potassium ferrioxalate solution: 1.179 g of potassium ferrioxalate and 556 μL of sulfuric acid (96%) were added to a 100 mL volumetric flask and filled to the mark with distilled water.
   - Phenanthroline solution: 0.2% by weight of 1,10-phenanthroline in water (200 mg in 100mL volumetric flask).
   - Buffer solution: to a 100 mL volumetric flask 4.94 g of NaOAc and 1 mL of sulfuric acid (96%) were added and filled to the mark with distilled water.
2. The actinometry measurements were done as follows:
   - 1 mL of the actinometer solution was added to a vial and placed 1 cm away from the 10W Blue LED lamp (450-460 nm) and irradiated for 5, 10, 20, 40, 60 seconds.
   - After irradiation all the actinometer solution was removed and placed in a 10 mL volumetric flask. 1 mL of 1,10-phenanthroline solution and 4 mL of buffer solution was added to this flask and filled to the mark with distilled water.
   - The UV-Vis spectra of actinometry samples were recorded for each time interval. The absorbance of the actinometry solution was monitored at 510 nm.

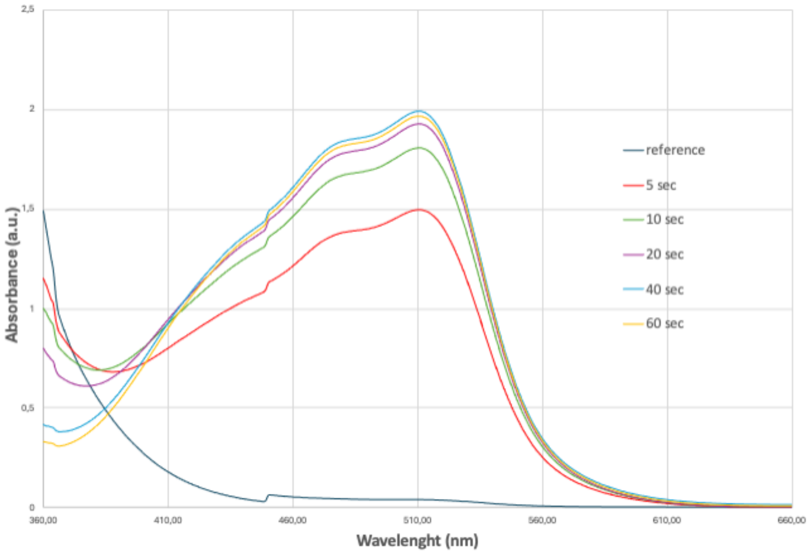

Figure S11: UV-Vis spectra of actinometry samples irradiated from 5 to 60 seconds.

3. The moles of Fe2+ formed for each sample is determined using Beers' Law:
   - Mols of Fe(II) = $[V1 \times V3 \times \Delta A(510\ \text{nm})]/[10^3 \times V2 \times l \times \varepsilon(510\ nm)]$

where $V1$ is the irradiated volume (1 mL), $V2$ is the aliquot of the irradiated solution taken for the determination of the ferrous ions (1 mL), $V3$ is the final volume after complexation with phenanthroline (10 mL), l is the optical path-length of the irradiation cell (1 cm), ΔA (510 nm) the optical difference in absorbance between the irradiated solution and that taken in the dark, ε (510 nm) is that of the complex $Fe(phen)_3{}^{2+}$ (11100 L mol^{-1} cm^{1}).

4. The moles of Fe^{2+} formed (N) are plotted as a function of time (t). The slope is a product of the photon flux (F) and the quantum yield for Fe^{2+} (φFe^{2+} = 1.13), F = N/ΦFe^{2+} t. The F was determined to be 9.51×10^{-6} einstein min^{-1}.

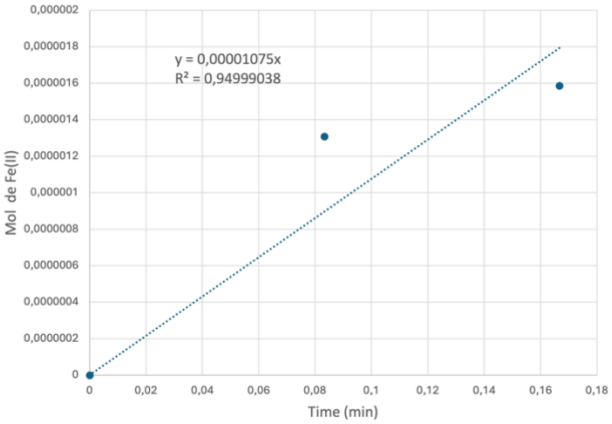

Figure S12: Determination of photon flux (F)

5. The moles of products formed for the indole arylation are described above. The moles of products formed were determined by qNMR using 1,3-benzodioxole as reference standard. The number of moles of product per unit time is related to the number of photons absorbed. The slope yields the quantum yield (Φ) of the photoreaction: Φ =0.25.

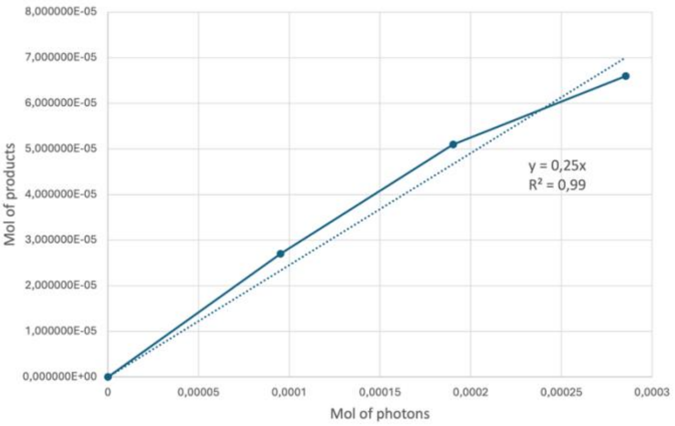

Figure S13: Determination of quantum yield (Φ) of the photoreaction.

5. HPLC Traces

5.1. Optical purity of 20

To verify the degree of racemization during reaction conditions, the product **20** was selected as an example and submitted to chiral HPLC analysis. The analysis was carried out on a Shimadzu Prominence LC-20A equipped with a PDA detector, using a Lux 5mm Cellulose-2 LC Column 250 x 4.6 mm. Mobile phases comprised HPLC grade Hexanes (Mobile phase A) and HPLC grade Isopropanol (mobile phase B). The method for chromatographic separation consisted of an isocratic run using 90% B for 90 minutes. The flow rate was set to 1 mL.min^{-1} and the injection volume was 10 μL. Column oven temperature was kept at room temperature throughout the analysis. The substances were detected spectrophotometrically at λ=254 nm.

A 1 mg.mL^{-1} solution of both racemic and enantiopure fully protected starting material **63** was injected and results are shown in figure S14.

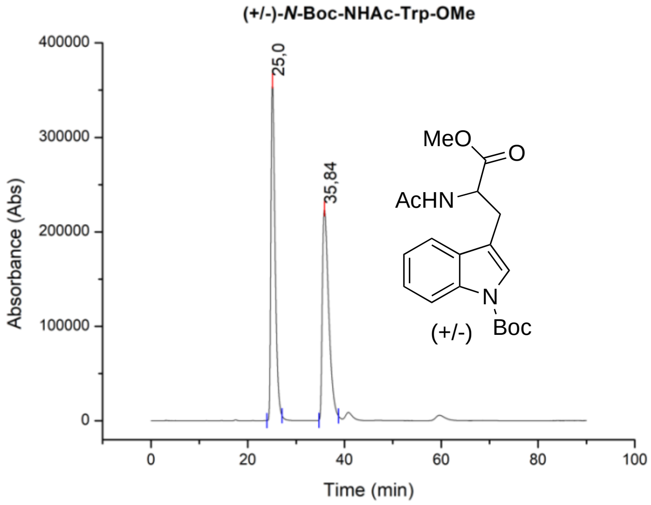

#	Retention Time (min)	Area	Relative Area (%)	*e.r.*
1	25.099	363705.182	51.6%	
2	35.840	341477.9315	48.4%	1 : 1

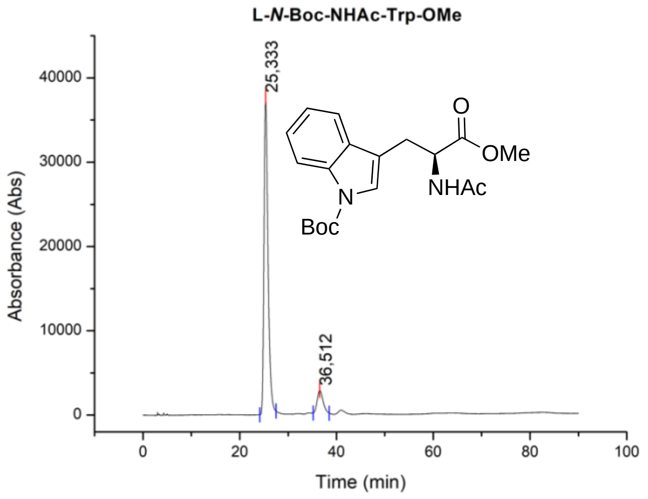

#	Retention Time (min)	Area	Relative Area (%)	*e.r.*
1	25.333	36884.2172	89.0%	
2	36.512	4545.63351	11.0%	89 : 11

Figure S14: HPLC traces of: (Top) Racemic starting material; (bottom) enantiopure starting material **63**. Extracted data are in table below each chromatogram.

The fully protected L-Tryptophane appeared in a retention time of 25.3 min with an enantiomeric ratio of 89:11 to the D enantiomer.

We then moved to the evaluation of the arylated product **20**. Similarly, a 1 mg.mL^{-1} solution of both racemic and enantiopure **20** were analyzed. Results can be seen in the figure below.

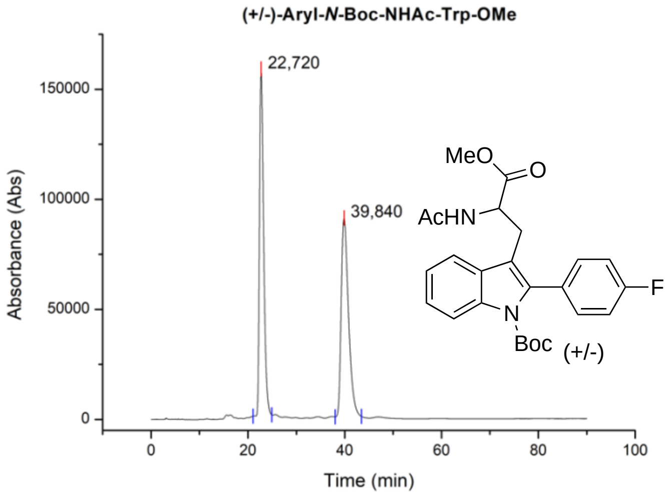

#	Retention Time (min)	Area	Relative Area (%)	*e.r.*
1	22.720	164122.7435	49.9%	
2	39.840	164507.9801	50.1%	1 : 1

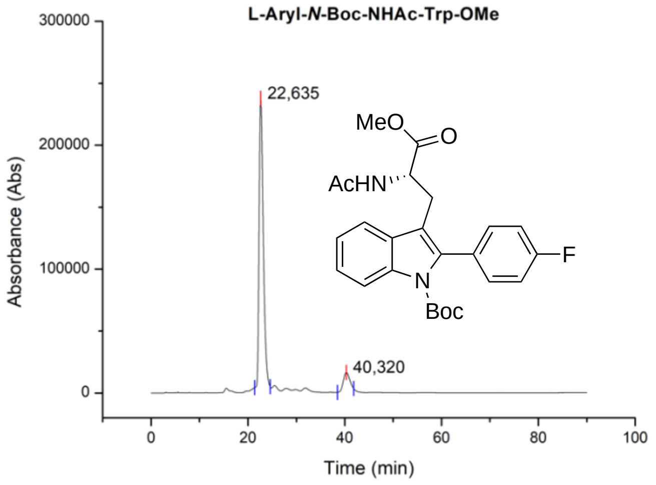

#	Retention Time (min)	Area	Relative Area (%)	*e.r.*
1	22.635	249344.2354	89.8%	
2	40.320	28251.33367	10.2%	90 : 10

Figure S15: HPLC traces of racemic (top) and enantipure (bottom) arylated Tryptophan **20**. Extracted data are in table below each chromatogram.

The results clearly indicate that Tryptophan is not racemized during reaction, since product and starting material enantiomeric ratios are identical.

6. Amino acid competition experiment

We conducted reactions between aryl diazonium salt 2 and each of the following amino acids: Ac-Phe-OMe, Ac-His-OMe, Ac-Tyr-OMe, Ac-Pro-OMe, Ac-Ser-OMe, and Ac-Cys-OMe. Subsequently, reactions using 2 were carried out with equimolar mixtures of Ac-Trp(Boc)-OMe and each of the aforementioned amino acids, individually. The results of each reaction were analyzed by ^{19}F NMR spectroscopy.

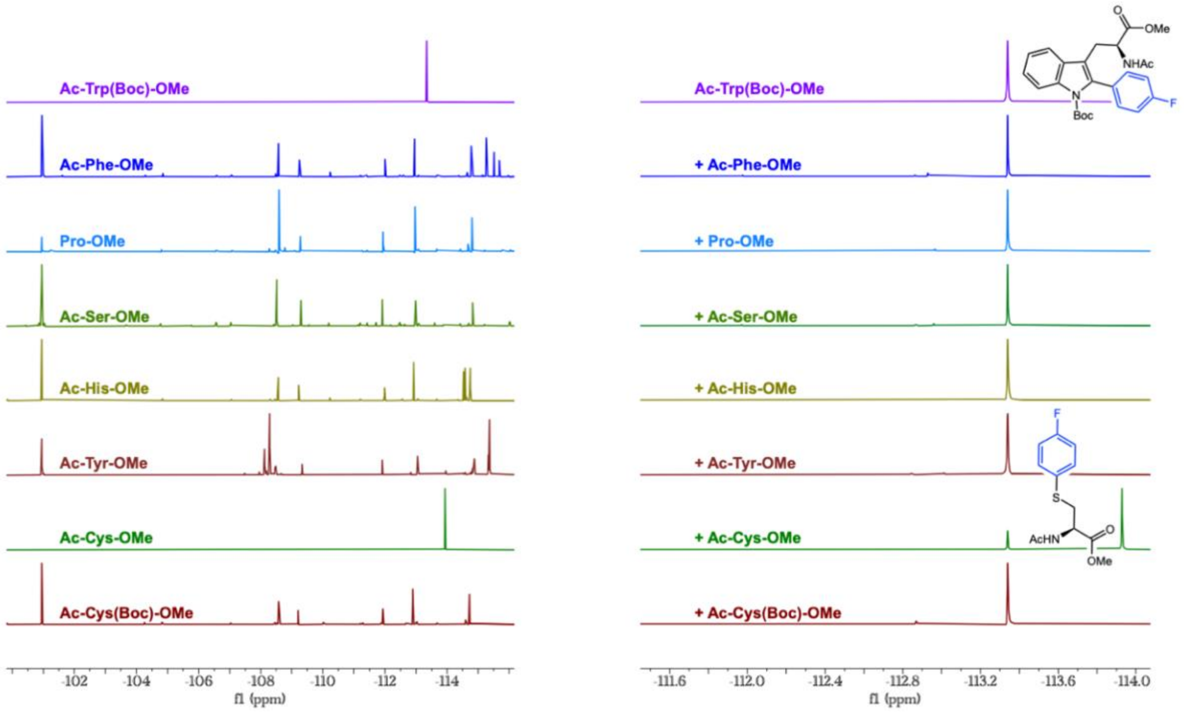

Figure S16: ^{19}F NMR spectra of crude reaction mixture. Individual reactivity for each amino acid (left). Amino acid competition experiment (rigth).

7. Peptide Substrates reaction and characterization

7.1. Model peptide 64 synthesis

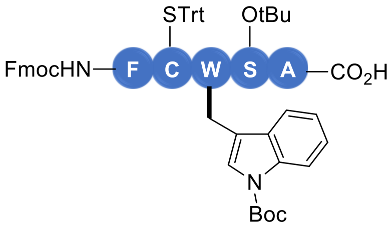

Automated peptide synthesis was carried out on a CEM Liberty Automated Microwave Peptide Synthesiser using H-L-Ala-2CT Resin (100-200 mesh, 0.59 mmol/g, 1% DVB). The scale for the resin-bound peptide reaction was 0.25 mmol. The automatic peptide coupling was performed with Fmoc-protected amino acids (0.2 M), Oxyma pure (1 M) and DIC (1 M) as coupling reagents in DMF. The following resin and amino acids amounts were employed: H-L-Ala-2CT Resin (0.423 mg in 20 mL of DMF), Fmoc-Ser(*t*-Bu)-OH (0.85 g in 11 mL of DMF), Fmoc-Trp(Boc)-OH (1.16 g in 11 mL of DMF), Fmoc-Cys(Trt)-OH (1.29 g in 11 mL of DMF), Fmoc-Phe-OH (0.85 g in 11 mL of DMF). Peptide was coupled at 23 °C for 2 hours (0 W), and 2×2 hours cycle was used for each subsequent amino acid. After the automatic peptide synthesis, the peptide was transferred to a plastic syringe equipped with a porous disk, washed with CH_2Cl_2 (5×10 mL), DMF (2×10 mL), CH_2Cl_2 again (2×10 mL), and stored dried in a freezer. Cleavage of the peptide from the resin was performed prior to HPLC purification by shaking with CH_2Cl_2/HFIP (2:1, 20 mL, v/v) for 4 h. The crude material was evaporated under a stream of nitrogen and dissolved in 3 mL of DMSO.

Purification was conducted in preparative HPLC eluting with a linear gradient system with mobile phases (A) 0.1% TFA in H_2O (v/v) and (B) 0.05% TFA in MeCN (v/v) over 23 min at a flow rate of 20 mL.min^{-1}. Preparative HPLC was monitored by UV absorbance at 299 nm. After purification, the desired peptide was obtained in 94% yield (290 mg, 0.235 mmol).

Analytical HPLC of pure peptide was carried out using an Agilent 1260 Infinity system with a reversed-phase Supelcosil™ ABZ+PLUS column (150 mm × 4.6 mm, 3 µm) eluting with a linear gradient system (solvent A: 0.05% (v/v) TFA in H_2O, solvent B: 0.05% (v/v) TFA in MeCN) over 15 minutes, at a flow rate of 1 mL/min. Analytical HPLC was monitored by UV absorbance at 220 and 254 nm.

UV-LCMS analysis was carried out using Waters ACQUITY H-Class UPLC with an ESCi Multi-Mode Ionization Waters SQ Detector 2 spectrometer using ACQUITY UPLC® CSH C18 column (2.1 mm × 50 mm, 1.7 µm, 130 Å) at 40 °C and flow rate 0.6 mL/min. Solvent A: 2 mM NH_4OAc in H_2O/MeCN (95:5), solvent B: MeCN, and solvent C: 2% formic acid. Gradient: 5-95% B with constant 5% C over 3 min. PDA eλ Detector 220-800 nm, interval 1.2 nm.

HPLC (ABZ+Plus, Water/MeCN (0.05% TFA) = 50/50 – 0/100 over 15 minutes, flow rate = 1 mL/min, λ = 254 nm) tR = 14.08 min.

HPLC (ABZ+Plus, Water/MeCN (0.05% TFA) = 30/70 – 0/100 over 15 minutes, flow rate = 1mL/min, λ = 254 nm) tR = 8.27 min.

LC-MS (CSH C18, 5% Water in MeCN (2mM NH_4OAc / MeCN / 2% Formic acid in water = 95 / 5 / 5 – 5 / 95 / 5 over 3 minutes, flow rate = 0.6 mL/min, λ = 299 nm, 40 ºC) tR = 3.56 min. MS (ESCi) m/z $[M-H]^-$ calcd for $C_{72}H_{76}N_6O_{11}S$ 1232.5; Found 1232.5.

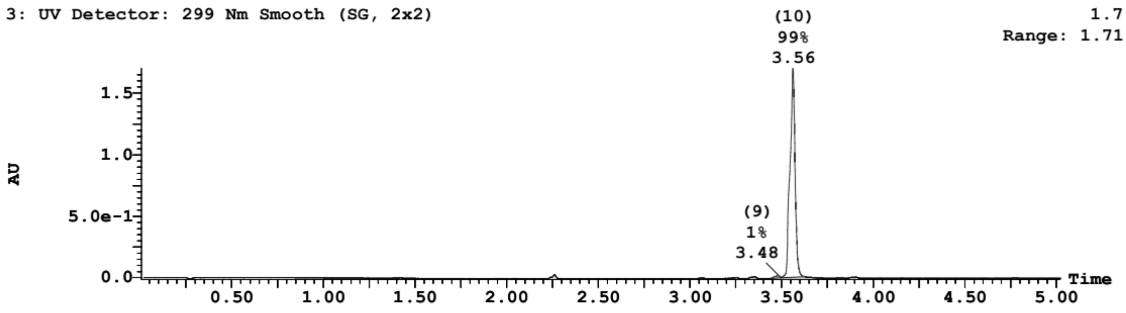

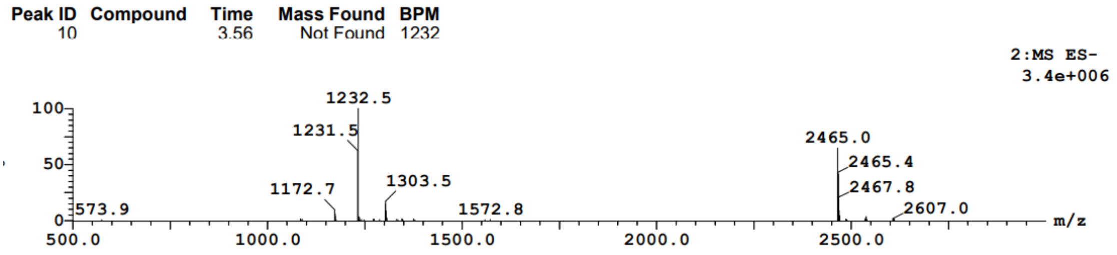

Figure S17: LC-MS analysis of model peptide **64**.(Top) 299 nm chromatogram. (Bottom) Mass spectrum of the corresponding peak.

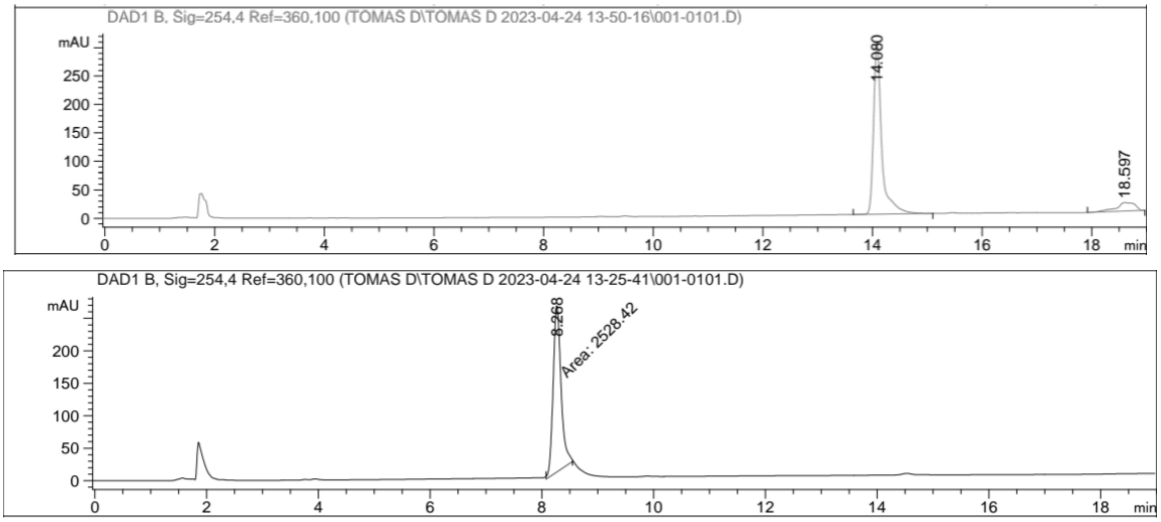

Figure S18: 254 nm chromatogram of the HPLC analysis of model peptide **64**.(Top) Gradient 50-100%B over 15min. (Bottom) Gradient 70-100% B over 15 min.

7.2. Model peptide arylation reaction optimization

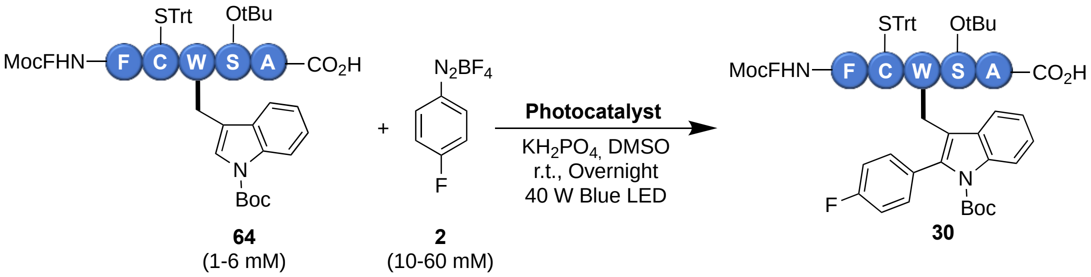

Scheme S8: Reaction optimization for model peptide **64**.

General conditions: A 2 mL glass vial containing a magnetic stirring bar was charged with the solid 4-fluorobenzenediazonium tetrafluoroborate (10 equiv.), a solution with 10 mM of peptide **64** in DMSO (1 equiv.), a solution with 1 mM of Eosin Y disodium salt in DMSO (0-0.1 equiv.) and a solution with 0.12 M of KH_2PO_4 in H_2O (2 equiv.) and DMSO to achieve the desired final concentration. The scale for the peptide arylation reaction was 0.5-1.5 μmol. The vial was then sealed with parafilm, purged with nitrogen for 15 minutes in the dark, and kept under nitrogen atmosphere. The homogeneous reaction mixture was irradiated with a Blue LED (450-460 nm) for 12 h. The vial was then opened to air, a 50 μL aliquot of the reaction mixture was diluted in 1 mL of H_2O/MeCN (1:1) and analyzed by UV-LCMS.

UV-LCMS analysis was carried out using Waters ACQUITY H-Class UPLC with an ESCi Multi-Mode Ionisation Waters SQ Detector 2 spectrometer using ACQUITY UPLC® CSH C18 column (2.1 mm × 50 mm, 1.7 μm, 130 Å) at 40°C and flow rate 0.6 mL/min.

Solvent A: 2 mM NH_4OAc in H_2O/MeCN (95:5), solvent B: MeCN, and solvent C: 2% formic acid. Gradient: 5-95% B with constant 5% C over 3 min. PDA eλ Detector 220-800 nm, interval 1.2 nm.

Conversion was estimated based on the TIC chromatogram of the limiting starting material. Conditions applied and resulting conversion are summarized in the table below.

Table S3: Reaction conditions optimization for peptide **64** arylation

Entry	ArN_2BF_4	Na_2Y	Blue LED Power	Peptide Concentration	Conversion
1	10 mM	10 mol%	40 W	1 mM	0%
2	30 mM	10 mol%	40 W	3 mM	30%
3	60 mM	10 mol%	40 W	3 mM	100%
4	60 mM	10 mol%	40 W	6 mM	100%
5	60 mM	10 mol%	10 W	6 mM	0%
6	60 mM	w/o catalyst	40 W	6 mM	100%
7	60 mM	w/o catalyst	40 W	3 mM	100%
8	60 mM	w/o catalyst	10 W	6 mM	0%

7.3. Scope of Aryl Diazonium Salts for the arylation of model peptide 64

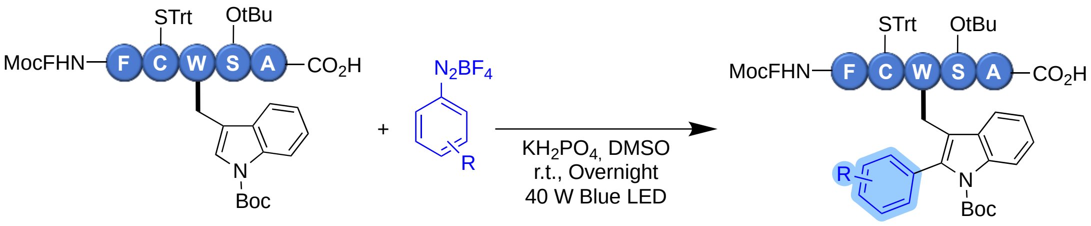

64 (6 mM) (60 mM)

Scheme S9: Scope of Aryl diazonium salts for arylation of model peptide **64**.

General conditions A 2 mL glass vial containing a magnetic stirring bar was charged with the solid arenediazonium tetrafluoroborate salts (60 μmol, 10 equiv.), a solution 0.01 M of peptide **64** in DMSO (300 μL, 1 equiv.), and a solution 0.12 M of KH_2PO_4 in H_2O (50 μL, 2 equiv.) and 150 μL DMSO to achieve the final concentration of 6 mM. The vial was then sealed with parafilm, purged with nitrogen for 15 minutes in the dark, and kept under nitrogen atmosphere. The homogeneous reaction mixture was irradiated with a 40 W Blue LED (450-460 nm) for 12 h. The vial was then opened to air, a 50 μL aliquot of the reaction mixture was diluted in 1 mL of H_2O/MeCN (1:1) and analyzed by UV-LCMS.

UV-LCMS analysis was carried out using Waters ACQUITY H-Class UPLC with an ESCi Multi-Mode Ionization Waters SQ Detector 2 spectrometer using ACQUITY UPLC® CSH C18 column (2.1 mm × 50 mm, 1.7 μm, 130 Å) at 40 °C and flow rate 0.6 mL/min. Solvent A: 2 mM NH_4OAc in H_2O/MeCN (95:5), solvent B: MeCN, and solvent C: 2% formic acid. Gradient: 5-95% B with constant 5% C over 3 min. PDA eλ Detector 220-800 nm, interval 1.2 nm.

Purification was conducted in preparative HPLC eluting with a linear gradient system with mobile phases (A) 0.1% TFA in H_2O (v/v) and (B) 0.05% TFA in MeCN (v/v) over 23 min at a flow rate of 20 $mL.min^{-1}$. Preparative HPLC was monitored by UV absorbance at 299 nm.

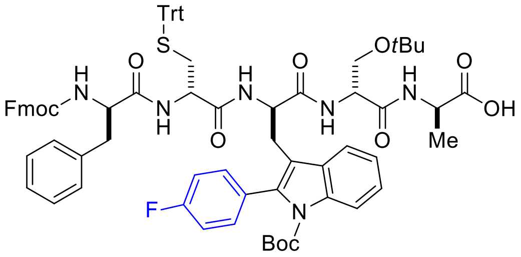

Product 30: Obtained using the general conditions outlined above using aryl diazonium salt **2**, with 55% isolated yield.

HPLC (ABZ+Plus, Water/MeCN (0.05% TFA) = 50/50 – 0/100 over 15 minutes, flow rate = 1 mL/min, λ = 254 nm) tR = 15.21 min.

HPLC (ABZ+Plus, Water/MeCN (0.05% TFA) = 30/70 – 0/100 over 15 minutes, flow rate = 1mL/min, λ = 254 nm) tR = 9.61 min.

LC-MS (CSH C18, 5% Water in MeCN (2mM NH_4OAc / MeCN / 2% Formic acid in water = 95 / 5 / 5 – 5 / 95 / 5 over 3 minutes, flow rate = 0.6 mL/min, λ = 299 nm, 40 ºC) tR = 3.77 min. MS (ESCi) m/z $[M-H]^-$ calcd for $C_{78}H_{79}FN_6O_{11}S$ m/z 1326.5; Found 1326.3.

^{19}F NMR (376 MHz, DMSO-d6): δ -73.42 (s)

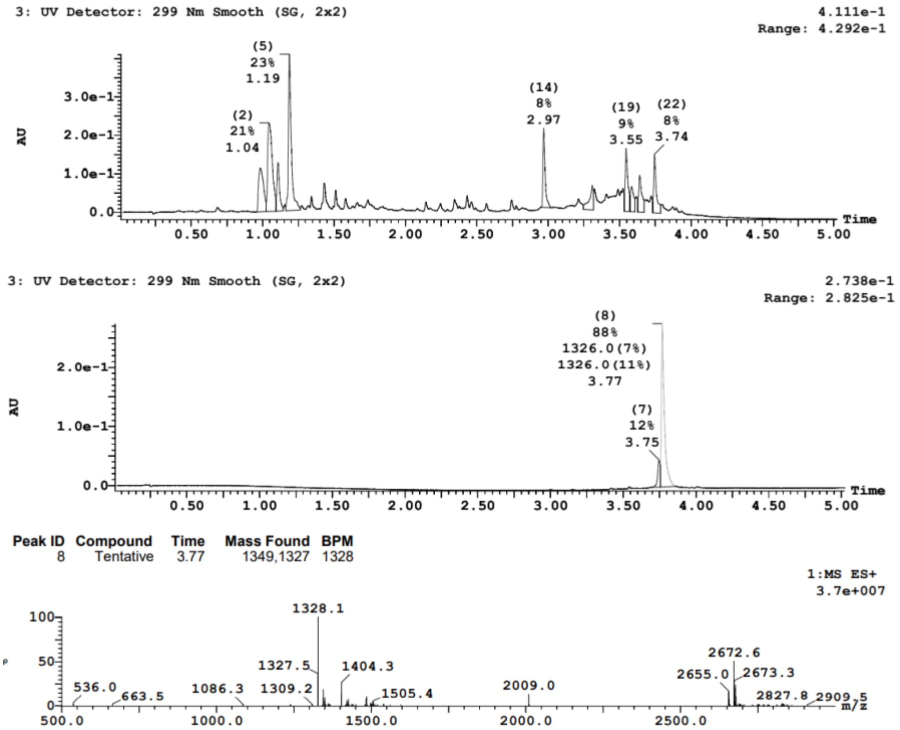

Figure S19: LC-MS analysis of **30**. (Top) 299 nm chromatogram of crude reaction mixture. (Middle) 299 nm chromatogram of purified product. (Bottom) Mass spectrum of the corresponding peak.

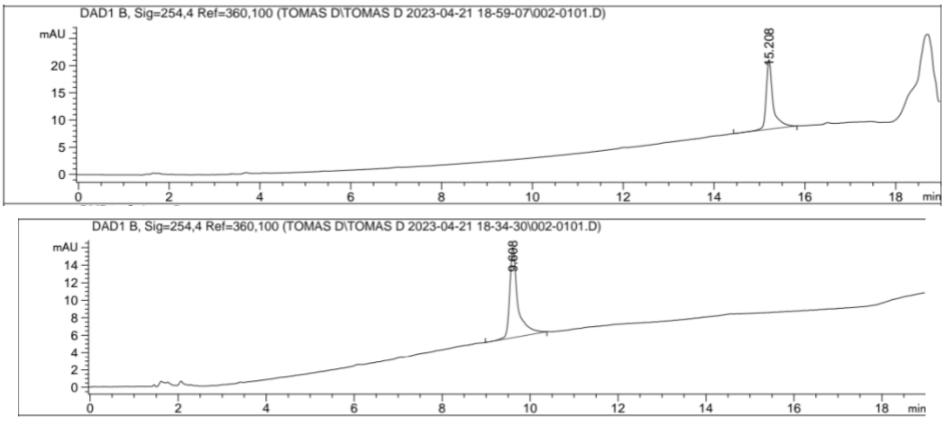

Figure S20: 254 nm chromatogram of the HPLC analysis of **30**. (Top) Gradient 50-100%B over 15min. (Bottom) Gradient 70-100% B over 15 min.

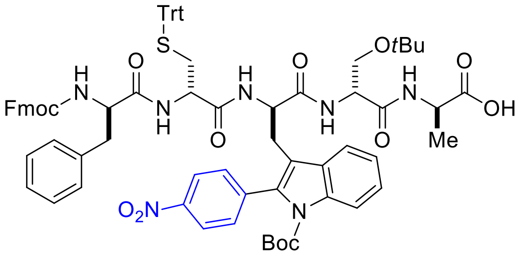

Product 31: Obtained using the general conditions outlined above using aryl diazonium salt **45**, with 79% isolated yield.

HPLC (ABZ+Plus, Water/MeCN (0.05% TFA) = 30/70 – 0/100 over 15 minutes, flow rate = 1 mL/min, λ = 254 nm) tR = 8.86 min.

HPLC (ABZ+Plus, Water/MeCN (0.05% TFA) = 20/80 – 0/100 over 15 minutes, flow rate = 1mL/min, λ = 254 nm) tR = 7.21 min.

LC-MS (CSH C18, 5% Water in MeCN (2mM NH_4OAc / MeCN / 2% Formic acid in water = 95 / 5 / 5 – 5 / 95 / 5 over 3 minutes, flow rate = 0.6 mL/min, λ = 299 nm, 40 ºC) tR = 3.72 min. MS (ESCi) m/z $[M-H]^-$ calcd for $C_{78}H_{79}N_7O_{13}S$ *m/z* 1353.5; Found 1353.6.

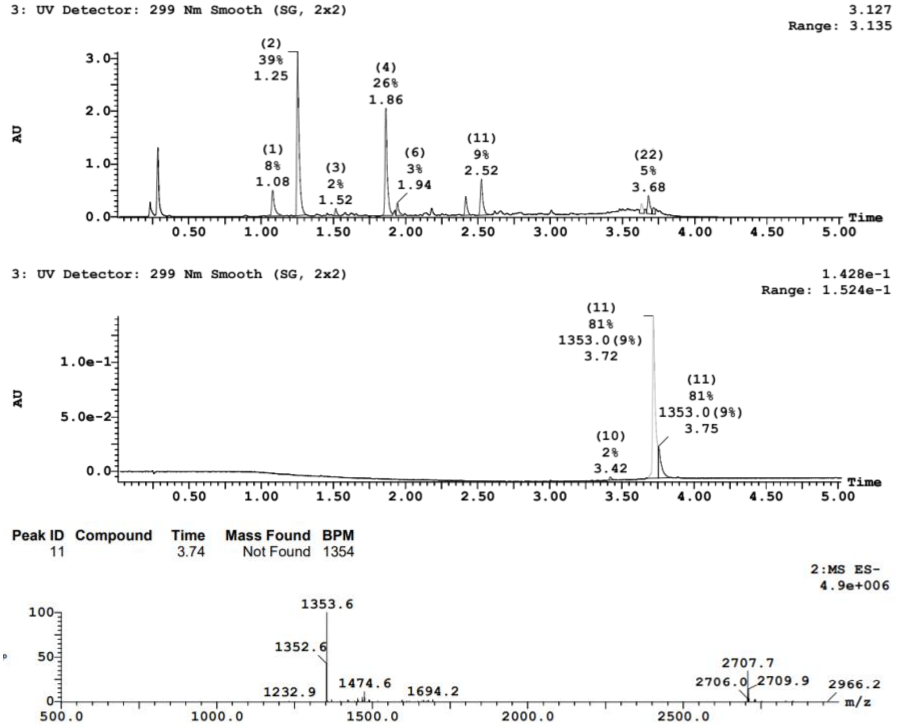

Figure S21: LC-MS analysis of **31**. (Top) 299 nm chromatogram of crude reaction mixture. (Middle) 299 nm chromatogram of purified product. (Bottom) Mass spectrum of the corresponding peak.

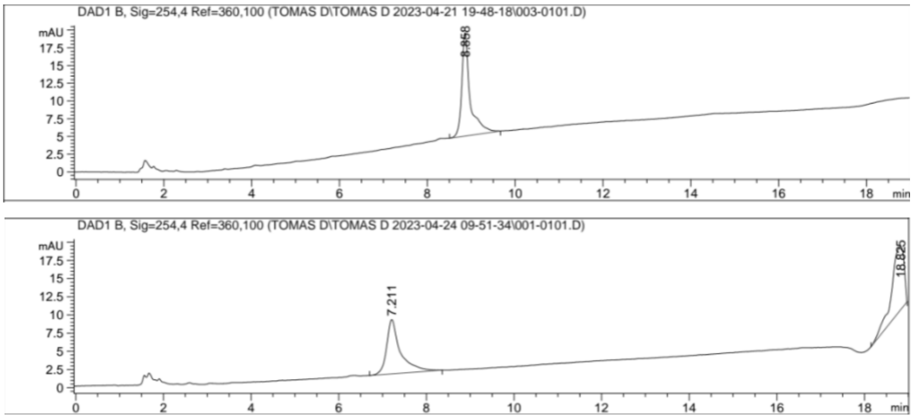

Figure S22: 254 nm chromatogram of the HPLC analysis of **31**. (Top) Gradient 70-100%B over 15min. (Bottom) Gradient 80-100% B over 15 min.

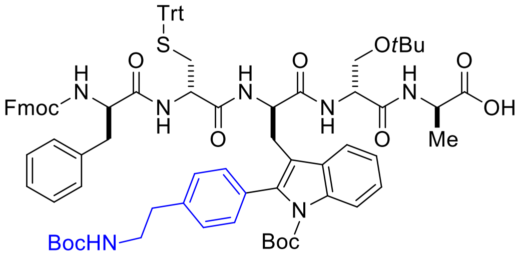

Product 32: Obtained using the general conditions outlined above using aryl diazonium salt **59**, with 23% isolated yield.

HPLC (ABZ+Plus, Water/MeCN (0.05% TFA) = 50/50 – 0/100 over 15 minutes, flow rate = 1 mL/min, λ = 254 nm) tR = 16.03 min.

HPLC (ABZ+Plus, Water/MeCN (0.05% TFA) = 30/70 – 0/100 over 15 minutes, flow rate = 1mL/min, λ = 254 nm) tR = 10.67 min.

LC-MS (CSH C18, 5% Water in MeCN (2mM NH_4OAc / MeCN / 2% Formic acid in water = 95 / 5 / 5 – 5 / 95 / 5 over 3 minutes, flow rate = 0.6 mL/min, λ = 299 nm, 40 ºC) tR = 3.87 min. MS (ESCi) m/z $[M-H]^-$ calcd for $C_{85}H_{93}N_7O_{13}S$ *m/z* 1451.6; Found 1451.3.

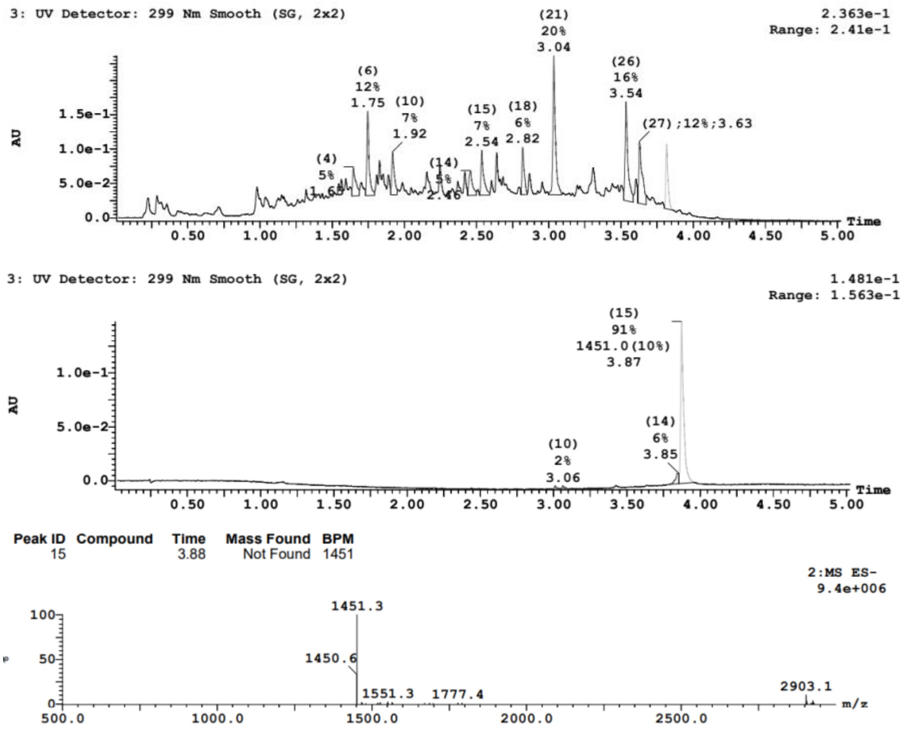

Figure S23: LC-MS analysis of **32**. (Top) 299 nm chromatogram of crude reaction mixture. (Middle) 299 nm chromatogram of purified product. (Bottom) Mass spectrum of the corresponding peak.

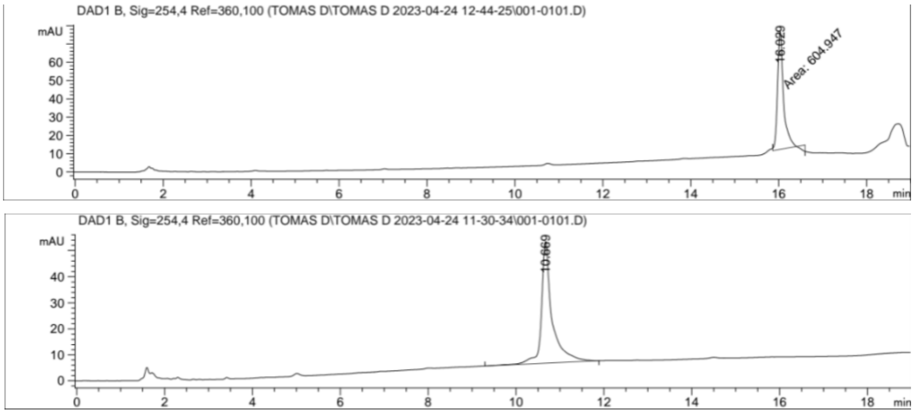

Figure S24: 254 nm chromatogram of the HPLC analysis of **32**. (Top) Gradient 50-100%B over 15min. (Bottom) Gradient 70-100% B over 15 min.

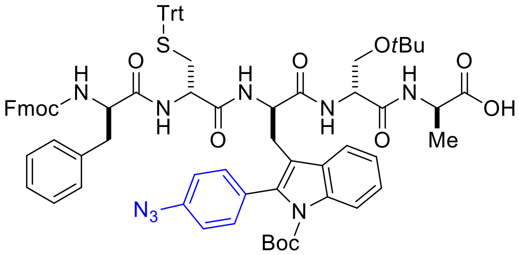

Product 33: Obtained using the general conditions outlined above using aryl diazonium salt **57**, with 36% isolated yield.

HPLC (ABZ+Plus, Water/MeCN (0.05% TFA) = 50/50 – 0/100 over 15 minutes, flow rate = 1 mL/min, λ = 254 nm) tR = 15.66 min.

HPLC (ABZ+Plus, Water/MeCN (0.05% TFA) = 30/70 – 0/100 over 15 minutes, flow rate = 1mL/min, λ = 254 nm) tR = 9.45 min.

LC-MS (CSH C18, 5% Water in MeCN (2mM NH_4OAc / MeCN / 2% Formic acid in water = 95 / 5 / 5 – 5 / 95 / 5 over 3 minutes, flow rate = 0.6 mL/min, λ = 299 nm, 40 ºC) tR = 3.83 min. MS (ESCi) m/z $[M-H]^-$ calcd for $C_{78}H_{79}N_9O_{11}S$ *m/z* 1349.5; Found 1349.3.

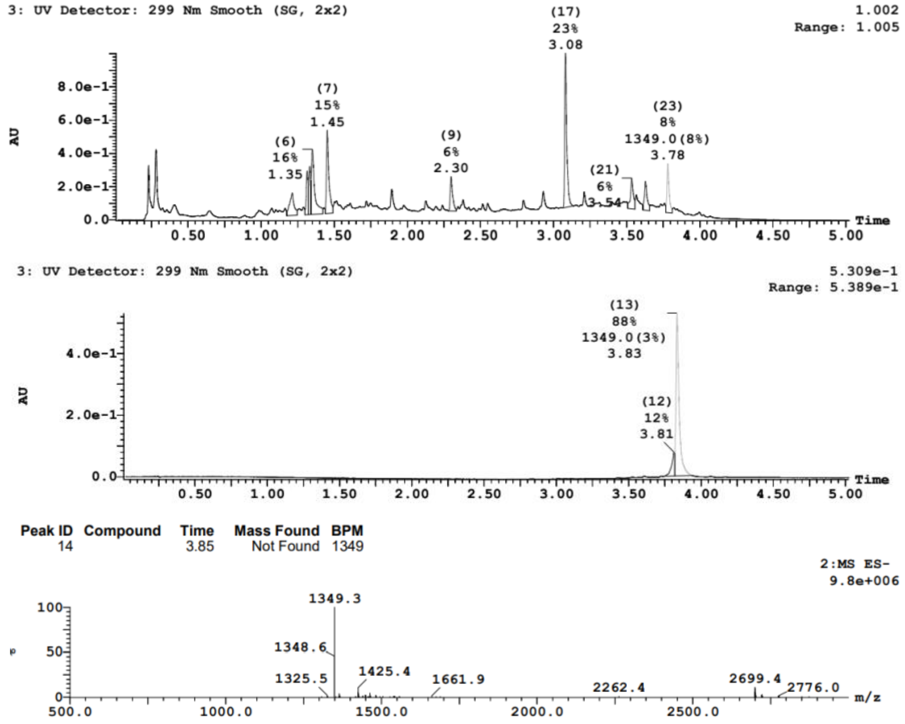

Figure S25: LC-MS analysis of **33**. (Top) 299 nm chromatogram of crude reaction mixture. (Middle) 299 nm chromatogram of purified product. (Bottom) Mass spectrum of the corresponding peak.

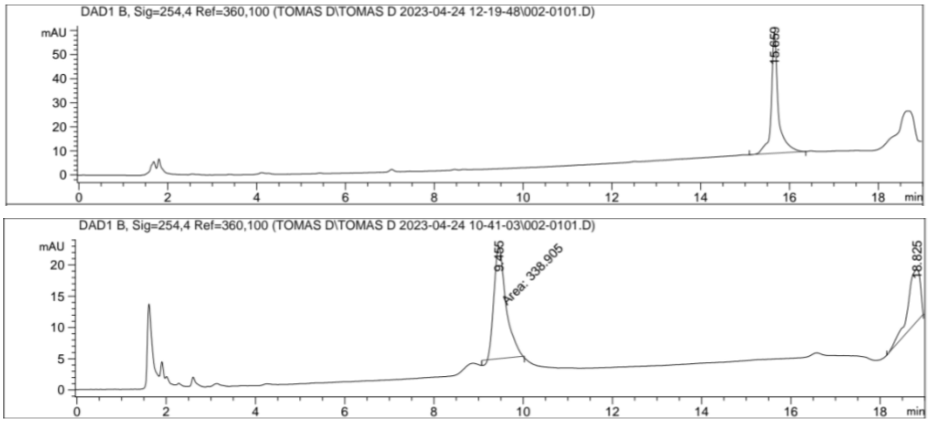

Figure S26: 254 nm chromatogram of the HPLC analysis of **33**. (Top) Gradient 50-100% B over 15min. (Bottom) Gradient 80-100% B over 15 min.

7.4. Arylation of Octreotide

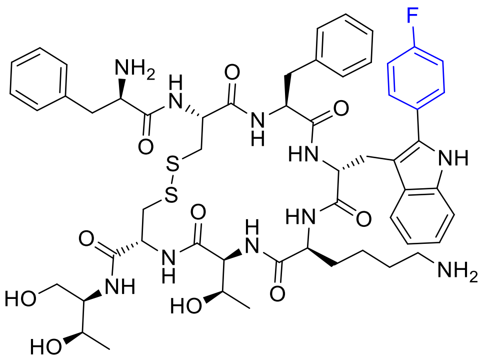

Product 34: A 1 mL glass vial containing a magnetic stirring bar was charged with a solution 3 mM of Octreotide acetate in DMSO (0.5 mL), and the solid 4-fluorobenzenediazonium tetrafluoroborate (6.2 mg, 30 μmol, 20 equiv., 60 mM final concentration). The vial was then sealed with parafilm, purged with argon for 15 minutes in the dark, and kept under argon atmosphere. The homogeneous reaction mixture was irradiated with a 10 W Blue LED (450-460 nm) for 3 h. The vial was then opened to air, the reaction mixture was diluted in 2.0 mL of H_2O submitted to subsequent analysis and isolation.

Product was isolated in 49% after purification through preparative HPLC using a Shimadzu Corp. Nexera 2 equipment with a Kromasil 100-10-C18 column (10x210 mm) eluting with a gradient system with mobile phases (A) 0.1% formic acid in H_2O (v/v) and (B) 0.1% formic acid in MeOH (v/v) over 35 min at a flow rate of 2 mL.min^{-1}. Gradient consisted of 4 minutes at 30% B, then 20 minutes with a linear increase until 95% B and then 8 minutes at 95% B. After that, the column was equilibrated with a 3 minutes linear decrease until the initial 30% B. Preparative HPLC was monitored by both UV absorbance at 295 nm and mass spectrometry using Bruker Daltonic's Amazon SL Ion Trap analyzer with ESI ionization in the positive mode.

Analytical HPLC was carried out using a Shimadzu Prominence LC-20A equipped with a PDA detector, using a BDS Hypersil C18 column (250 mm x 4.6 mm, 5 μm, 120 Å) at 40 ºC and flowrate of 1.0 mL/min. Solvent A: 0.1% formic acid in H2O (v/v), Solvent B: 0.1% formic acid in MeOH (v/v). Method: (I) 30% B Isocratic for 5 minutes, 30-65% B in a linear increase over the next 10 minutes, isocratic at 65% B for the next 10 minutes, 65-95% B in a linear increase for 1 minutes, then isocratic 95% B over the next 4 minutes. (II) 50% B isocratic for 5 minutes, then linear increase 50-95% for the next 10 minutes and isocratic 95% B over the next 5 minutes. The substances were detected spectrophotometrically at λ=295 nm.

The purified product was analyzed through HRMS using a Bruker Daltonic Solarix XR of 7 Tesla with an FT-ICR analyzer and an ESI source.

HPLC (Hypersil C18, Water/MeOH (0.1% Formic acid) = 70/30 (5 min.); 70/30 - 35/65 (10 min.); 35/65 - 5/95 (1 min.); 5/95 (4 min.), flow rate = 1 mL/min, λ = 295 nm, 40 ºC) tR = 17.0 min.

HPLC (Hypersil C18, Water/MeOH (0.1% Formic acid) = 50/50 (5 min.); 50/50 - 5/95 (10 min.); 5/95 (5 min.), flow rate = 1 mL/min, λ = 295 nm, 40 ºC) tR = 12.7 min.

HRMS (ESI) m/z: $[M+2H]^{2+}$ calcd for $C_{55}H_{69}FN_{10}O_{10}S_2$ 557.2390; Found 557.2382.

^{19}F NMR (470 MHz, D_2O) = δ -75.63.

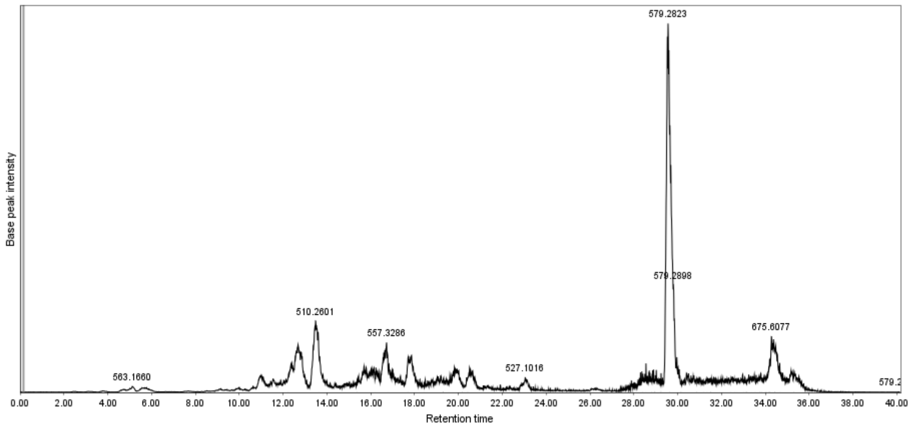

Figure S27: Preparative LC/MS analysis of crude reaction mixture. Extracted-ion chromatogram for base peaks with 500-1200 m/z.

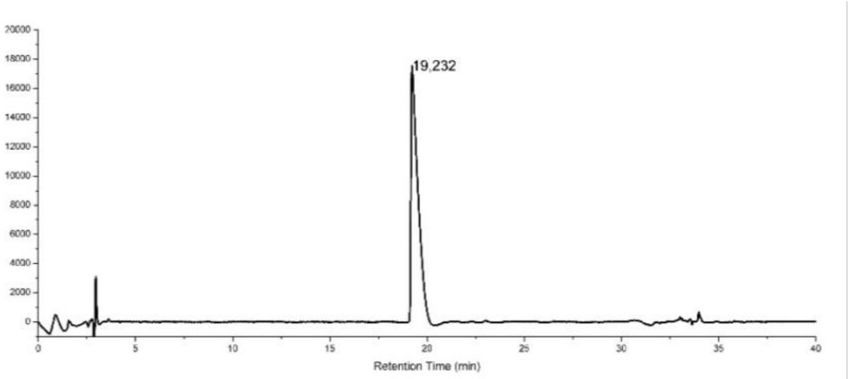

Figure S28: HPLC analysis (Method I) of Octreotide Acetate starting material.

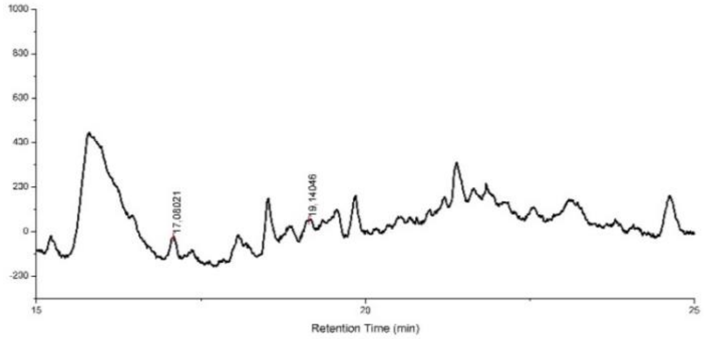

Figure S29: HPLC analysis (Method I) of crude reaction mixture.

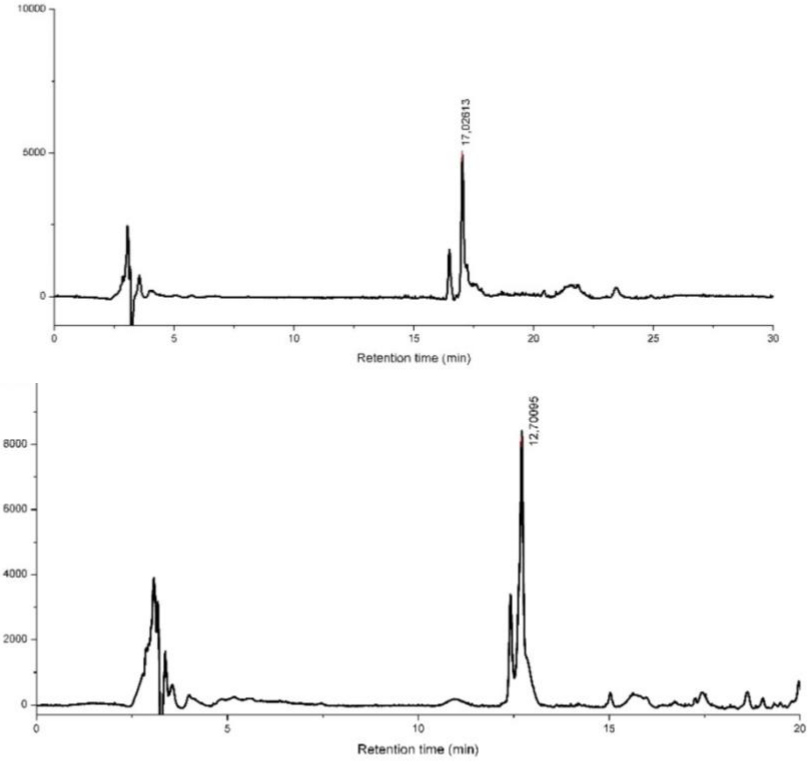

Figure S30: HPLC analysis of purified product **34**. (Top) Method I. (Bottom) Method II.

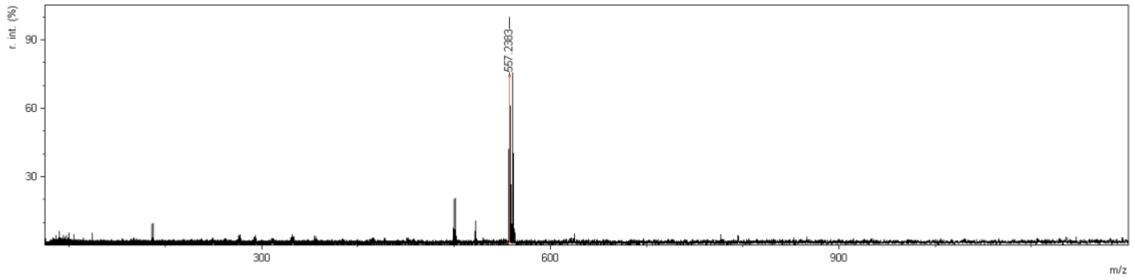

Figure S31: High-Resolution MS[1] spectra of product **34**.

8. Enzyme substrate reaction and characterization

8.1. Lysozyme arylation protocol

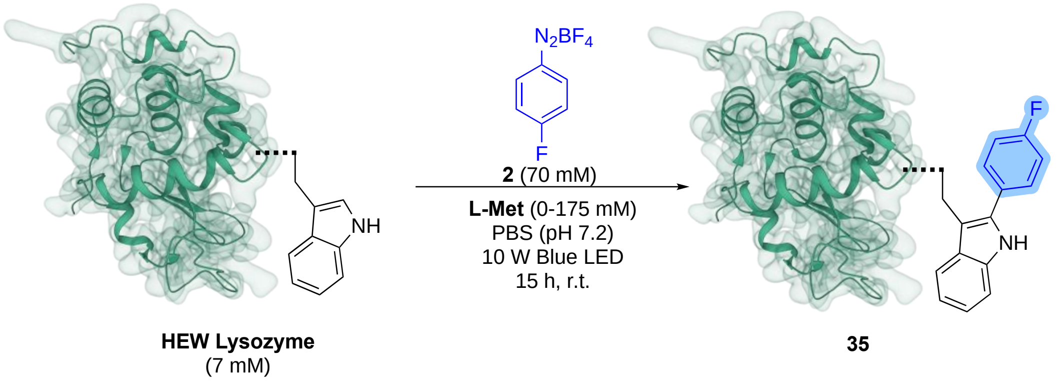

Scheme S10: Lyzosyme arylation.

Without Methionine addition: A 1 mL glass vial containing a magnetic stirring bar was charged with HEW lysozyme (50 mg, 14 kDa, 3.5 μmol) and freshly prepared phosphate buffer saline (0.5 mL, pH 7.2). After complete homogenization, 4-fluorobenzenediazonium salt (7.0 mg, 35 μmol, 10 equiv.) was added. The vial was then sealed with parafilm, purged with argon for 15 minutes in the dark, and kept under argon atmosphere. The homogeneous reaction mixture was irradiated with a 10 W Blue LED (450-460 nm) for 15 h. The vial was then opened to air, the reaction mixture was diluted in 2.0 mL of H_2O and centrifuged to decant solid particulates. The supernatant was eluted through a PD-10 desalting column packed with Sephadex™ G-25 resin using mili-Q grade water to separate the proteins from small reaction components. Fractions of 1 mL were collected and submitted to further analysis.

With Methionine addition: A 1 mL glass vial containing a magnetic stirring bar was charged with HEW lysozyme (50 mg, 14 kDa, 3.5 μmol), L-Methionine (13 mg, 88 μmol, 25 equiv.) and freshly prepared phosphate buffer saline (0.5 mL, pH 7.2). After complete homogenization, 4-fluorobenzenediazonium salt (7.0 mg, 35 μmol, 10 equiv.) was added. The vial was then sealed with parafilm, purged with argon for 15 minutes in the dark, and kept under argon atmosphere. The homogeneous reaction mixture was irradiated with a 10 W Blue LED (450-460 nm) for 15 h. The vial was then opened to air, the reaction mixture was diluted in 2.0 mL of H_2O and centrifuged to decant solid particulates. The supernatant was eluted through a PD-10 desalting column packed with Sephadex™ G-25 resin using mili-Q grade water to separate the proteins from small reaction components. Fractions of 1 mL were collected and submitted to further analysis.

8.2. Product characterization – Without Methionine addition

A ^{19}F NMR analysis (470 MHz, D_2O) was performed to detect arylation. It was possible to detect signals at -122 ppm that are compatible with indole arylated structures. The signals in -150 ppm refer to BF_4^- anions that were probably complexed to the protein structure.

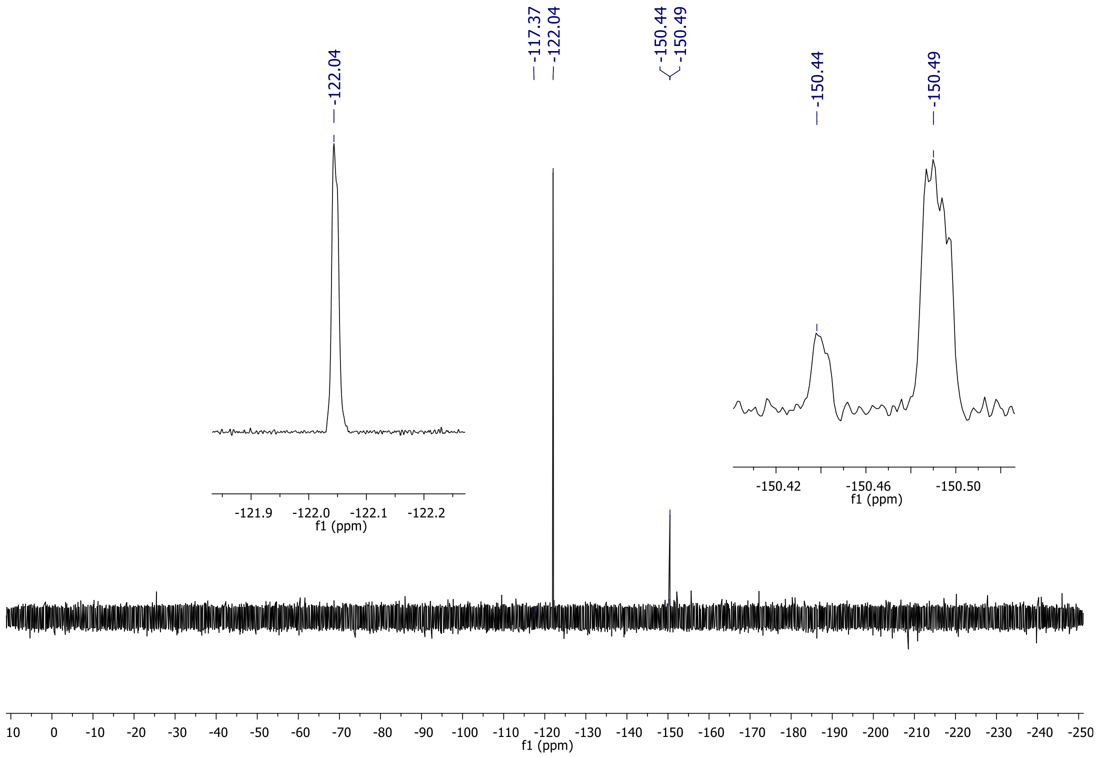

Figure S32: ^{19}F NMR of desalted reaction mixture containing fluorine signals comptible with Tryptophan fluorination.

These samples were further purified using ZipTip protocol before they were submitted to intact protein mass spectrometry analysis using a Bruker Impact HD mass spectrometer equipped with an ESI font and Q-TOF analyzer.

Mass spectrum of the unreacted protein was obtained in the positive mode, showing the expected peak distribution due to different charge states, and indicating an average mass of 14305 Da for the protein.

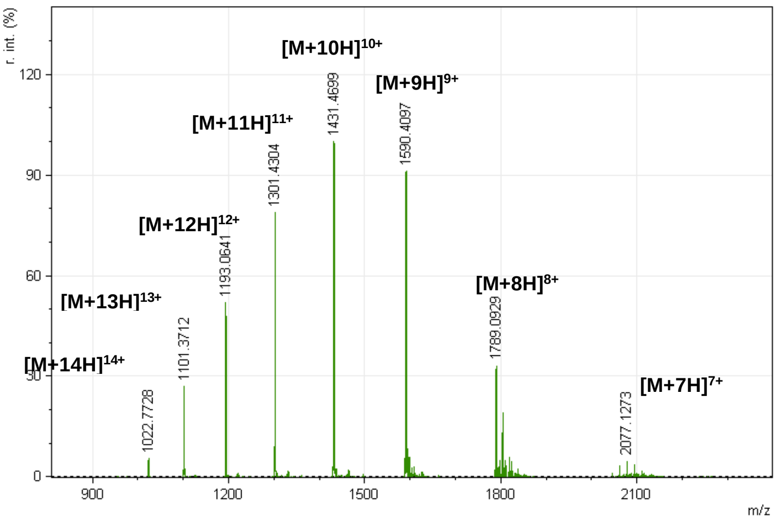

Figure S33: Unreacted Lysozyme intact mass spectrum. Positive mode.

The intact mass analysis of the samples after reaction are shown in the figures below.

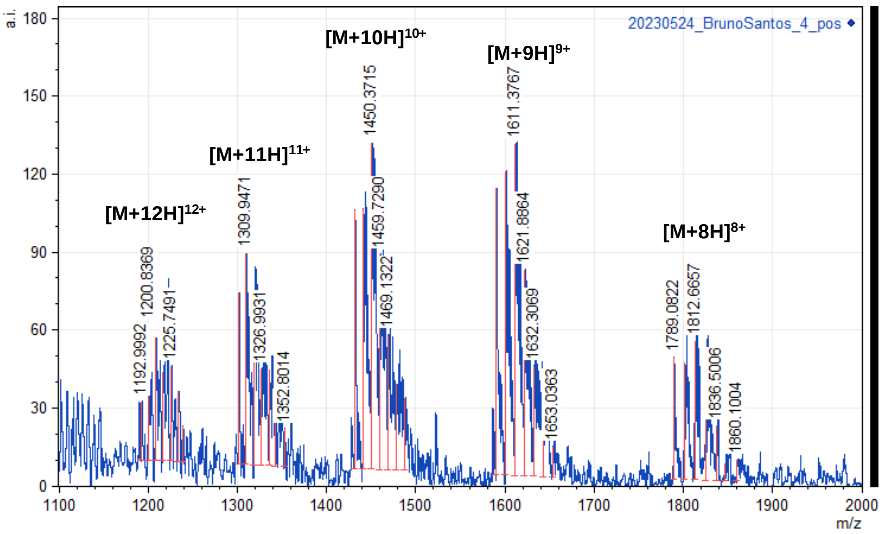

Figure S34: Intact mass spectrum of protein samples after reaction. Positive mode.

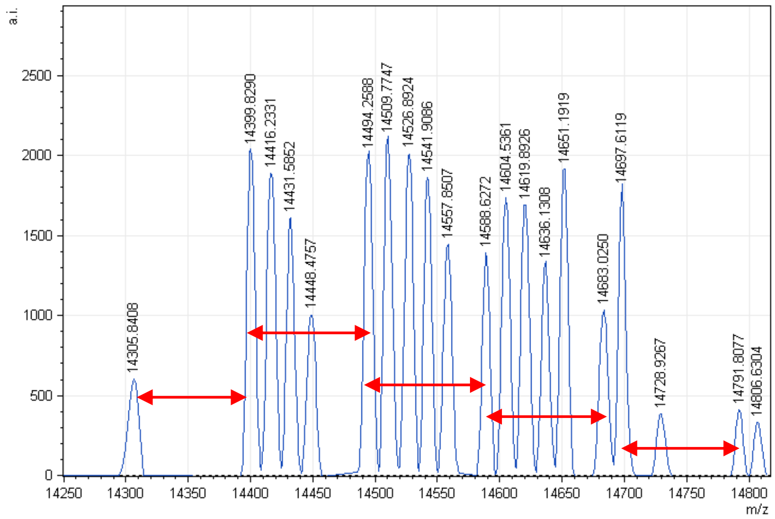

Figure S35: Deconvoluted mass spectra. The 94 Da differences in mass that indicate arylation are marked with red arrows.

The intact protein analysis indicate that the reaction led to a mixture with different levels of arylated proteins. The 94 Da differences in mass that are highlighted in Figure S35 refer to the substitution of a hydrogen atom by a 4-fluorophenyl radical. We could achieve the arylation of up to five amino acid residues in a single protein, but there was still some unreacted Lysozyme in the final reaction mixture. We could also detect several 15 Da differences in mass, indicating the oxidation of the Sulphur-containing residues in the protein.

The characterization of the arylation sites was performed by peptide mapping strategy using tandem LC-MS/MS spectrometry. A standard trypsin digest protocol was employed after cystine-bridge reduction using DTT and consecutive carbamidomethylation of Cysteine residues. The results were interpreted in Bruker Daltonics BioTools, using the Lysozyme sequence (based on UniProt P00698 entry) below. They show a good sequence coverage (81%) at the MS^1 level, including all Tryptophan residues. Tryptic peptides used to confirm arylation sites are highlighted in the following sequence and their MS^2 analysis was performed using Mmass software. Predicted b and y ions are tabulated for each peptide, with the observed ions highlighted in red. A full list of the matched ions is provided immediately after. The arylation of the Tryptophan residue (W-ArF) causes an increment of +94.0219 Da in monoisotopic mass. Carbamidomethylated cysteine (C-CAM) from sample preparation for trypsin digestion causes an increment of +57.0215 Da. Methionine oxidation causes an increment of +15.9949 Da.

We could detect arylation in Trp46, Trp126, Trp129 and Trp141 residues. Only one residue among Trp80-81 is arylated, without any observable preference or selectivity. We could not detect any tryptic peptide that indicated arylation of both units.

MRSLLILVLC FLPLAALGKV FGRCELAAAM KRHGLDNYR**G YSLGNWVCAA K**FESNFNTQA TNRNTDGSTD

YGILQINSR**W WCNDGR**TPGS RNLCNIPCSA LLSSDITASV NCAKK**IVSDG NGMNAWVAWR NR**CK**GTDVQA**

WIRGCRL

Figure S36: Lysozyme sequence (Uniprot ID P00698). Tryptic peptides used to confirm arylation sites are highlighted.

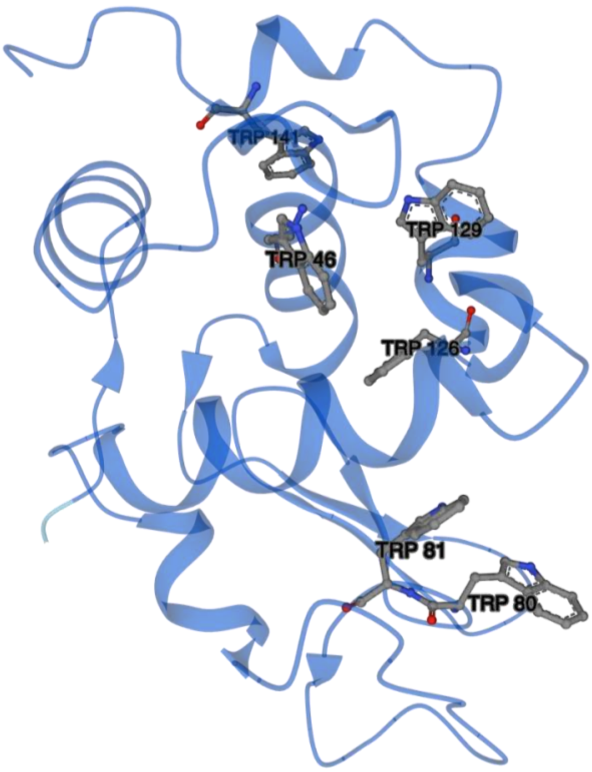

Figure S37: Lysozyme structure, evidencing Tryptophan residues. Image generated bt AlphaFold Protein Structure Database, based on informations contained in UniProt Database.

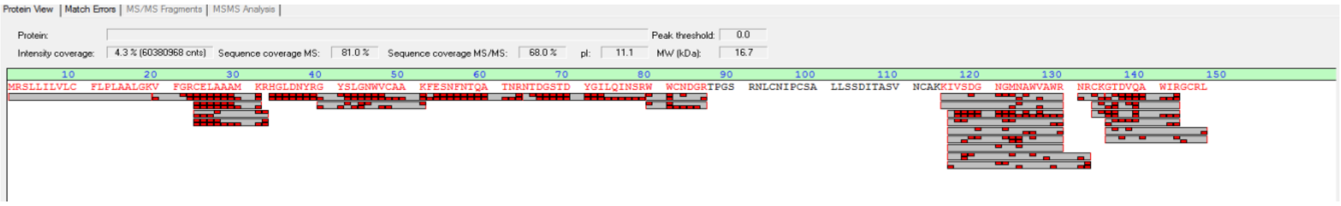

Figure S38: Bruker Daltonic BioTools output from the LC-MS/MS analysis of protein digest, showing sequence coverage of 81% for the protein at the MS level (All Tryptophan residues included).

Peptide GTDVQAW[141]IRGCRL [Sequence range: 135-147]

The MS^2 data was obtained from the tryptic peptide selecting m/z 813.3283 for collision-induced dissociation with 40.7 eV of collision energy. The table with some of the predicted fragments is listed, and the main matching parameters are highlighted in red. A detailed table of matching parameters is also provided.

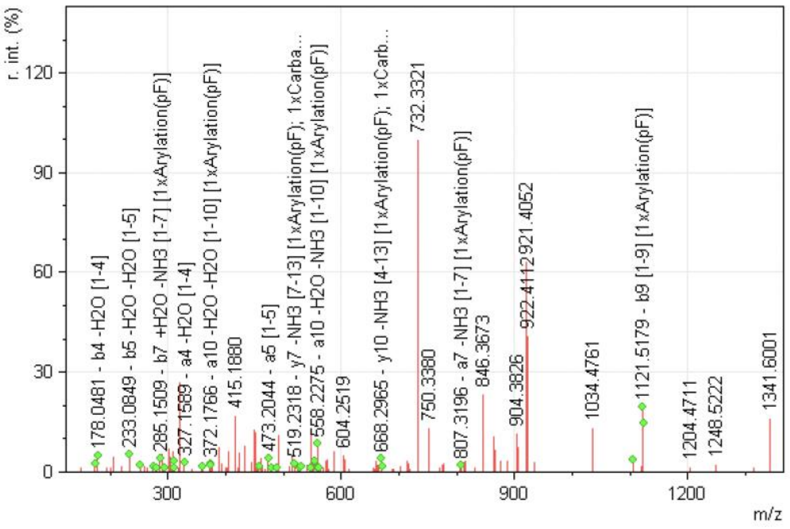

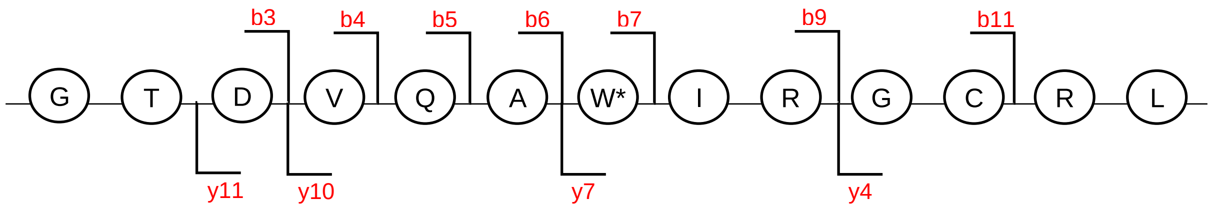

Predicted b and y ions

(Matched ions highlighted in red. Predicted values)

b^{3+}	b^{2+}	b^{+}		Sequence		y^{+}	y^{2+}	y^{3+}
		58.0288	1	**G**	13	1625.8018		
53.697	80,0418	159.0764	2	**T**	12	1568.7803	784.8937	523.5983
92.0393	137,5553	**274.1034**	3	**D**	11	1467.7326	734.3699	**489.9157**
125.0621	187,0895	**373.1718**	4	**V**	10	1352.7057	**676.8564**	451.5734
167.7483	251,1188	**501.2304**	5	**Q**	9	1253.6373	627.3222	418.5506
191.4273	**286,6374**	572.2675	6	**A**	8	1125.5787	563.2929	375.8644
**284.7944**	426,688	852.3687	7	**W**-ArF	7	1054.5416	**527.7744**	352.1853
322.4891	483,23	965.4527	8	**I**	6	774.4404	387.7238	258.8183
374.5228	561,2806	**1121.5538**	9	**R**	5	661.3563	331.1818	221.1236
393.5299	589,7913	1178.5753	10	**G**	4	**505.2552**	253.1312	169.0899
446.8735	**669,8066**	1338.6059	11	**C** -CAM	3	448.2337	224.6205	150.0827
498.9072	747,8572	1494.7071	12	**R**	2	288.2031	144.6051	96.7392
		1607.7912	13	**L**	1	132.1019	66.5546	44.7055

Complete list of matched ions

Meas. m/z	Calc. m/z	δ (Da)	z	Annotation
173.1233	173.0921	0.0312	2	a4 [1-4]
178.0481	178.0842	-0.0361	2	b4 -H2O [1-4]
233.0849	233.1083	-0.0233	2	b5 -H2O -H2O [1-5]
251.0468	251.1188	-0.072	2	b5 [1-5]
274.1517	274.1034	0.0484	1	b3 [1-3]

278.1059	278.1241	-0.0182	2	b6 -NH3 [1-6]
285.1509	285.1224	0.0285	3	b7 +H2O -NH3 [1-7] [1xArylation(pF)]
292.1265	292.1139	0.0126	1	b3 +H2O [1-3]
309.1231	309.1557	-0.0326	1	a4 -H2O -H2O [1-4]
309.1606	309.1557	0.0048	1	a4 -H2O -H2O [1-4]
327.1589	327.1663	-0.0074	1	a4 -H2O [1-4]
359.1429	359.1876	-0.0448	3	a9 -H2O [1-9] [1xArylation(pF)]
372.1766	372.1913	-0.0146	3	a10 -H2O -H2O [1-10] [1xArylation(pF)]
373.1786	373.1718	0.0068	1	b4 [1-4]
456.1785	456.2089	-0.0304	1	a5 -NH3 [1-5]
473.2044	473.2354	-0.031	1	a5 [1-5]
478.2069	478.2367	-0.0298	3	y11 -H2O -NH3 [3-13] [1xArylation(pF); 1xCarbamidomethyl]
478.2069	478.2245	-0.0177	3	a12 -NH3 -NH3 [1-12] [1xArylation(pF); 1xCarbamidomethyl]
488.194	488.2286	-0.0346	1	y4 -NH3 [10-13] [1xCarbamidomethyl]
519.2318	519.2611	-0.0293	2	y7 -NH3 [7-13] [1xArylation(pF); 1xCarbamidomethyl]
519.2318	519.2409	-0.0091	1	b5 +H2O [1-5]
527.2394	527.246	-0.0066	1	a6 -NH3 [1-6]
529.2124	529.2725	-0.0601	2	a9 -H2O -H2O [1-9] [1xArylation(pF)]
547.2333	547.2831	-0.0498	2	a9 [1-9] [1xArylation(pF)]
552.7454	552.7673	-0.0218	2	b9 -NH3 [1-9] [1xArylation(pF)]
558.2275	558.2753	-0.0478	2	a10 -H2O -NH3 [1-10] [1xArylation(pF)]
558.2558	558.2753	-0.0195	2	a10 -H2O -NH3 [1-10] [1xArylation(pF)]
558.7559	558.7673	-0.0114	2	a10 -NH3 -NH3 [1-10] [1xArylation(pF)]
561.2711	561.2806	-0.0095	2	b9 [1-9] [1xArylation(pF)]
668.2965	668.3432	-0.0467	2	y10 -NH3 [4-13] [1xArylation(pF); 1xCarbamidomethyl]
669.7767	669.8066	-0.0299	2	b11 [1-11] [1xArylation(pF); 1xCarbamidomethyl]
807.3196	807.3472	-0.0276	1	a7 -NH3 [1-7] [1xArylation(pF)]
1105.501	1105.511	-0.0098	1	b9 +H2O -NH3 -NH3 [1-9] [1xArylation(pF)]
1121.518	1121.554	-0.0359	1	b9 [1-9] [1xArylation(pF)]
1122.522	1122.538	-0.0155	1	b9 +H2O -NH3 [1-9] [1xArylation(pF)]

Peptide IVSDGNGMNAW^{126}VAW^{129}RNR [Sequence range: 116-132]

The MS^2 data was obtained from the tryptic peptide selecting m/z 717.3177 for collision-induced dissociation with 29.3 eV of collision energy. The table with some of the predicted fragments is listed, and the main matching parameters are highlighted in red. A detailed table of matching parameters is also provided.

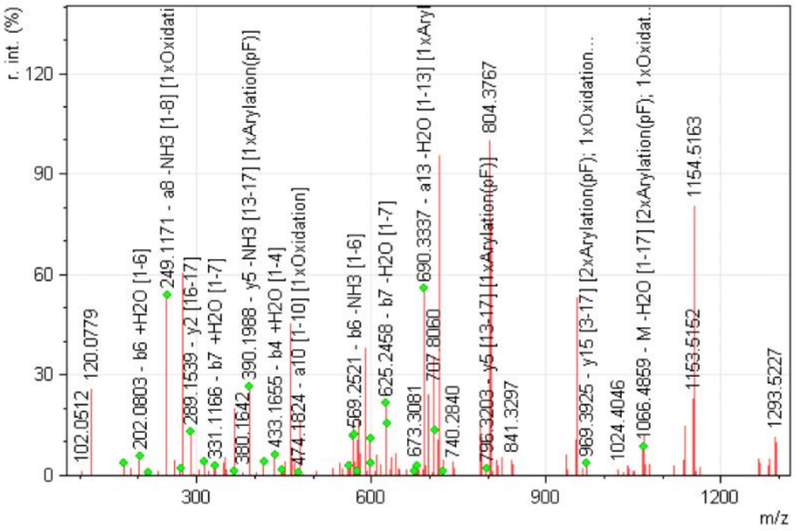

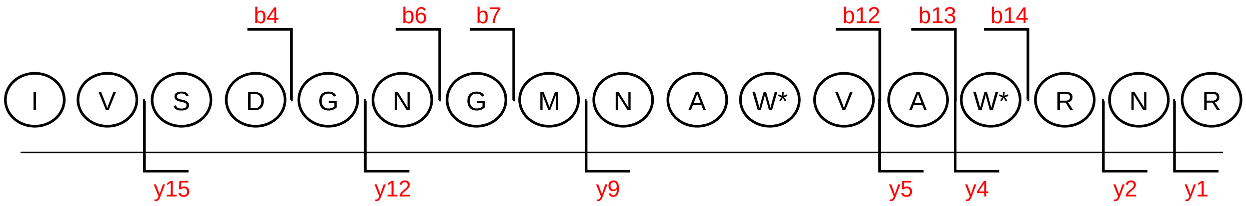

Predicted b and y ions

(Matched ions highlighted in red. Predicted values)

b^{3+}	b^{2+}	b^{+}		Sequence		y^{+}	y^{2+}	y^{3+}
			1	**I**	17			
71.7248	107.0835	213.1598	2	**V**	16	2036.9	1018.953	679.638
100.7354	150.5995	300.1918	3	**S**	15	1937.831	**969.4192**	646.6152
139.0778	208.113	**415.2187**	4	**D**	14	1850.799	925.9032	617.6046
158.0849	236.6237	472.2402	5	**G**	13	1735.772	868.3897	579.2622
196.0992	293.6452	**586.2831**	6	**N**	12	1678.751	839.879	**560.2551**
215.1064	322.1559	**643.3046**	7	**G**	11	1564.708	782.8575	522.2408
264.1182	395.6736	790.34	8	**M**	10	1507.686	754.3468	503.2336
302.1325	452.6951	904.3829	9	**N**	9	1360.651	**680.8291**	454.2218
325.8115	488.2136	975.42	10	**A**	8	1246.608	623.8076	416.2075
419.1786	628.2642	1255.521	11	**W**-ArF	7	1175.571	588.2891	392.5285
452.2014	**677.7985**	1354.59	12	**V**	6	895.4697	448.2385	299.1614
475.8804	**713.317**	1425.627	13	**A**	5	**796.4013**	398.7043	266.1386
**569.2475**	853.3676	1705.728	14	**W**-ArF	4	**725.3642**	363.1857	242.4596
621.2812	931.4182	1861.829	15	**R**	3	445.263	223.1351	149.0925
659.2955	988.4396	1975.872	16	**N**	2	**289.1619**	145.0846	97.0588
			17	**R**	1	**175.119**	88.0631	59.0445

Complete list of matched ions

Meas. m/z	Calc. m/z	δ (Da)	z	Annotation
175.114	175.119	-0.005	1	y1 [17-17]
202.0803	202.1027	-0.0224	3	b6 +H2O [1-6]
217.0916	217.1183	-0.0267	2	b4 +H2O [1-4]

249.1171	249.111	0.0061	3	a8 -NH3 [1-8] [1xOxidation]
272.1272	272.1969	-0.0697	1	a3 [1-3]
272.1272	272.1353	-0.0082	1	y2 -NH3 [16-17]
289.1539	289.1619	-0.008	1	y2 [16-17]
313.1057	313.1506	-0.045	2	b7 -H2O [1-7]
331.1166	331.1612	-0.0446	2	b7 +H2O [1-7]
363.161	363.1857	-0.0247	2	y4 [14-17] [1xArylation(pF)]
390.1988	390.191	0.0078	2	y5 -NH3 [13-17] [1xArylation(pF)]
415.1557	415.2187	-0.063	1	b4 [1-4]
433.1655	433.2293	-0.0638	1	b4 +H2O [1-4]
446.1567	446.1979	-0.0411	3	b12 -H2O [1-12] [1xArylation(pF); 1xOxidation]
474.1824	474.2162	-0.0338	2	a10 [1-10] [1xOxidation]
560.2387	560.2551	-0.0164	3	y12 [6-17] [2xArylation(pF); 1xOxidation]
568.2526	568.2726	-0.0199	1	b6 -H2O[1-6]
569.2521	569.2566	-0.0045	1	b6 -NH3 [1-6]
569.2521	569.2475	0.0046	3	b14 [1-14] [2xArylation(pF); 1xOxidation]
575.2031	575.251	-0.0479	3	b14 +H2O [1-14] [2xArylation(pF); 1xOxidation]
597.2525	597.2991	-0.0466	1	a7 -H2O [1-7]
598.2538	598.2831	-0.0293	1	a7 -NH3 [1-7]
625.2458	625.294	-0.0482	1	b7 -H2O [1-7]
626.249	626.278	-0.0291	1	b7 -NH3 [1-7]
672.3242	672.3158	0.0083	2	y9 -NH3 [9-17] [2xArylation(pF)]
678.2451	678.2905	-0.0453	2	b12 +H2O -NH3 [1-12] [1xArylation(pF); 1xOxidation]
690.3337	690.3143	0.0194	2	a13 -H2O [1-13] [1xArylation(pF); 1xOxidation]
708.3042	708.3376	-0.0334	1	y4 -NH3 [14-17] [1xArylation(pF)]
722.2719	722.3223	-0.0504	2	b13 +H2O [1-13] [1xArylation(pF); 1xOxidation]
796.3203	796.4013	-0.081	1	y5 [13-17] [1xArylation(pF)]
969.3925	969.4192	-0.0268	2	y15 [3-17] [2xArylation(pF); 1xOxidation]
1066.486	1066.49	-0.0043	2	M -H2O [1-17] [2xArylation(pF); 1xOxidation]

Peptide $W^{80}W^{81}$CNDGR [Sequence range: 80-86]

The MS^2 data was obtained from the tryptic peptide selecting m/z 544.2027 for collision-induced dissociation with 29.7 eV of collision energy. The table with some of the predicted fragments is listed, and the main matching parameters are highlighted in red. A detailed table of matching parameters is also provided.

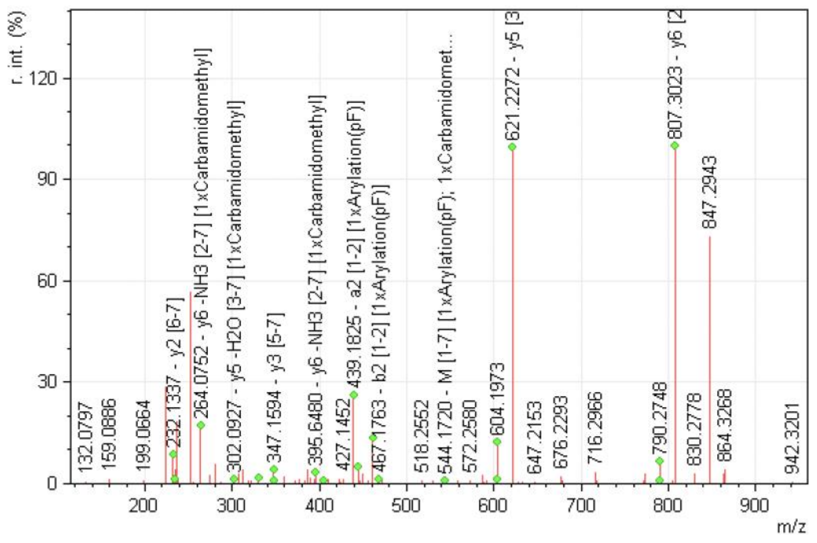

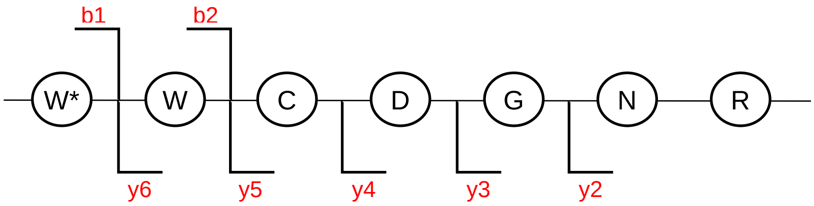

Predicted b and y ions

(Matched ions highlighted in red. Predicted values)

b^{3+}	b^{2+}	b^{+}		Sequence		y^{+}	y^{2+}	y^{3+}
		**281.1085**	1	**W**-ArF	7			
156.4008	234.0975	**467.1878**	2	**W**	6	**807.3202**	404.1638	269.7783
209.7443	314.1129	627.2184	3	**C**-CAM	5	**621.2409**	311.1241	207.7518
247.7586	371.1343	741.2614	4	**N**	4	**461.2103**	231.1088	154.4083
286.101	428.6478	856.2883	5	**D**	3	**347.1674**	174.0873	116.394
305.1081	457.1585	913.3098	6	**G**	2	**232.1404**	116.5738	78.0517
			7	**R**	1	175.119	88.0631	59.0445

Complete list of matched ions

Meas. m/z	Calc. m/z	δ (Da)	z	Annotation
232.1337	232.1404	-0.0067	1	y2 [6-7]
234.0643	234.0975	-0.0332	2	b2 [1-2] [1xArylation(pF)]
264.0752	264.1028	-0.0276	3	y6 -NH3 [2-7] [1xCarbamidomethyl]
281.1000	281.1085	+0.0085	1	b1 [1xArylation(pF)]
302.0927	302.1188	-0.0262	2	y5 -H2O [3-7] [1xCarbamidomethyl]
312.1235	312.1302	-0.0067	1	y3 -H2O -NH3 [5-7]
312.1479	312.1302	0.0176	1	y3 -H2O -NH3 [5-7]
330.1323	330.1408	-0.0085	1	y3 -NH3 [5-7]
347.0966	347.1674	-0.0707	1	y3 [5-7]
347.1213	347.1674	-0.046	1	y3 [5-7]
347.1594	347.1674	-0.0079	1	y3 [5-7]
387.1038	387.1372	-0.0334	2	y6 -NH3 -NH3 [2-7] [1xCarbamidomethyl]

395.648	395.6505	-0.0025	2	y6 -NH3 [2-7] [1xCarbamidomethyl]
404.1585	404.1638	-0.0053	2	y6 [2-7] [1xCarbamidomethyl]
427.1452	427.1572	-0.012	1	y4 -NH3 -NH3 [4-7]
439.1825	439.1929	-0.0104	1	a2 [1-2] [1xArylation(pF)]
444.1747	444.1837	-0.0091	1	y4 -NH3 [4-7]
461.1992	461.2103	-0.0111	1	y4 [4-7]
467.1763	467.1878	-0.0115	1	b2 [1-2] [1xArylation(pF)]
544.172	544.2144	-0.0424	2	M [1-7] [1xArylation(pF); 1xCarbamidomethyl]
587.165	587.1878	-0.0228	1	y5 -NH3 -NH3 [3-7] [1xCarbamidomethyl]
603.213	603.2304	-0.0174	1	y5 -H2O [3-7] [1xCarbamidomethyl]
604.1973	604.2144	-0.0171	1	y5 -NH3 [3-7] [1xCarbamidomethyl]
621.2272	621.2409	-0.0137	1	y5 [3-7] [1xCarbamidomethyl]
772.2744	772.2831	-0.0087	1	y6 -H2O -NH3 [2-7] [1xCarbamidomethyl]
773.2464	773.2671	-0.0208	1	y6 -NH3 -NH3 [2-7] [1xCarbamidomethyl]
789.3001	789.3097	-0.0096	1	y6 -H2O [2-7] [1xCarbamidomethyl]
790.2748	790.2937	-0.0189	1	y6 -NH3 [2-7] [1xCarbamidomethyl]
807.3023	807.3202	-0.018	1	y6 [2-7] [1xCarbamidomethyl]

Peptide W[80]W[81]CNDGR [Sequence range: 80-86]

The MS^2 data was obtained from the tryptic peptide selecting m/z 544.2162 for collision-induced dissociation with 29.7 eV of collision energy. The table with some of the predicted fragments is listed, and the main matching parameters are highlighted in red. A detailed table of matching parameters is also provided.

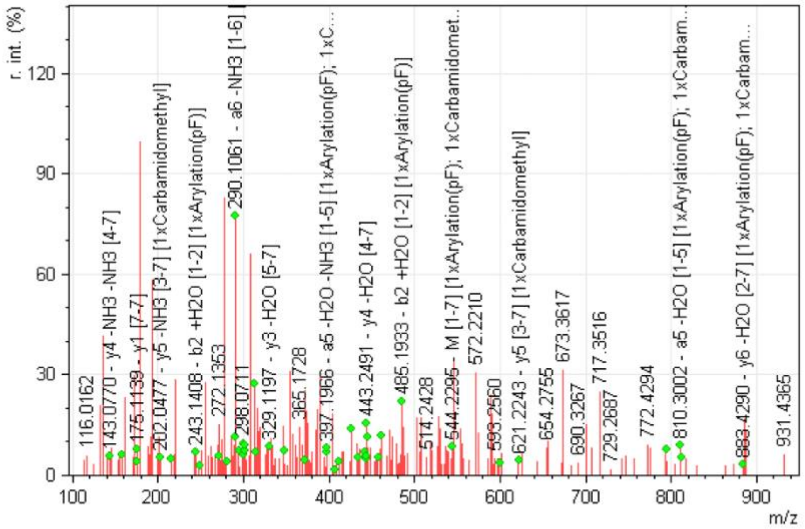

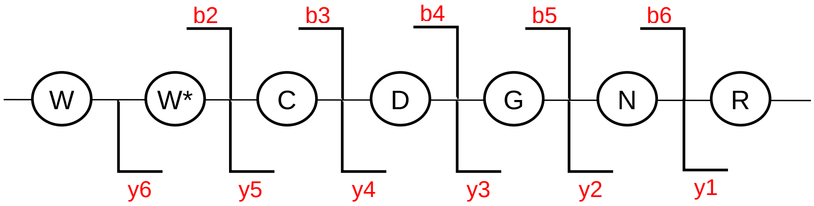

Predicted b and y ions

(Matched ions highlighted in red. Predicted values)

b^{3+}	b^{2+}	b^{+}		Sequence		y^{+}	y^{2+}	y^{3+}
		187.0867	1	**W**	7			
156.4008	234.0975	**467.1878**	2	**W**-ArF	6	**901.3421**	451.1747	301.1189
209.7443	**314.1129**	627.2184	3	**C**-CAM	5	**621.2409**	311.1241	207.7518
247.7586	**371.1343**	741.2614	4	**N**	4	**461.2103**	231.1088	154.4083
286.101	**428.6478**	856.2883	5	**D**	3	**347.1674**	174.0873	116.394
305.1081	**457.1585**	913.3098	6	**G**	2	**232.1404**	116.5738	78.0517
			7	**R**	1	**175.119**	88.0631	59.0445

Complete list of matched ions

Meas. m/z	Calc. m/z	δ (Da)	z	Annotation
143.077	143.0572	0.0197	3	y4 -NH3 -NH3 [4-7]
158.0553	158.0924	-0.0371	1	y1 -NH3 [7-7]
175.0873	175.119	-0.0317	1	y1 [7-7]
175.1139	175.119	-0.005	1	y1 [7-7]
202.0477	202.0763	-0.0286	3	y5 -NH3 [3-7] [1xCarbamidomethyl]
215.099	215.1139	-0.0149	1	y2 -NH3 [6-7]
243.1408	243.1028	0.038	2	b2 +H2O [1-2] [1xArylation(pF)]
248.1018	248.0866	0.0152	3	b4 +H2O -NH3 [1-4] [1xArylation(pF); 1xCarbamidomethyl]
271.1685	271.0938	0.0747	3	a5 -NH3 [1-5] [1xArylation(pF); 1xCarbamidomethyl]
280.1289	280.0974	0.0315	3	b5 -H2O [1-5] [1xArylation(pF); 1xCarbamidomethyl]
290.1061	290.101	0.0051	3	a6 -NH3 [1-6] [1xArylation(pF); 1xCarbamidomethyl]
290.1401	290.101	0.0391	3	a6 -NH3 [1-6] [1xArylation(pF); 1xCarbamidomethyl]
294.1406	294.0976	0.0431	2	y5 -NH3 -NH3 [3-7] [1xCarbamidomethyl]
299.0629	299.1046	-0.0417	3	b6 -H2O [1-6] [1xArylation(pF); 1xCarbamidomethyl]
300.1273	300.1154	0.0119	2	a3 [1-3] [1xArylation(pF); 1xCarbamidomethyl]
301.1092	301.1189	-0.0097	3	y6 [2-7] [1xArylation(pF); 1xCarbamidomethyl]
301.1768	301.1189	0.0579	3	y6 [2-7] [1xArylation(pF); 1xCarbamidomethyl]
312.1464	312.1302	0.0161	1	y3 -H2O -NH3 [5-7]
314.1833	314.1129	0.0704	2	b3 [1-3] [1xArylation(pF); 1xCarbamidomethyl]
329.1197	329.1568	-0.0371	1	y3 -H2O [5-7]
347.1314	347.1674	-0.0359	1	y3 [5-7]
371.1862	371.1343	0.0519	2	b4 [1-4] [1xArylation(pF); 1xCarbamidomethyl]
397.1543	397.1318	0.0226	2	a5 -H2O -NH3 [1-5] [1xArylation(pF); 1xCarbamidomethyl]
397.1966	397.1318	0.0648	2	a5 -H2O -NH3 [1-5] [1xArylation(pF); 1xCarbamidomethyl]
405.6537	405.645	0.0087	2	a5 -H2O [1-5] [1xArylation(pF); 1xCarbamidomethyl]
411.1873	411.1292	0.058	2	b5 -H2O -NH3 [1-5] [1xArylation(pF); 1xCarbamidomethyl]
426.1696	426.1732	-0.0036	1	y4 -H2O -NH3 [4-7]
434.1623	434.1558	0.0065	2	a6 -H2O [1-6] [1xArylation(pF); 1xCarbamidomethyl]
434.1623	434.1482	0.0142	2	y6 -NH3 -NH3 [2-7] [1xArylation(pF); 1xCarbamidomethyl]
442.1232	442.1694	-0.0462	2	y6 -H2O [2-7] [1xArylation(pF); 1xCarbamidomethyl]
442.2181	442.1694	0.0487	2	y6 -H2O [2-7] [1xArylation(pF); 1xCarbamidomethyl]
443.1955	443.1997	-0.0043	1	y4 -H2O [4-7]

443.1955	443.1611	0.0344	2	a6 [1-6] [1xArylation(pF); 1xCarbamidomethyl]
443.2491	443.1997	0.0494	1	y4 -H2O [4-7]
443.2491	443.1611	0.088	2	a6 [1-6] [1xArylation(pF); 1xCarbamidomethyl]
444.1875	444.1837	0.0038	1	y4 -NH3 [4-7]
457.1637	457.1585	0.0052	2	b6 [1-6] [1xArylation(pF); 1xCarbamidomethyl]
461.1453	461.2103	-0.065	1	y4 [4-7]
485.1933	485.1983	-0.0051	1	b2 +H2O [1-2] [1xArylation(pF)]
544.2295	544.2144	0.0151	2	M [1-7] [1xArylation(pF); 1xCarbamidomethyl]
599.3092	599.2235	0.0856	1	a3 [1-3] [1xArylation(pF); 1xCarbamidomethyl]
621.2243	621.2409	-0.0166	1	y5 [3-7] [1xCarbamidomethyl]
793.2998	793.2563	0.0435	1	a5 -H2O -NH3 [1-5] [1xArylation(pF); 1xCarbamidomethyl]
810.3002	810.2828	0.0174	1	a5 -H2O [1-5] [1xArylation(pF); 1xCarbamidomethyl]
811.3284	811.2668	0.0616	1	a5 -NH3 [1-5] [1xArylation(pF); 1xCarbamidomethyl]
883.429	883.3316	0.0974	1	y6 -H2O [2-7] [1xArylation(pF); 1xCarbamidomethyl]

Peptide GYSLGNW^{46}VCAAK [Sequence range: 40-51]

The MS^2 data was obtained from the tryptic peptide selecting m/z 710.2980 for collision-induced dissociation with 28.3 eV of collision energy. The table with some of the predicted fragments is listed, and the main matching parameters are highlighted in red. A detailed table of matching parameters is also provided.

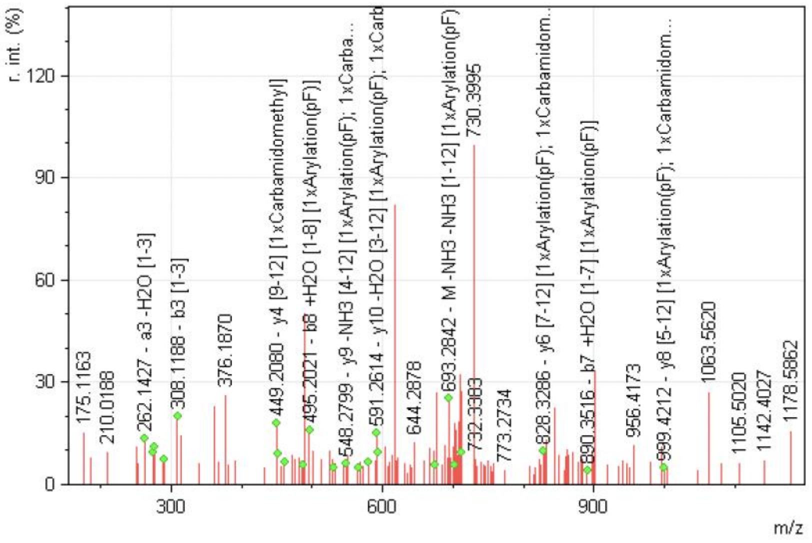

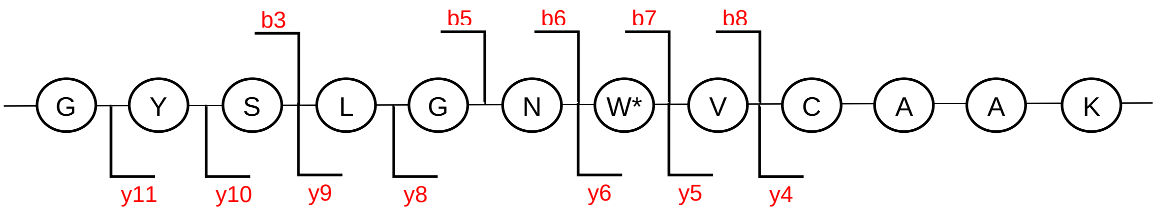

Predicted b and y ions

(Matched ions highlighted in red. Predicted values)

b^{3+}	b^{2+}	b^{+}		Sequence		y^{+}	y^{2+}	y^{3+}
			1	**G**	12			
74.3689	111.0497	221.0921	2	**Y**	11	1362.631	**681.8192**	454.8819
103.3796	154.5657	**308.1241**	3	**S**	10	1199.568	**600.2875**	400.5274
141.0742	211.1077	421.2082	4	**L**	9	1112.536	**556.7715**	371.5168
160.0814	239.6185	**478.2296**	5	**G**	8	**999.4517**	500.2295	333.8221
198.0957	296.6399	**592.2726**	6	**N**	7	942.4302	471.7187	314.8149
291.4628	436.6905	**872.3737**	7	**W-**ArF	6	**828.3873**	414.6973	276.8006
324.4856	**486.2247**	971.4422	8	**V**	5	**548.2861**	274.6467	183.4335
377.8291	566.24	1131.473	9	**C-**CAM	4	**449.2177**	225.1125	150.4107
401.5082	601.7586	1202.51	10	**A**	3	289.187	145.0972	97.0672
425.1872	637.2772	1273.547	11	**A**	2	218.1499	109.5786	73.3882
			12	**K**	1	147.1128	74.06	49.7091

Complete list of matched ions

Meas. m/z	Calc. m/z	δ (Da)	z	Annotation
262.1427	262.1186	0.024	1	a3 -H2O [1-3]
274.0993	274.1292	-0.0299	2	a6 -NH3 [1-6]
276.0945	276.1276	-0.0331	3	a7 -H2O [1-7] [1xArylation(pF)]
288.1994	288.1266	0.0727	2	b6 -NH3 [1-6]
290.0898	290.1135	-0.0238	1	b3 -H2O [1-3]
308.1188	308.1241	-0.0053	1	b3 [1-3]
449.208	449.2177	-0.0097	1	y4 [9-12] [1xCarbamidomethyl]
449.208	449.2064	0.0016	3	y11 -NH3 [2-12] [1xArylation(pF); 1xCarbamidomethyl]
450.2002	450.2347	-0.0345	1	a5 [1-5]
460.174	460.2191	-0.045	1	b5 -H2O [1-5]
486.2753	486.2247	0.0505	2	b8 [1-8] [1xArylation(pF)]
495.2021	495.23	-0.0279	2	b8 +H2O [1-8] [1xArylation(pF)]
529.2556	529.2405	0.015	1	a6 -H2O -NH3 [1-6]
548.2799	548.2861	-0.0062	1	y5 [8-12] [1xCarbamidomethyl]
548.2799	548.2582	0.0216	2	y9 -NH3 [4-12] [1xArylation(pF); 1xCarbamidomethyl]
564.2559	564.2776	-0.0218	1	a6 [1-6]
579.264	579.2479	0.0161	2	a10 -NH3 [1-10] [1xArylation(pF); 1xCarbamidomethyl]
591.2614	591.2822	-0.0208	2	y10 -H2O [3-12] [1xArylation(pF); 1xCarbamidomethyl]
592.2603	592.2726	-0.0122	1	b6 [1-6]
673.2809	673.3059	-0.025	2	y11 -NH3 [2-12] [1xArylation(pF); 1xCarbamidomethyl]
693.2842	693.3034	-0.0191	2	M -NH3 -NH3 [1-12] [1xArylation(pF); 1xCarbamidomethyl]
701.2741	701.3246	-0.0505	2	M -H2O [1-12] [1xArylation(pF); 1xCarbamidomethyl]
710.3499	710.3299	0.02	2	M [1-12] [1xArylation(pF); 1xCarbamidomethyl]
828.3286	828.3873	-0.0587	1	y6 [7-12] [1xArylation(pF); 1xCarbamidomethyl]
890.3516	890.3843	-0.0327	1	b7 +H2O [1-7] [1xArylation(pF)]
999.4212	999.4517	-0.0305	1	y8 [5-12] [1xArylation(pF); 1xCarbamidomethyl]

8.3. Product characterization – With addition of L-methionine as antioxidant

After the reaction time, reaction containing L-Methionine as antioxidant agent was purified following the same protocol as described above and was characterized at the intact level using mass spectrometry analysis. The results are depicted in the figures below.

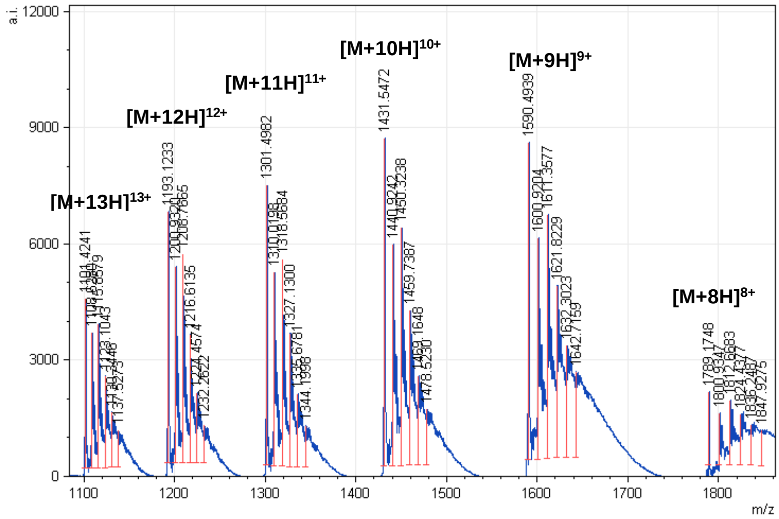

Figure S39: Intact mass spectrum of enzyme after reaction in the presence of Methionine.

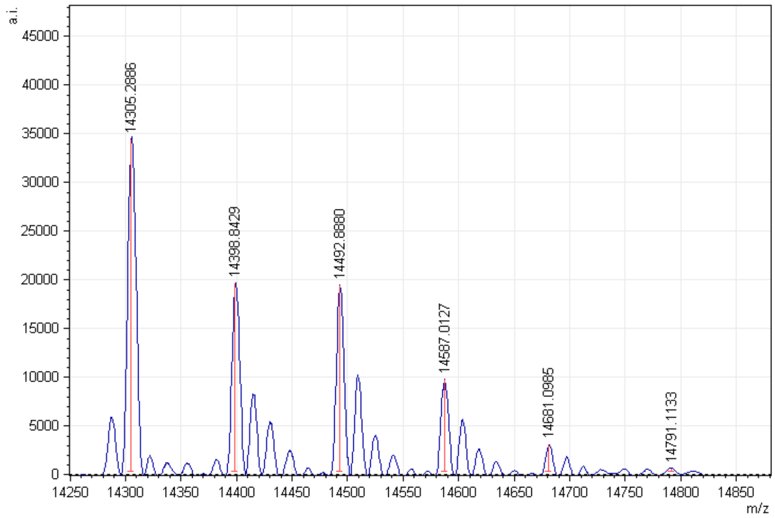

Figure S40: Deconvoluted intact mass spectrum of enzyme after reaction in the presence of Methionine.

We can clearly see from the results that the addition of 25 equivalents of L-Methionine significantly decreased the rate of oxidation when compared to the results outlined in section 8.2.

9. NMR Spectra

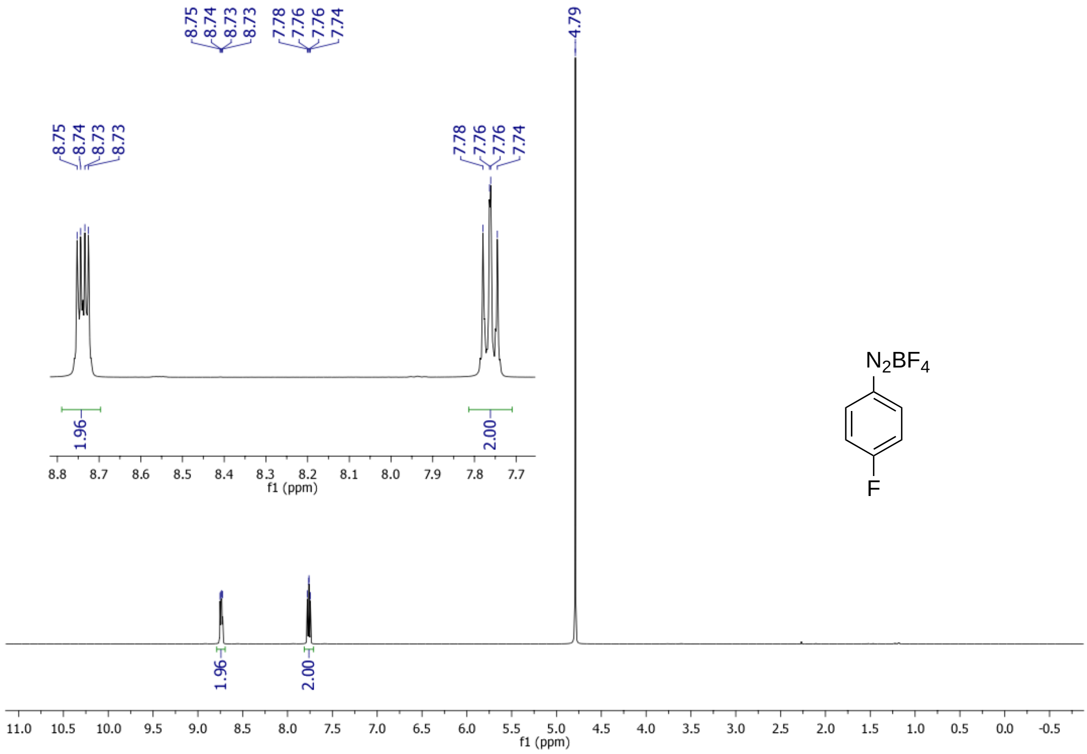

^{1}**H NMR (500 MHz, D$_2$O)** of compound **2**.

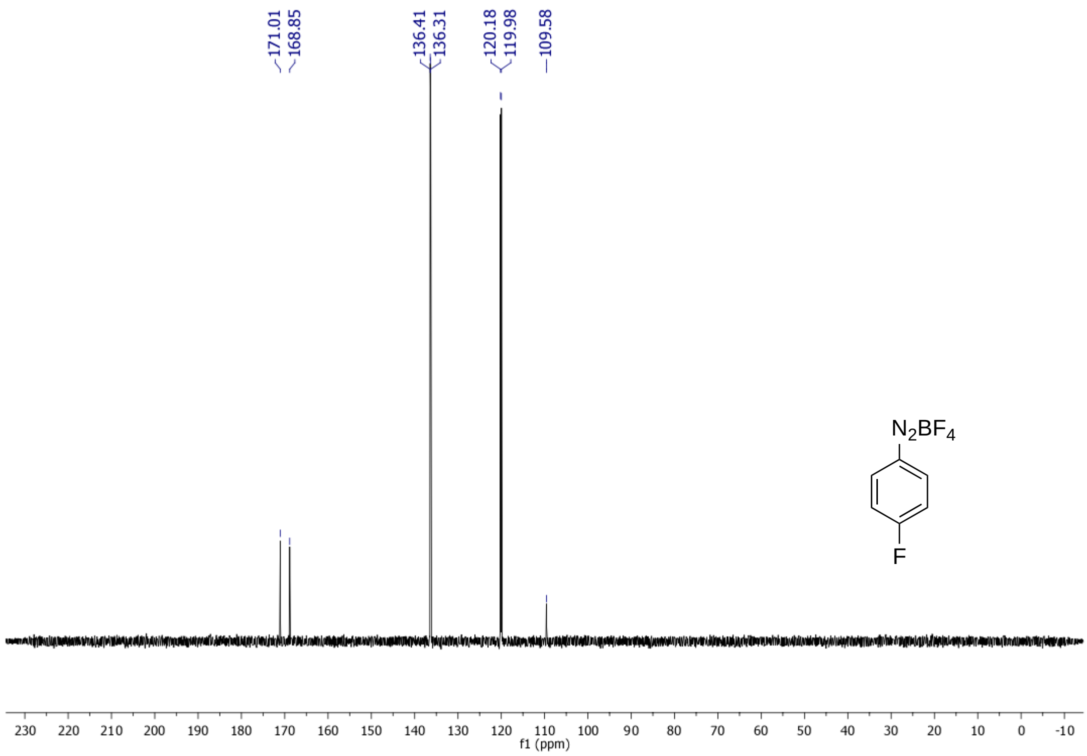

^{13}**C NMR (126 MHz, D$_2$O)** of compound **2**.

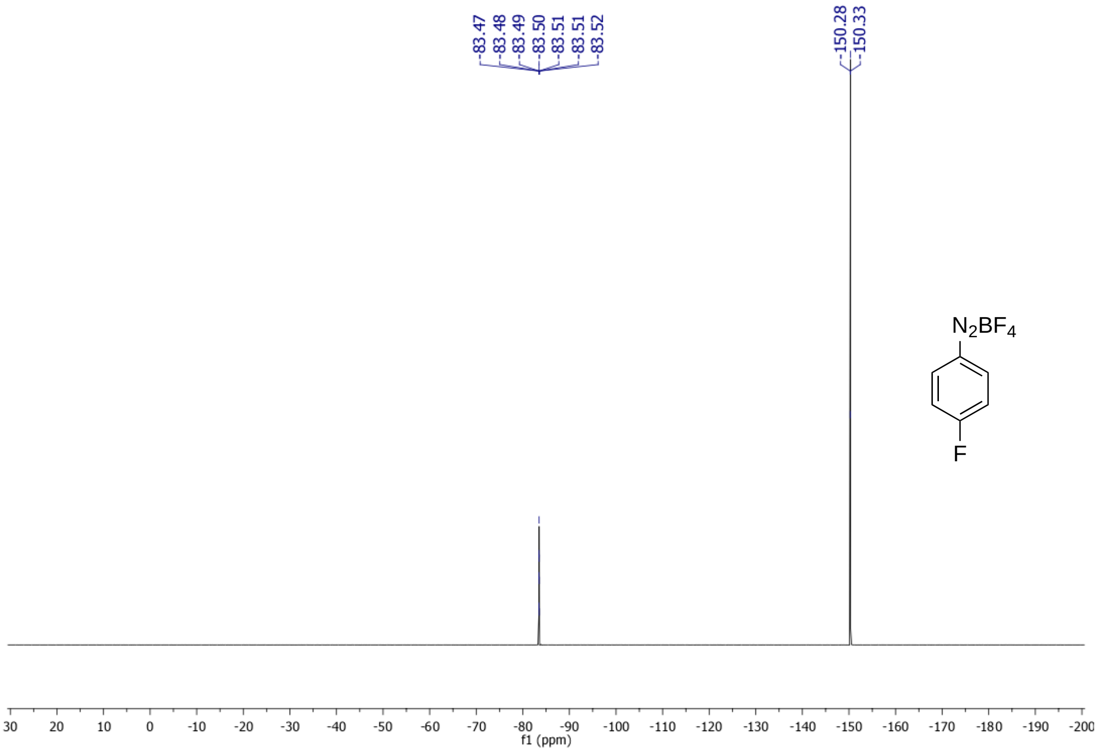

^{19}F NMR (470 MHz, D_2O) of Compound **2**.

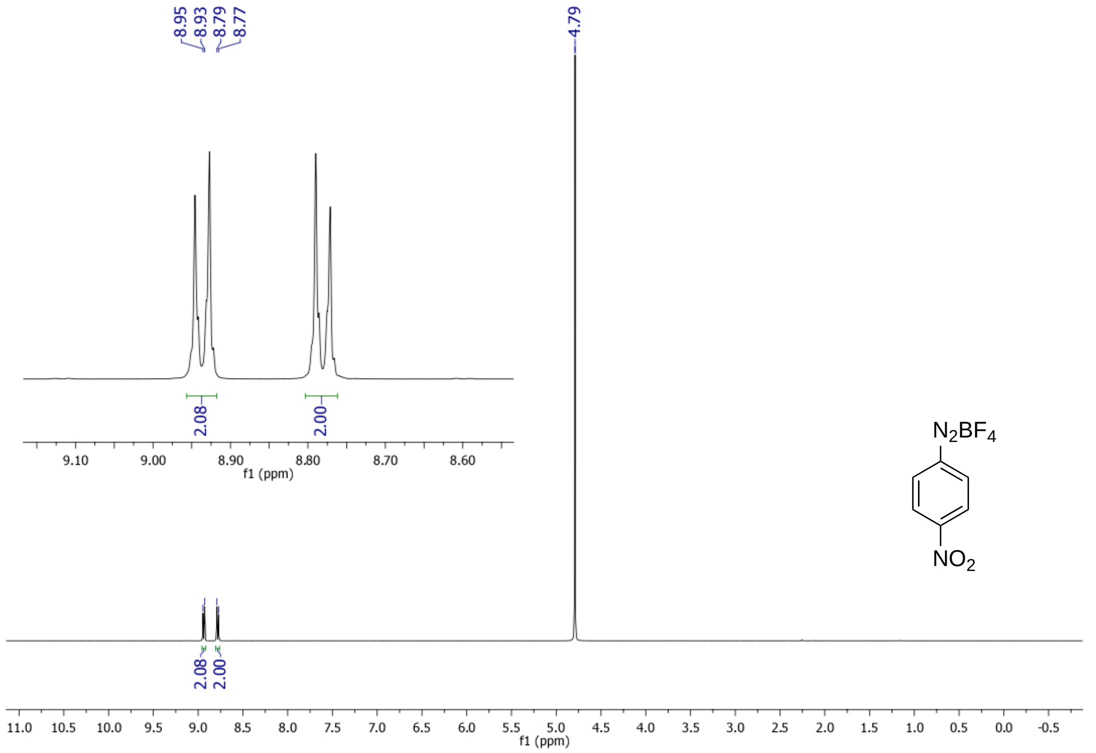

^{1}H NMR (500 MHz, D_2O) of compound **45**.

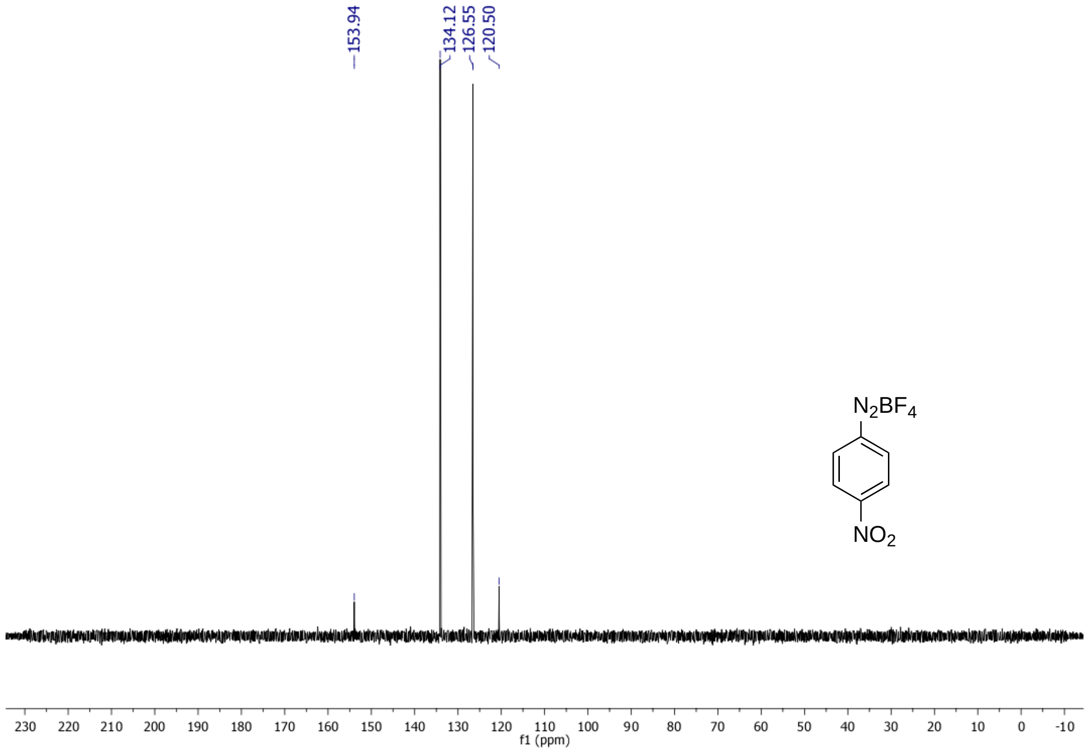

^{13}C NMR (126 MHz, D_2O) of compound **45**.

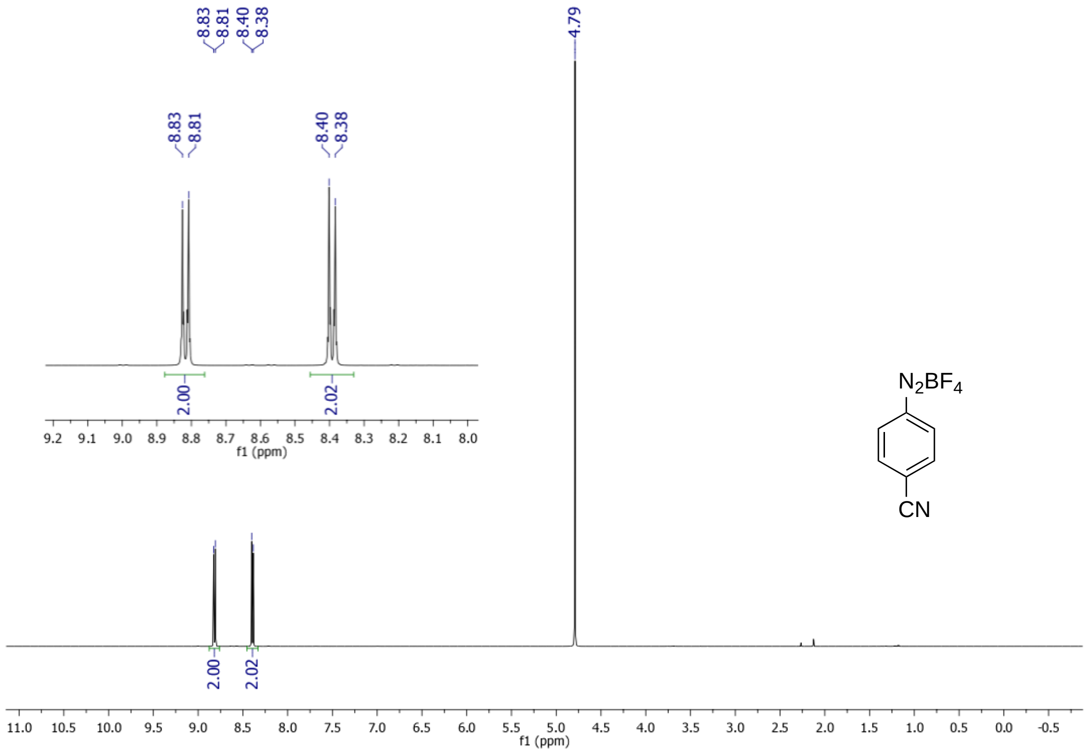

^{1}H NMR (500 MHz, D_2O) of compound **46**.

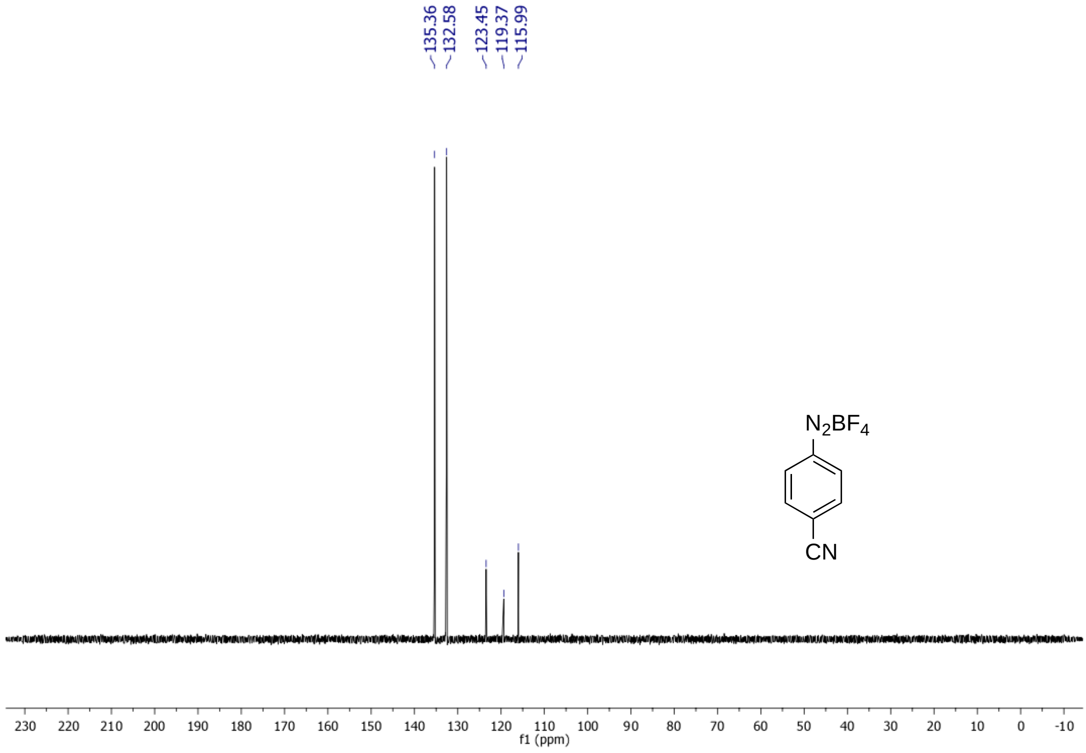

^{13}C NMR (126 MHz, D_2O) of compound 46.

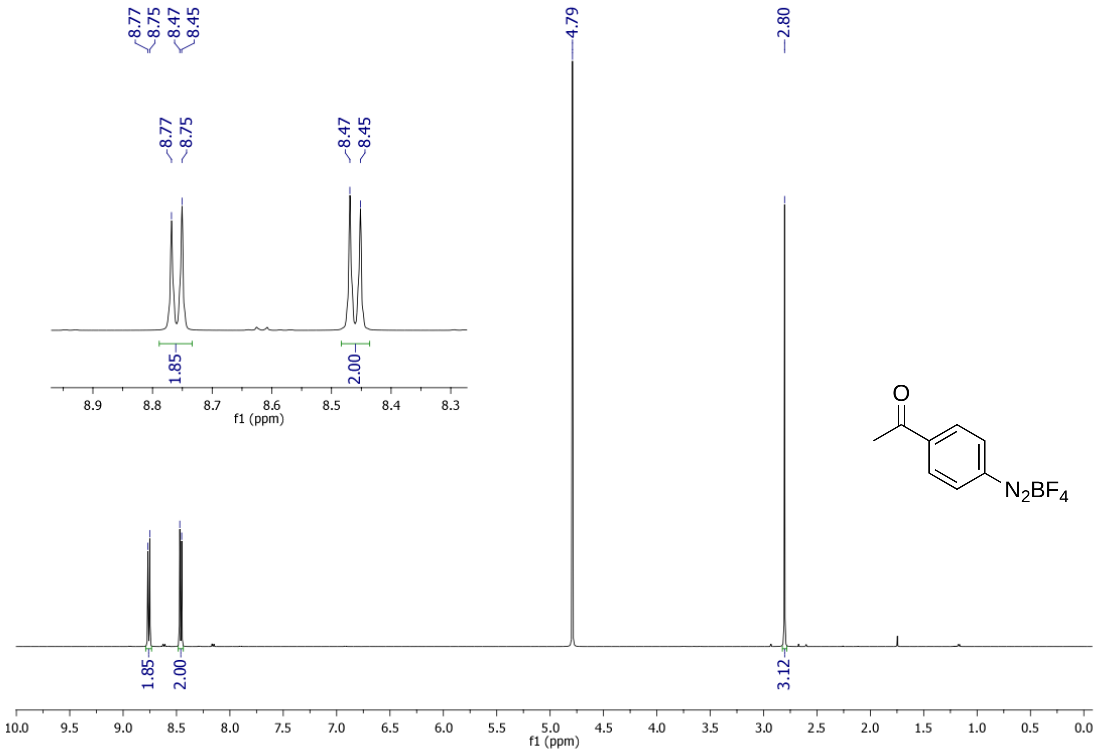

^{1}H NMR (500 MHz, D_2O) of compound 47.

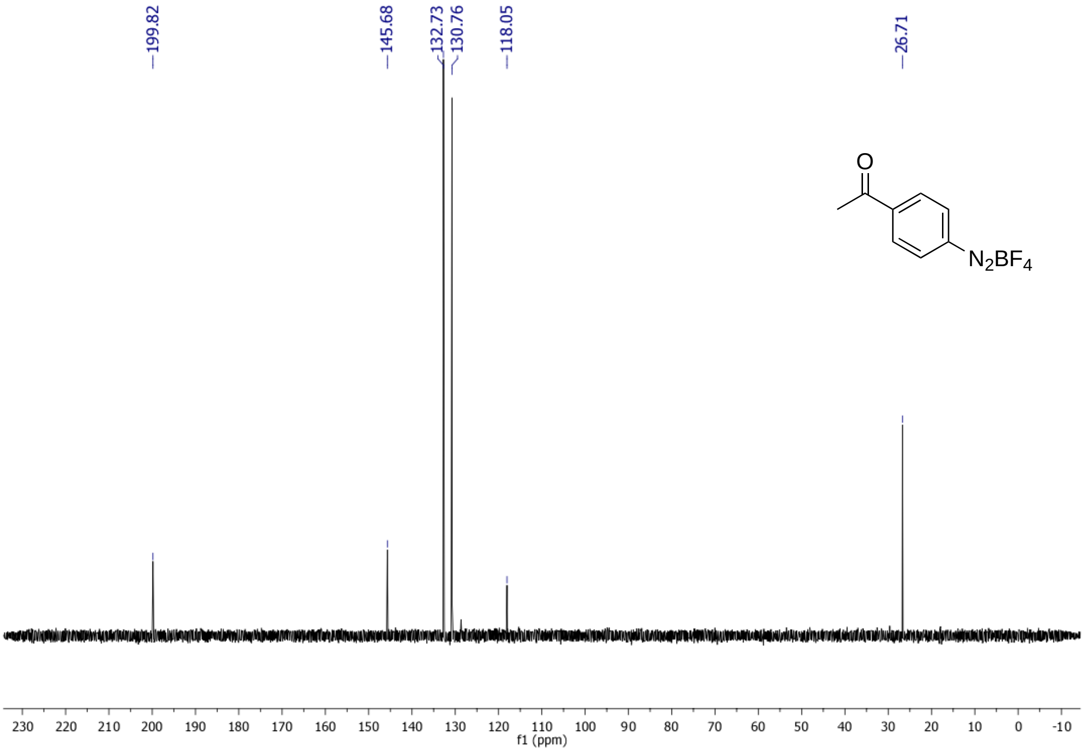

^{13}C NMR (126 MHz, D_2O) of compound **47**.

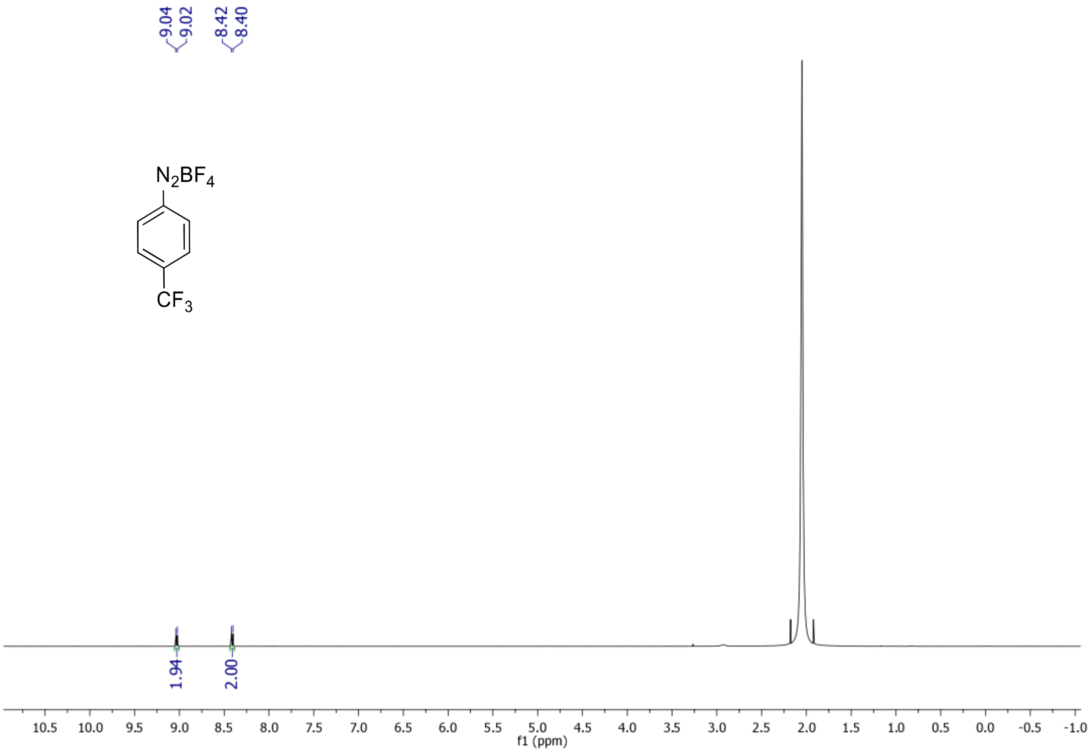

^{1}H NMR (500 MHz, Acetone) of compound **48**.

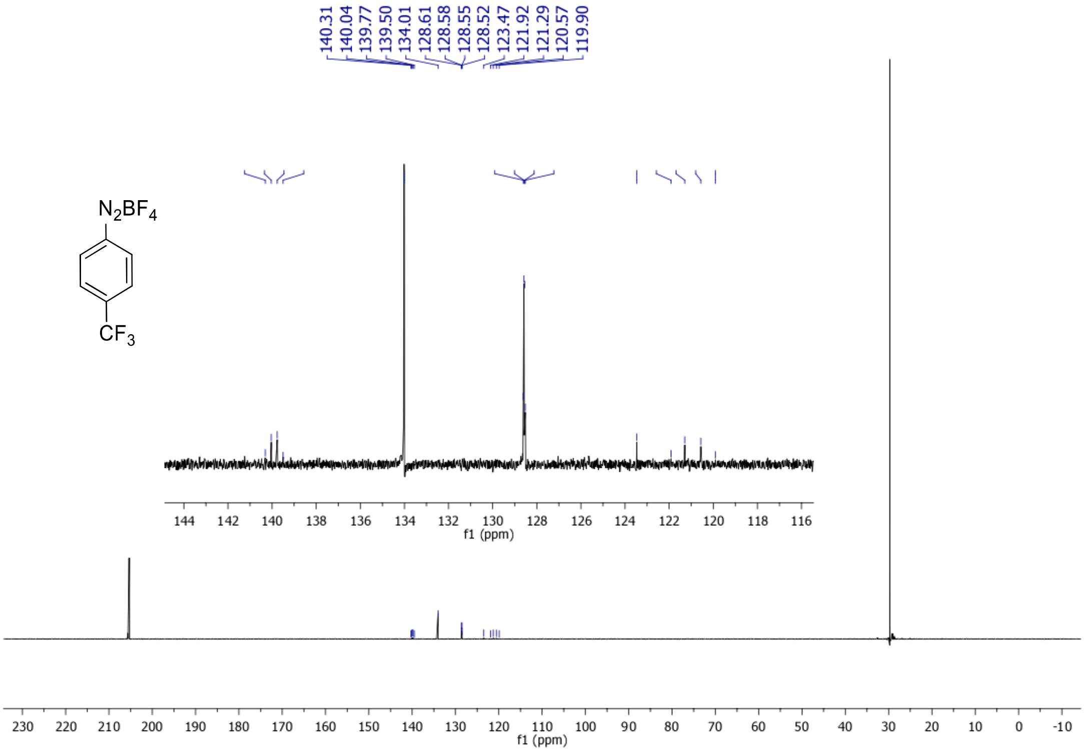

^{13}C NMR (126 MHz, Acetone) of compound **48**.

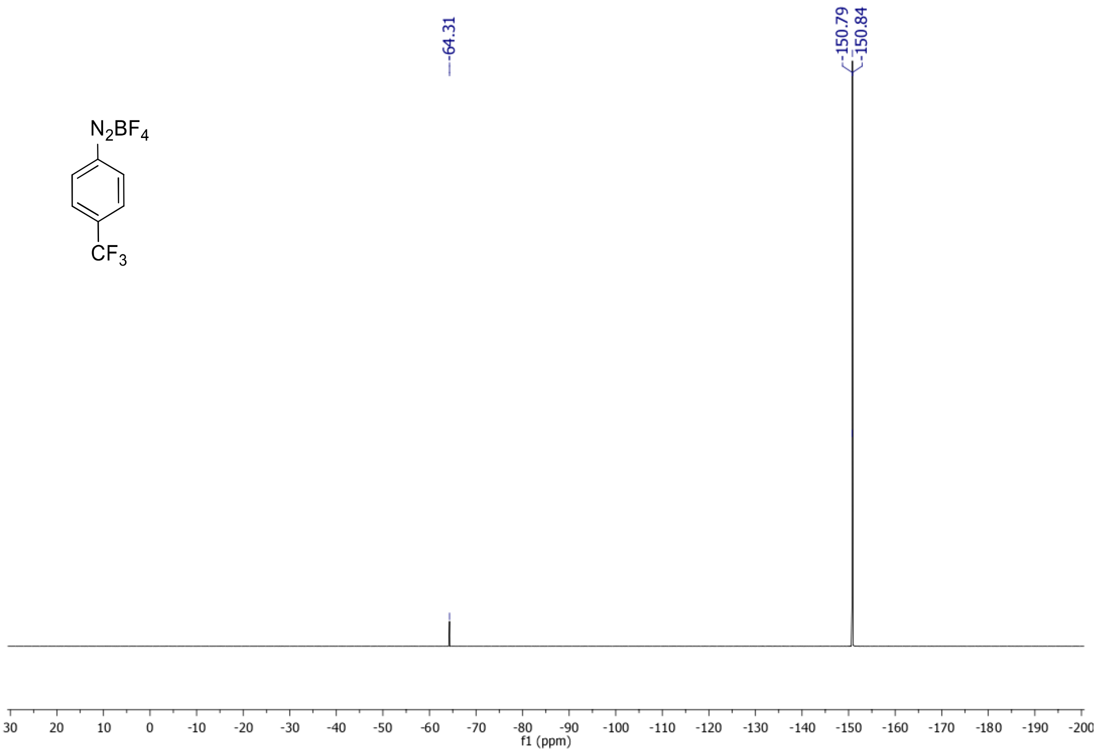

^{19}F NMR (470 MHz, D_2O) of Compound **48**.

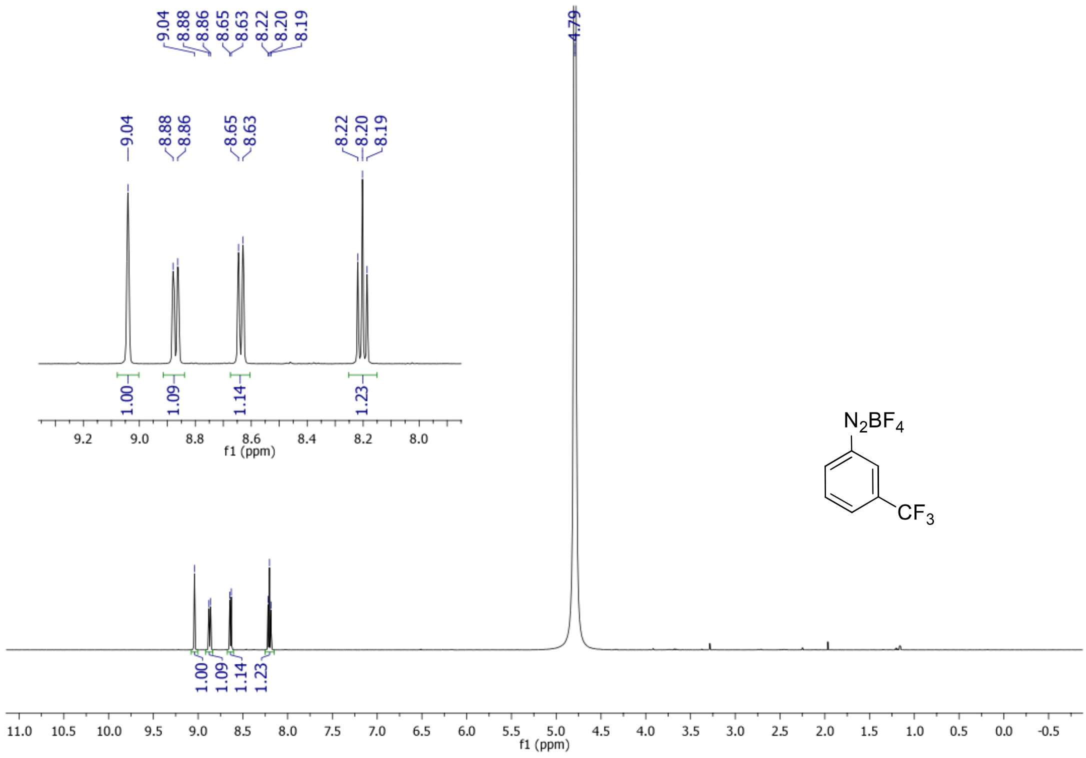

^{1}H NMR (500 MHz, D_2O) of compound **49**.

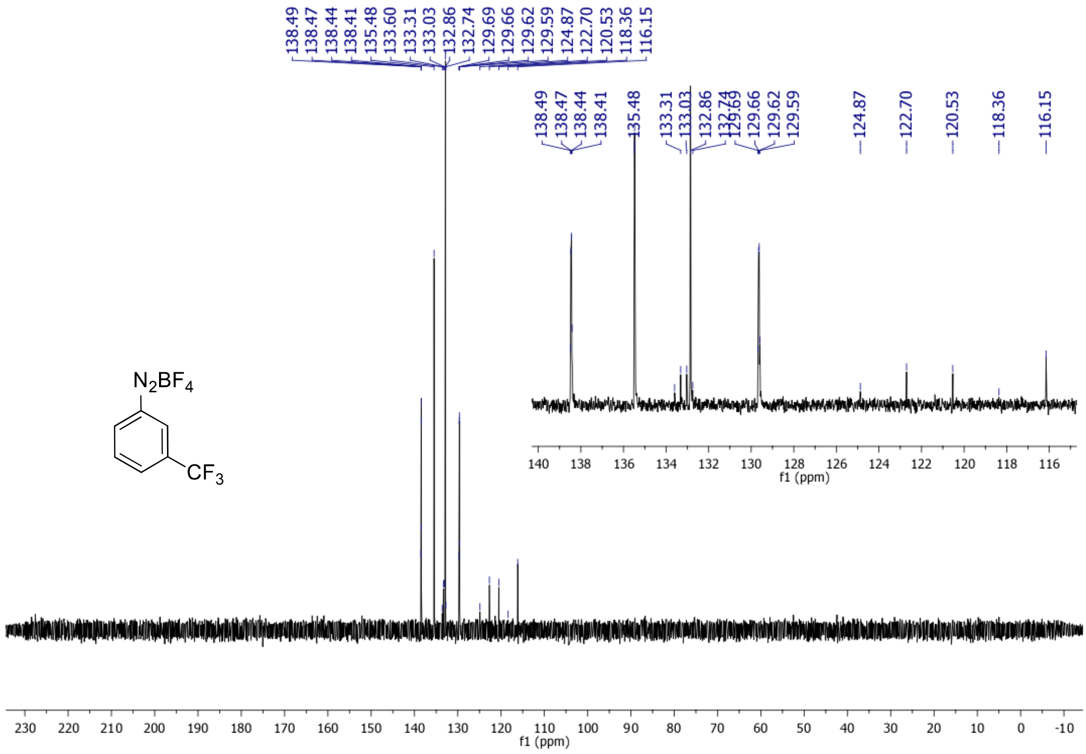

^{13}C NMR (126 MHz, D_2O) of compound **49**.

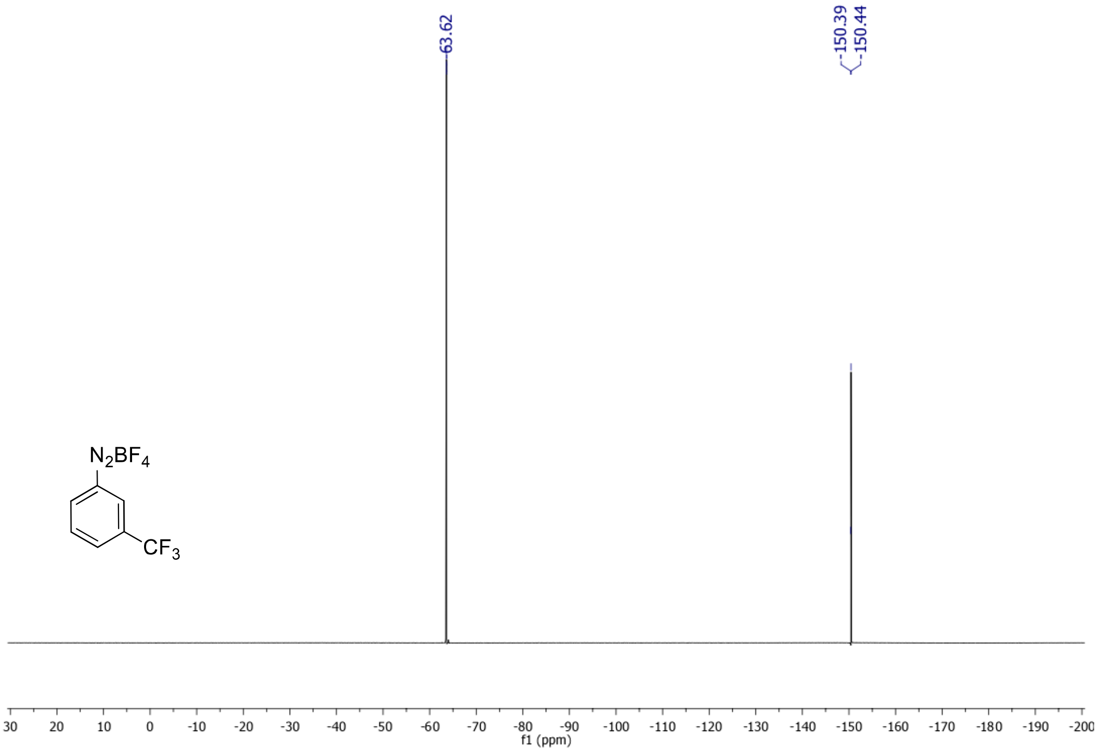

^{19}F NMR (470 MHz, D_2O) of Compound **49**.

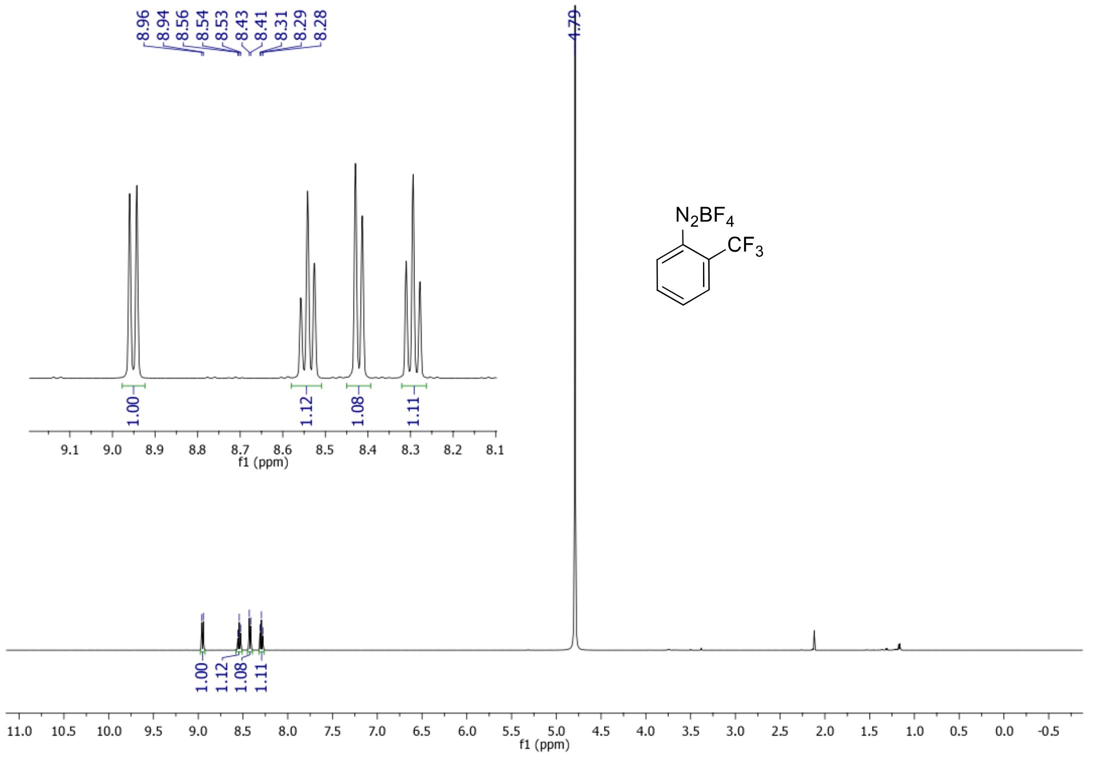

^{1}H NMR (500 MHz, D_2O) of compound **50**.

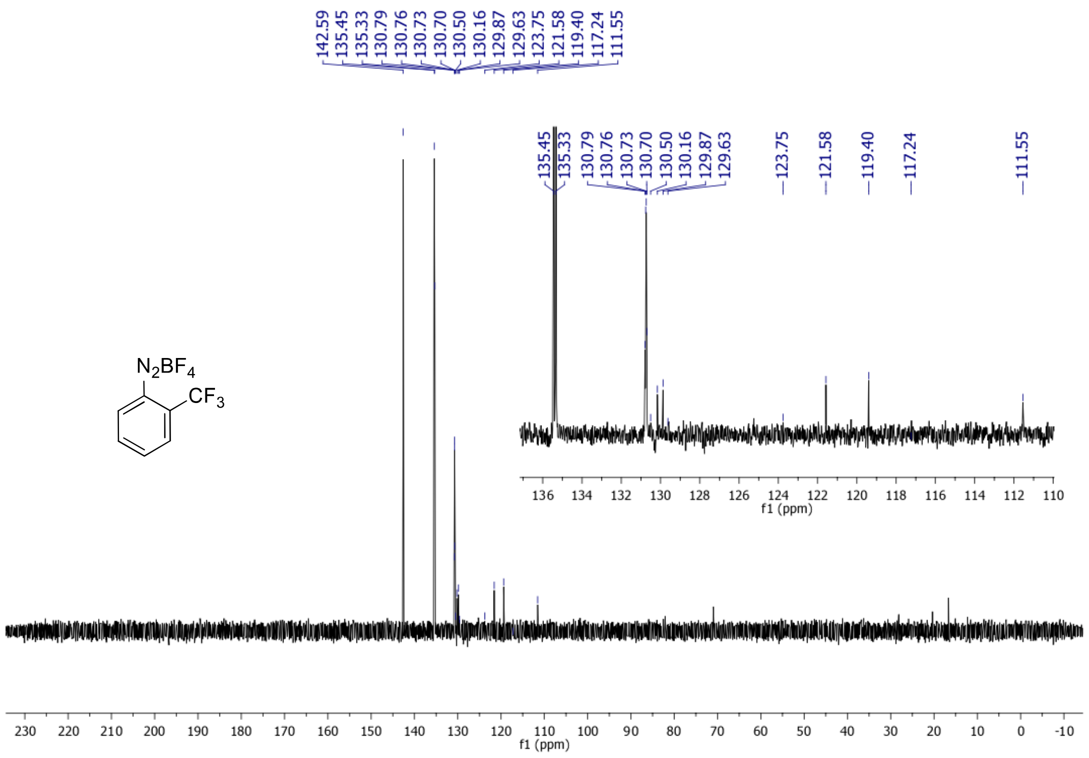

^{13}C NMR (126 MHz, D_2O) of compound **50**.

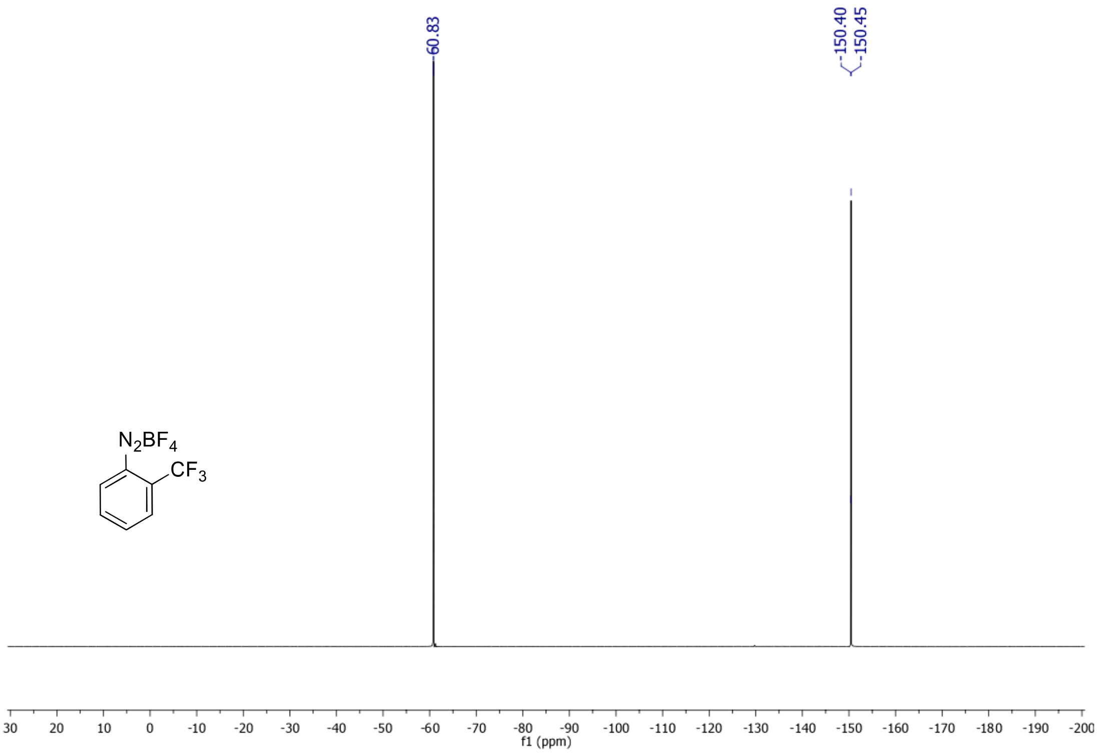

^{19}F NMR (470 MHz, D_2O) of Compound **50**.

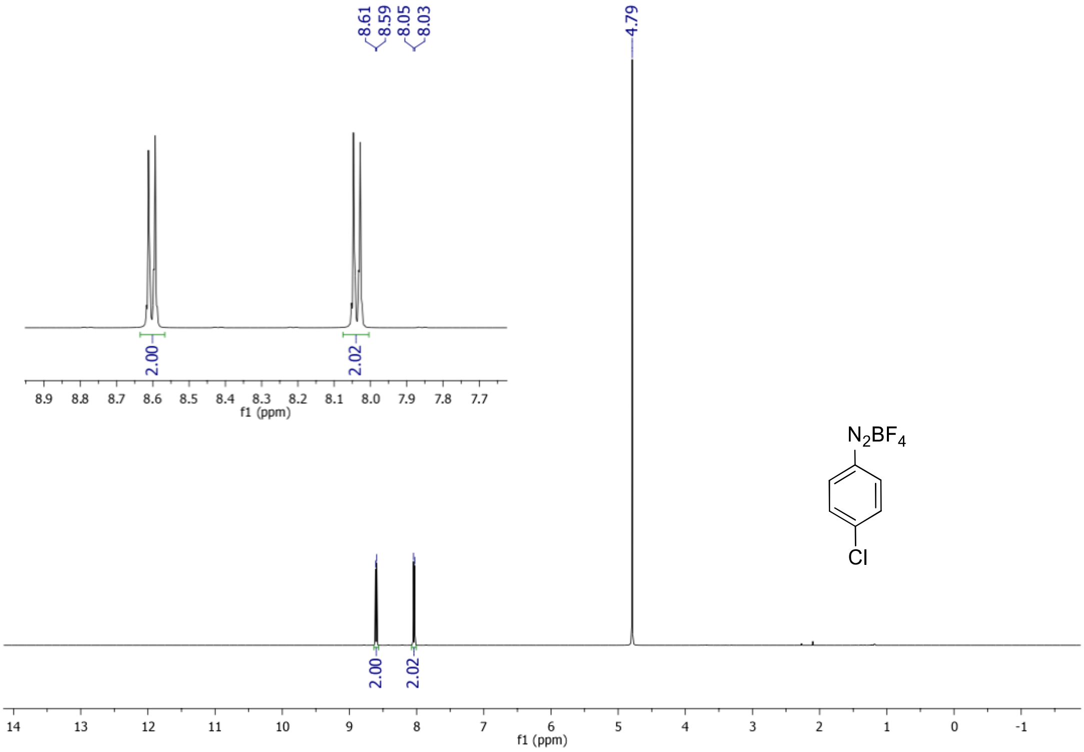

^{1}H NMR (500 MHz, D_2O) of compound **41**.

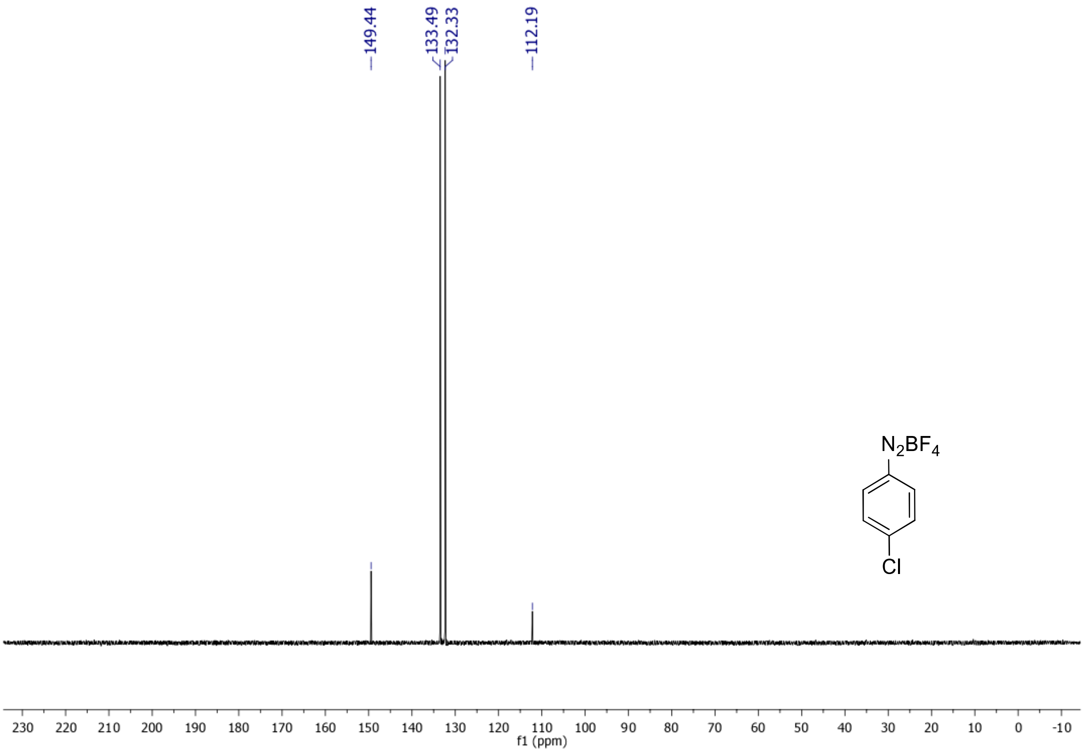

^{13}C NMR (126 MHz, D_2O) of compound **41**.

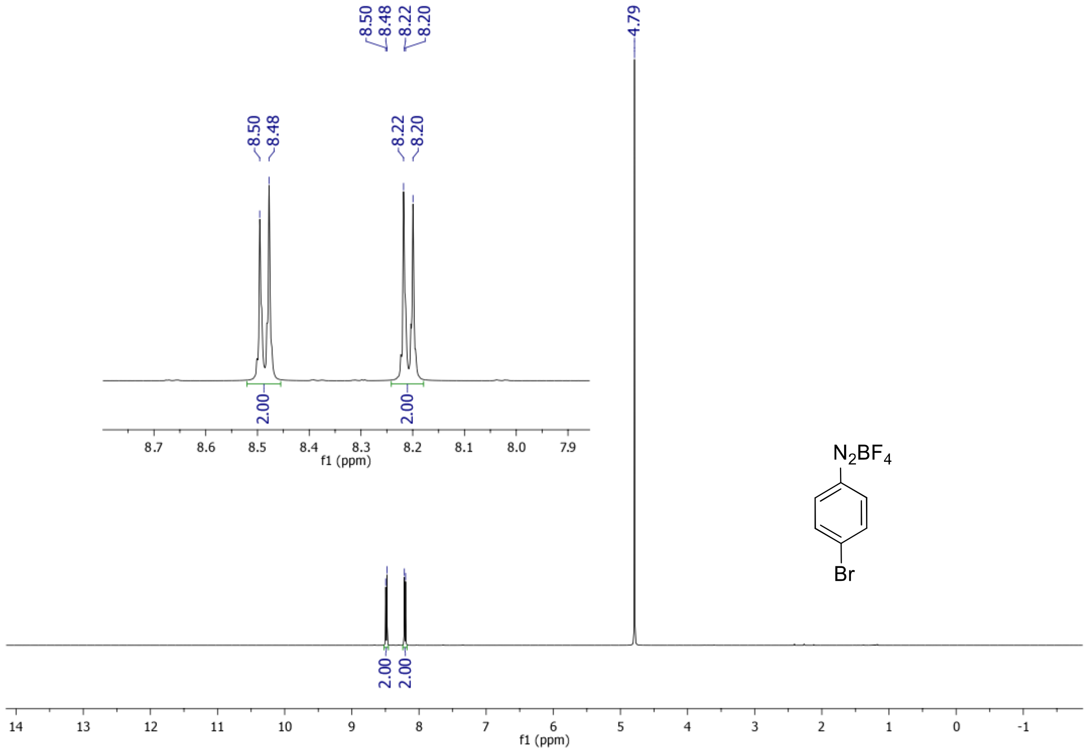

^{1}H NMR (500 MHz, D_2O) of compound **51**.

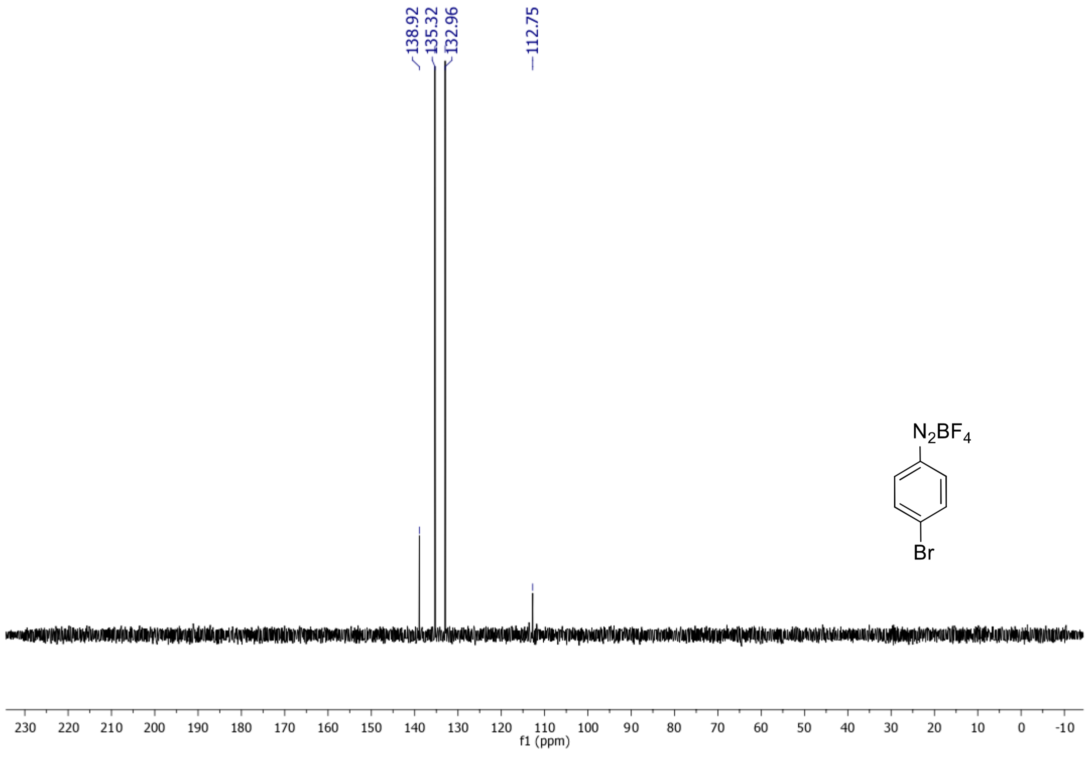

^{13}C NMR (126 MHz, D_2O) of compound **51**.

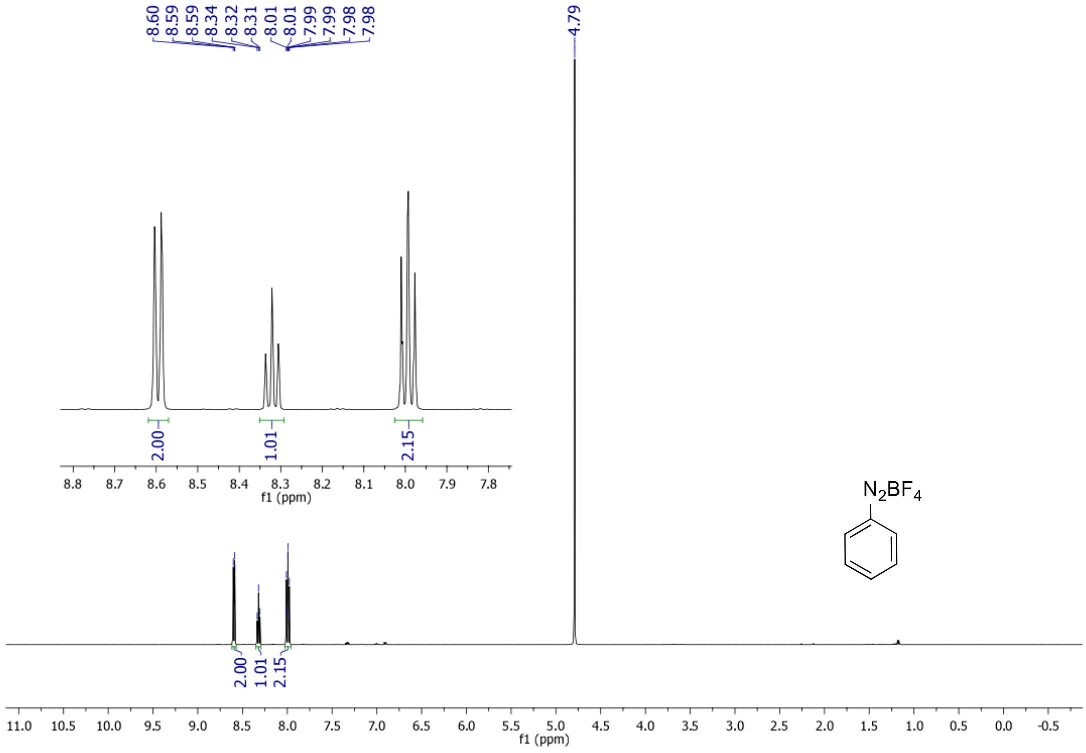

^{1}H NMR (500 MHz, D_2O) of compound **52**.

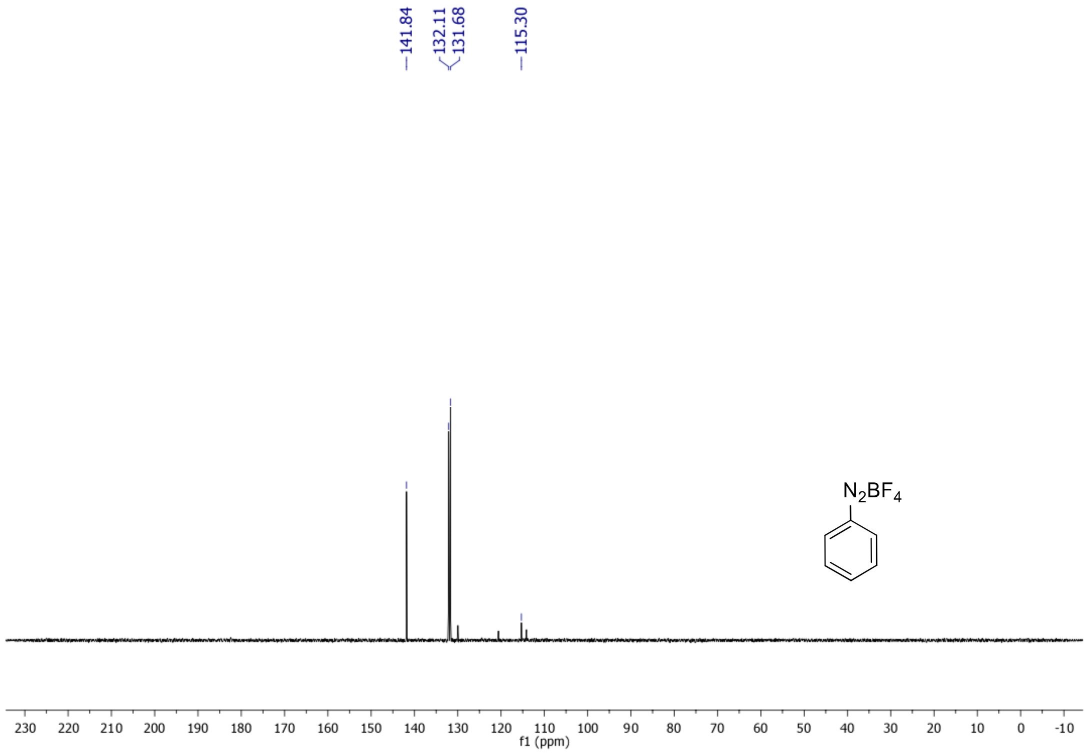

^{13}C NMR (126 MHz, D_2O) of compound **52**.

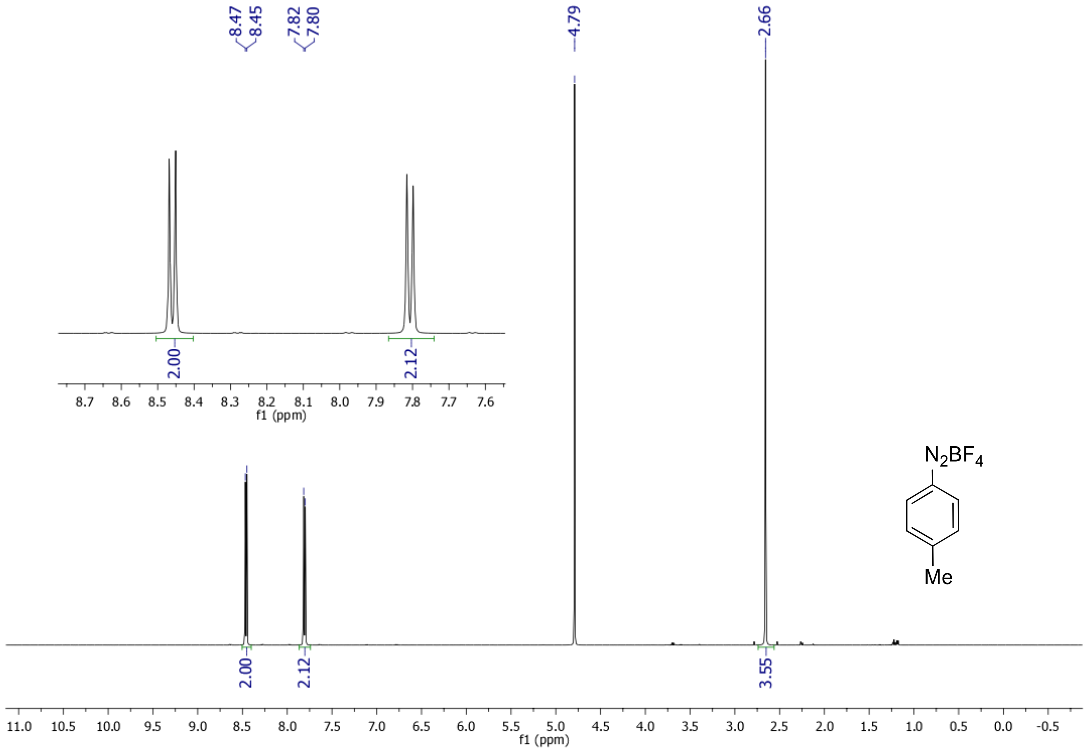

^{1}H NMR (500 MHz, D_2O) of compound **53**.

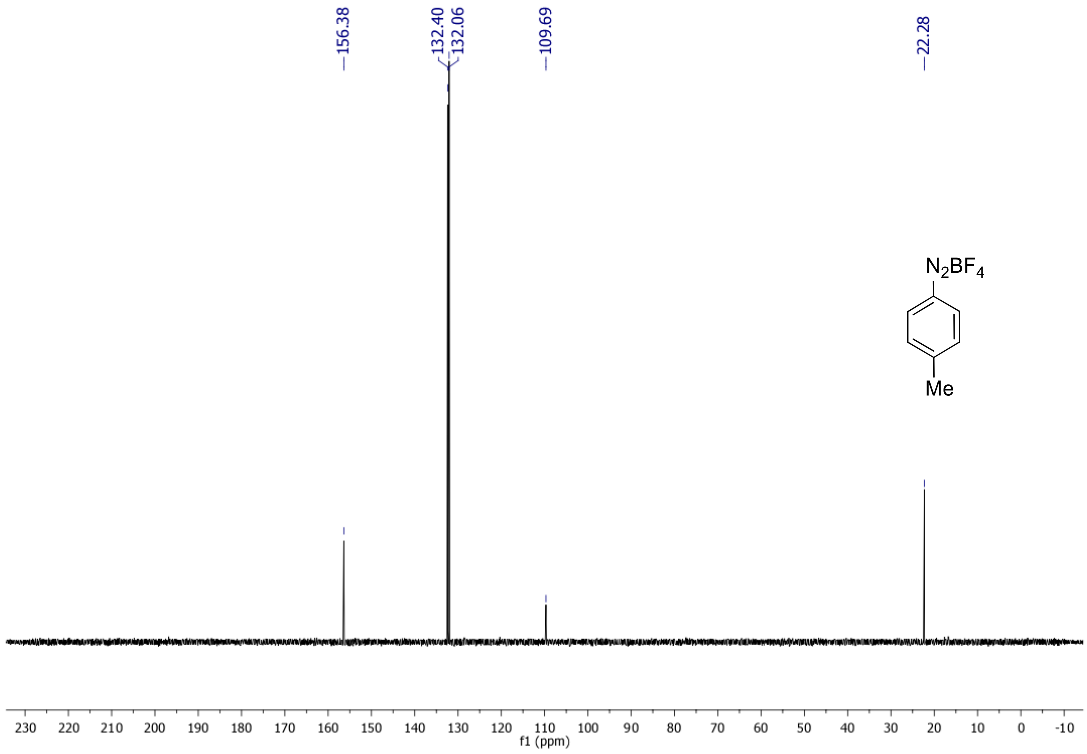

^{13}C NMR (126 MHz, D_2O) of compound **53**.

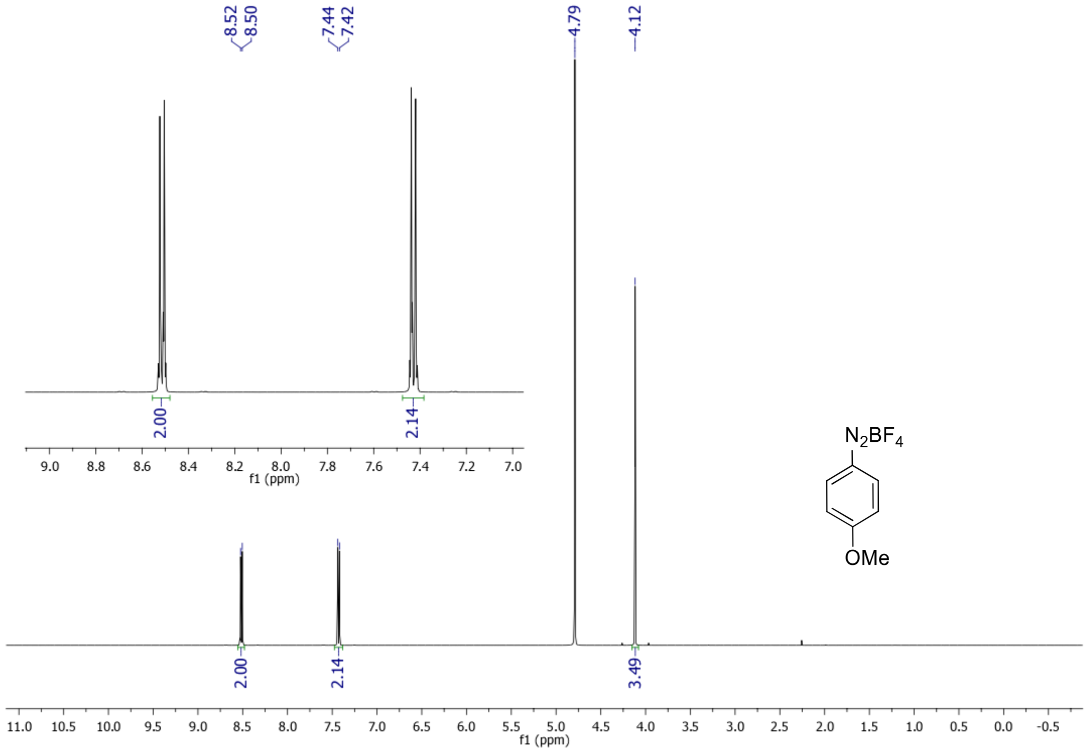

^{1}H NMR (500 MHz, D_2O) of compound **54**.

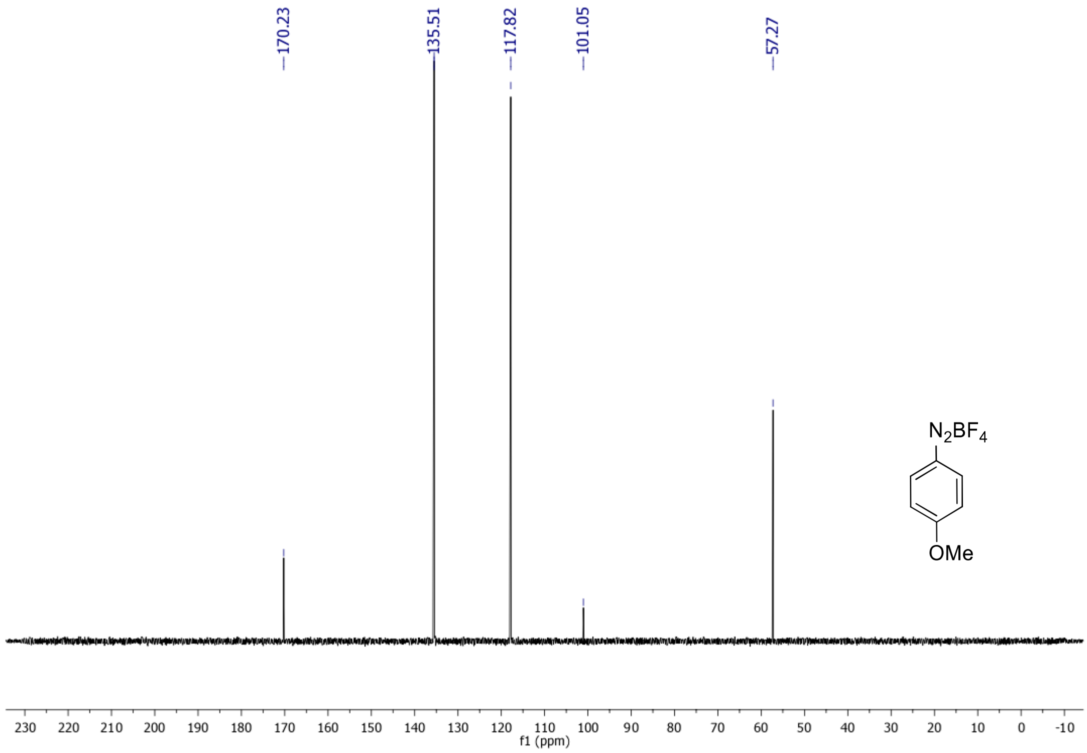

^{13}C NMR (126 MHz, D_2O) of compound **54**.

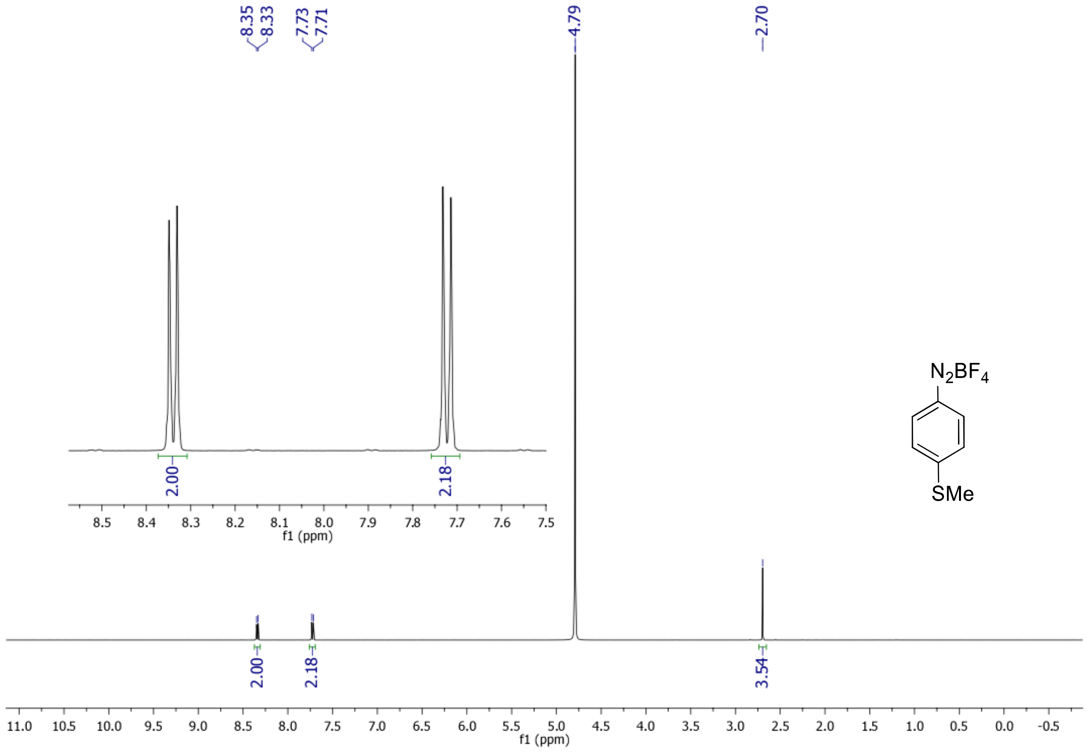

^{1}H NMR (500 MHz, D_2O) of compound **55**.

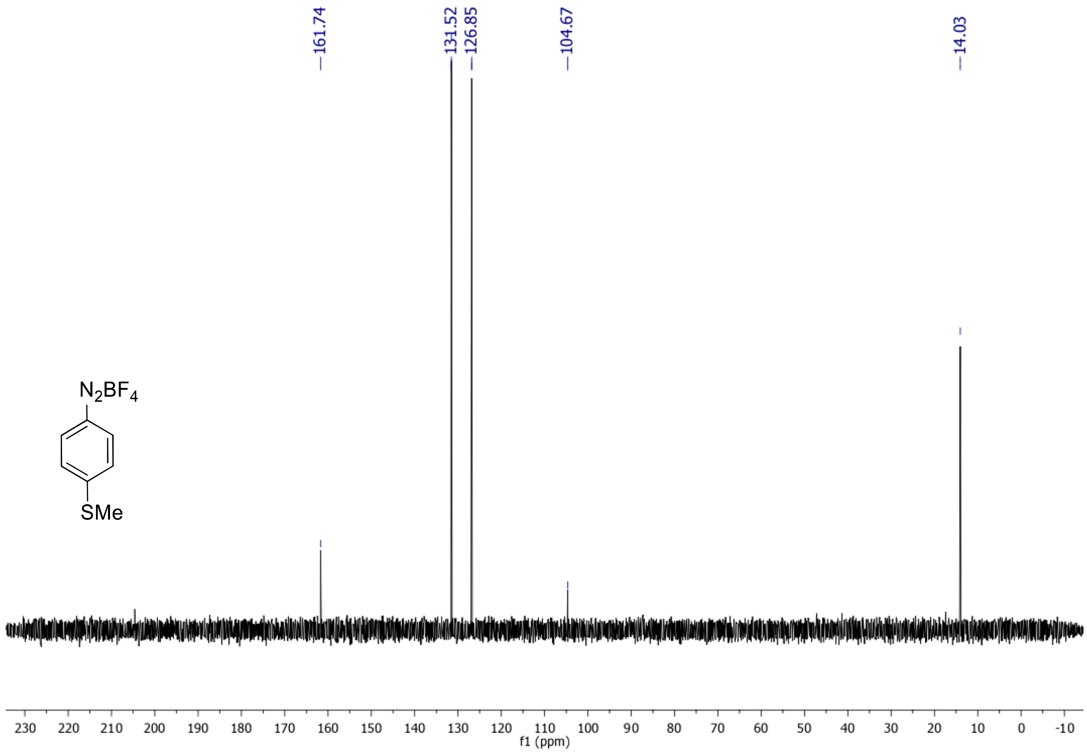

^{13}C NMR (126 MHz, D_2O) of compound **55**.

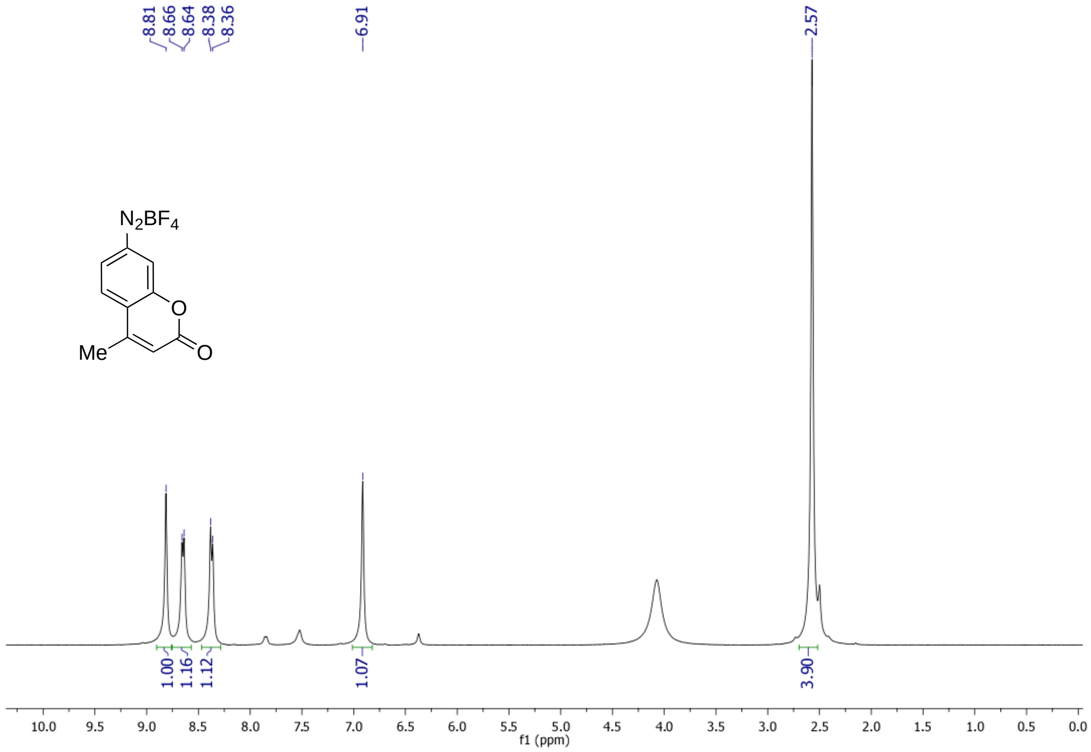

^{1}H NMR (400 MHz, DMSO) of compound **56**.

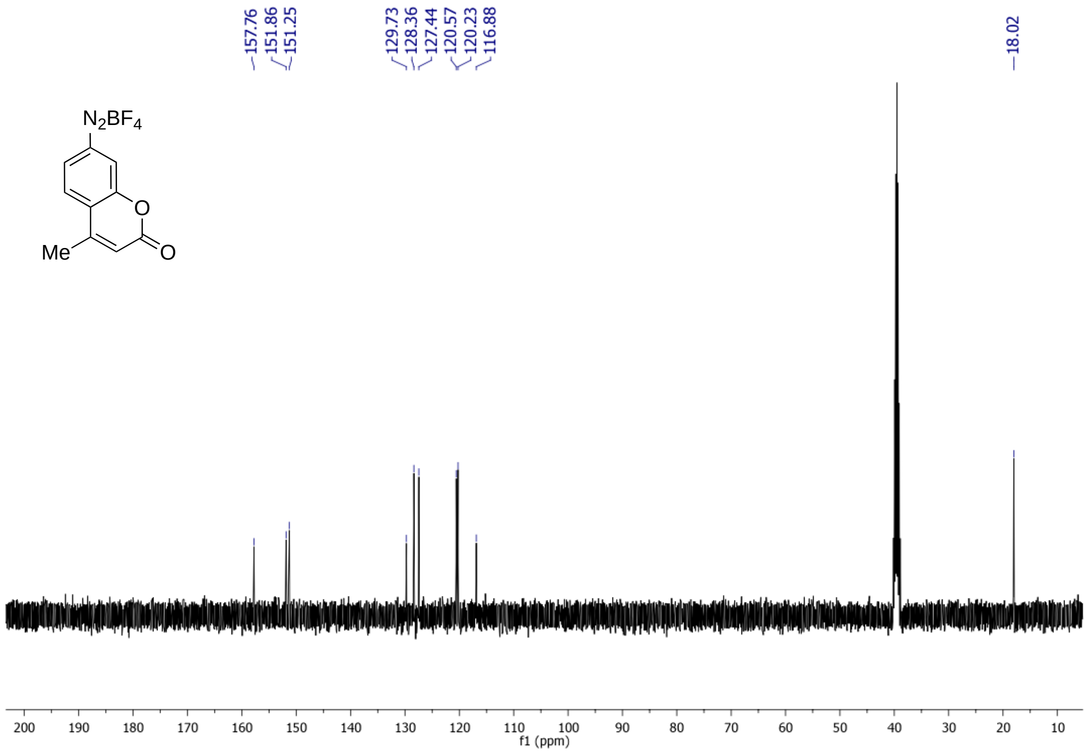

^{13}C NMR (101 MHz, DMSO) of compound **56**.

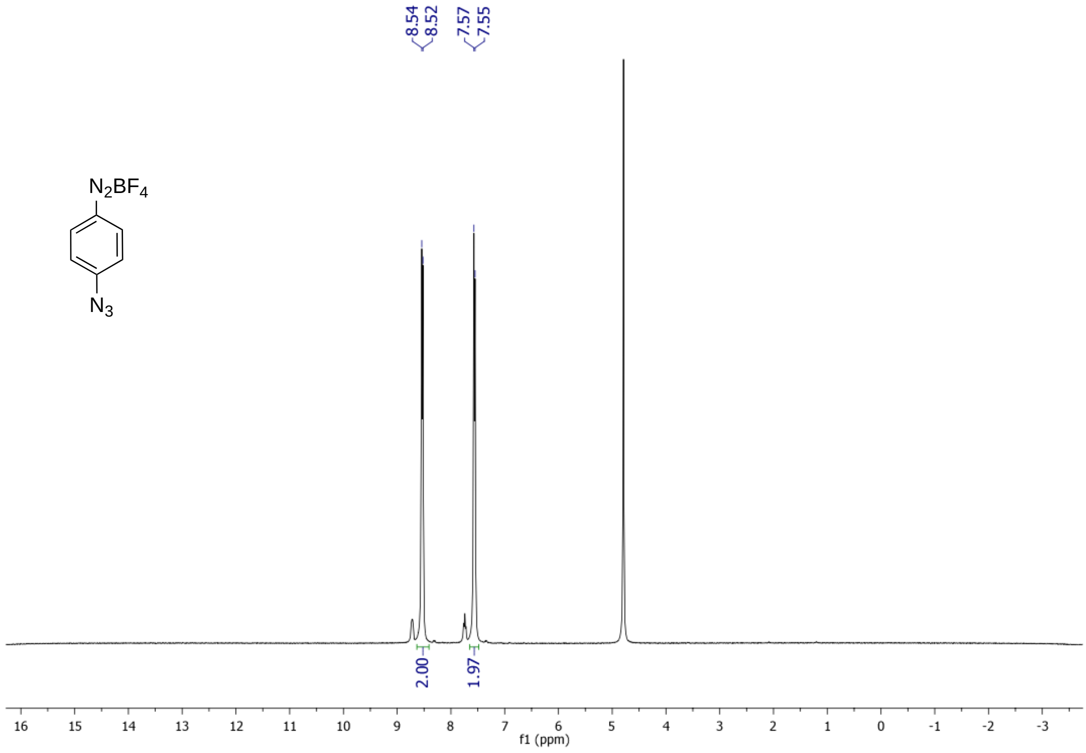

^{1}H NMR (400 MHz, D_2O) of compound **57**.

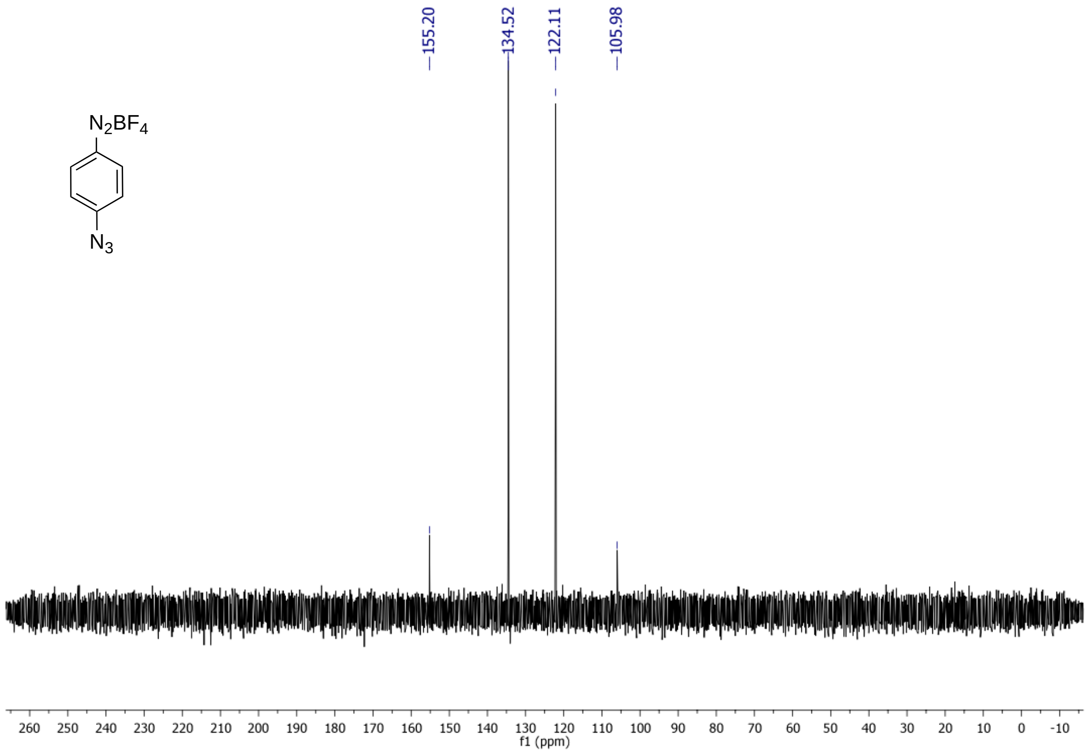

^{13}C NMR (101 MHz, D_2O) of compound **57**.

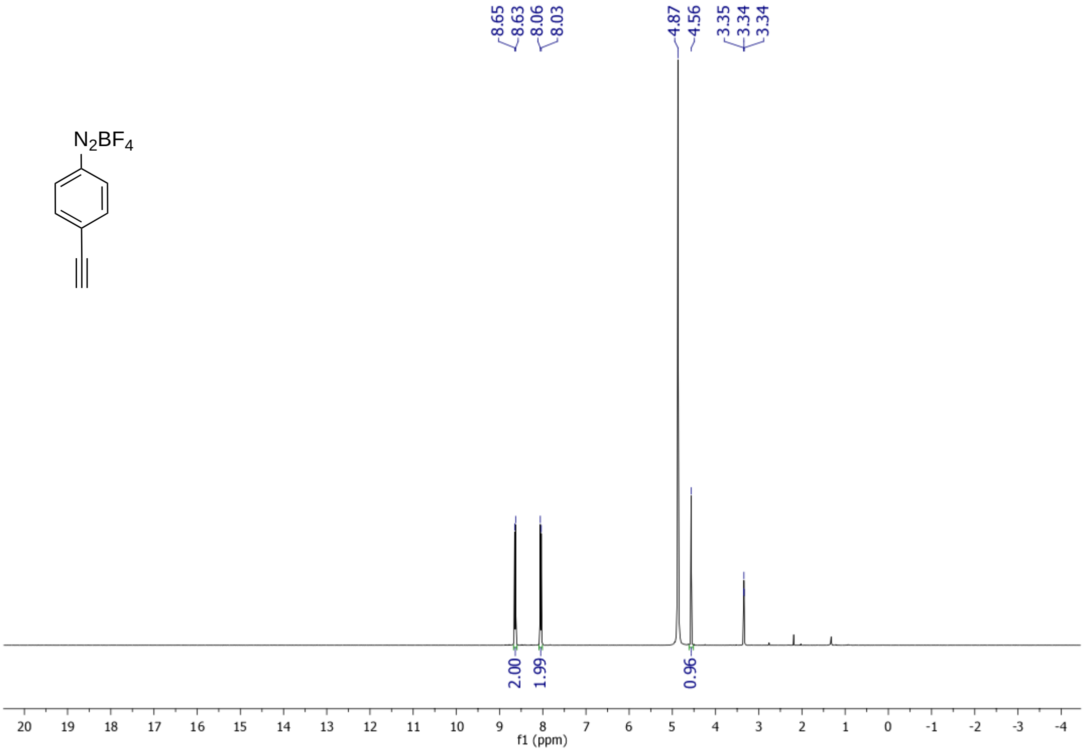

^{1}H NMR (400 MHz, MeOD) of compound **58**.

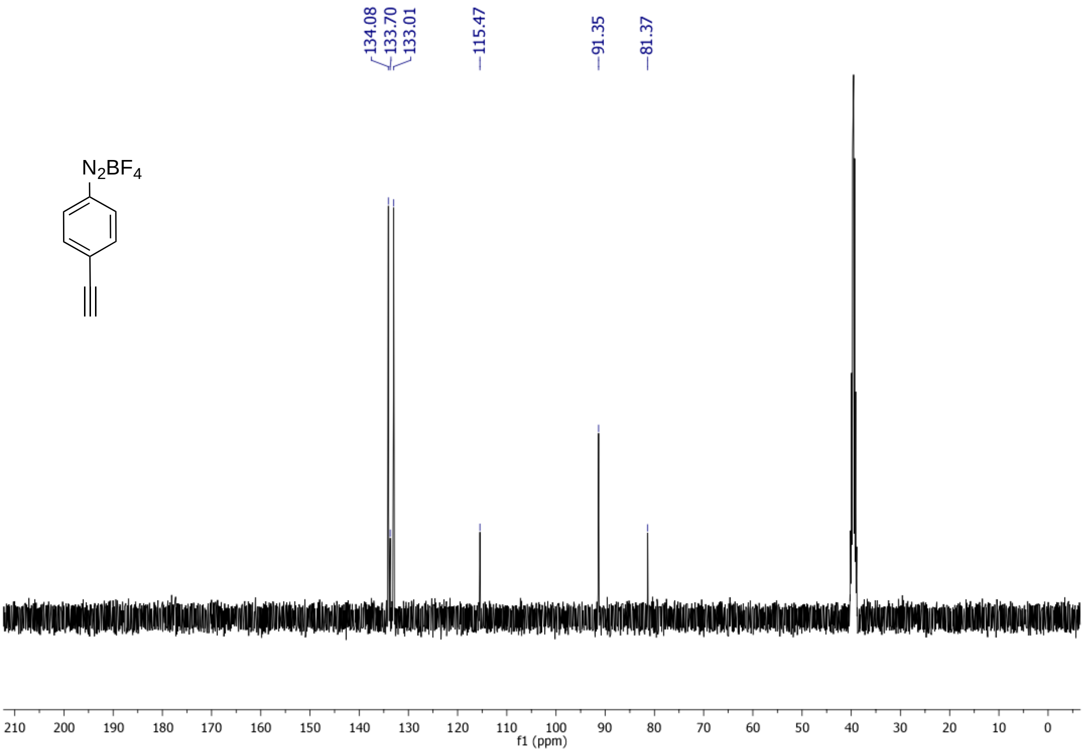

^{13}C NMR (101 MHz, DMSO) of compound **58**.

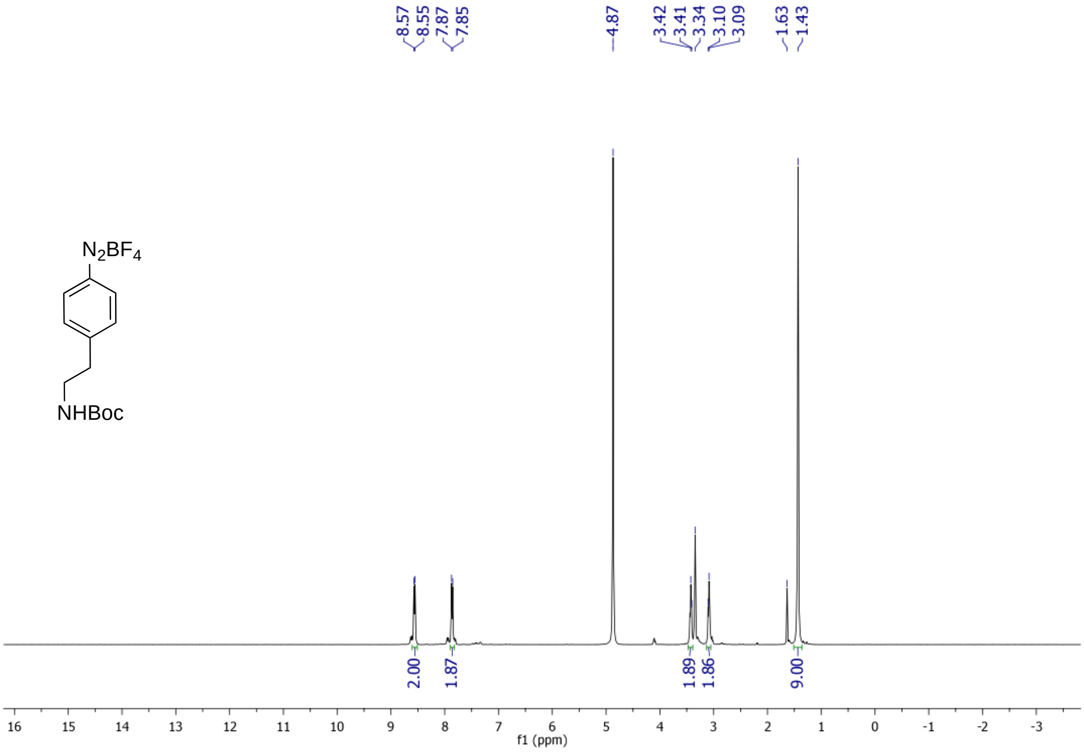

^{1}H NMR (400 MHz, MeOD) of compound **59**.

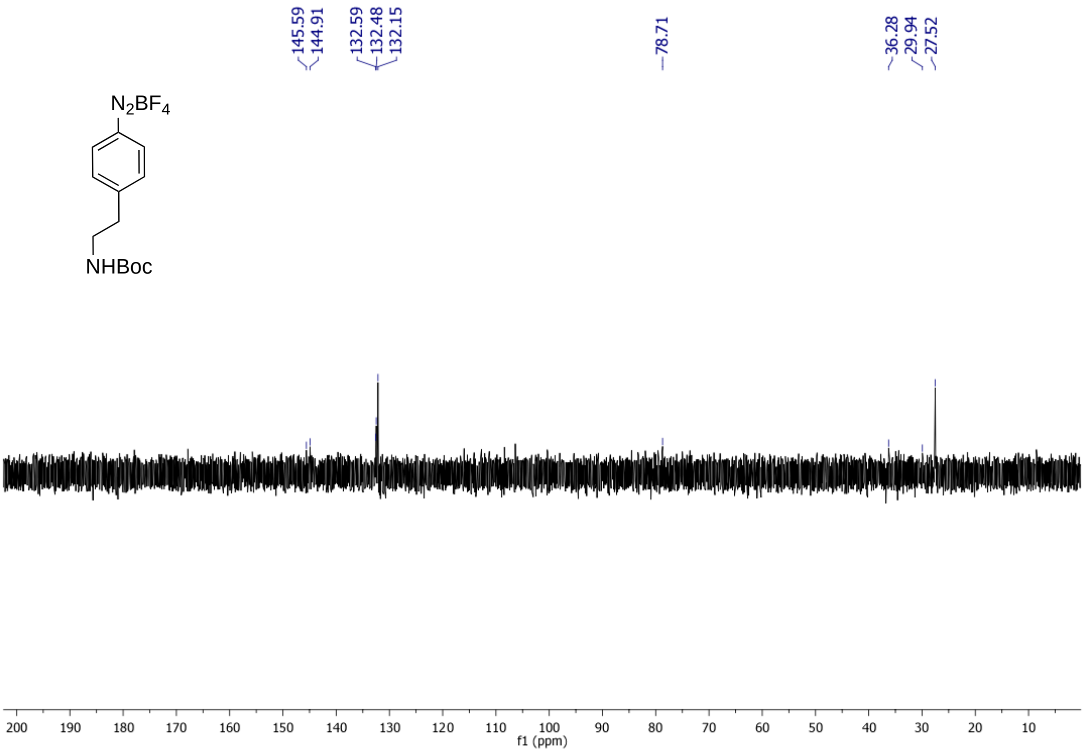

^{13}C NMR (101 MHz, D_2O) of compound **59**.

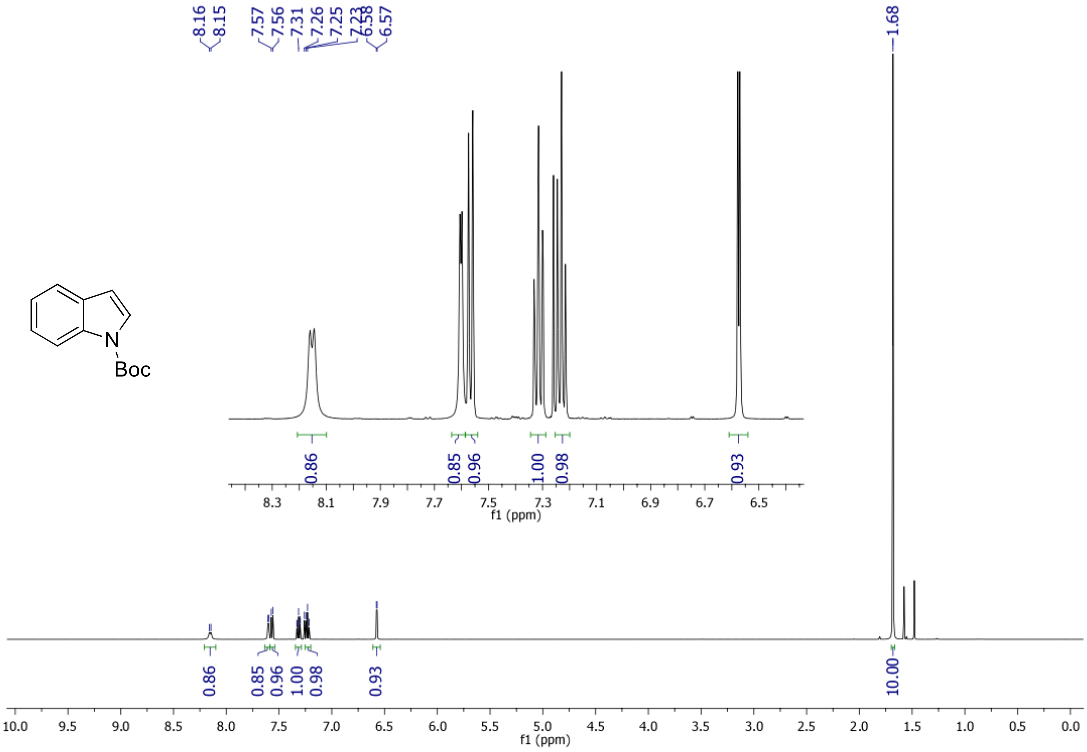

^{1}H NMR (500 MHz, $CDCl_3$) of compound **1**.

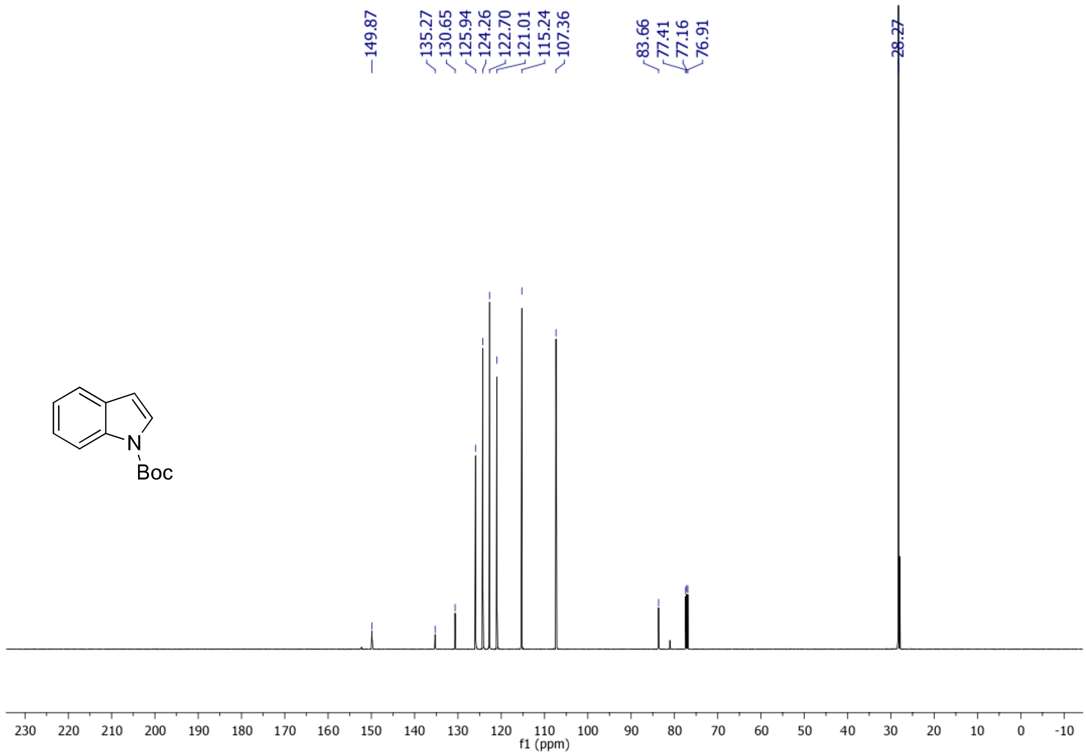

^{13}C NMR (126 MHz, $CDCl_3$) of compound **1**.

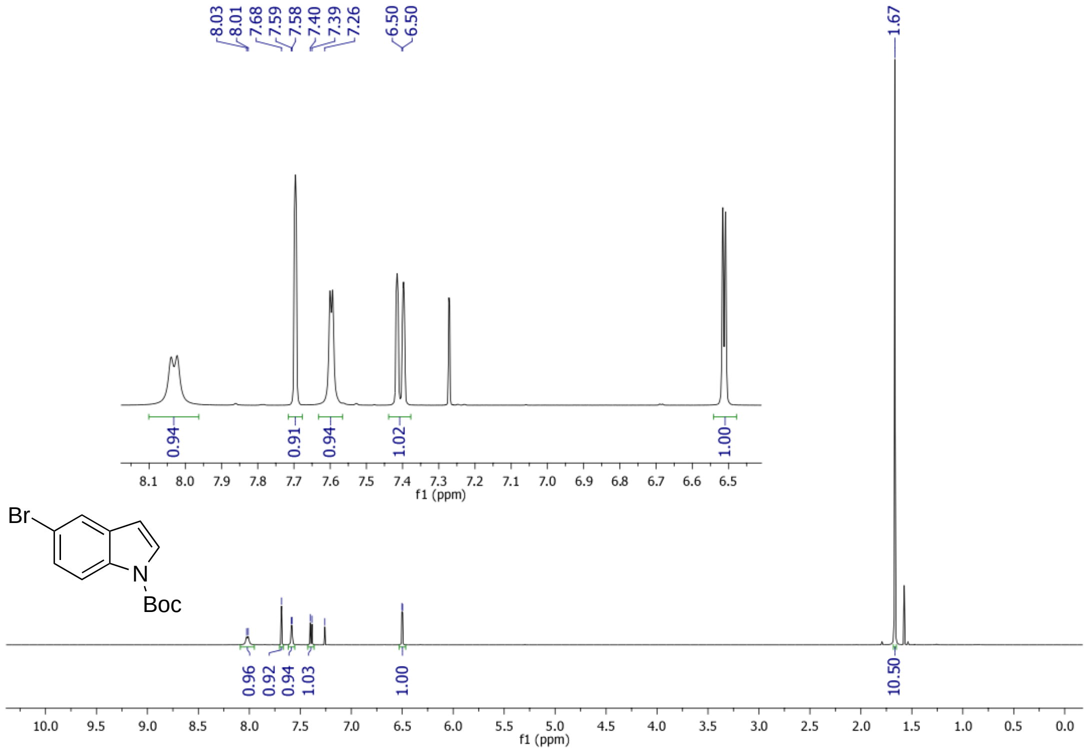

^{1}H NMR (500 MHz, CDCl$_3$) of compound **60**.

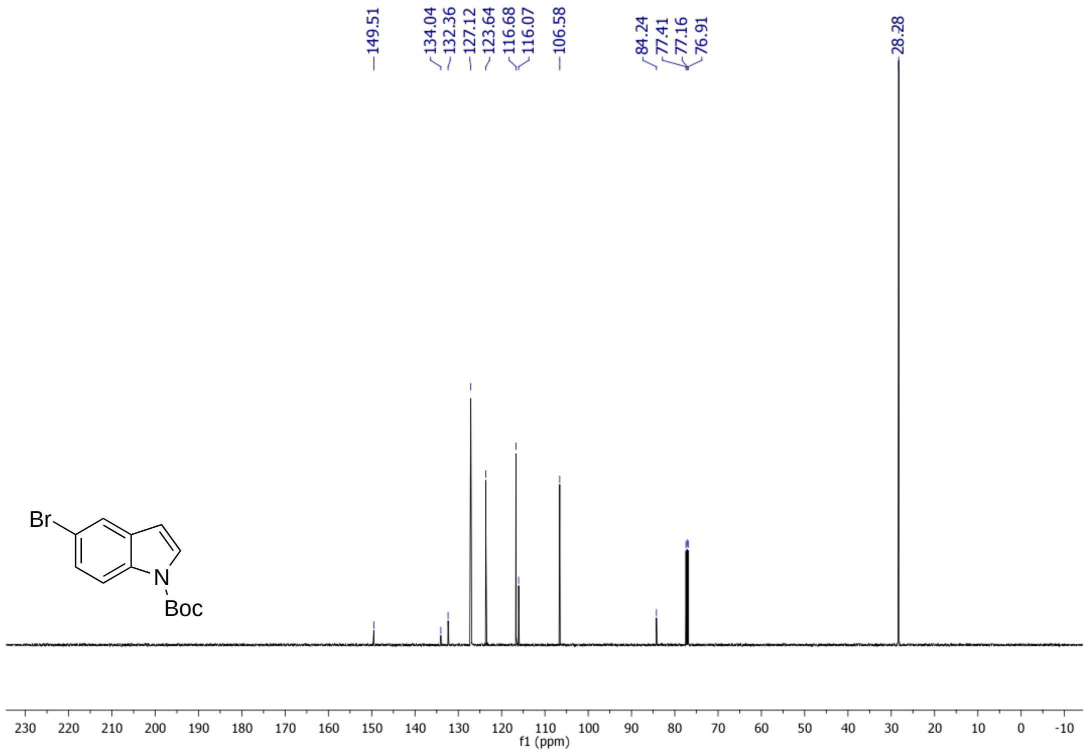

^{13}C NMR (126 MHz, CDCl$_3$) of compound **60**.

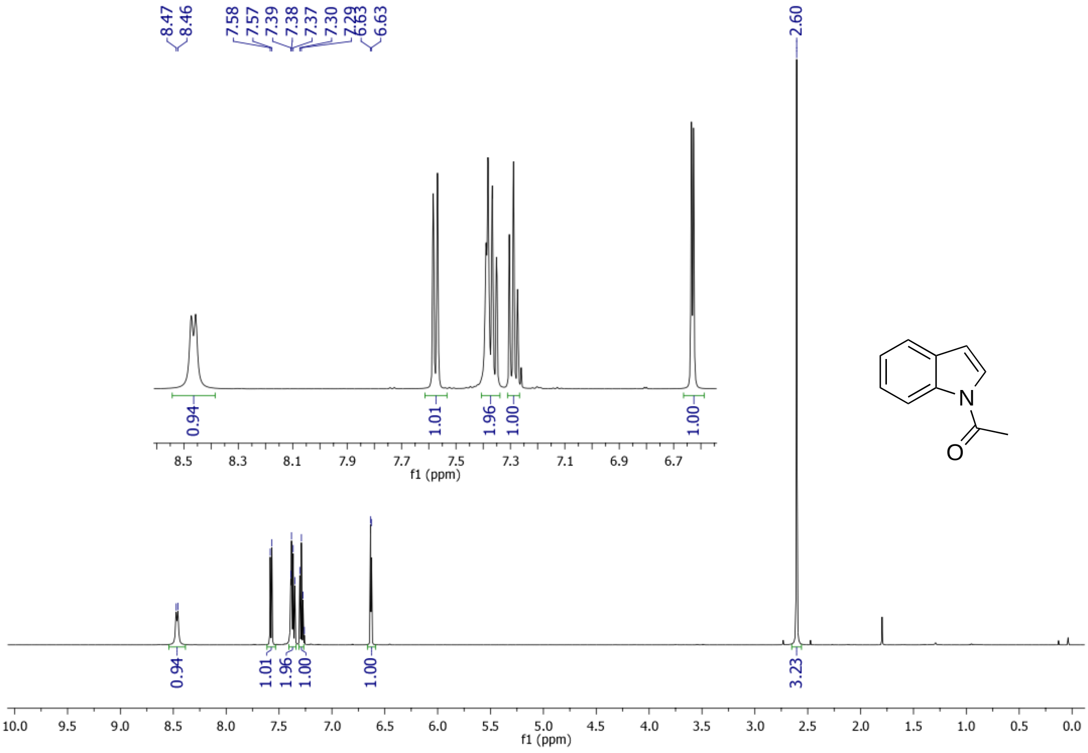

^{1}H NMR (500 MHz, CDCl$_3$) of compound **61**.

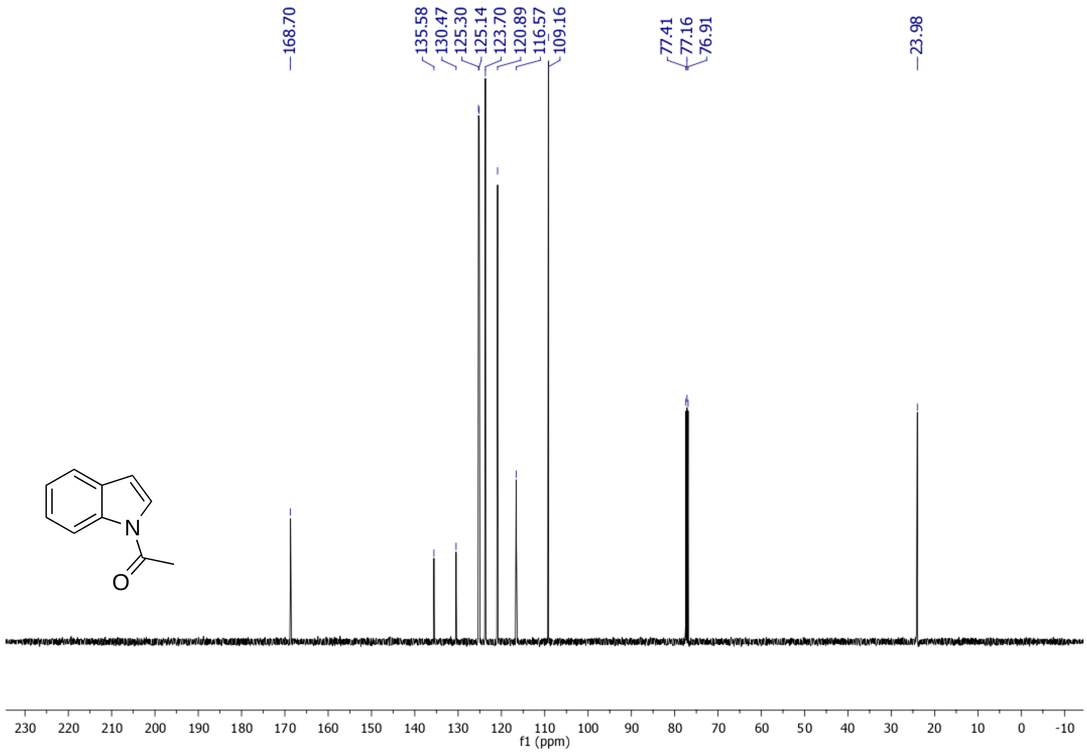

^{13}C NMR (126 MHz, CDCl$_3$) of compound **61**.

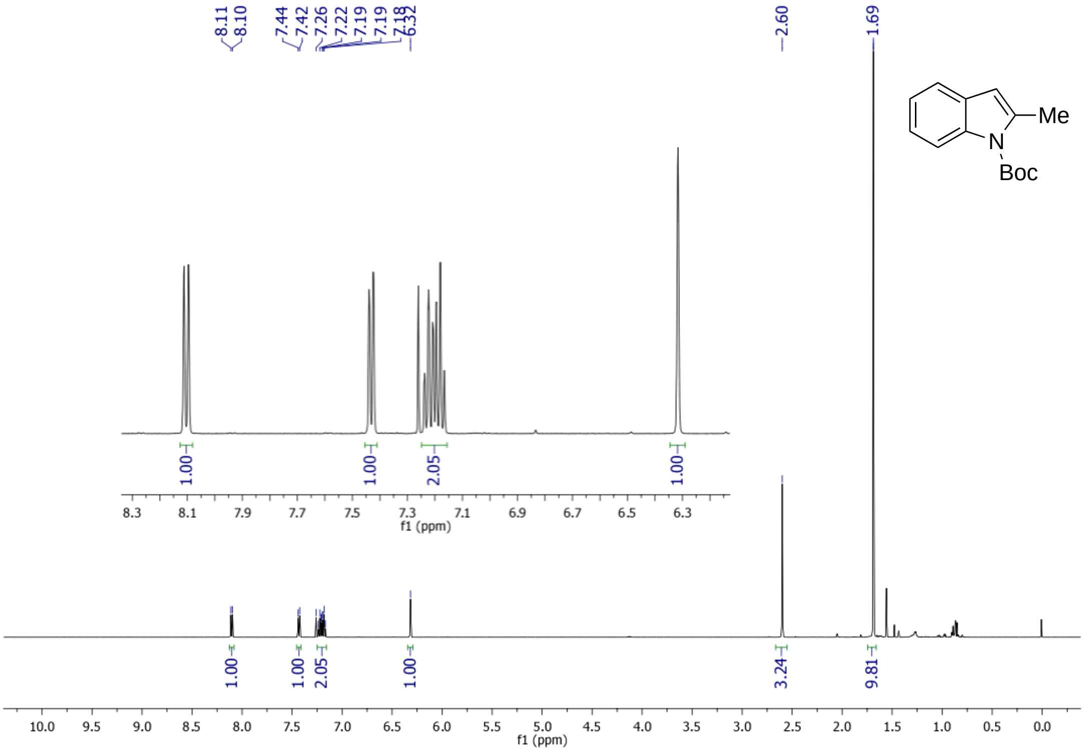

^{1}H NMR (500 MHz, CDCl₃) of compound **62**.

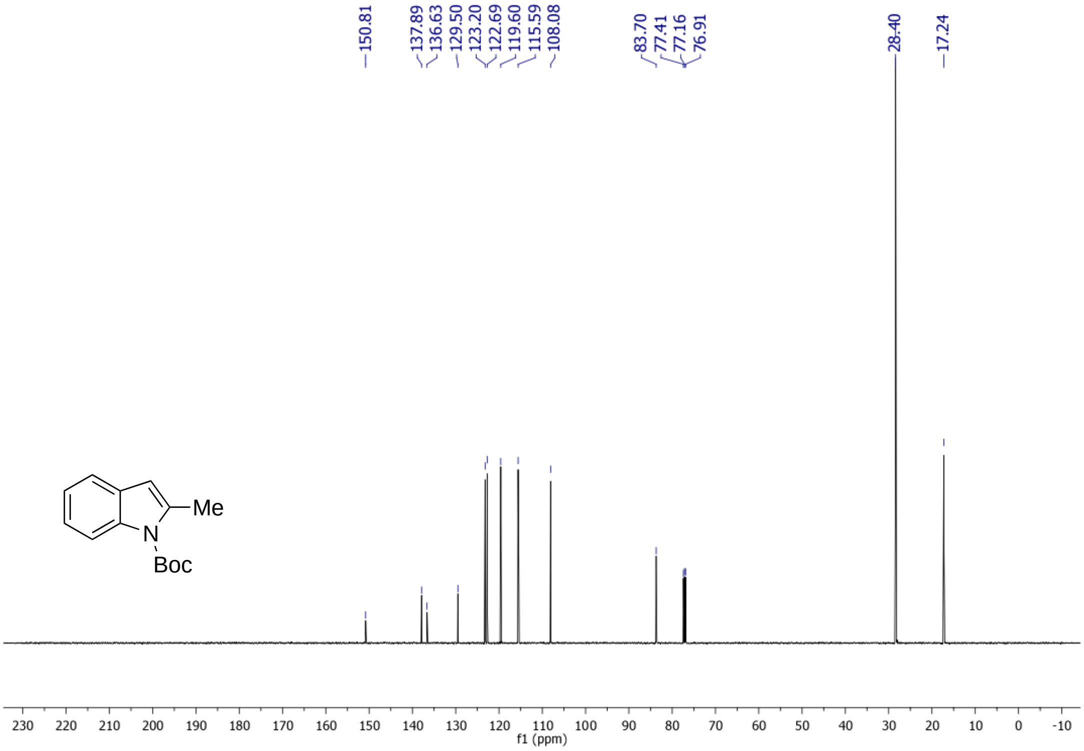

^{13}C NMR (126 MHz, CDCl₃) of compound **62**.

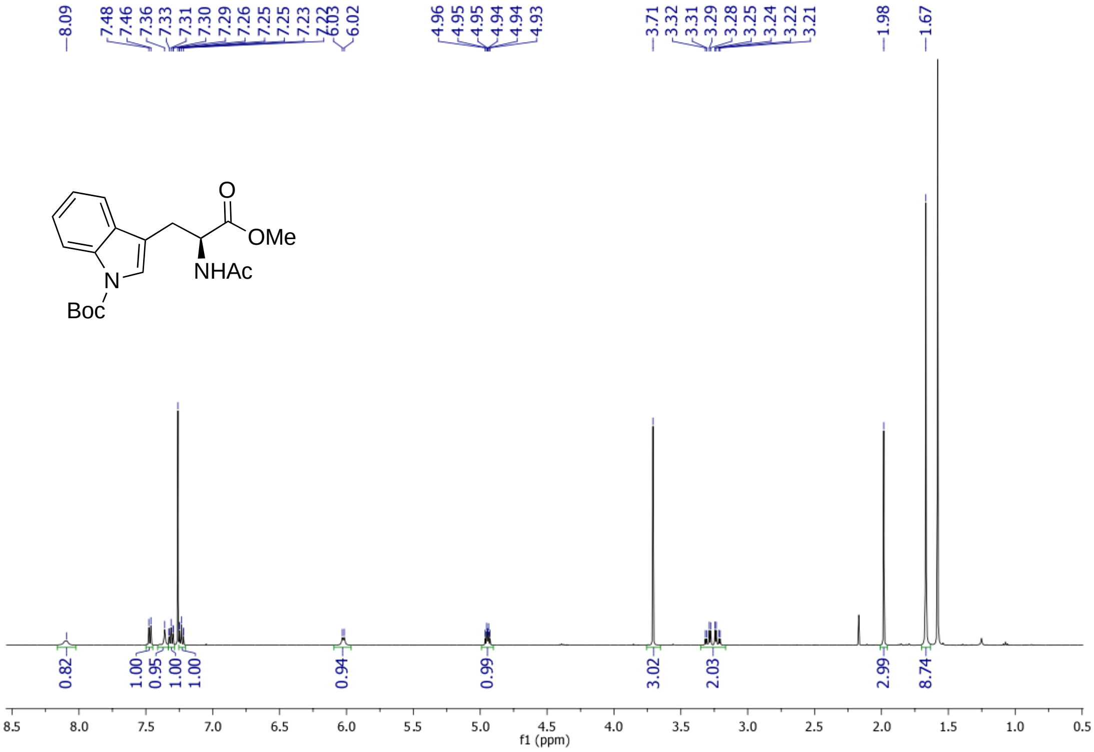

^{1}H NMR (500 MHz, CDCl₃) of compound **63**.

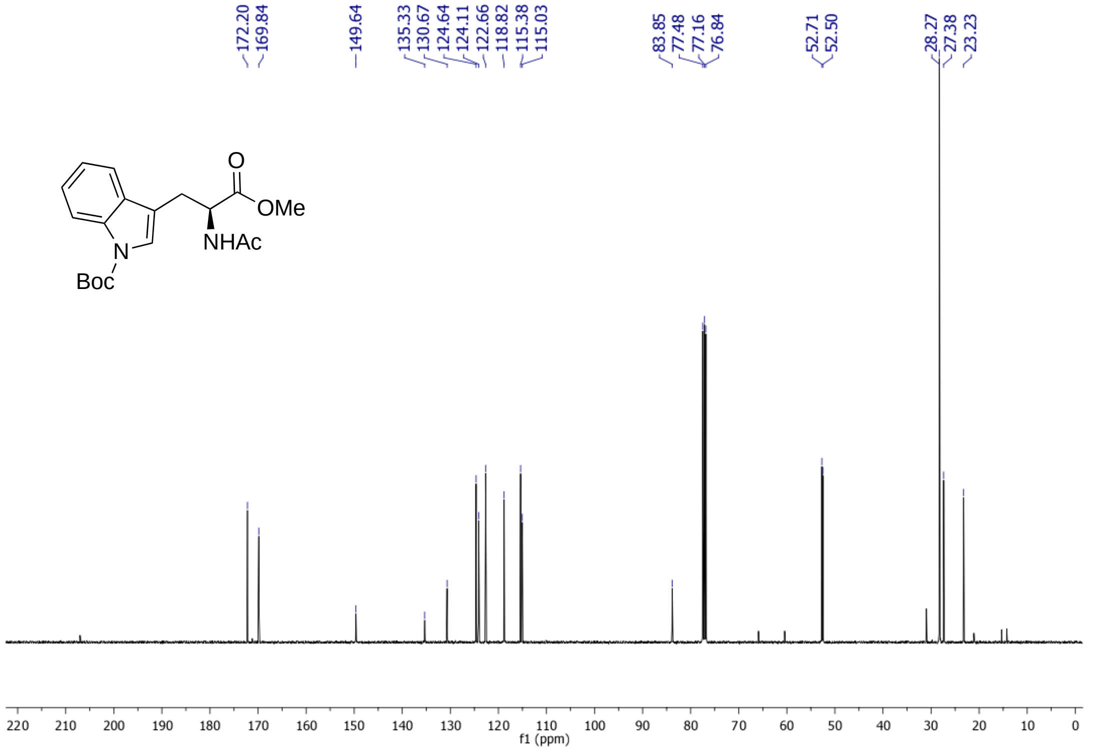

^{13}C NMR (101 MHz, CDCl₃) of compound **63**.

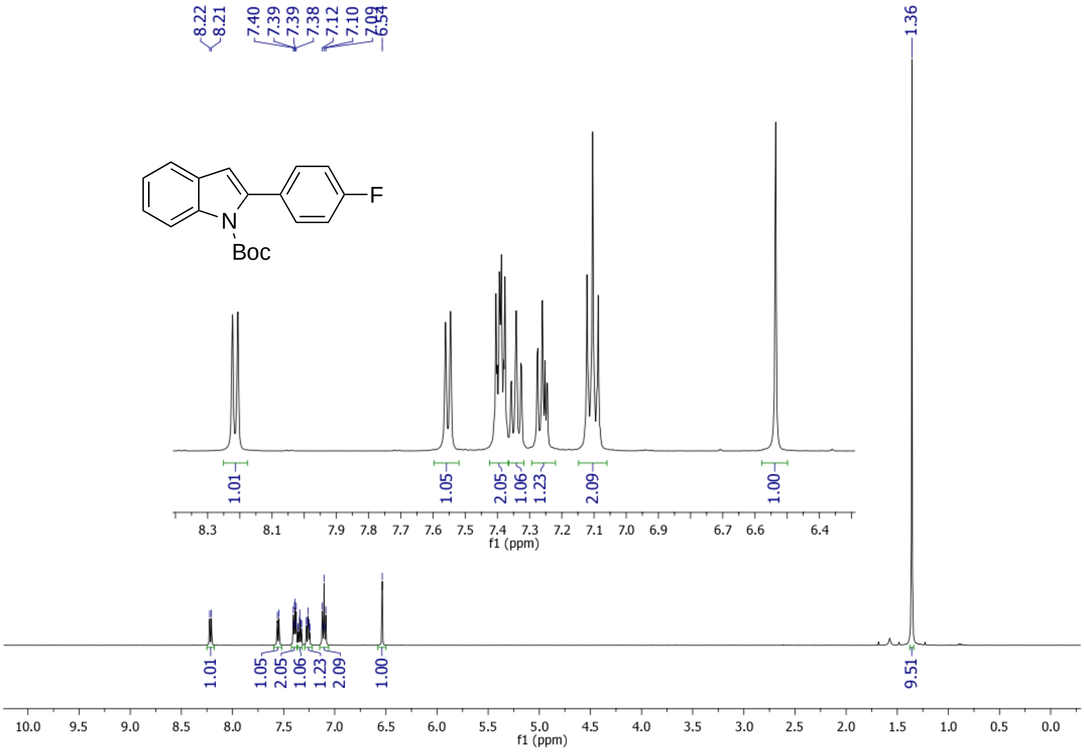

^{1}H NMR (500 MHz, CDCl$_3$) of compound **3**.

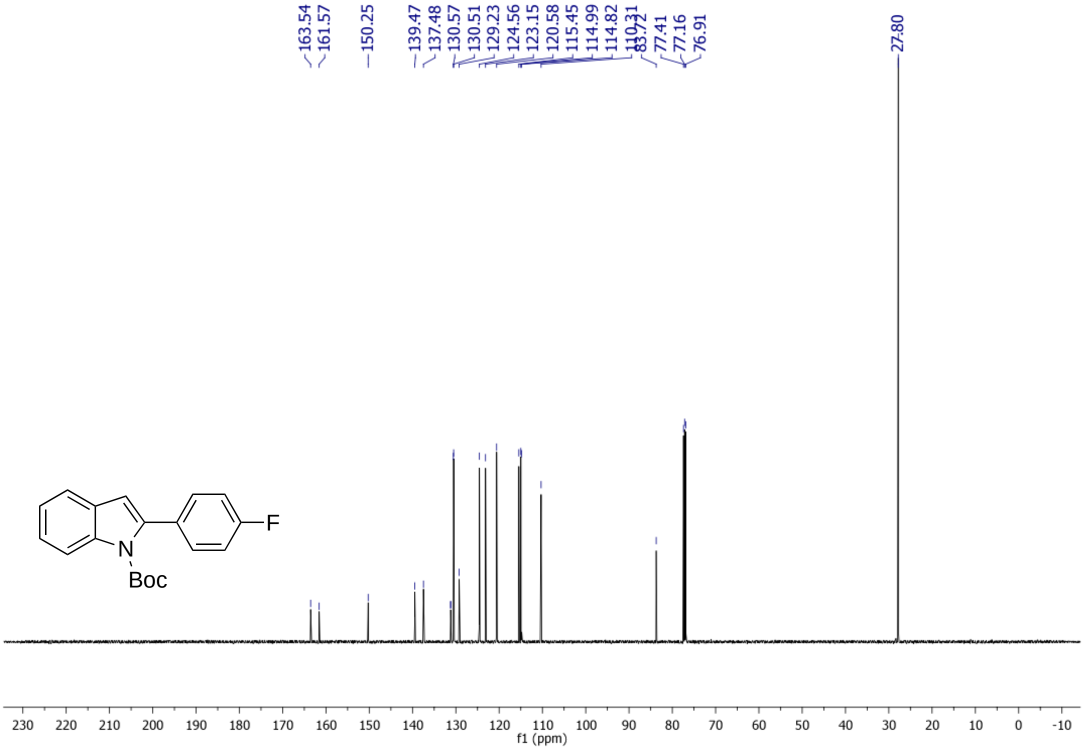

^{13}C NMR (126 MHz, CDCl$_3$) of compound **3**.

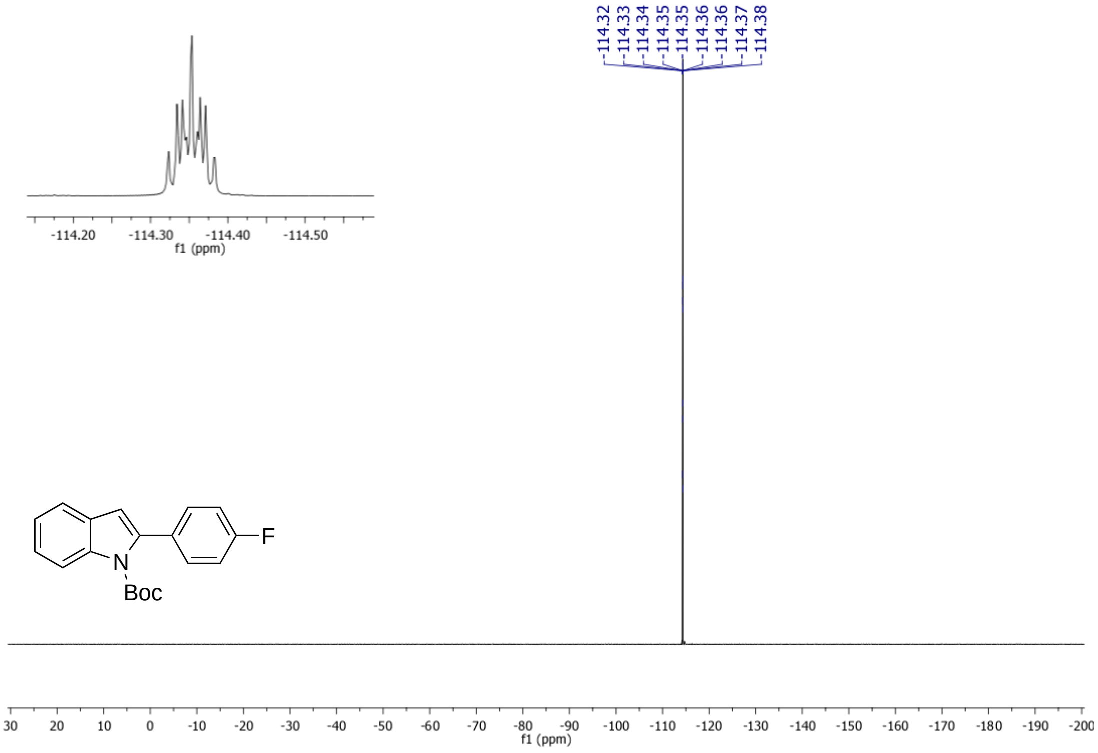

^{19}F NMR (470 MHz, $CDCl_3$) of Compound **3**.

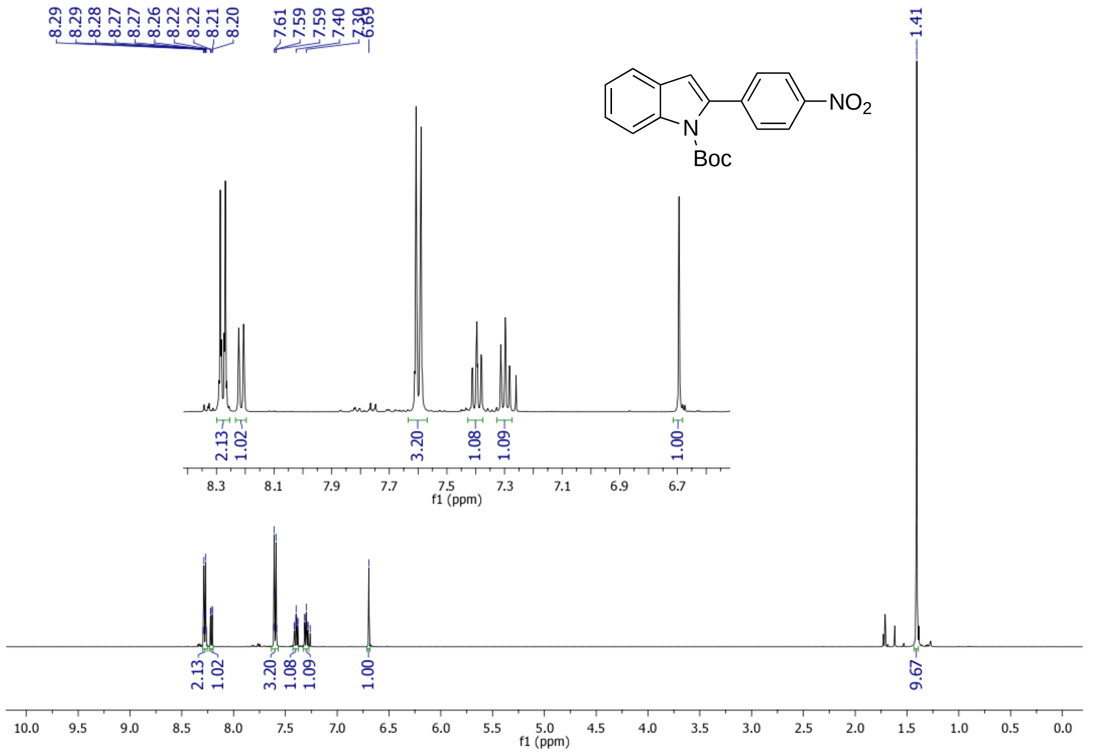

^{1}H NMR (500 MHz, $CDCl_3$) of compound **4**.

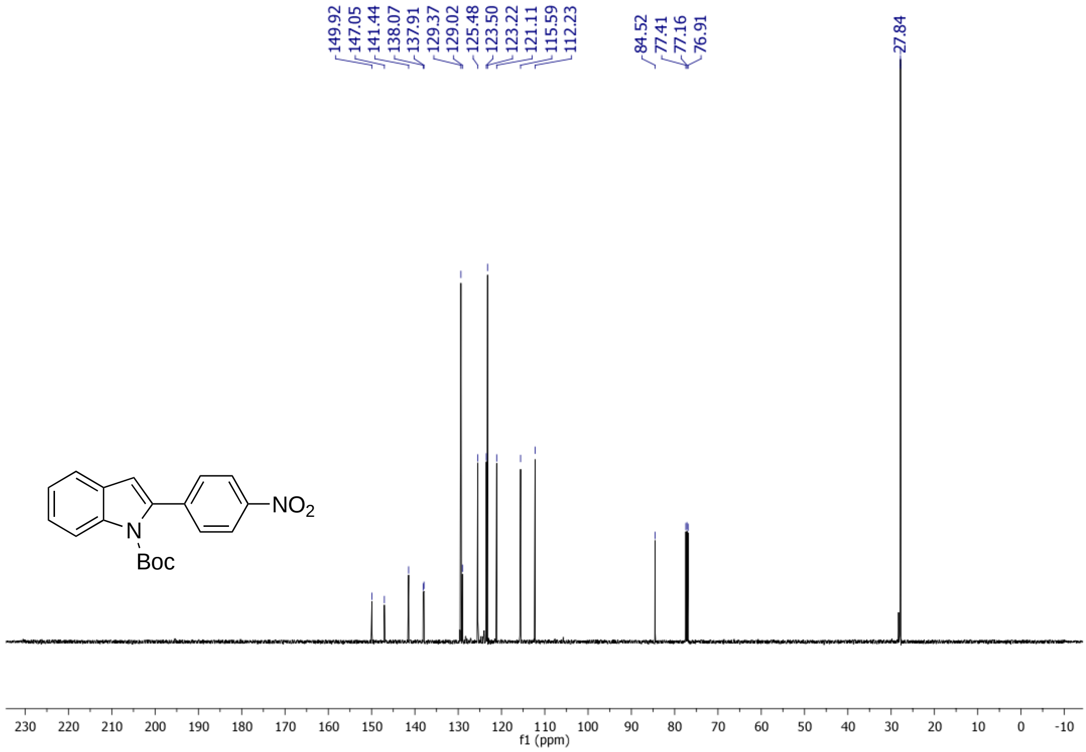

^{13}C NMR (126 MHz, CDCl$_3$) of compound **4**.

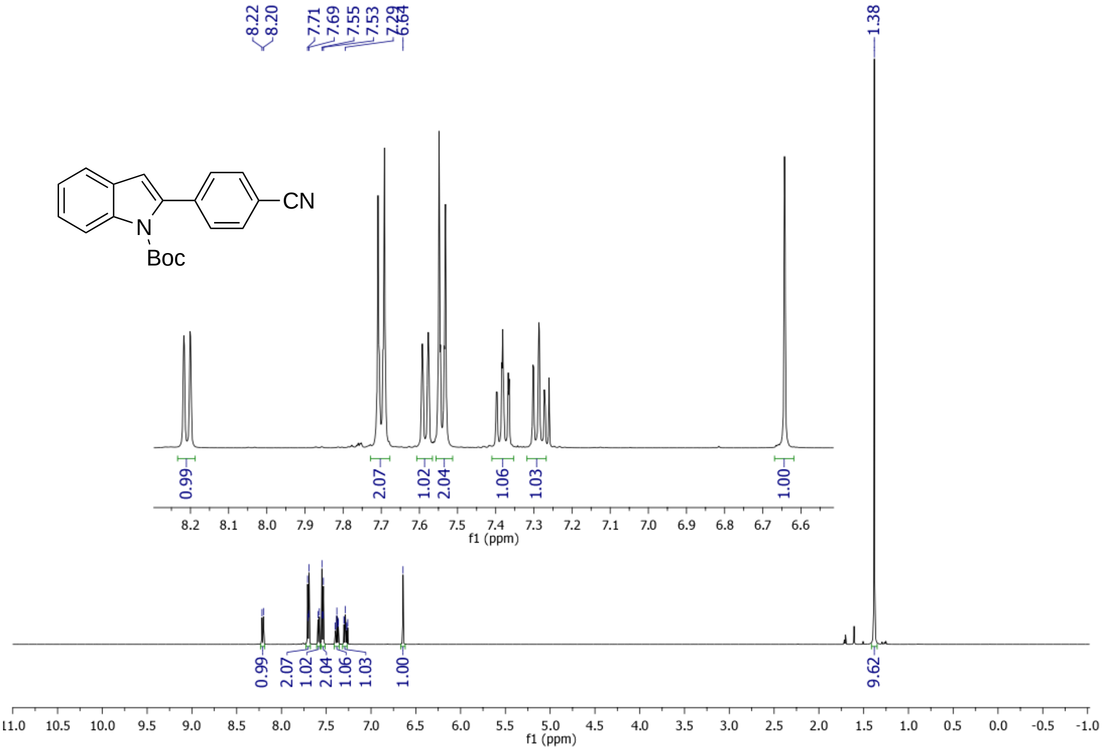

^{1}H NMR (500 MHz, CDCl$_3$) of compound **5**.

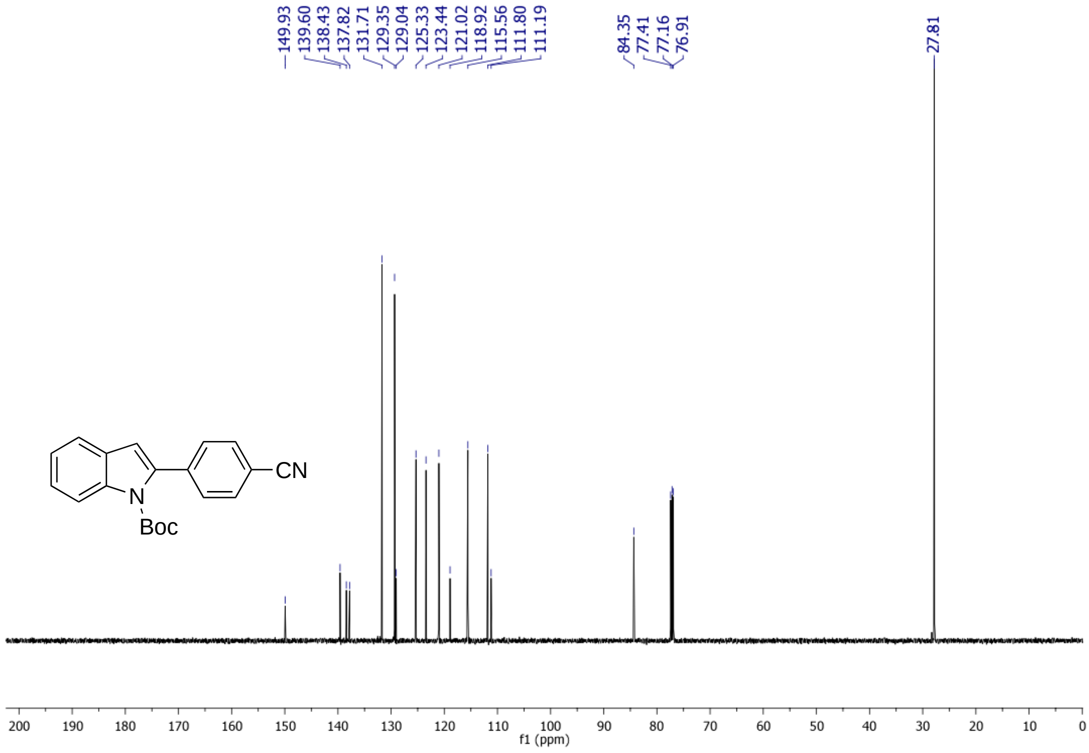

^{13}C NMR (126 MHz, CDCl$_3$) of compound **5**.

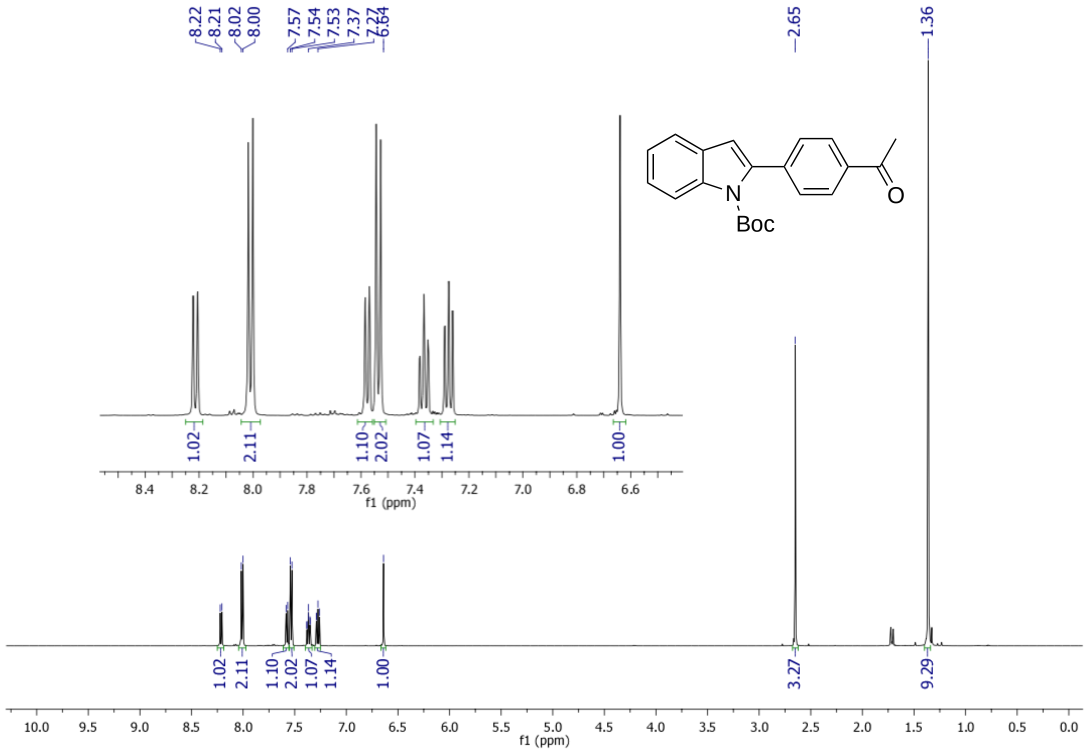

^{1}H NMR (500 MHz, CDCl$_3$) of compound **6**.

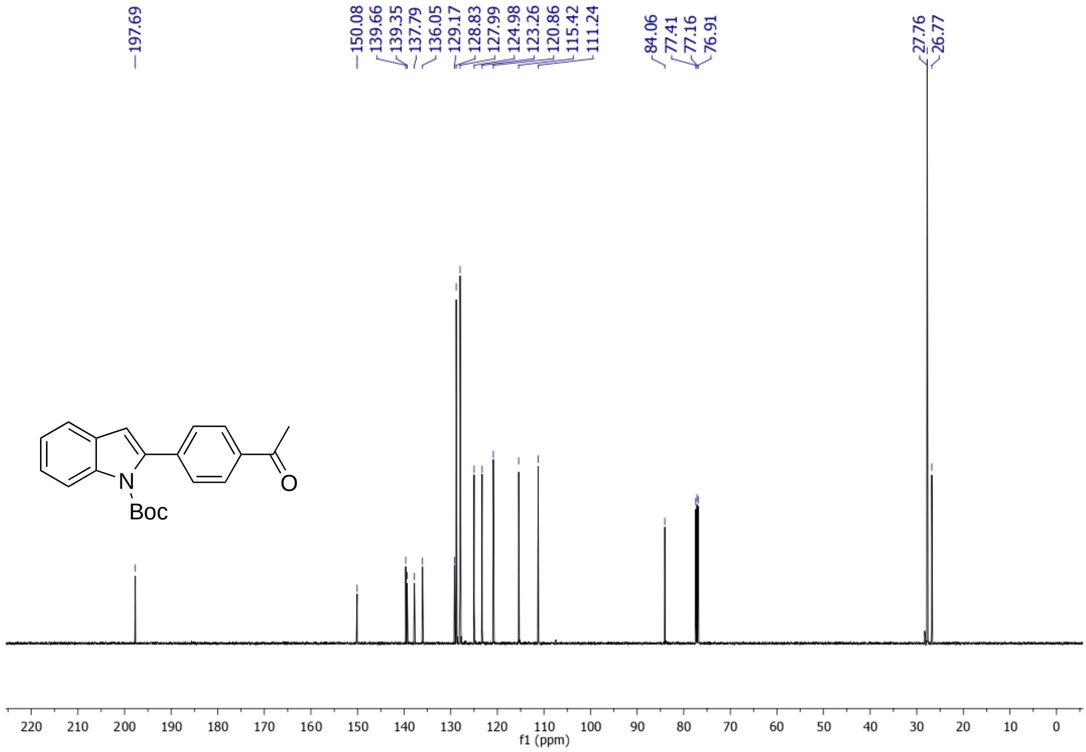

^{13}C **NMR (126 MHz, $CDCl_3$)** of compound **6**.

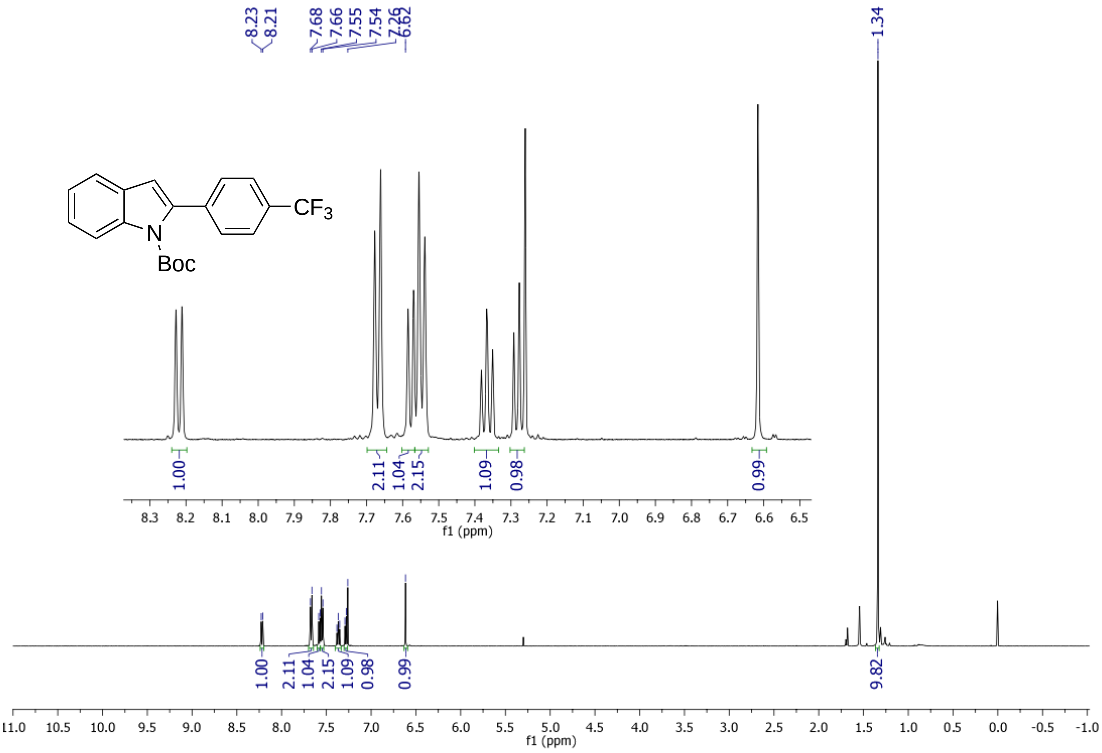

^{1}H **NMR (500 MHz, $CDCl_3$)** of compound **7**.

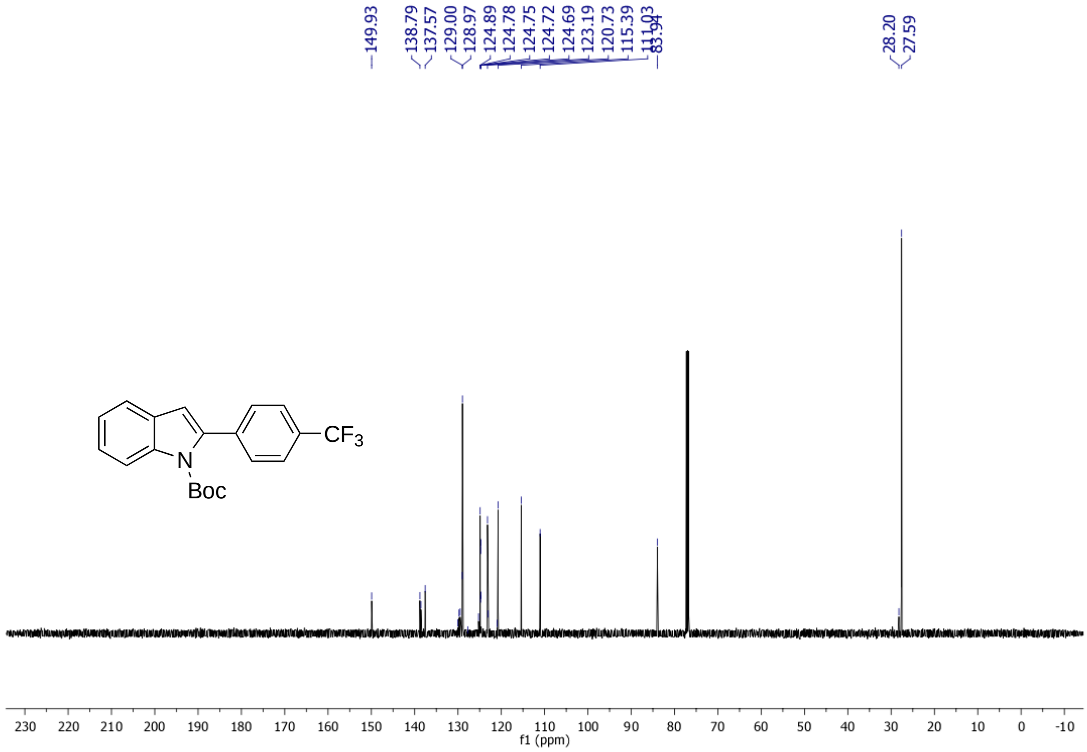

^{13}C NMR (126 MHz, CDCl$_3$) of compound **7**.

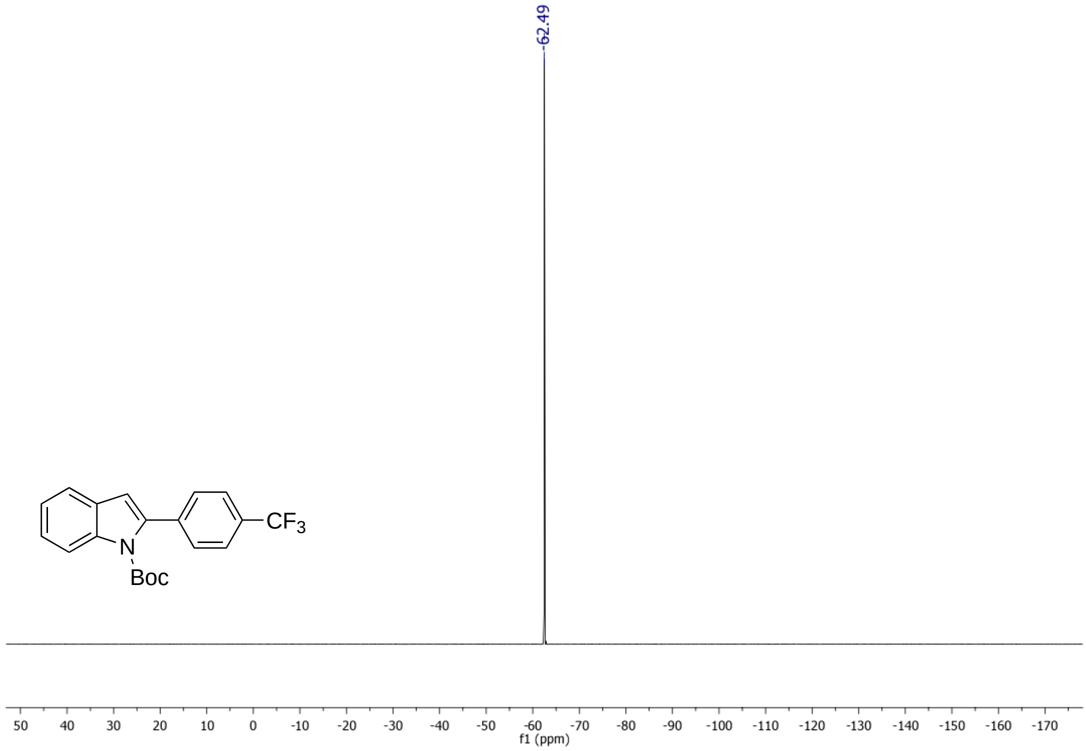

^{19}F NMR (470 MHz, CDCl$_3$) of Compound **7**.

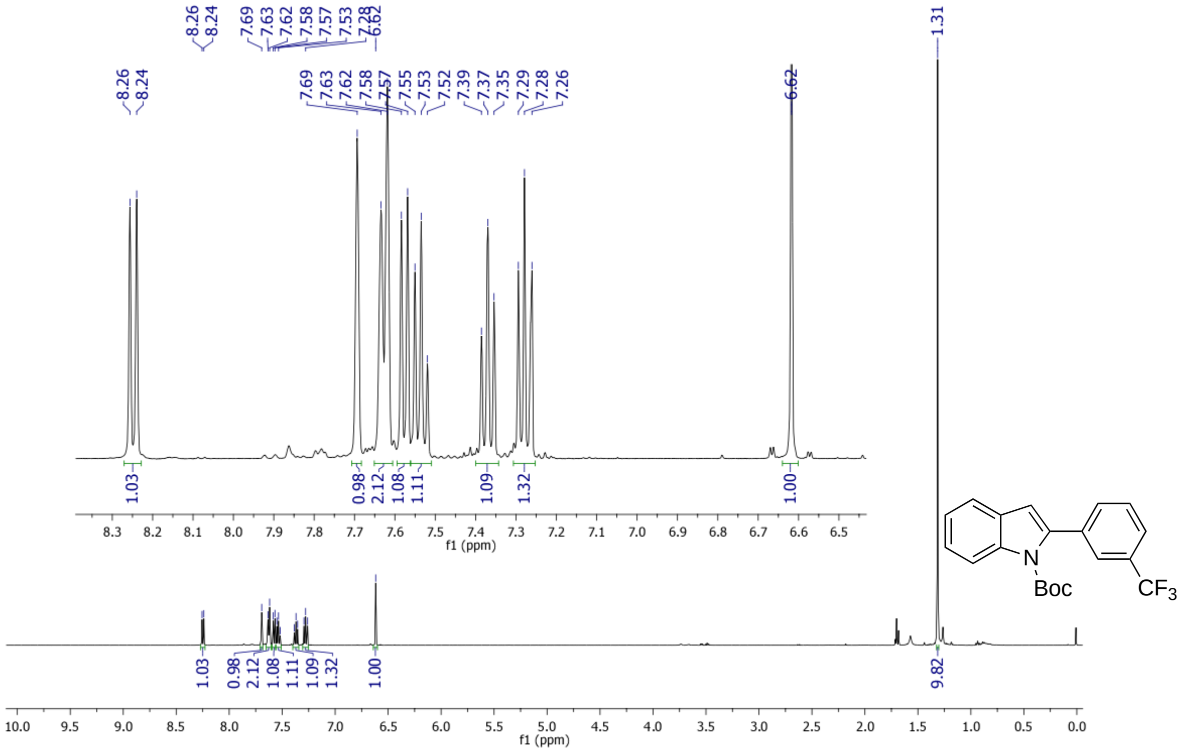

^{1}H NMR (500 MHz, CDCl3) of compound **8**.

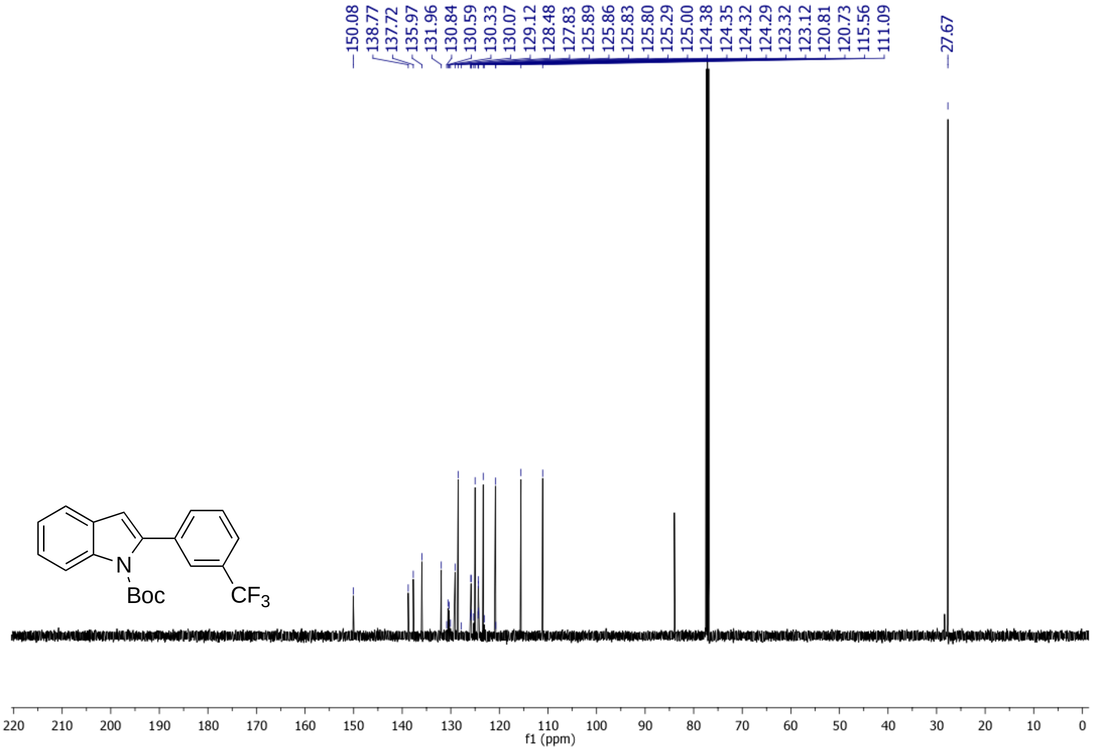

^{13}C NMR (126 MHz, CDCl3) of compound **8**.

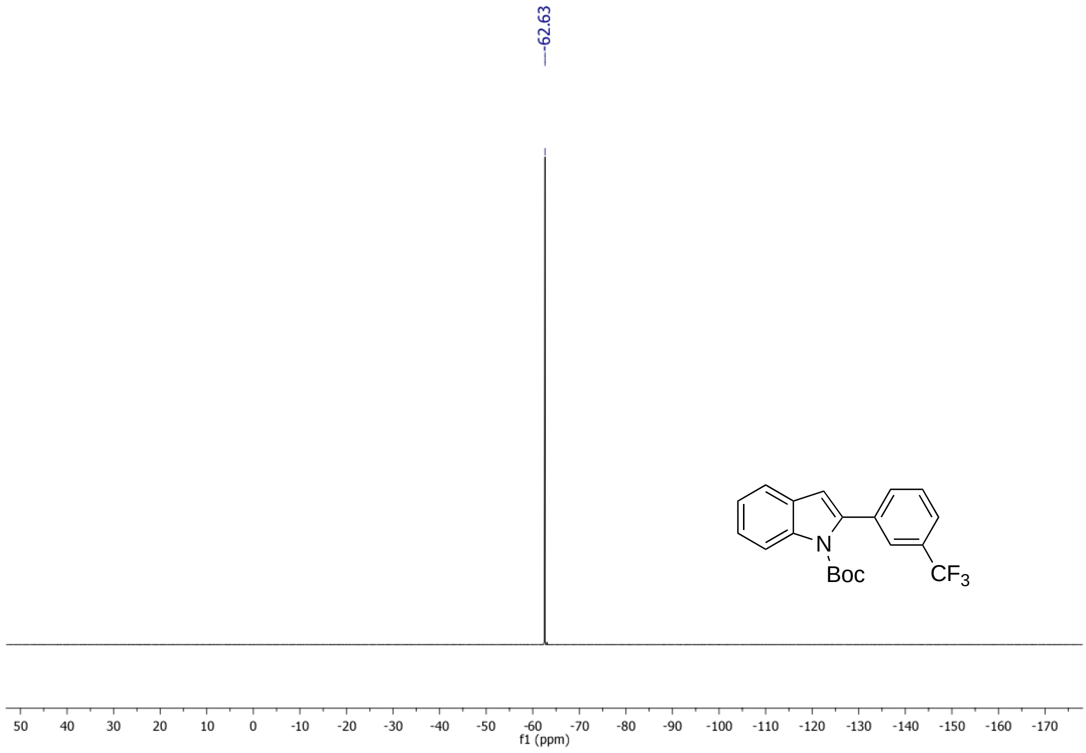

^{19}F NMR (470 MHz, $CDCl_3$) of Compound **8**.

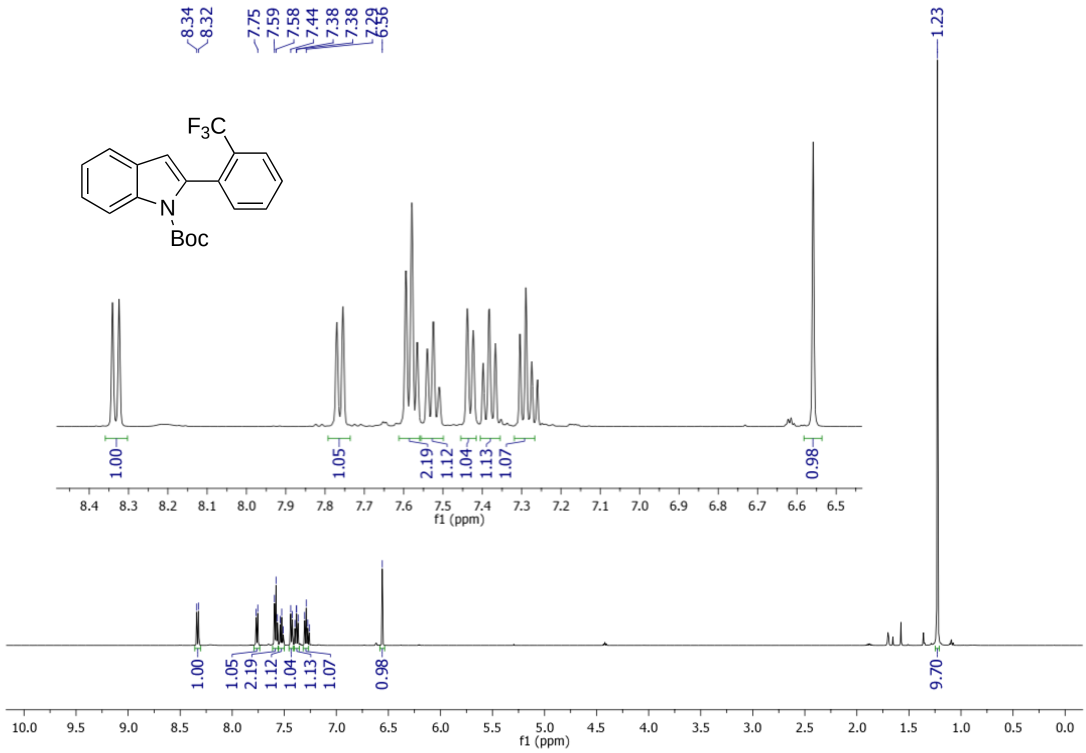

^{1}H NMR (500 MHz, $CDCl_3$) of compound **9**.

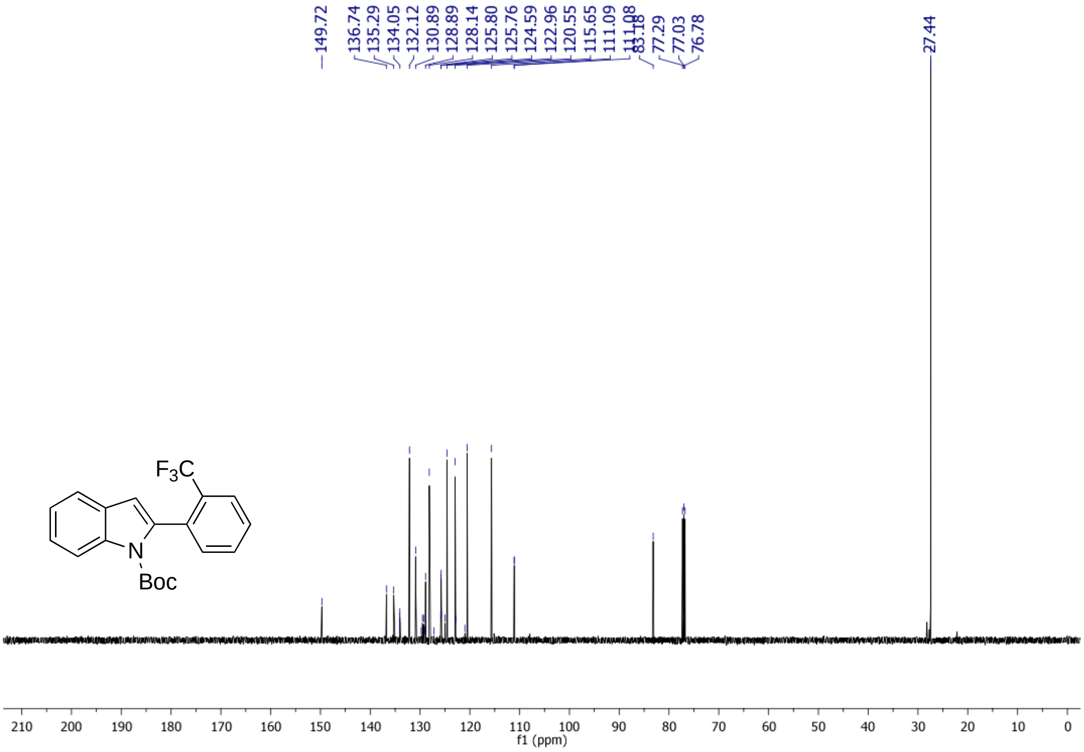

^{13}C NMR (126 MHz, $CDCl_3$) of compound **9**.

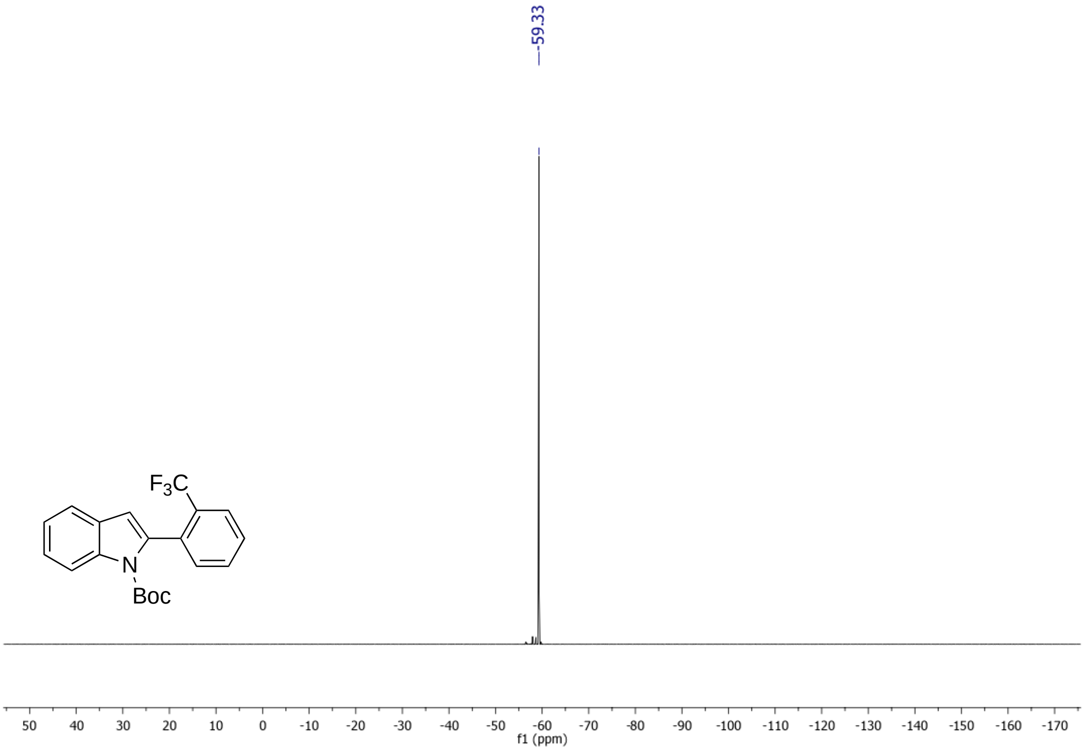

^{19}F NMR (470 MHz, $CDCl_3$) of Compound **9**.

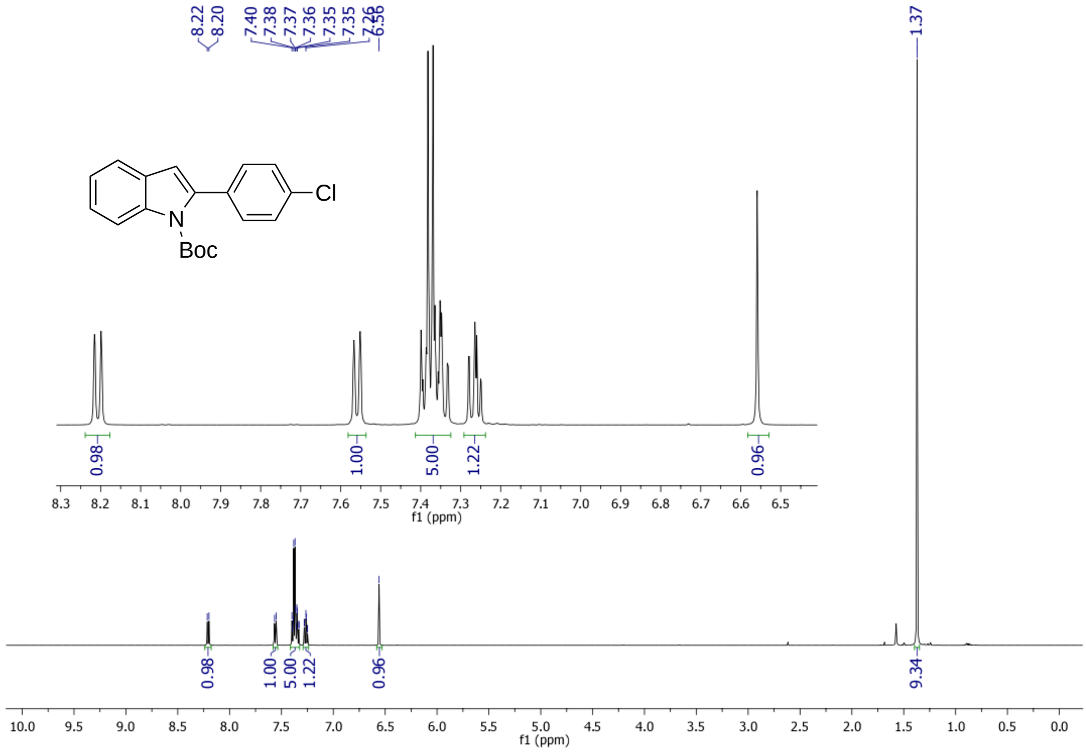

^{1}H NMR (500 MHz, CDCl3) of compound **10**.

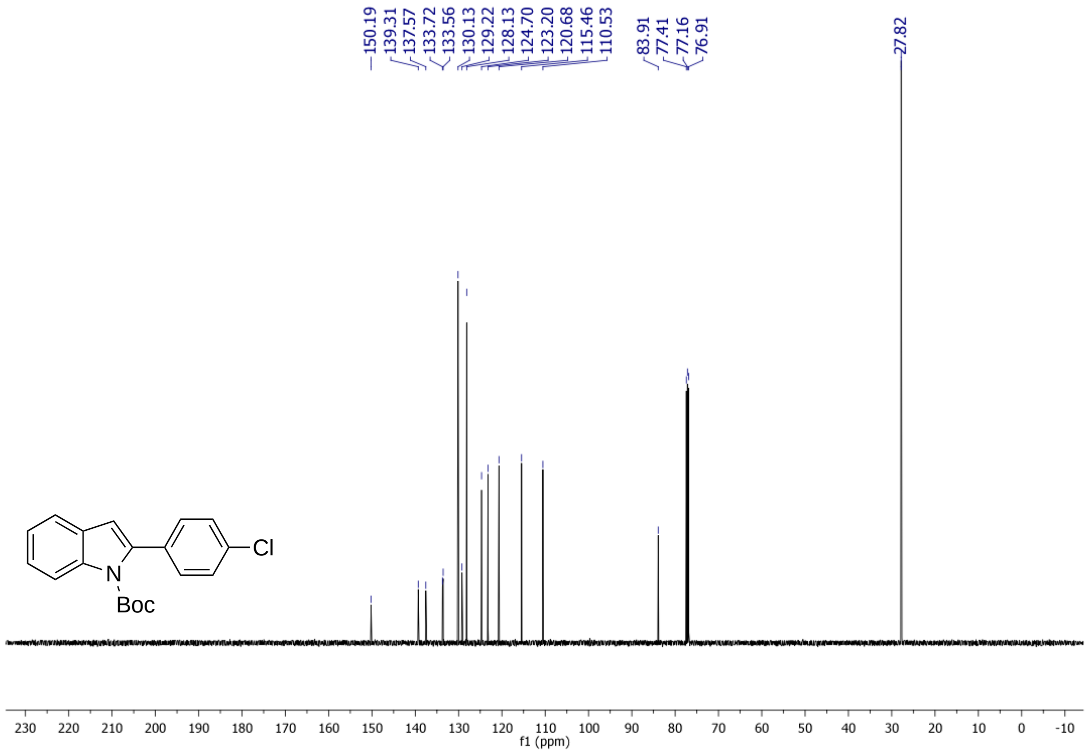

^{13}C NMR (126 MHz, CDCl3) of compound **10**.

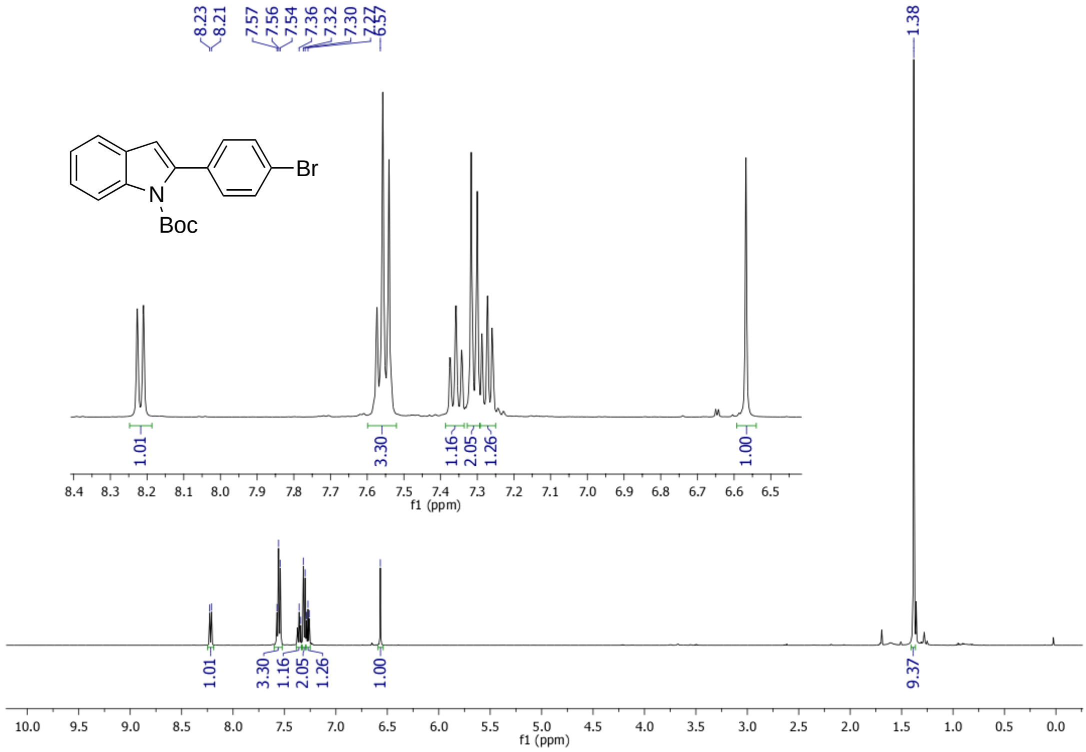

^{1}**H NMR (500 MHz, CDCl$_3$)** of compound **11**.

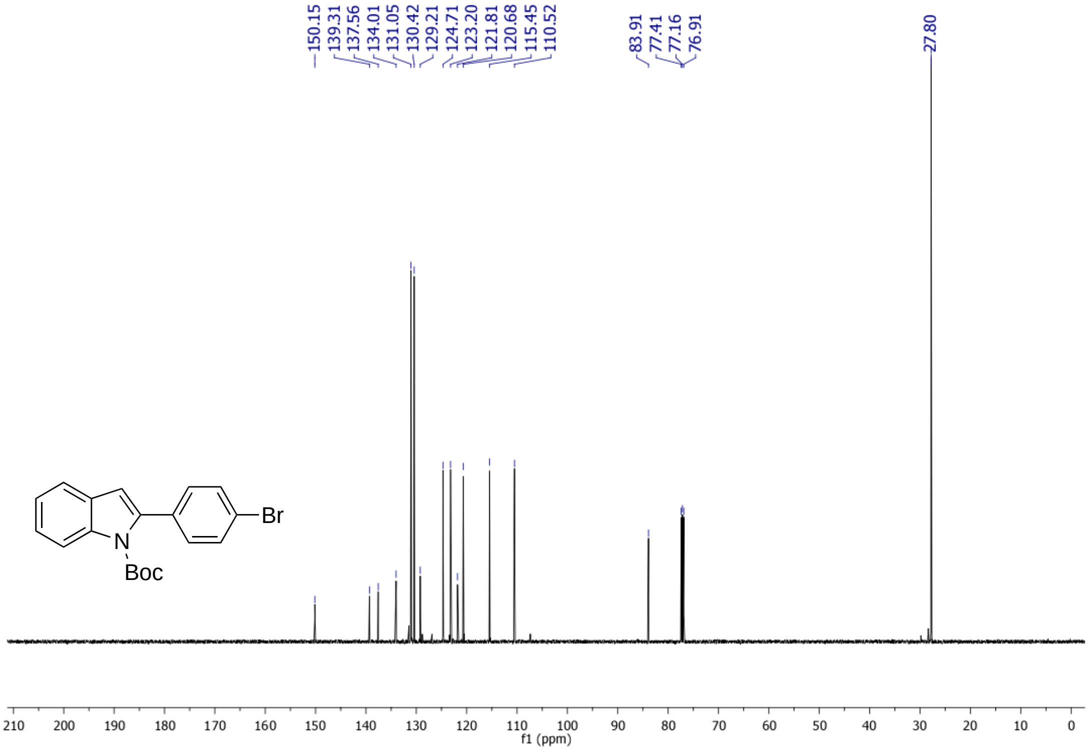

^{13}**C NMR (126 MHz, CDCl$_3$)** of compound **11**.

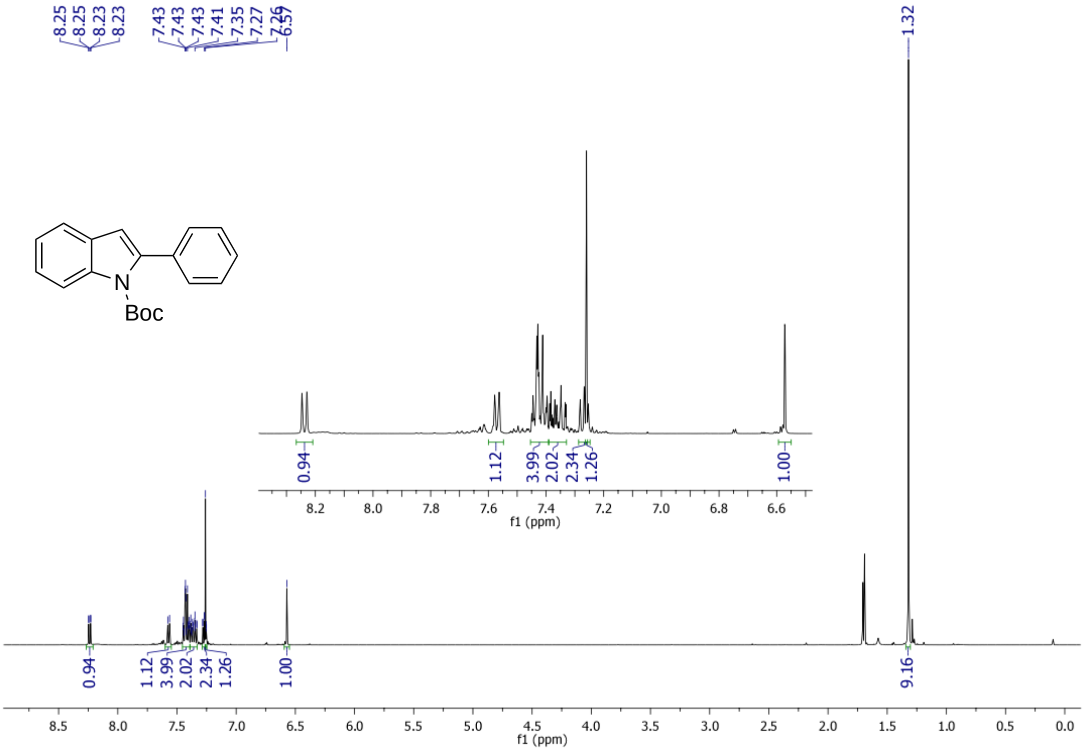

^{1}H NMR (500 MHz, $CDCl_3$) of compound **12**.

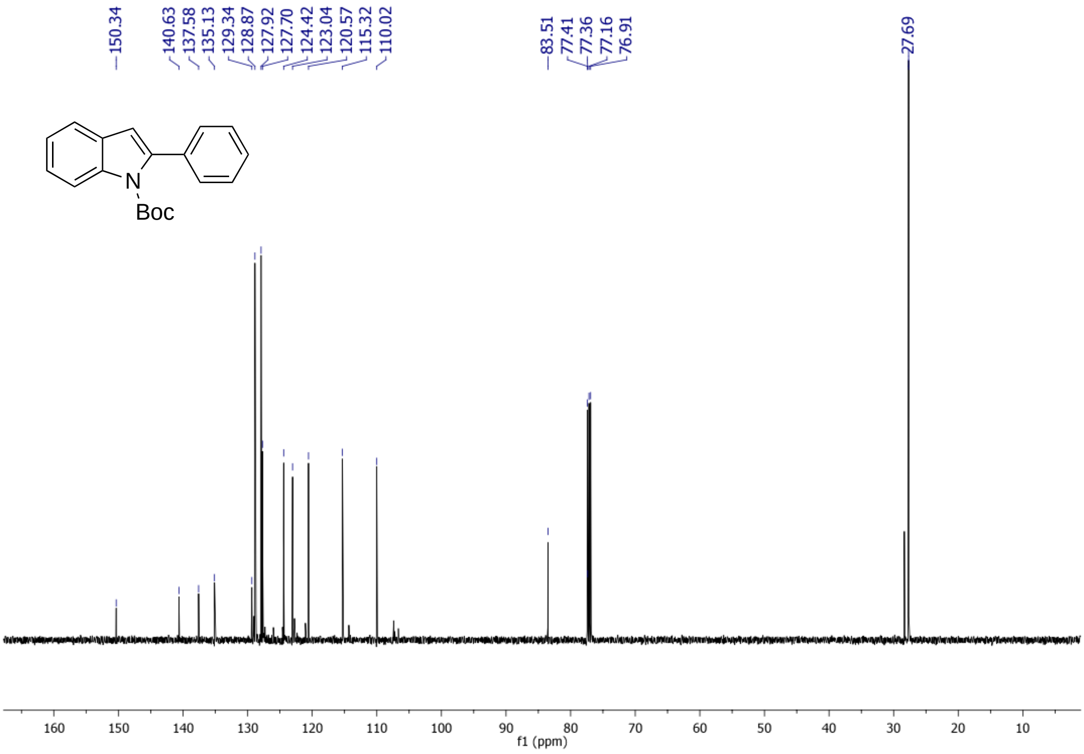

^{13}C NMR (126 MHz, $CDCl_3$) of compound **12**.

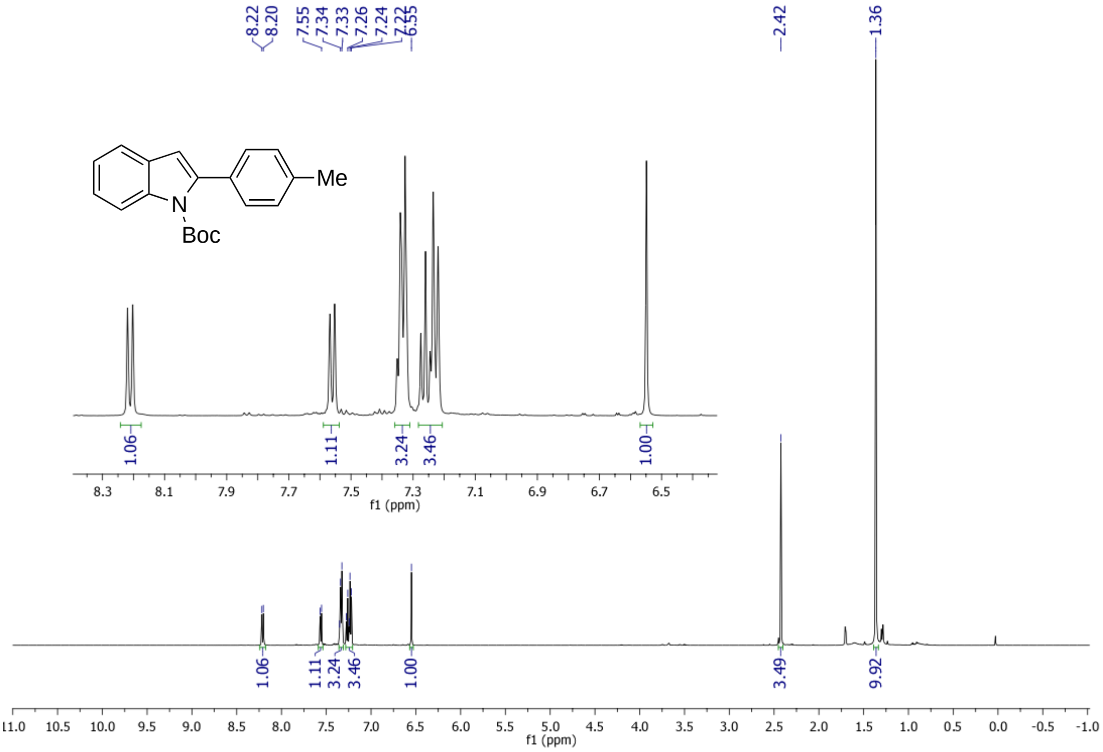

^{1}H NMR (500 MHz, $CDCl_3$) of compound **13**.

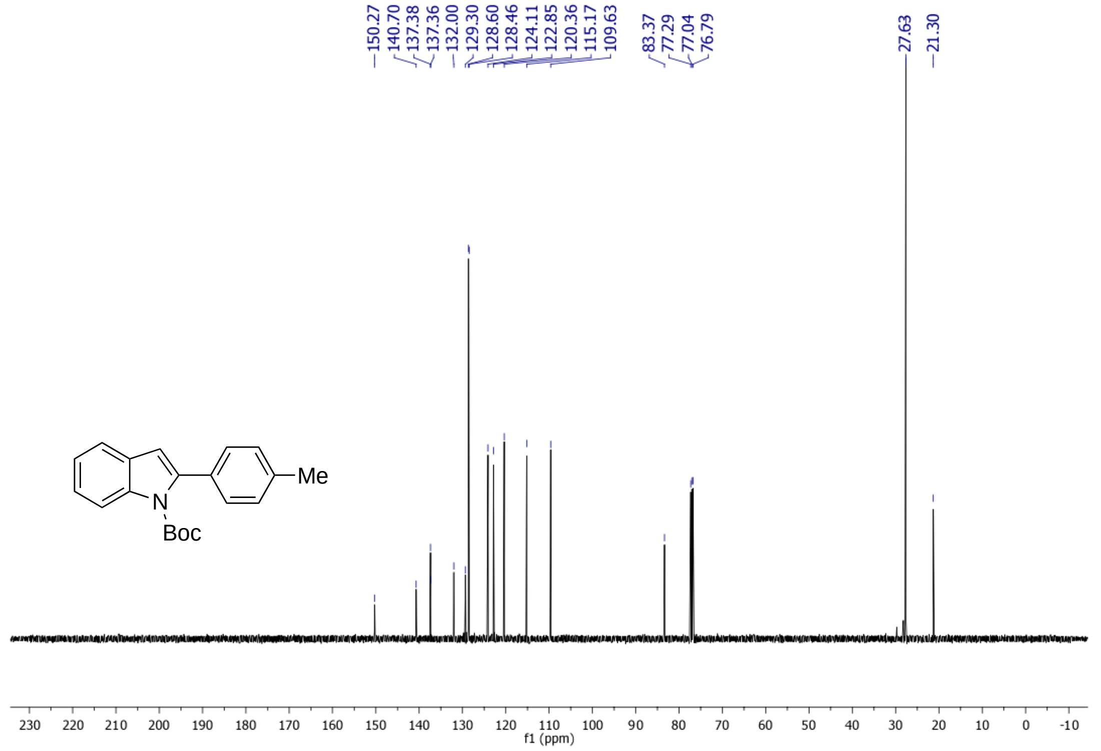

^{13}C NMR (126 MHz, $CDCl_3$) of compound **13**.

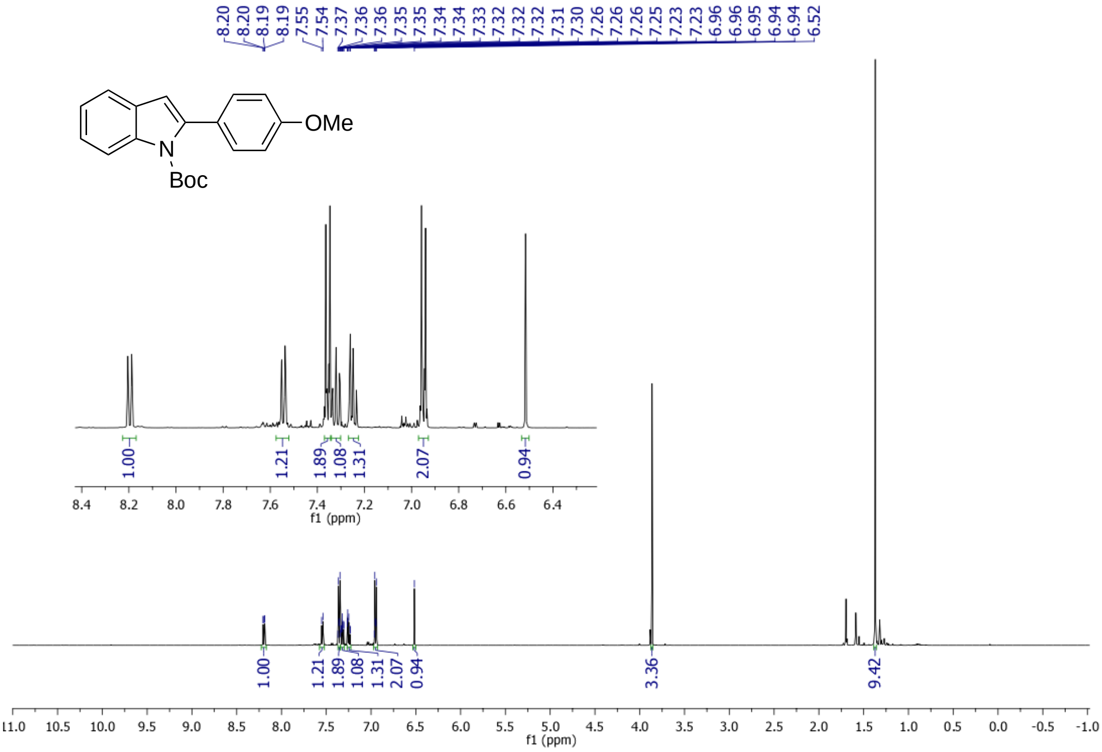

^{1}H NMR (500 MHz, $CDCl_3$) of compound **14**.

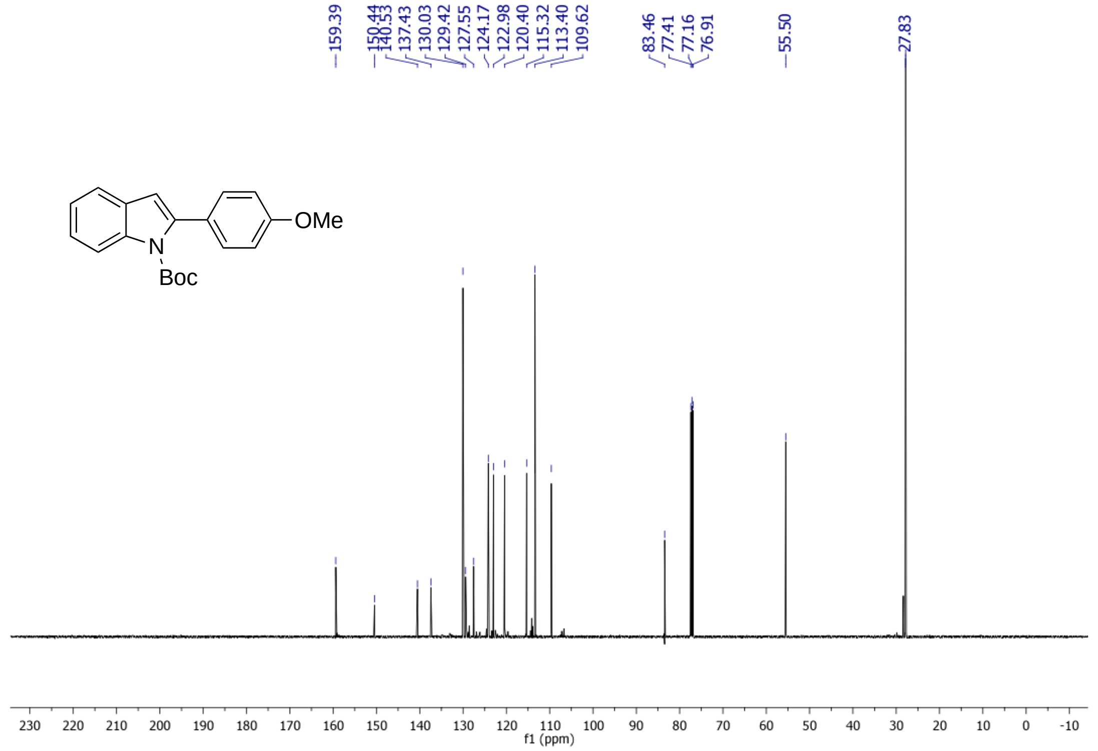

^{13}C NMR (126 MHz, $CDCl_3$) of compound **14**.

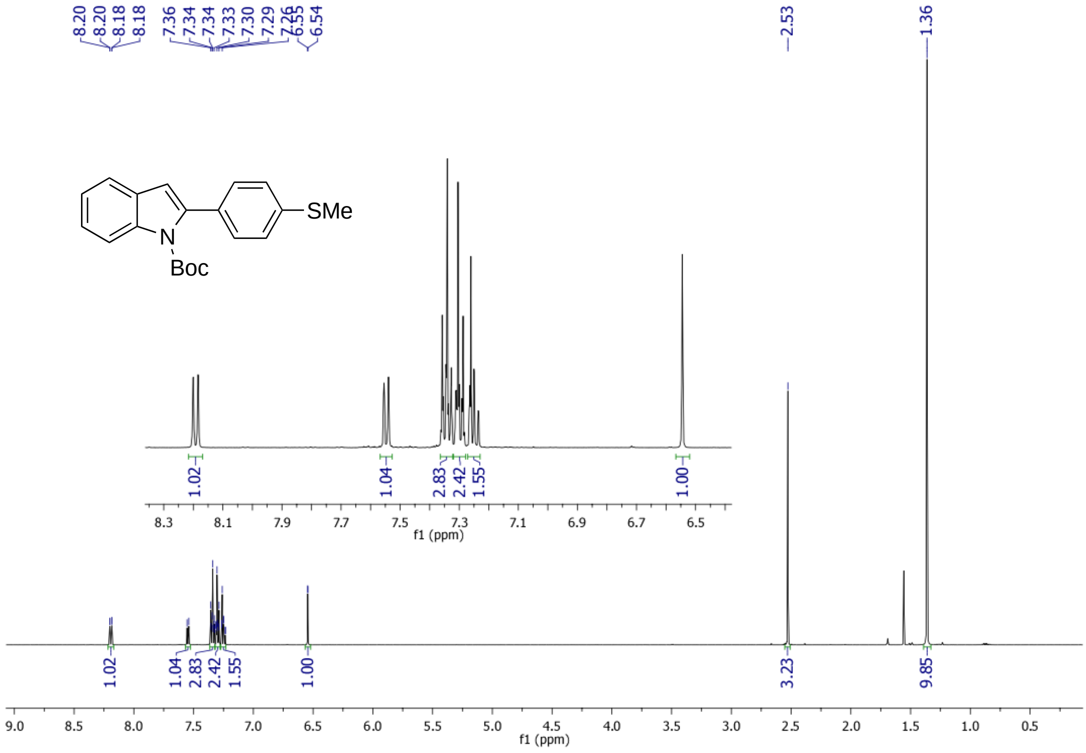

^{1}H NMR (500 MHz, CDCl$_3$) of compound **15**.

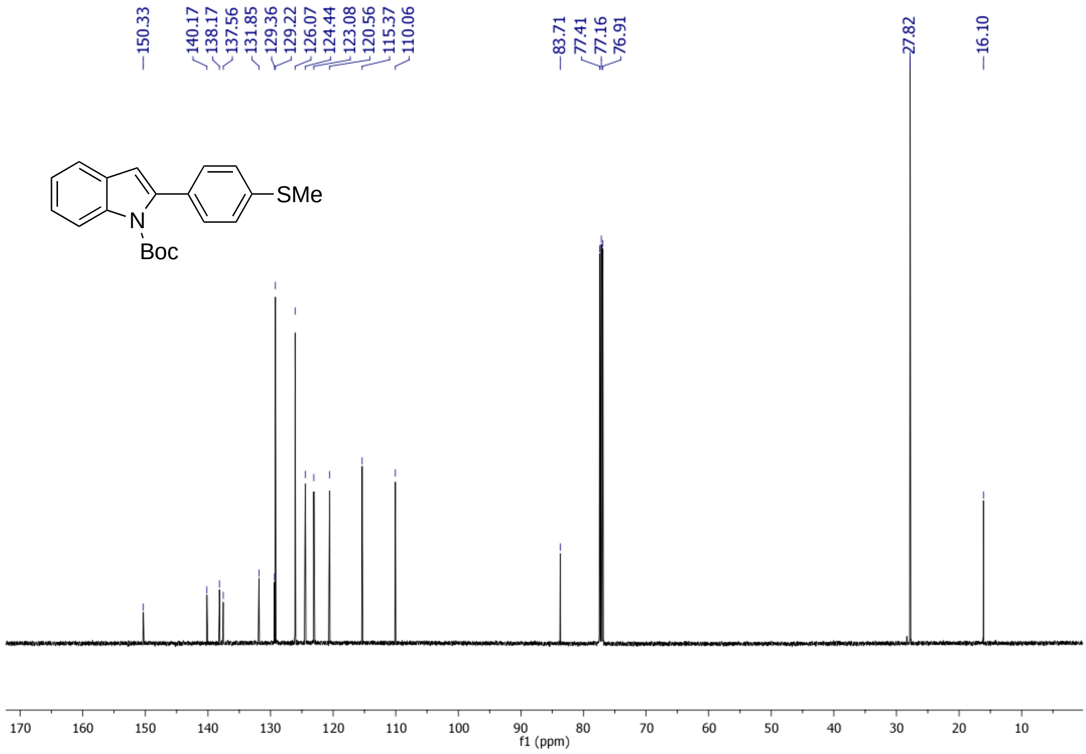

^{13}C NMR (126 MHz, CDCl$_3$) of compound **15**.

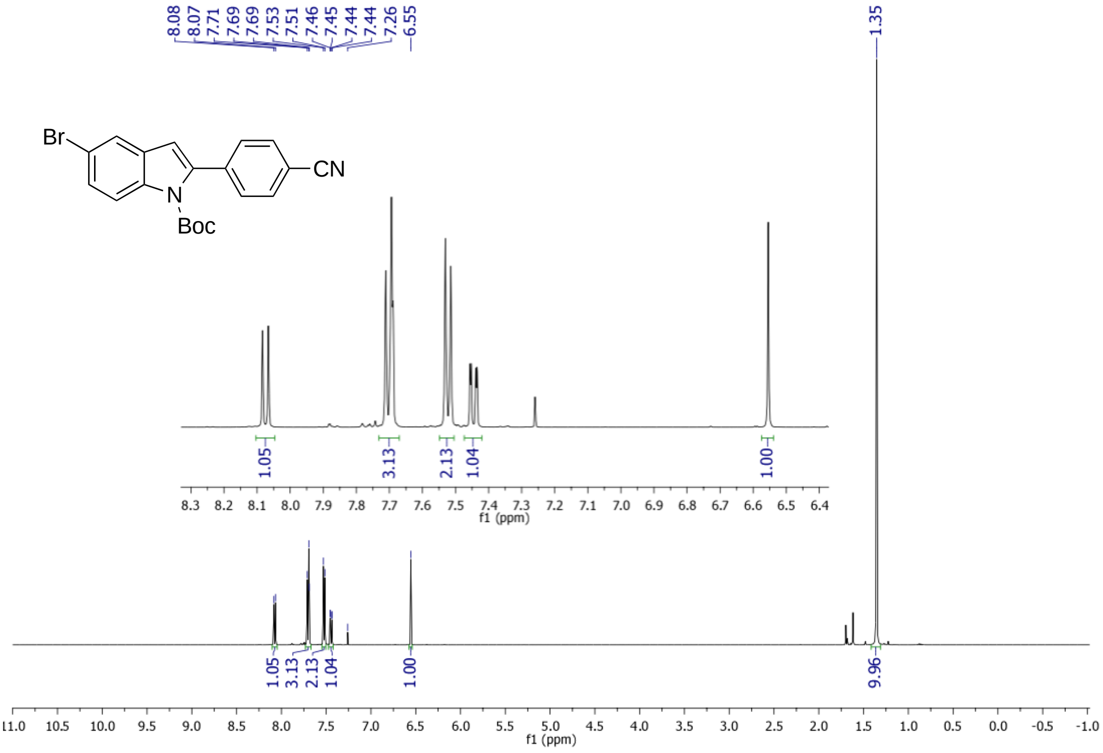

^{1}H NMR (500 MHz, $CDCl_3$) of compound **16**.

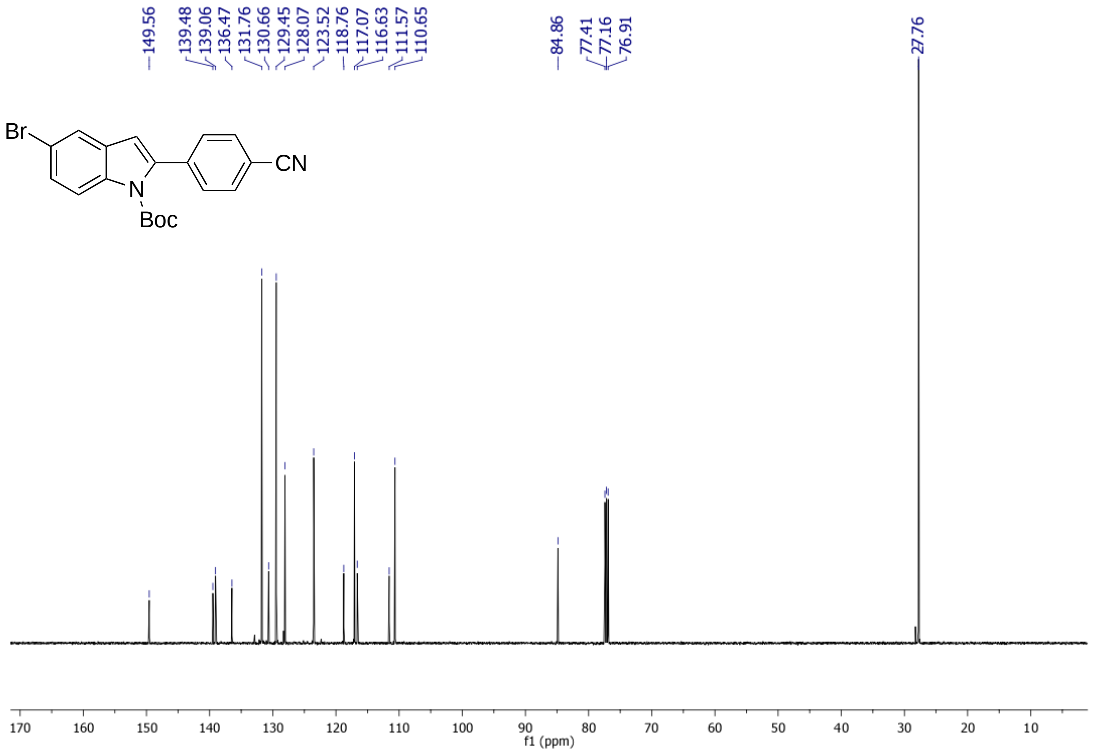

^{13}C NMR (126 MHz, $CDCl_3$) of compound **16**.

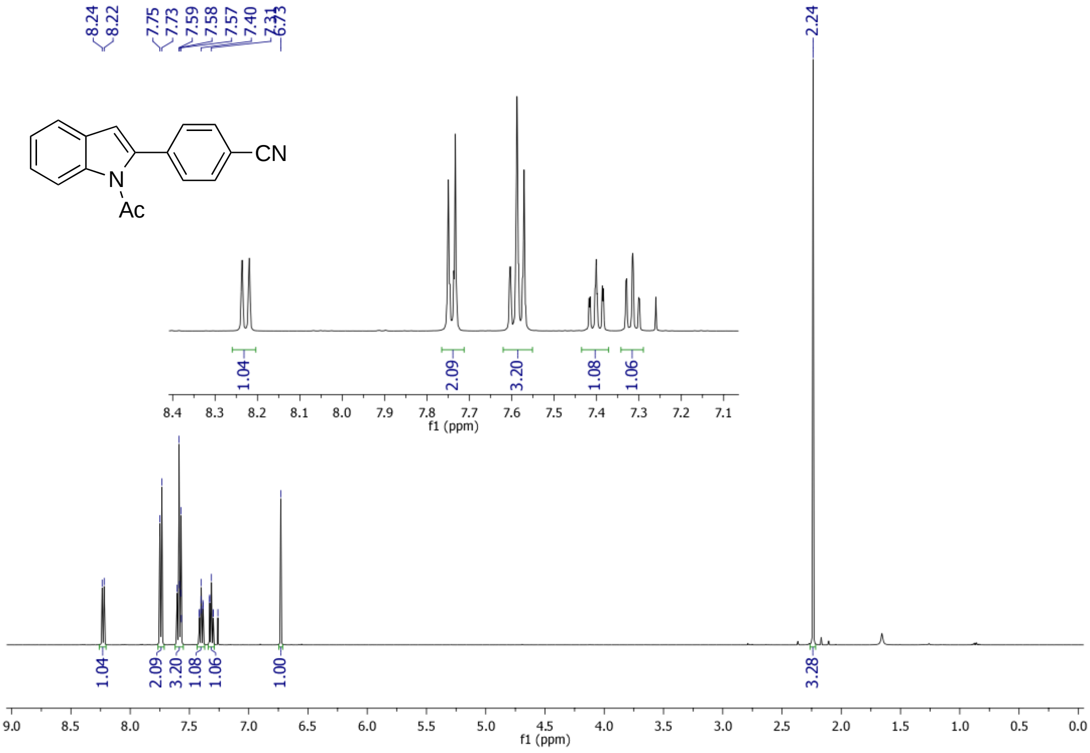

^{1}**H NMR (500 MHz, CDCl$_3$)** of compound **17**.

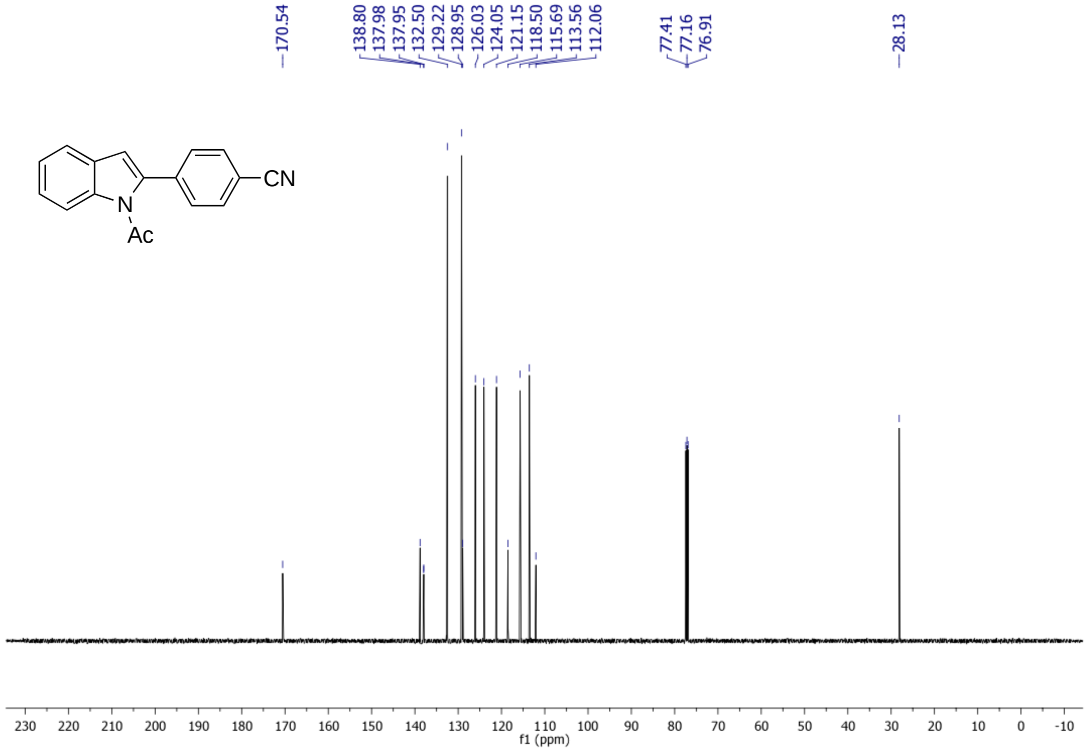

^{13}**C NMR (126 MHz, CDCl$_3$)** of compound **17**.

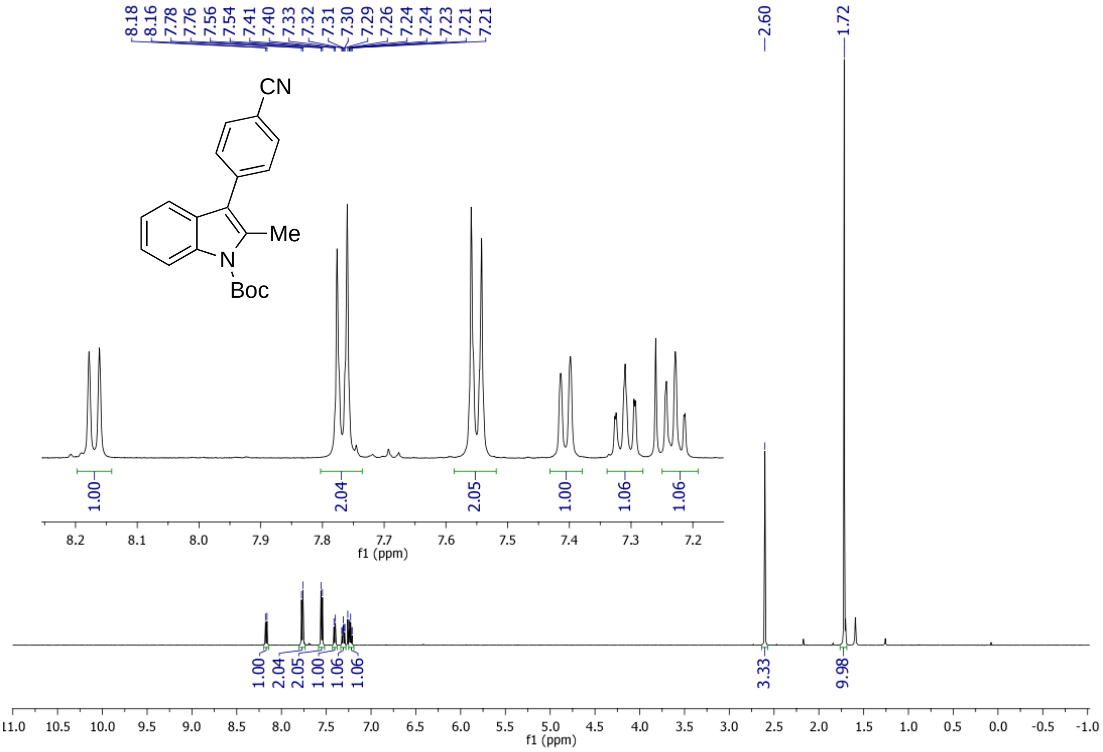

^{1}**H NMR (500 MHz, CDCl$_3$)** of compound **18**.

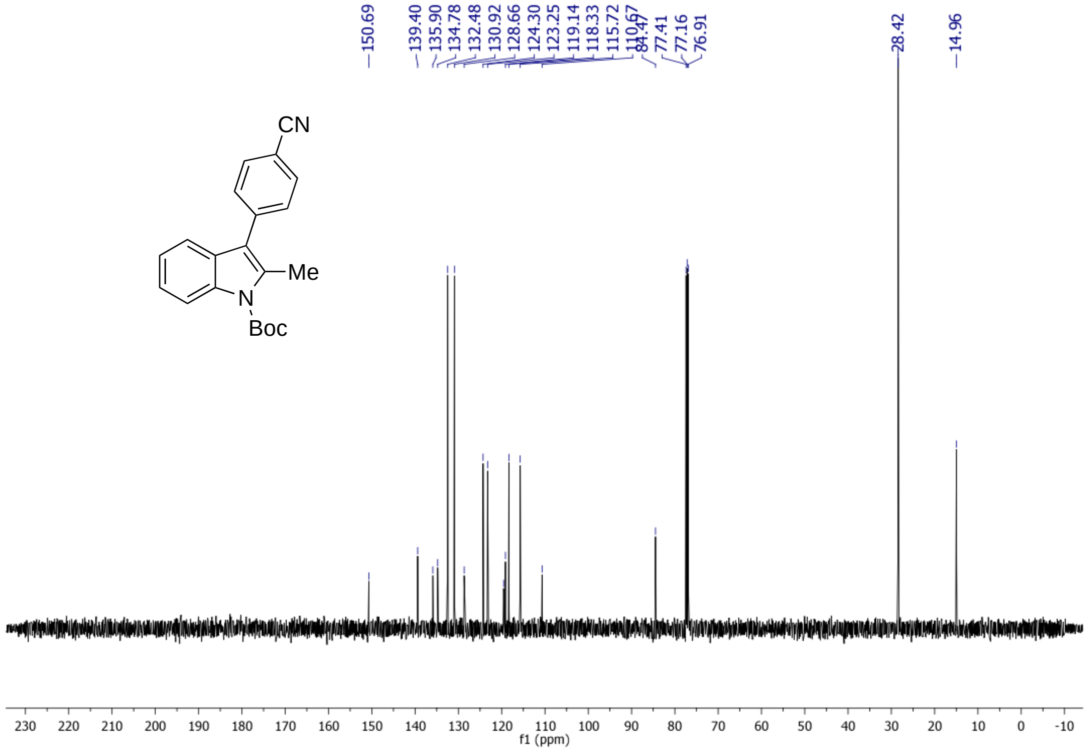

^{13}**C NMR (126 MHz, CDCl$_3$)** of compound **18**.

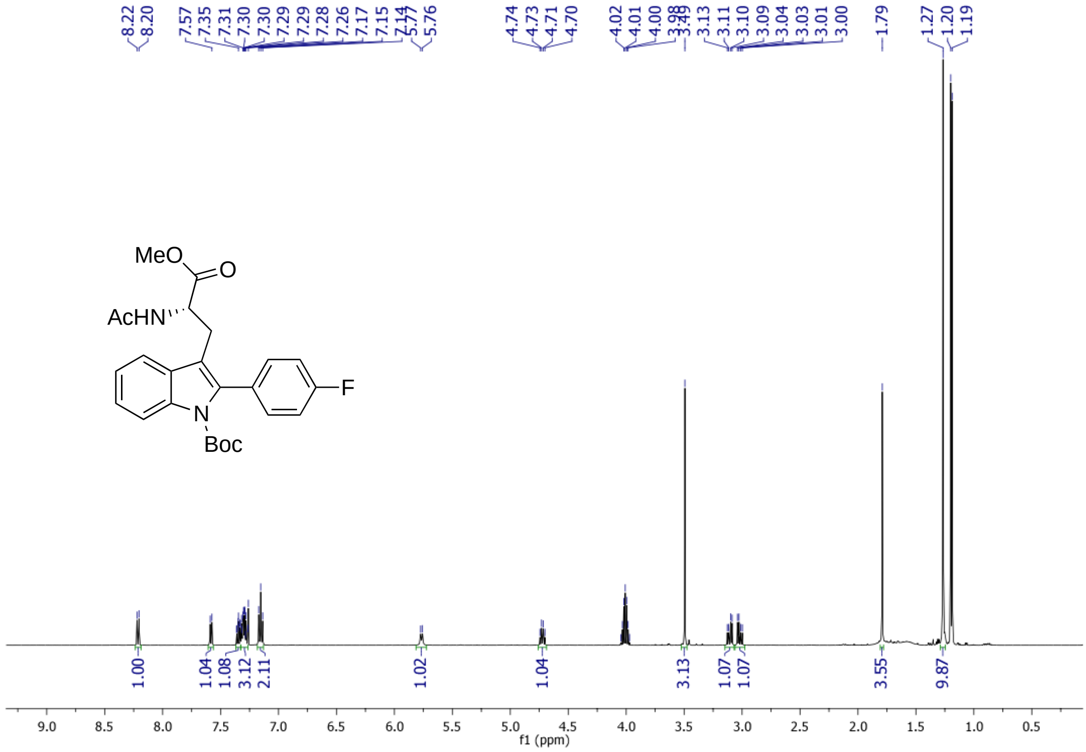

^{1}H NMR (500 MHz, $CDCl_3$) of compound **20**.

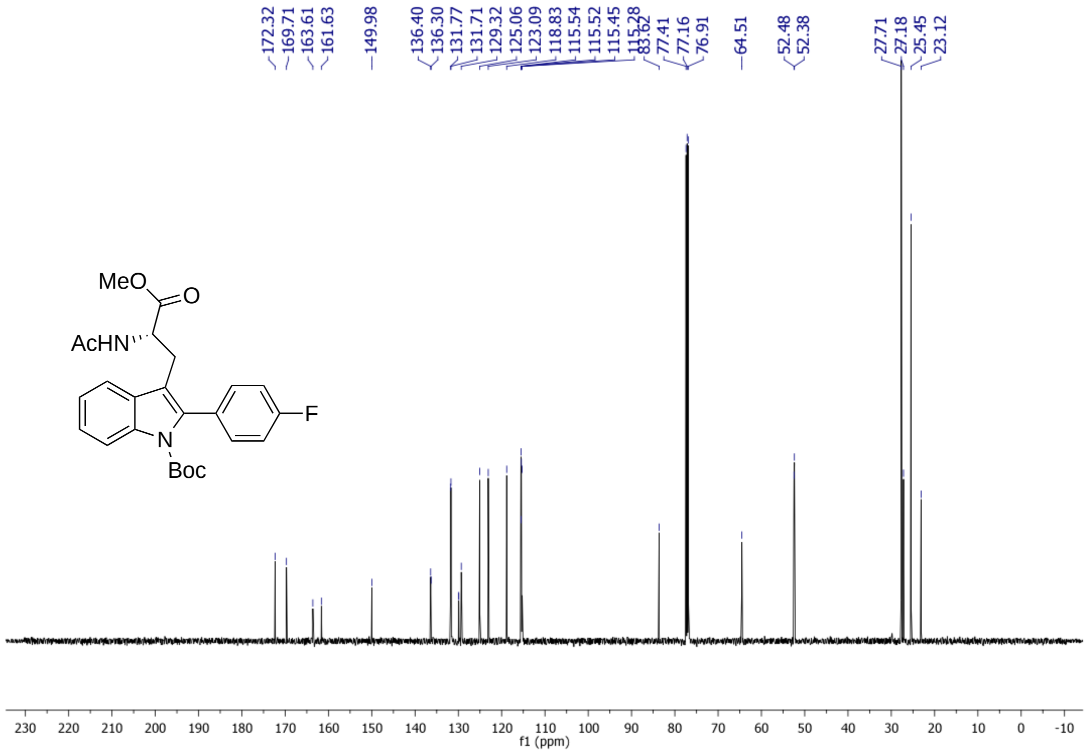

^{13}C NMR (126 MHz, $CDCl_3$) of compound **20**.

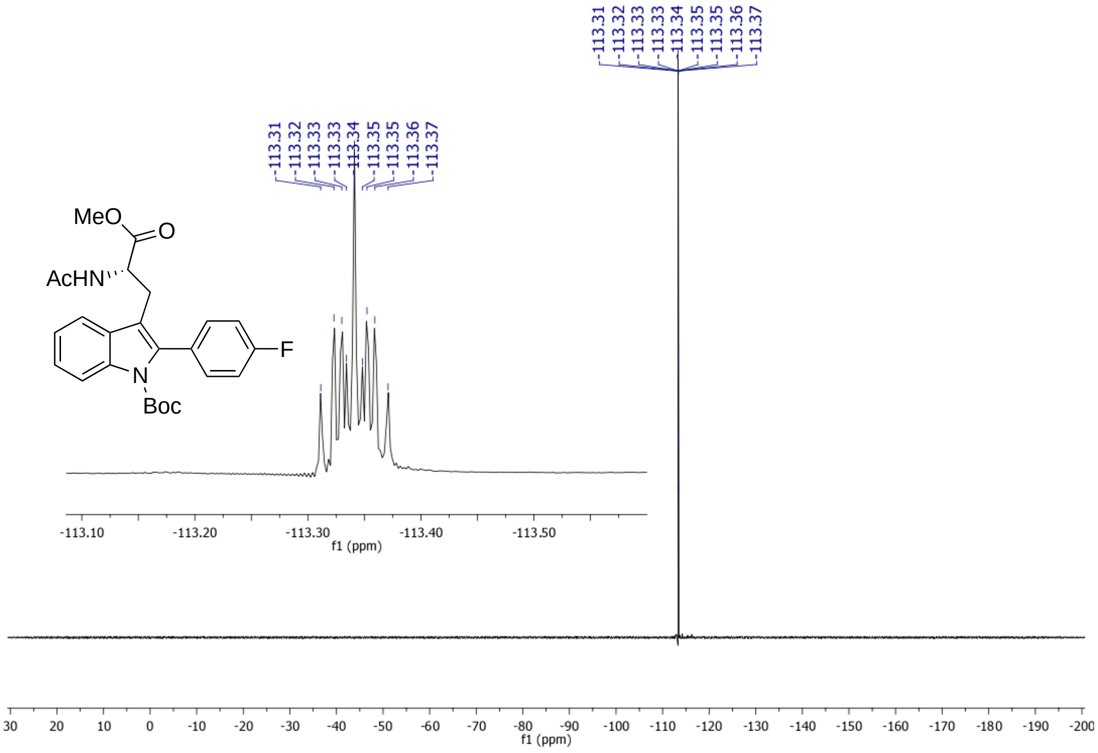

^{19}F NMR (470 MHz, CDCl$_3$) of Compound **20**.

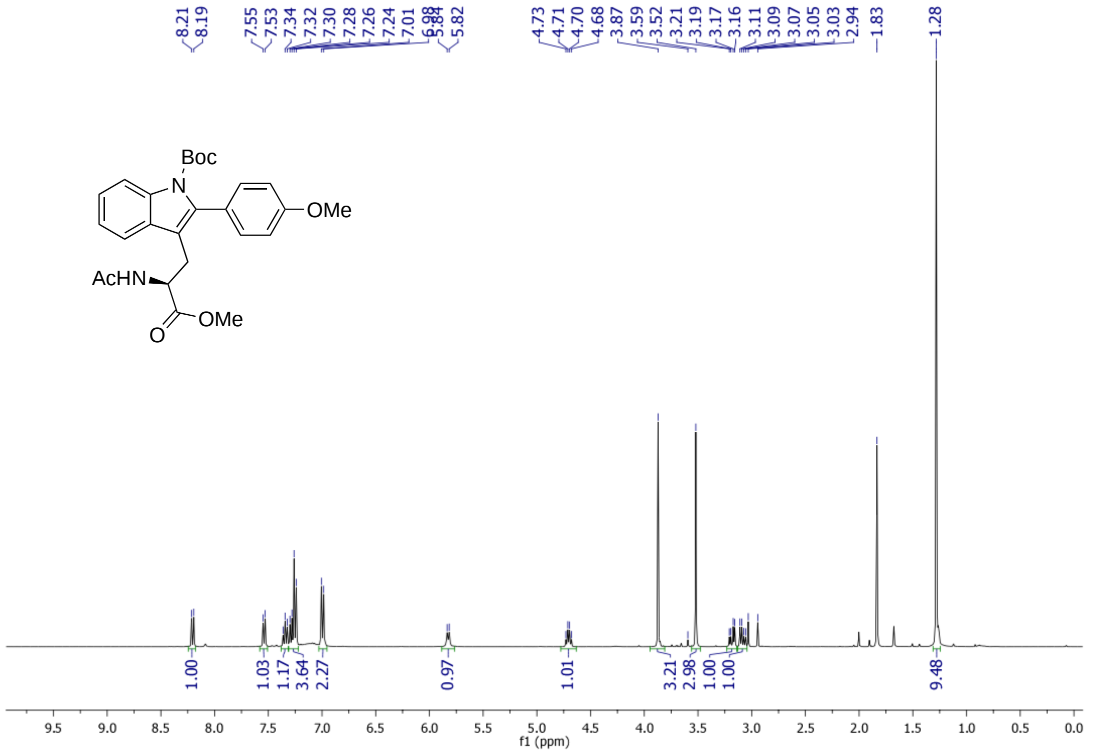

^{1}H NMR (400 MHz, CDCl$_3$) of compound **21**.

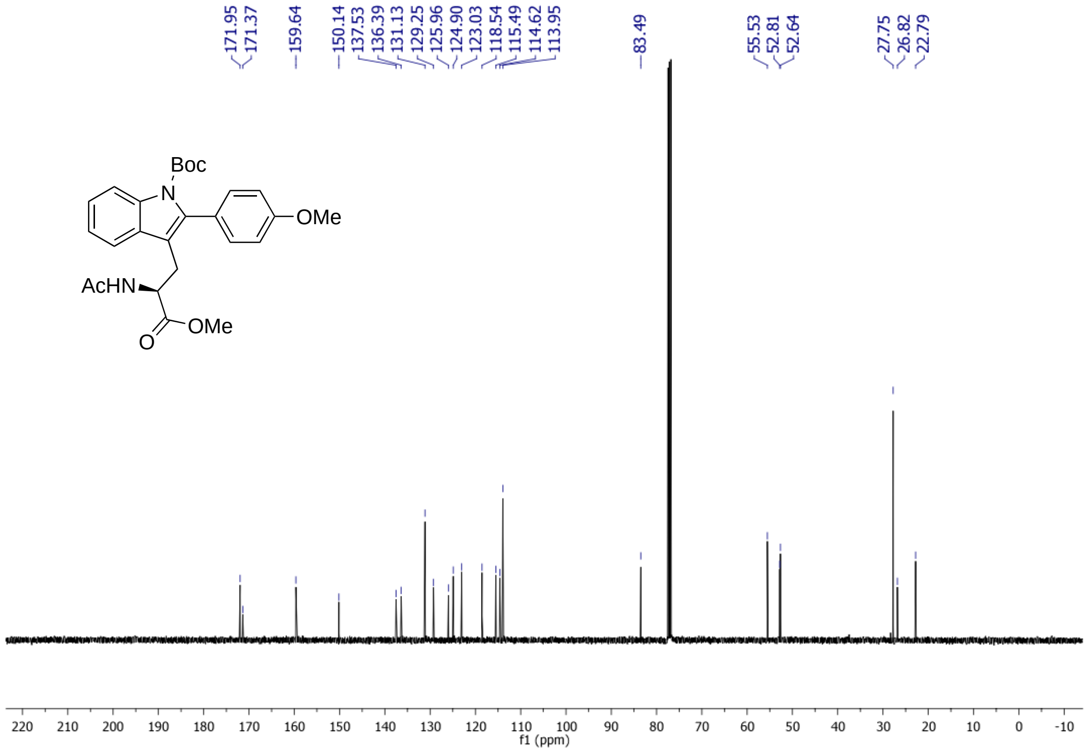

^{13}C NMR (101 MHz, CDCl$_3$) of compound **21**.

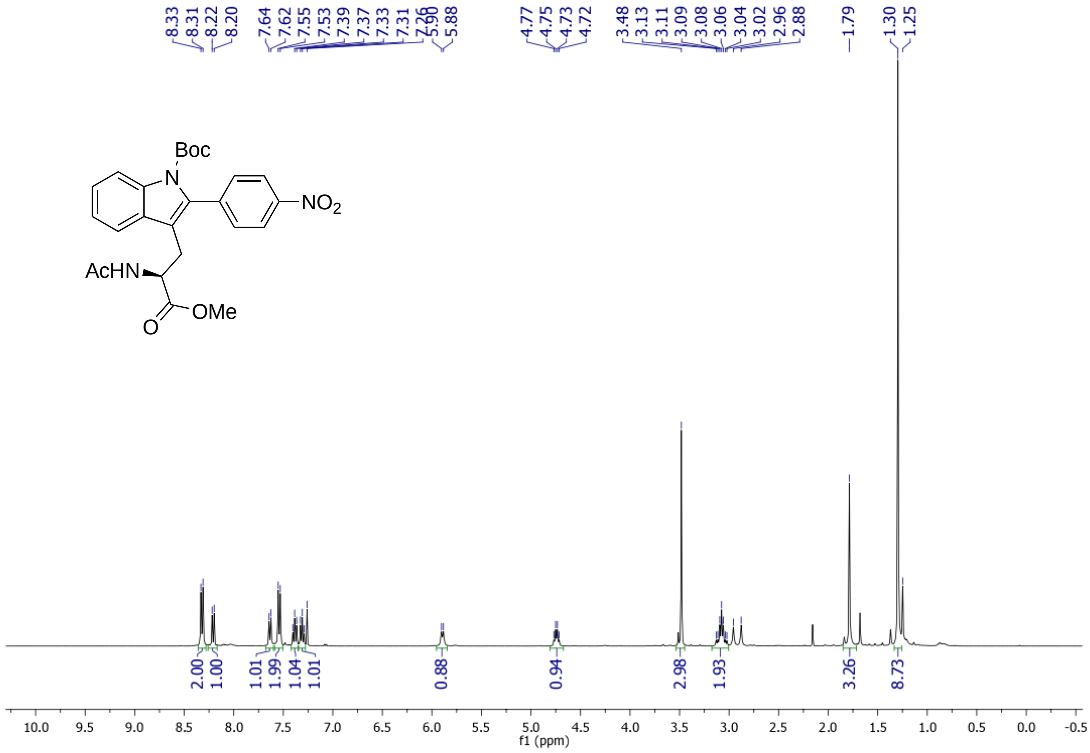

^{1}H NMR (400 MHz, CDCl$_3$) of compound **22**.

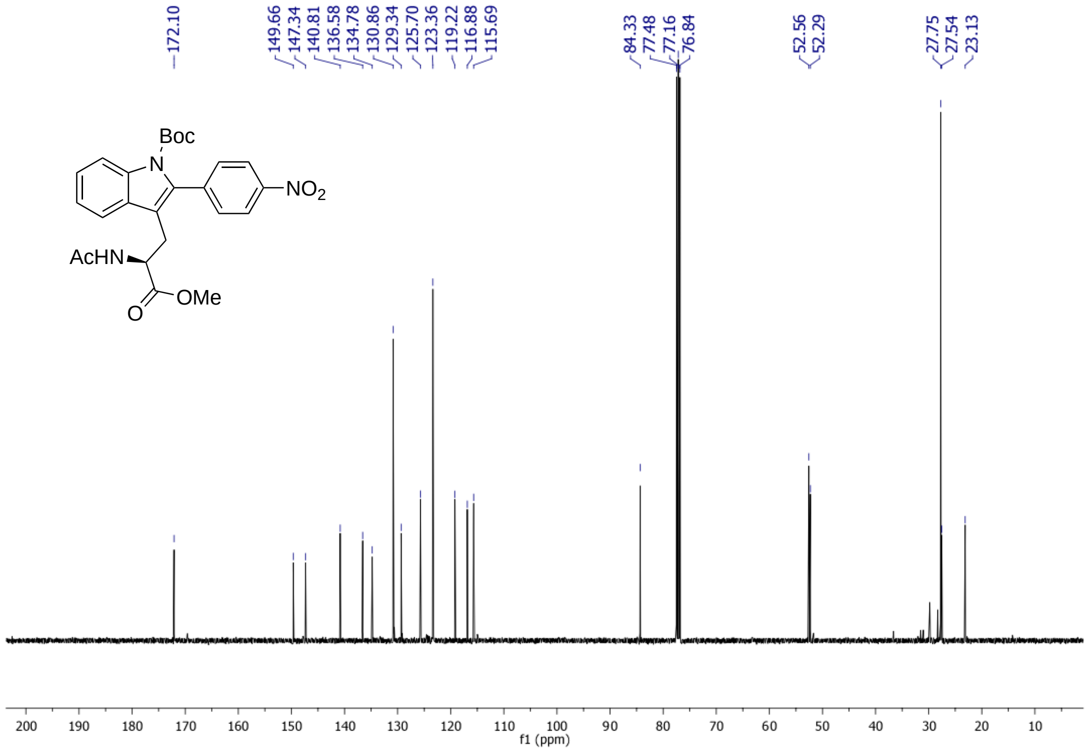

^{13}C NMR (101 MHz, $CDCl_3$) of compound **22**.

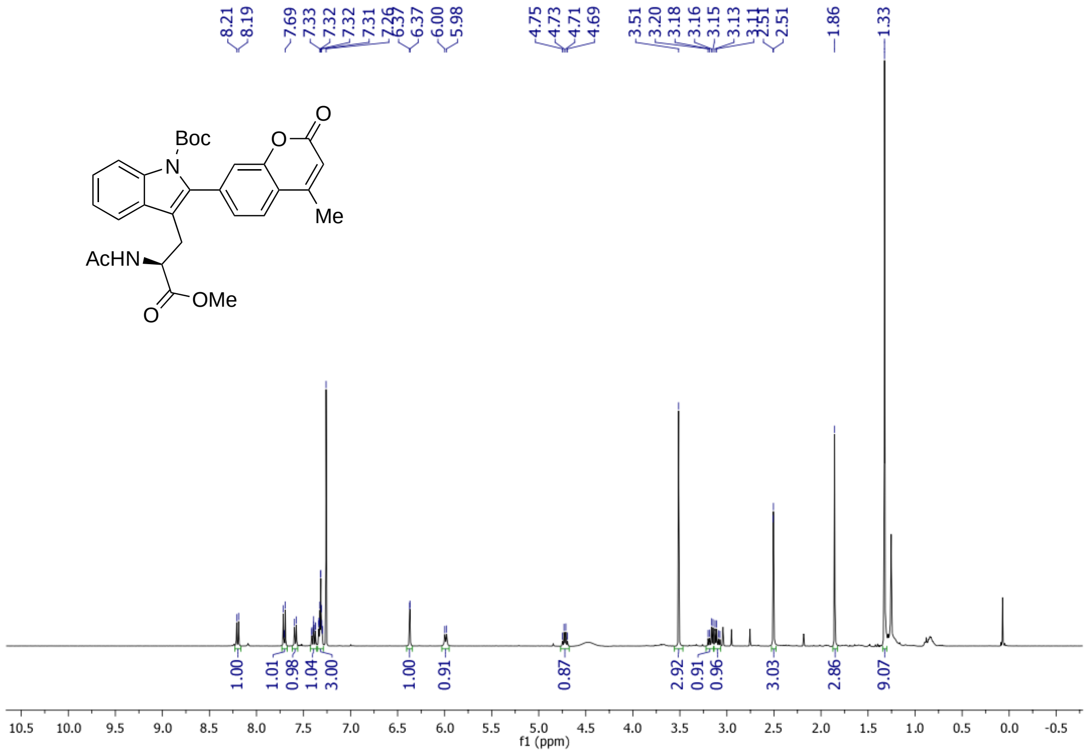

^{1}H NMR (400 MHz, $CDCl_3$) of compound **23**.

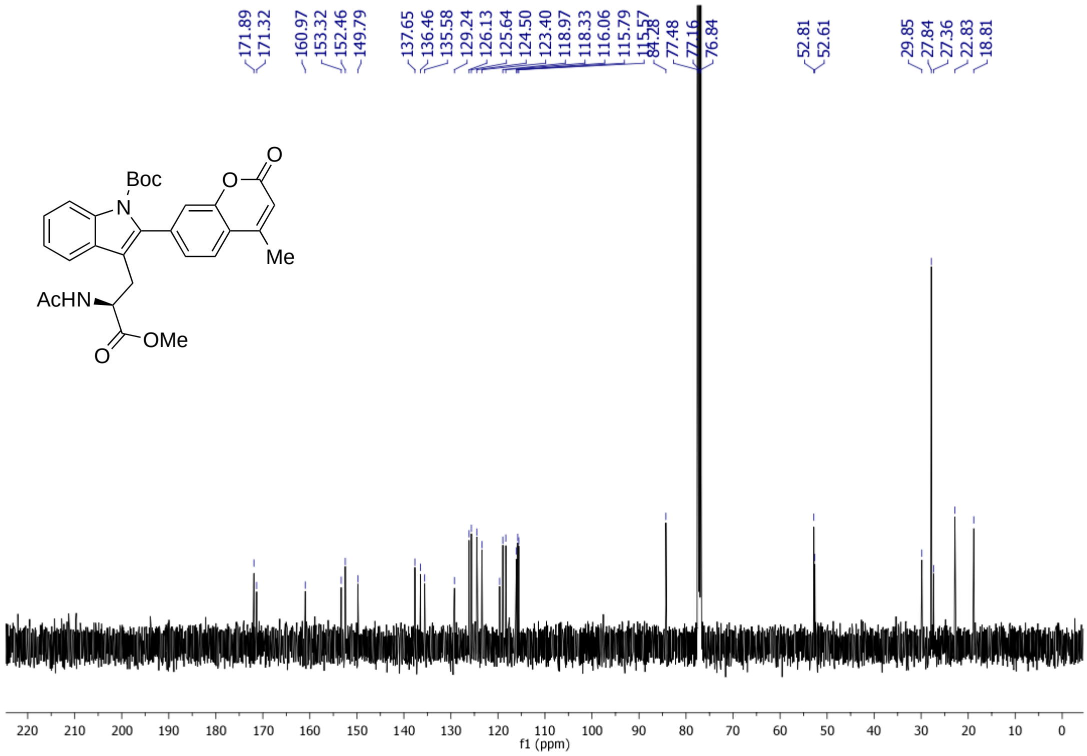

^{13}C NMR (101 MHz, CDCl$_3$) of compound **23**.

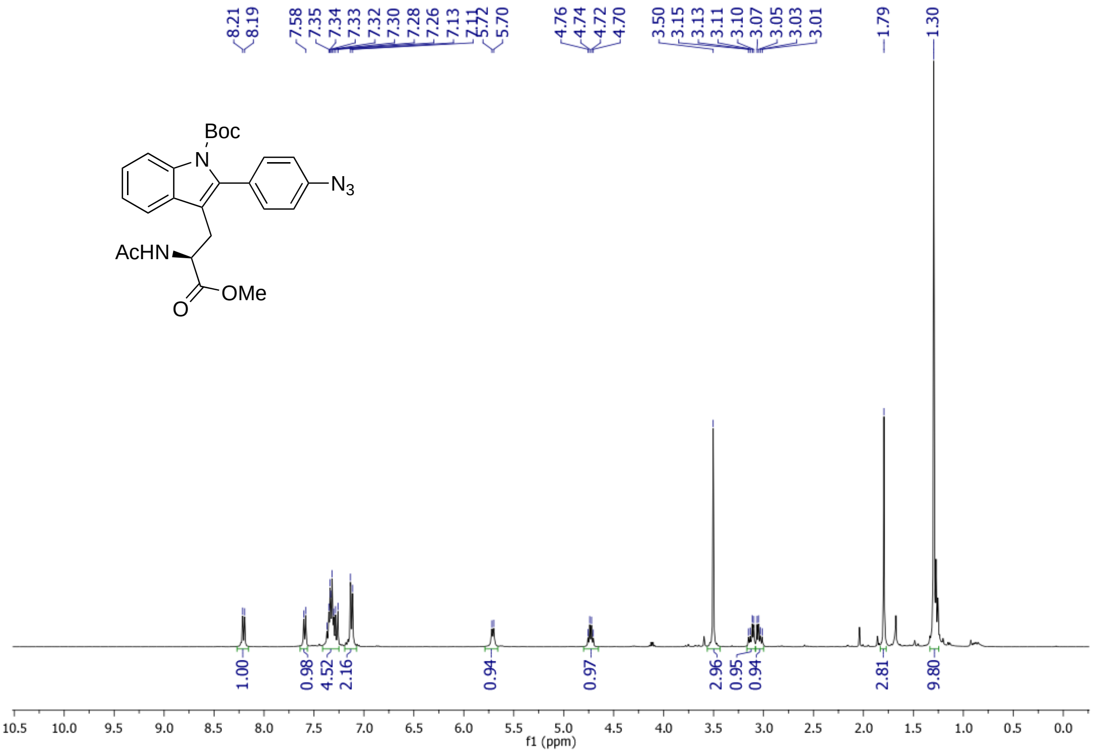

^{1}H NMR (400 MHz, CDCl$_3$) of compound **24**.

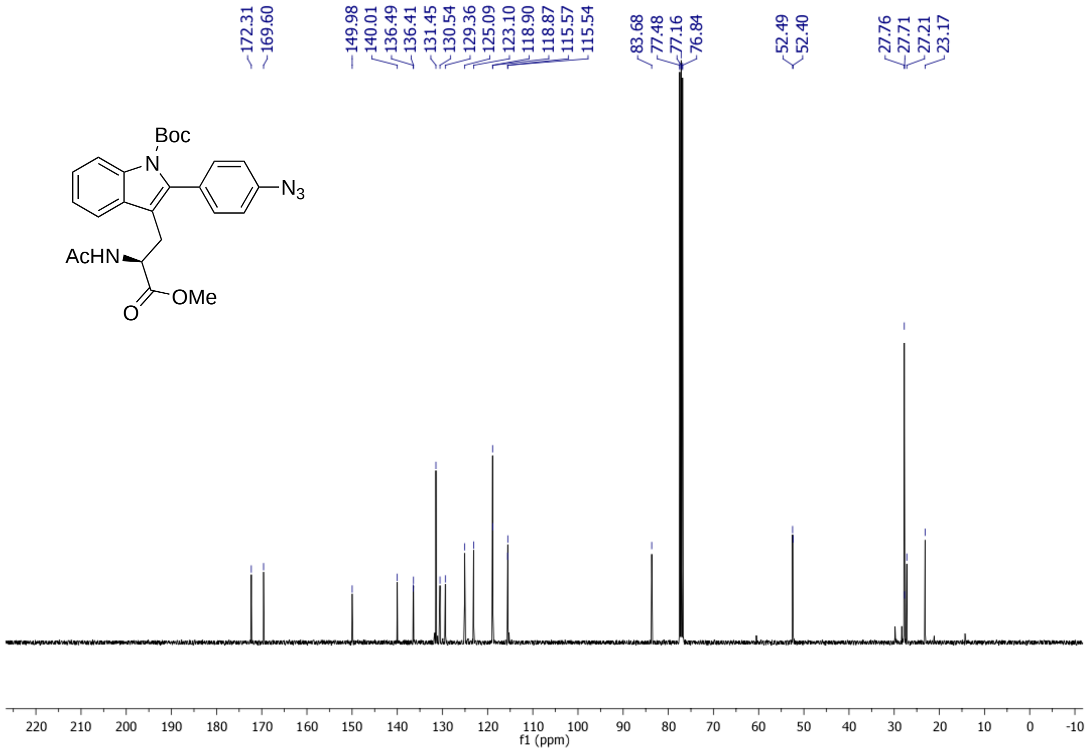

^{13}C NMR (101 MHz, CDCl$_3$) of compound **24**.

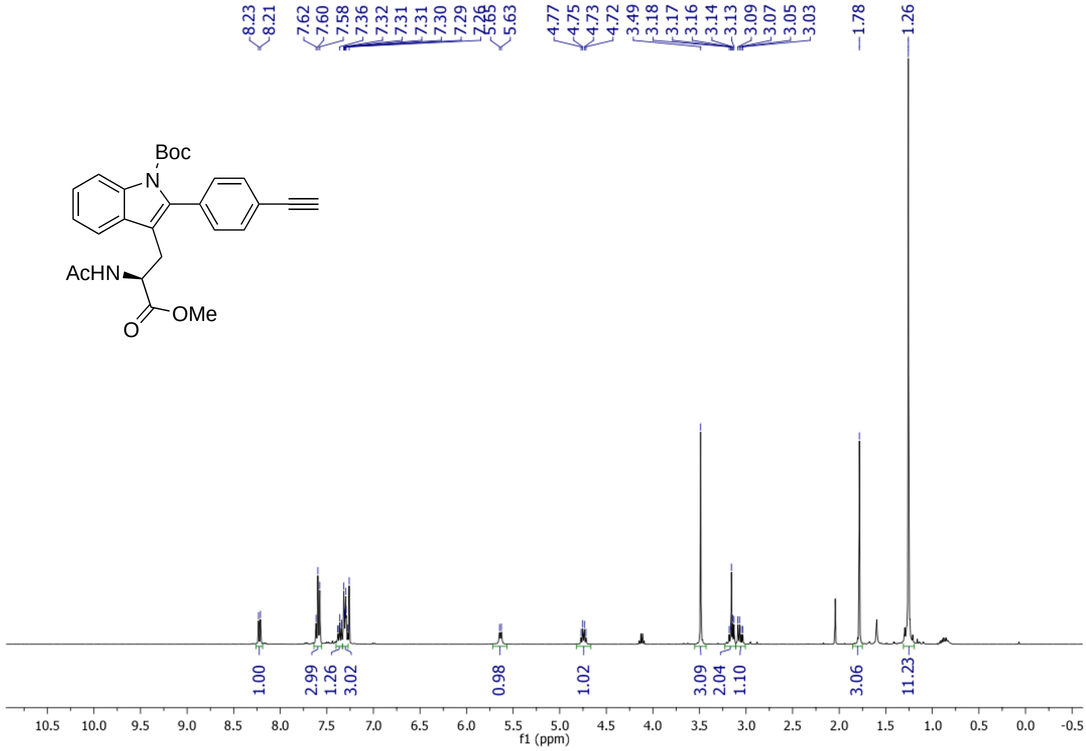

^{1}H NMR (400 MHz, CDCl$_3$) of compound **25**.

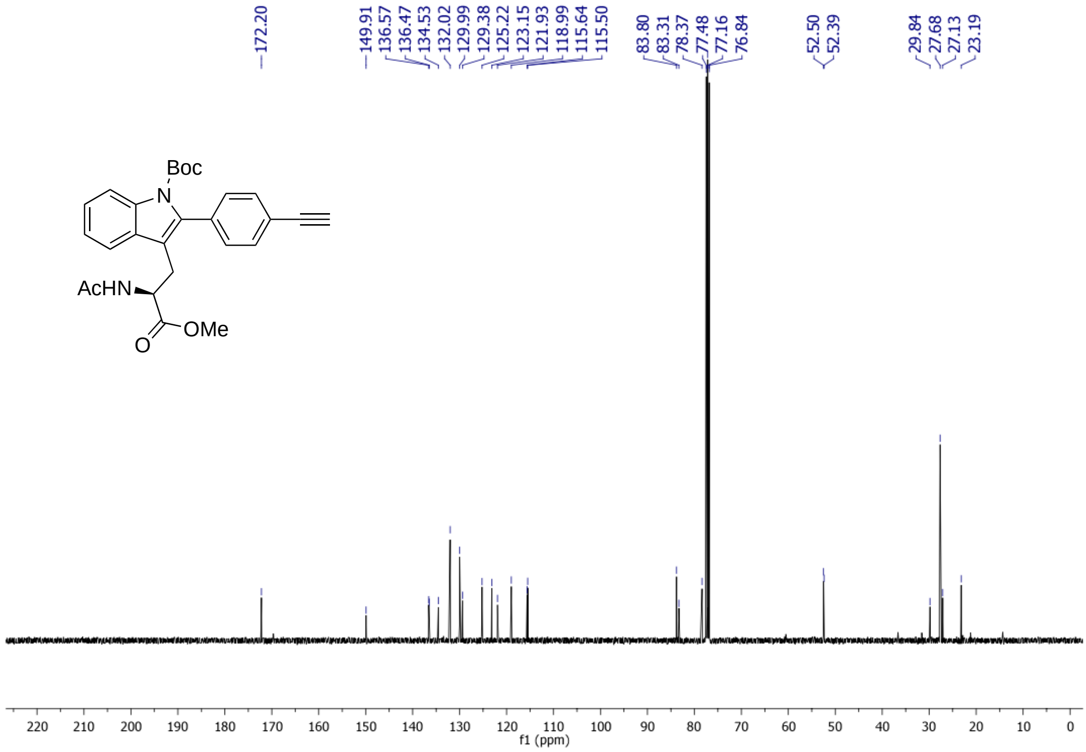

^{13}C NMR (101 MHz, CDCl$_3$) of compound **25**.

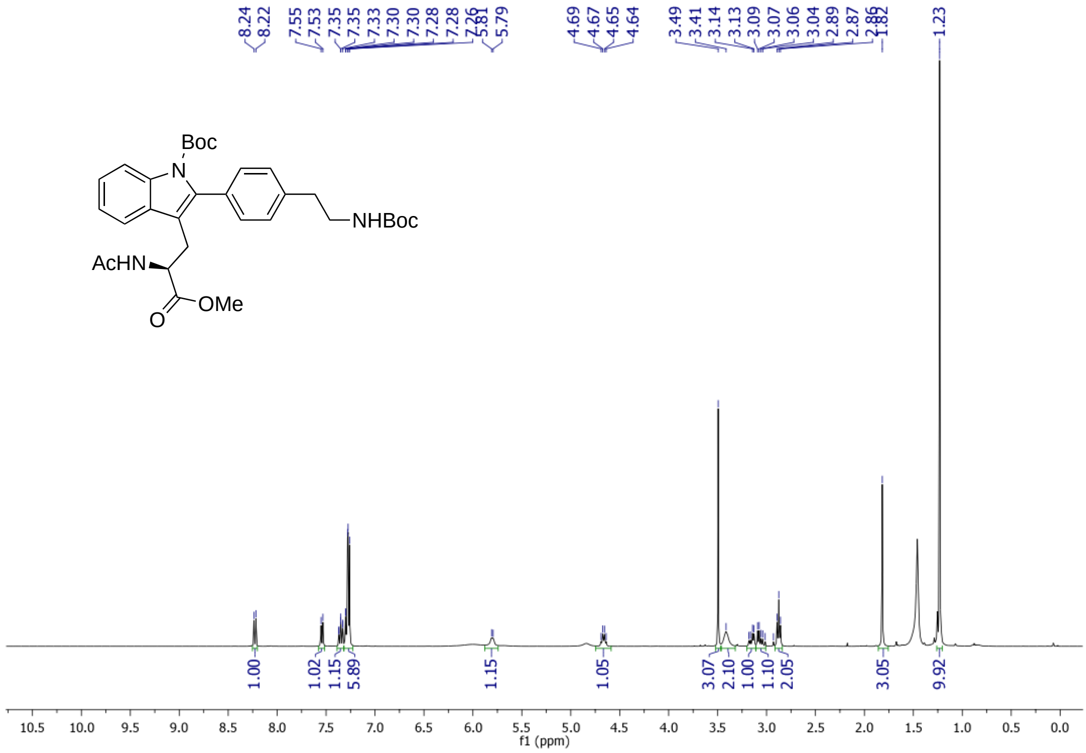

^{1}H NMR (400 MHz, CDCl$_3$) of compound **26**.

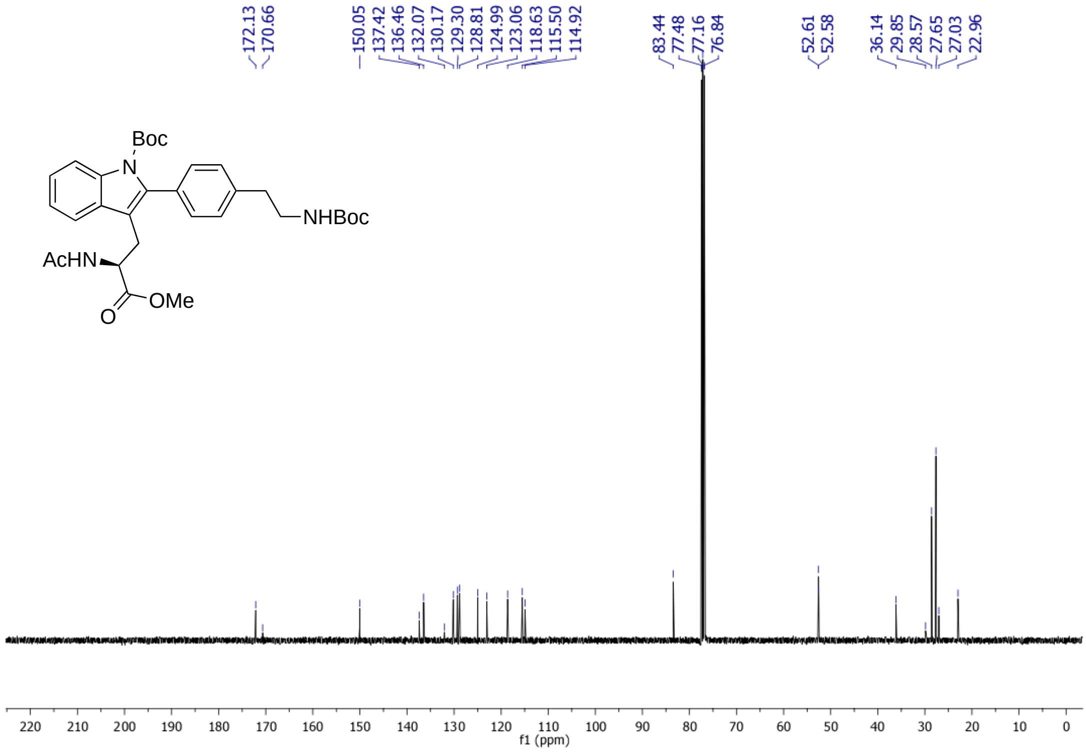

^{13}C NMR (101 MHz, CDCl$_3$) of compound **26**.

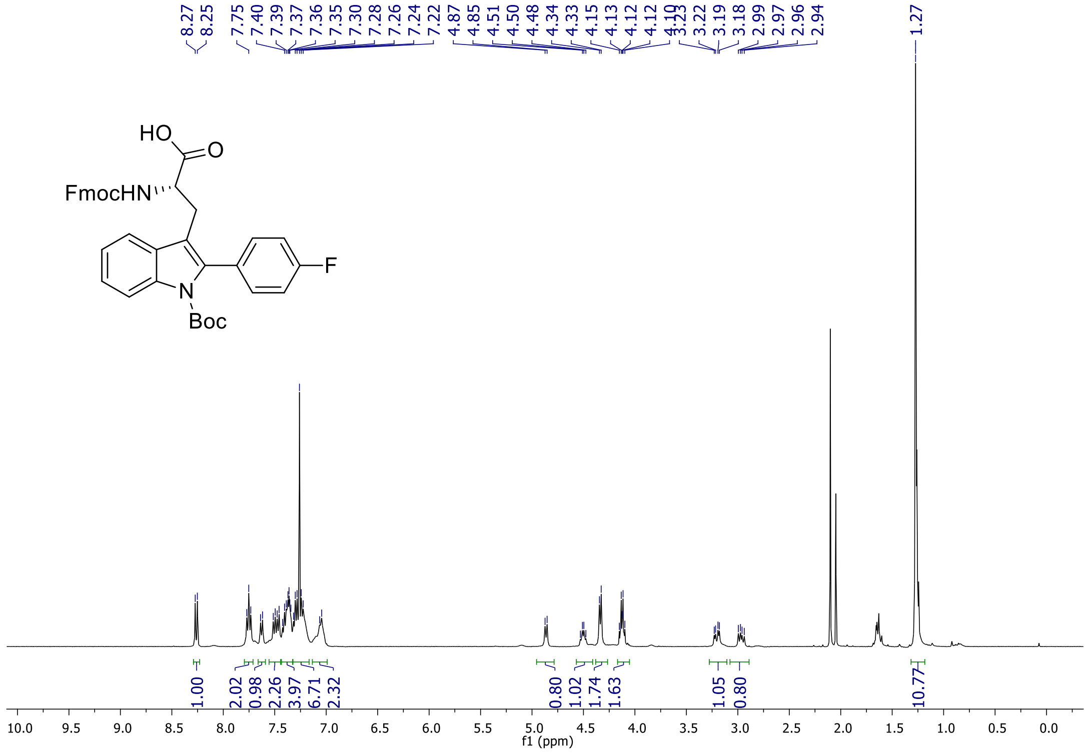

^{1}H NMR (400 MHz, CDCl$_3$) of compound **27**.

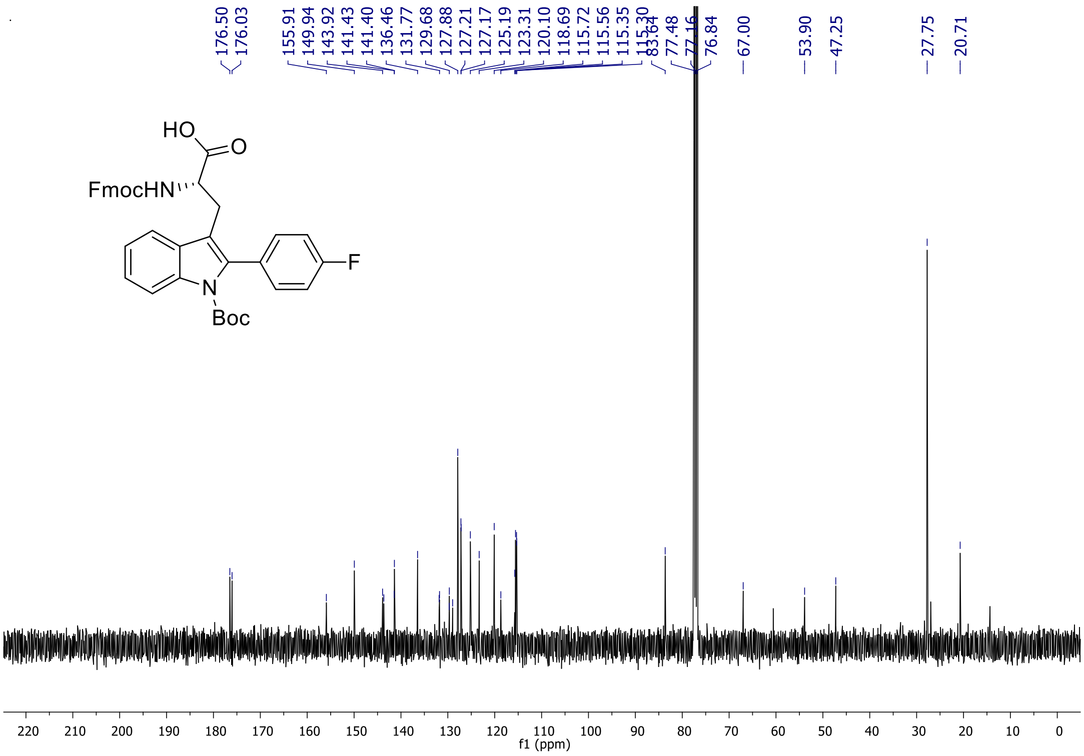

^{13}C NMR (101 MHz, $CDCl_3$) of compound **27**.

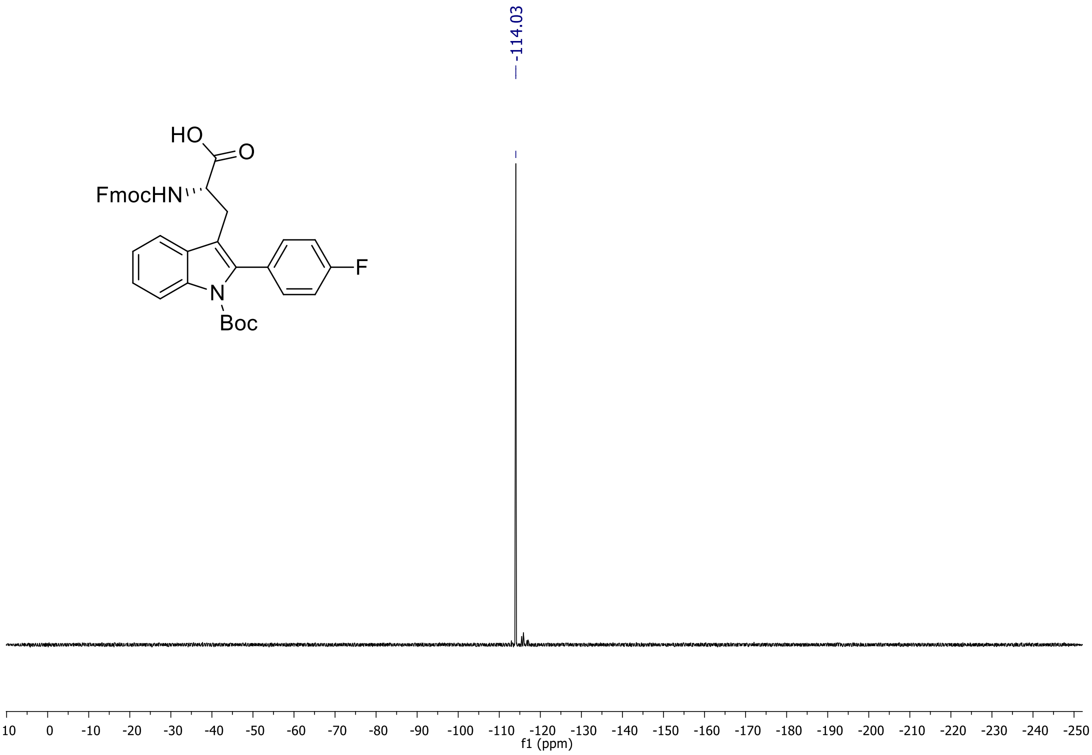

^{19}F NMR (376 MHz, $CDCl_3$) of Compound **27**.

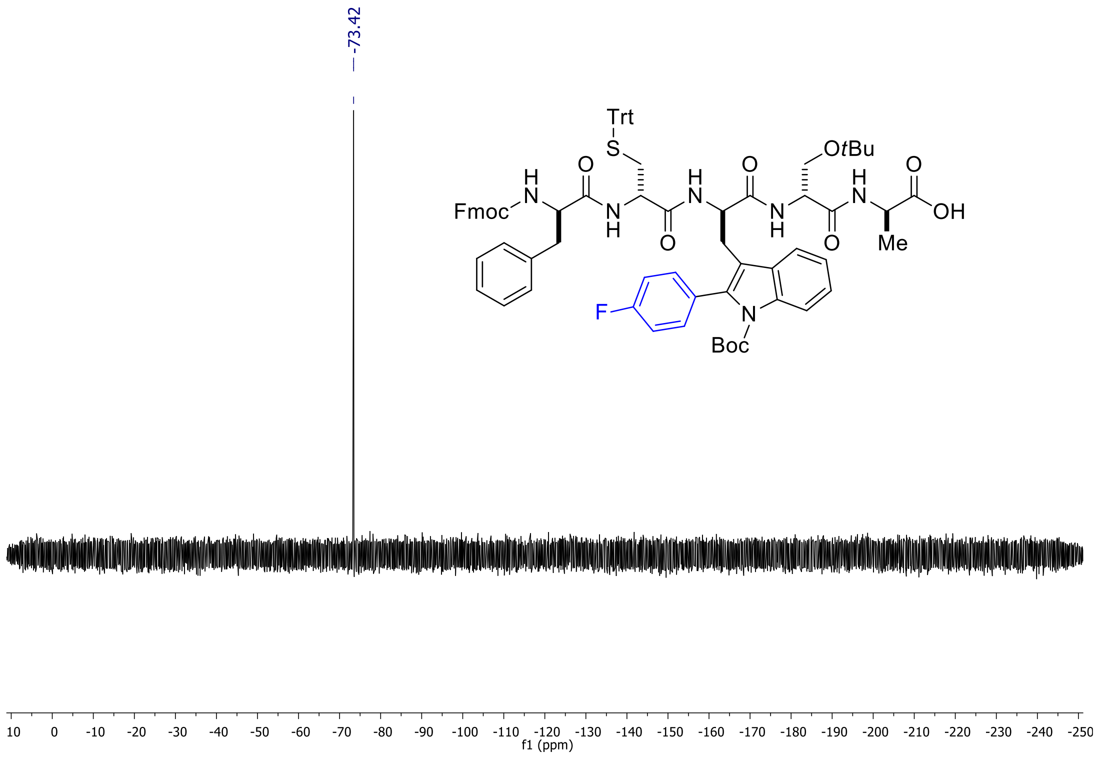

^{19}F NMR (376 MHz, DMSO-d6) of Compound **30**.

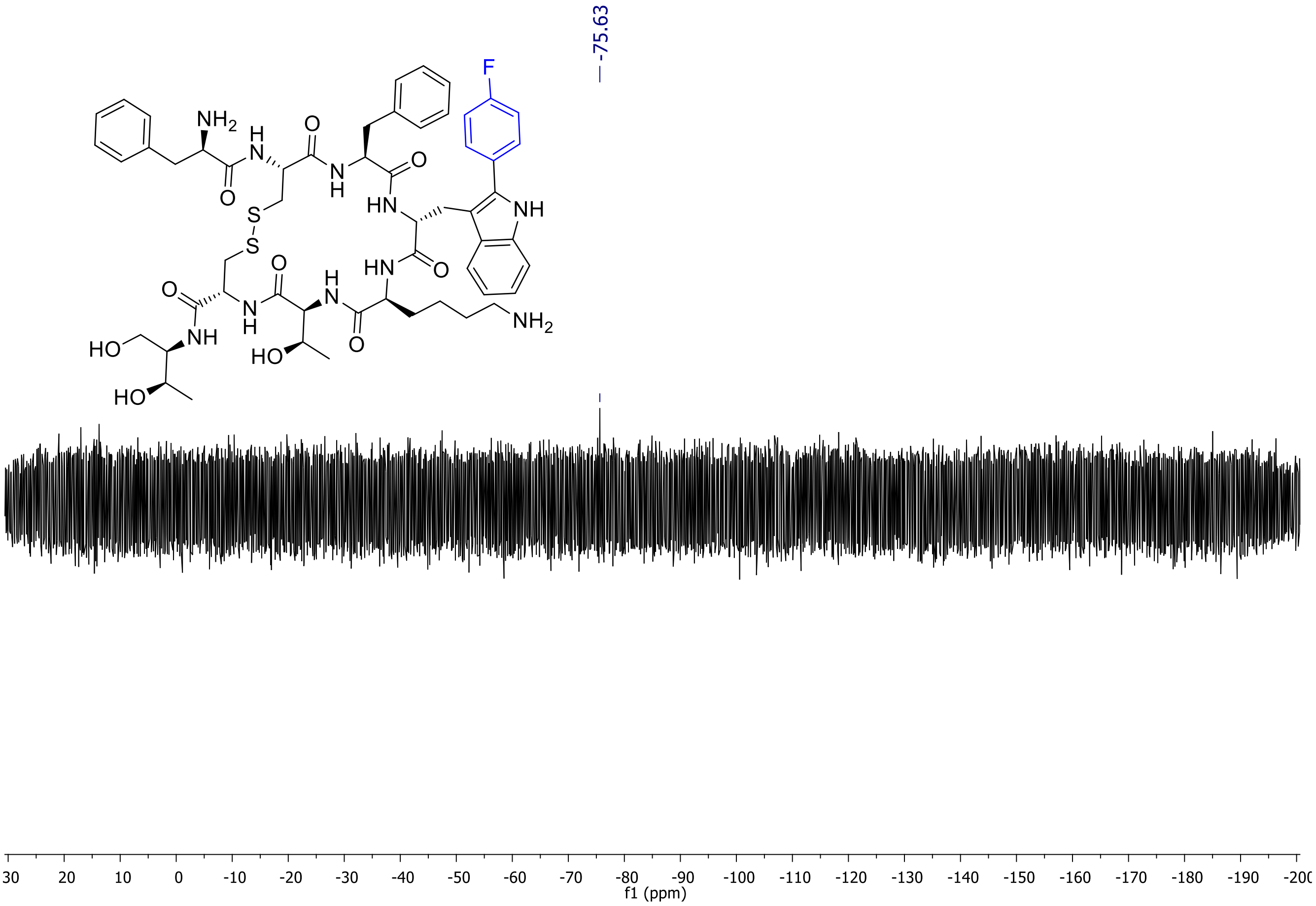

^{19}F NMR (470 MHz, D_2O) of Compound **34**.

10. References

[1] Bartolomeu, A. D. A.; Silva, R. C.; Brocksom, T. J.; Noël, T.; De Oliveira, K. T. Photoarylation of Pyridines Using Aryldiazonium Salts and Visible Light: An EDA Approach. *J. Org. Chem.* **2019**, *84* (16), 10459–10471. https://doi.org/10.1021/acs.joc.9b01879.

[2] Webb, E. W.; Cheng, K.; Wright, J. S.; Cha, J.; Shao, X.; Sanford, M. S.; Scott, P. J. H. Room-Temperature Copper-Mediated Radiocyanation of Aryldiazonium Salts and Aryl Iodides via Aryl Radical Intermediates. *J. Am. Chem. Soc.* **2023**, *145* (12), 6921–6926. https://doi.org/10.1021/jacs.3c00422.

[3] Chen, G.; Xu, J.; Xiong, B.; Song, H.; Zhang, X.; Ma, X.; Lian, Z. Copper-Catalyzed Trifluoromethylthio-Arylsulfonylation of Styrene Derivatives via the Insertion of Sulfur Dioxide. *Org. Lett.* **2022**, *24* (5), 1207–1212.https://doi.org/10.1021/acs.orglett.1c04371

[4] Gentil, S.; Rousselot-Pailley, P.; Sancho, F.; Robert, V.; Mekmouche, Y.; Guallar, V.; Tron, T.; Le Goff, A. Efficiency of Site-Specific Clicked Laccase–Carbon Nanotubes Biocathodes towards O $_2$ Reduction. *Chem. Eur. J.* **2020**, *26* (21), 4798–4804. https://doi.org/10.1002/chem.201905234.

[5] Brymora, K.; Fouineau, J.; Eddarir, A.; Chau, F.; Yaacoub, N.; Grenèche, J.-M.; Pinson, J.; Ammar, S.; Calvayrac, F. Grafting of Diazonium Salts on Oxides Surface: Formation of Aryl-*O* Bonds on Iron Oxide Nanoparticles. *J. Nanopart. Res.* **2015**, *17* (11), 438. https://doi.org/10.1007/s11051-015-3232-x.

[6] Huang, J.; Liu, F.; Zeng, L. H.; Li, S.; Chen, Z.; Wu, J. Accessing Chiral Sulfones Bearing Quaternary Carbon Stereocenters via Photoinduced Radical Sulfur Dioxide Insertion and Truce–Smiles Rearrangement. *Nat. Commun.* **2022**, *13* (1), 7081. https://doi.org/10.1038/s41467-022-34836-y.

[7] Liu, T.; Shao, X.; Wu, Y.; Shen, Q. Highly Selective Trifluoromethylation of 1,3-Disubstituted Arenes through Iridium-Catalyzed Arene Borylation. *Angew. Chem. Int. Ed.* **2012**, *51* (2), 540–543. https://doi.org/10.1002/anie.201106673.

[8] Lee, K. N.; Lei, Z.; Ngai, M.-Y. β-Selective Reductive Coupling of Alkenylpyridines with Aldehydes and Imines via Synergistic Lewis Acid/Photoredox Catalysis. *J. Am. Chem. Soc.* **2017**, *139* (14), 5003–5006. https://doi.org/10.1021/jacs.7b01373.

[9] Macleod, C.; McKiernan, G. J.; Guthrie, E. J.; Farrugia, L. J.; Hamprecht, D. W.; Macritchie, J.; Hartley, R. C. Synthesis of 2-Substituted Benzofurans and Indoles Using Functionalized Titanium Benzylidene Reagents on Solid Phase. *J. Org. Chem.* **2003**, *68* (2), 387–401. https://doi.org/10.1021/jo026384o.

[10] Özüduru, G.; Schubach, T.; Boysen, M. M. K. Enantioselective Cyclopropanation of Indoles: Construction of All-Carbon Quaternary Stereocenters. *Org. Lett.* **2012**, *14* (19), 4990–4993. https://doi.org/10.1021/ol302388t.

[11] Ruiz-Rodríguez, J.; Albericio, F.; Lavilla, R. Postsynthetic Modification of Peptides: Chemoselective *C*-Arylation of Tryptophan Residues. *Chem. Eur. J.* **2010**, *16* (4), 1124–1127. https://doi.org/10.1002/chem.200902676.

[12] Coste, A.; Toumi, M.; Wright, K.; Razafimahaléo, V.; Couty, F.; Marrot, J.; Evano, G. Copper-Catalyzed Cyclization of Iodo-Tryptophans: A Straightforward Synthesis of Pyrroloindoles. *Org. Lett.* **2008**, *10* (17), 3841–3844. https://doi.org/10.1021/ol8015513.

[13] Kuwano, R.; Kashiwabara, M. Ruthenium-Catalyzed Asymmetric Hydrogenation of *N* -Boc-Indoles. *Org. Lett.* **2006**, *8* (12), 2653–2655. https://doi.org/10.1021/ol061039x.

[14] Denmark, S. E.; Baird, J. D.; Regens, C. S. Palladium-Catalyzed Cross-Coupling of Five-Membered Heterocyclic Silanolates. *J. Org. Chem.* **2008**, *73* (4), 1440–1455. https://doi.org/10.1021/jo7023784.

[15] Ackermann, L.; Barfüßer, S.; Potukuchi, H. K. Copper-Catalyzed *N* -Arylation/Hydroamin(d)Ation Domino Synthesis of Indoles and Its Application to the Preparation of a Chek1/KDR Kinase Inhibitor Pharmacophore. *Adv. Synth. Catal.* **2009**, *351* (7–8), 1064–1072. https://doi.org/10.1002/adsc.200900004.

[16] Chakrabarty, I.; Akram, M. O.; Biswas, S.; Patil, N. T. Visible Light Mediated Desilylative C(Sp^2)–C(Sp^2) Cross-Coupling Reactions of Arylsilanes with Aryldiazonium Salts under Au(I)/Au(III) Catalysis. *Chem. Comm.* **2018**, *54* (52), 7223–7226. https://doi.org/10.1039/C8CC03925A.

[17] Ma, N.; Li, P.; Wang, Z.; Dai, Q.; Hu, C. Synthesis of Indoles from Aroyloxycarbamates with Alkynes *via* Decarboxylation/Cyclization. *Org. Biomol. Chem.* **2018**, *16* (14), 2421–2426. https://doi.org/10.1039/C8OB00086G.

[18] Farahat, A. A.; Paliakov, E.; Kumar, A.; Barghash, A.-E. M.; Goda, F. E.; Eisa, H. M.; Wenzler, T.; Brun, R.; Liu, Y.; Wilson, W. D.; Boykin, D. W. Exploration of Larger Central Ring Linkers in Furamidine Analogues: Synthesis and Evaluation of Their DNA Binding, Antiparasitic and Fluorescence Properties. *Bioorg. Med. Chem.* **2011**, *19* (7), 2156–2167. https://doi.org/10.1016/j.bmc.2011.02.045.

[19] Saejueng, P.; Bates, C. G.; Venkataraman, D. Copper(I)-Catalyzed Coupling of Terminal Acetylenes with Aryl or Vinyl Halides. *Synthesis (Stuttg)* **2005**, *10*, 1706–1712. https://doi.org/10.1055/s-2005-869893.

[20] Sun, K.; Shi, A.; Liu, Y.; Chen, X.; Xiang, P.; Wang, X.; Qu, L.; Yu, B. A General Electron Donor–Acceptor Complex for Photoactivation of Arenes *via* Thianthrenation. *Chem. Sci.* **2022**, *13* (19), 5659–5666. https://doi.org/10.1039/D2SC01241C.

[21] Murphy, J.; Bastida, D.; Paria, S.; Fagnoni, M.; Melchiorre, P. Asymmetric catalytic formation of quaternary carbons by iminium ion trapping of radicals. *Nature* **2016**, *532*, 218–222. https://doi.org/10.1038/nature17438.
